# Structure–Photoreactivity Relationship Study of Substituted 3-Hydroxyflavones and 3-Hydroxyflavothiones for Improving Carbon Monoxide Photorelease

**DOI:** 10.1021/acs.joc.4c00070

**Published:** 2024-03-22

**Authors:** Yann A. Jézéquel, Filip Svěrák, Andrea Ramundo, Vojtěch Orel, Marek Martínek, Petr Klán

**Affiliations:** † Department of Chemistry, Faculty of Science, Masaryk University, Kamenice 5, 625 00 Brno, Czech Republic; ‡ RECETOX, Faculty of Science, Masaryk University, Kamenice 5, 625 00 Brno, Czech Republic

## Abstract

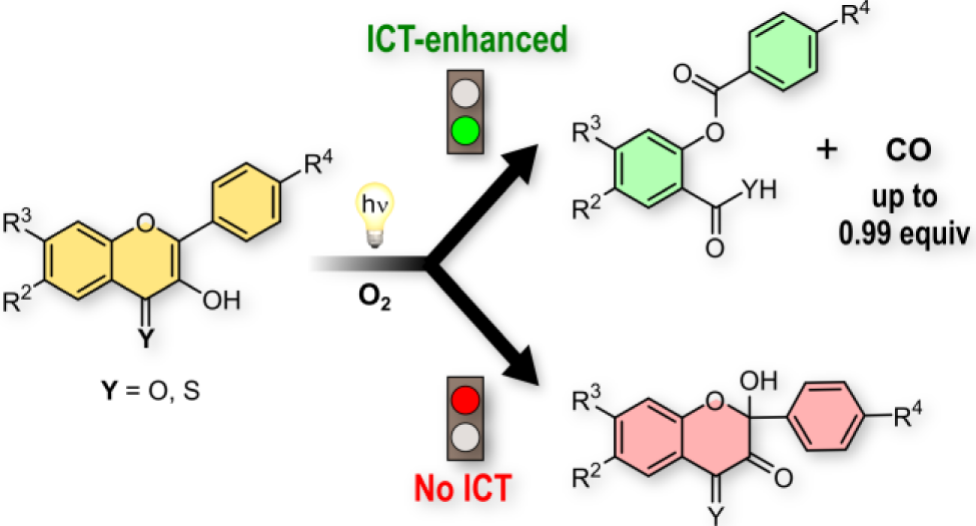

Carbon monoxide (CO) is notorious for its toxic effects but is also recognized as a gasotransmitter with considerable therapeutic potential. Due to the inherent challenges in its delivery, the utilization of organic CO photoreleasing molecules (photoCORMs) represents an interesting alternative to CO administration characterized by high spatial and temporal precision of release. This paper focused on the design, synthesis, and photophysical and photochemical studies of 20 3-hydroxyflavone (flavonol) and 3-hydroxyflavothione derivatives as photoCORMs. Newly synthesized compounds bearing various electron-donating and electron-withdrawing groups show bathochromically shifted absorption maxima and considerably enhanced CO release yields compared to the parent unsubstituted flavonol, exceeding 0.8 equiv of released CO in derivatives exhibiting excited states with a charge-transfer character. Until now, such outcomes have been limited to flavonol derivatives possessing a π-extended aromatic system. In addition, thione analogs of flavonols, 3-hydroxyflavothiones, show substantial bathochromic shifts of their absorption maxima and enhanced photosensitivity but provide lower yields of CO formation. Our study elucidates in detail the mechanism of CO photorelease from flavonols and flavothiones, utilizing steady-state and time-resolved spectroscopies and photoproduct analyses, with a particular emphasis on unraveling the structure–photoreactivity relationship and understanding competing side processes.

## Introduction

Carbon monoxide (CO) is a colorless, tasteless, and odorless gas^1^ known for its toxic properties^2^ due to competitive binding with the heme moiety of hemoglobin.^3^ CO is also produced endogenously in mammals due to heme catabolism^4^ and acts as a neural messenger^5^ and signaling molecule.^6^ There is growing interest in using CO as a therapeutic agent, for which cardioprotective,^7^ cytoprotective,^8^ and anti-inflammatory^9^ effects have been recognized. Carbon monoxide has also been considered a potential antitumor agent.^10,11^

CO-releasing molecules (CORMs) that liberate CO on demand using, for example, enzymatic^12^ or ligand-exchange activations,^13^ are developed to deliver CO to an organism in a controlled manner to avoid overexposure.^14−17^ Photoactivatable CORMs (photoCORMs) offer better spatial and temporal control of CO release. The first generation of photoCORMs was based on metal–carbonyl complexes,^18,19^ but several metal-free photoCORMs have already been developed, for example, based on cyclopropenone,^20^ BODIPY,^21^ or α-diketone derivatives.^22^ Today, special attention is paid to the development of photoCORMs that absorb in the near-infrared (NIR) range of 650–900 nm, known as the phototherapeutic window.^17,23^ Significant developments have also been made in the field of prodrugs that release CO under physiological conditions.^24−26^

**Figure 1:**
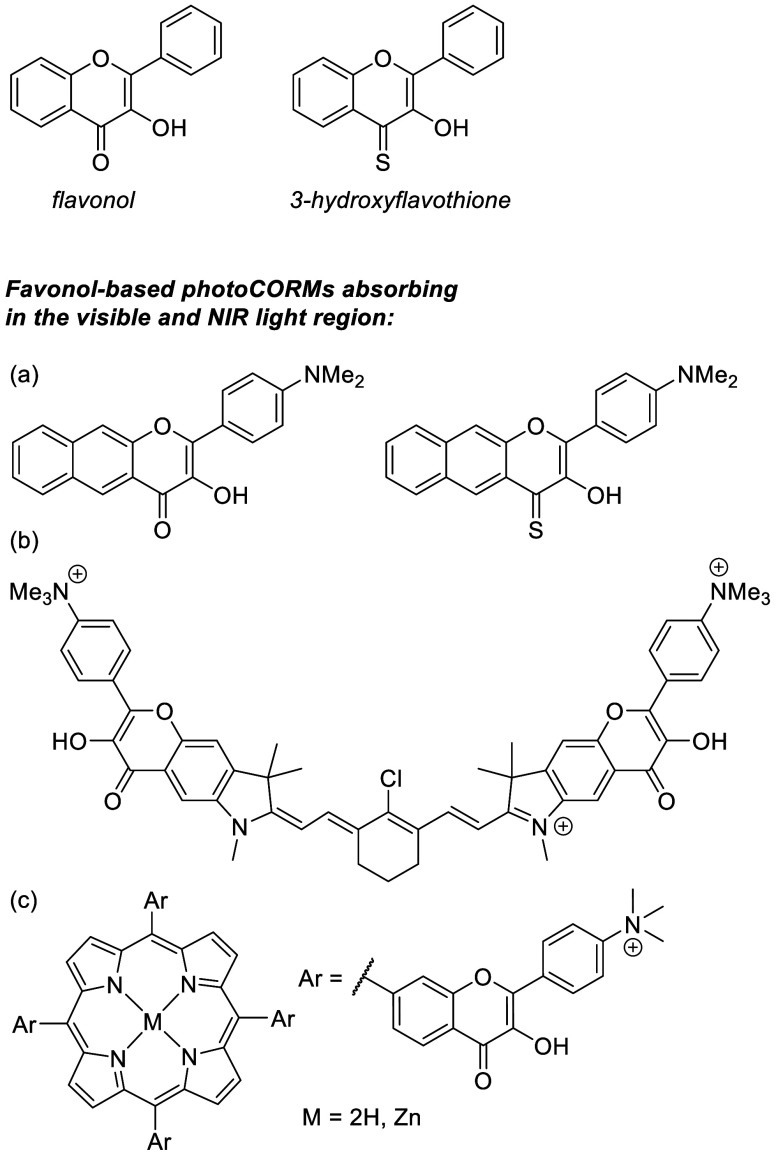
Flavonol- and 3-hydroxyflavothione-based photoCORMs: (a) ref (28), (b) ref (30), and (c) ref (31).

3-Hydroxyflavone (flavonol; Figure 1) derivatives belong to the family of flavonoids, common in all plant tissues, that possess antioxidant, anti-inflammatory, antimutagenic, and anticarcinogenic properties.^27^ PhotoCORMs based on a flavonol scaffold have already been explored for their biocompatibility and easily modifiable structure. The first report of a π-extended flavonol-based photoCORM (Figure 1a) was introduced by Berreau and co-workers, releasing nearly quantitative amounts of CO upon irradiation by blue light.^28^ Its thione analog (3-hydroxyflavothione) allowed CO release upon irradiation at longer wavelengths (Figure 1a).^29^ Recently, we reported on cyanine-flavonol^30^ (Figure 1b) and porphyrin-flavonol^31^ (Figure 1c) hybrid photoCORMs that can liberate CO upon irradiation with red to near-infrared light.

**Scheme 1:**
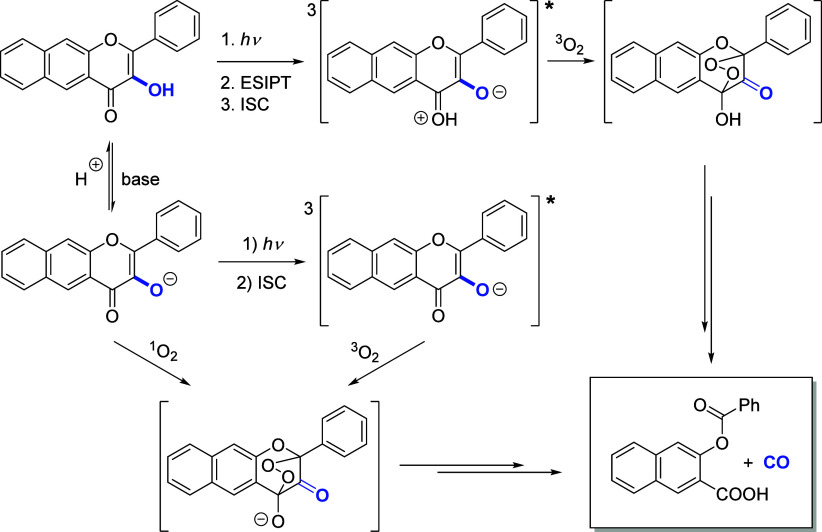
Reported Decarbonylation Mechanism for π-Extended Flavonol^34,35^

The mechanism of aerobic photodecarbonylation of flavonol derivatives^32−38^ involves a singlet excited state that undergoes rapid excited-state intramolecular proton transfer (ESIPT)^39,40^ in polar protic solvents followed by intersystem crossing (ISC) to give a triplet excited state, which reacts with triplet oxygen to release CO, possibly via an endoperoxide intermediate (Scheme 1).^34,35^ The conjugate base of flavonol liberates CO via an oxygenation reaction with singlet oxygen formed by the triplet sensitization and partially via oxidation of the triplet excited state with ^3^O_2_. Both forms also undergo inefficient photorearrangement to release CO in the absence of oxygen (not shown).

Photodecarbonylation of π-extended flavonol derivatives (Figure 1b,c) is efficient due to larger molar absorption coefficients (and thus uncaging cross section, Φ_CO_ε_max_) of bathochromically shifted absorption maxima, which is advantageous for potential biological applications. Nevertheless, these compounds are large, and their synthesis is challenging. In this work, we aimed to increase the atom economy of photodecarbonylation utilizing minimal structural modifications of the parent flavonol and 3-hydroxyflavothione. Twenty derivatives substituted by electron-donating and electron-withdrawing groups were studied using steady-state optical, time-resolved, and NMR spectroscopies and high-resolution mass spectrometry (HRMS) to provide insights into the reaction mechanism and structure–photochemical property relationships. We show that elementary structural changes can significantly improve the properties of flavonol photoCORMs.

## Results and Discussion

**Figure 2:**
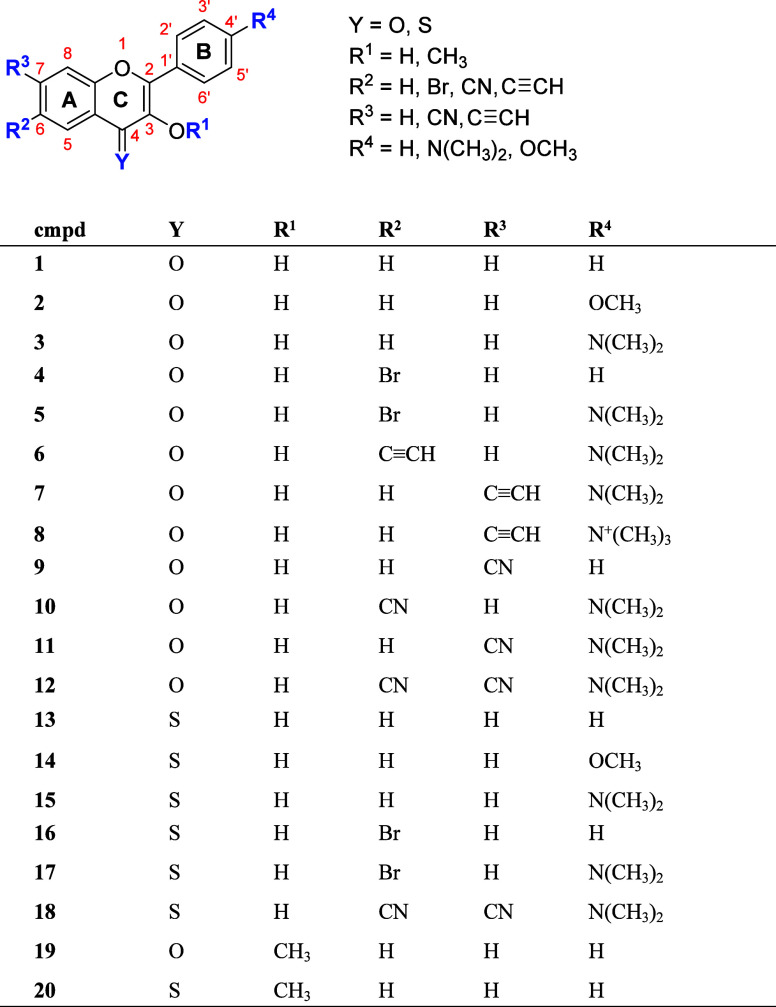
Flavones and flavothiones.

In this work, we synthesized and studied photophysical and photochemical properties of 3-hydroxyflavone (**1**) derivatives **2**–**12** and 3-hydroxyflavothione (**13**) derivatives **14**–**18**, bearing various substituents in positions C6, C7 (ring A), and C4′ (ring B), and 3-methoxyflavone (**19**) and 3-methoxyflavothione (**20**) (Figure 2) to demonstrate the effects of diverse structural features on their CO photoreleasing abilities. The main aim of this investigation was to understand the mechanism of their phototransformations using steady-state and time-resolved absorption spectroscopies and modify their structures to bathochromically shift their absorption maxima and improve the chemical and quantum yields of CO release by minimizing the formation of undesired sideproducts.

### Synthesis of 3-Hydroxyflavone Derivatives

**Scheme 2:**
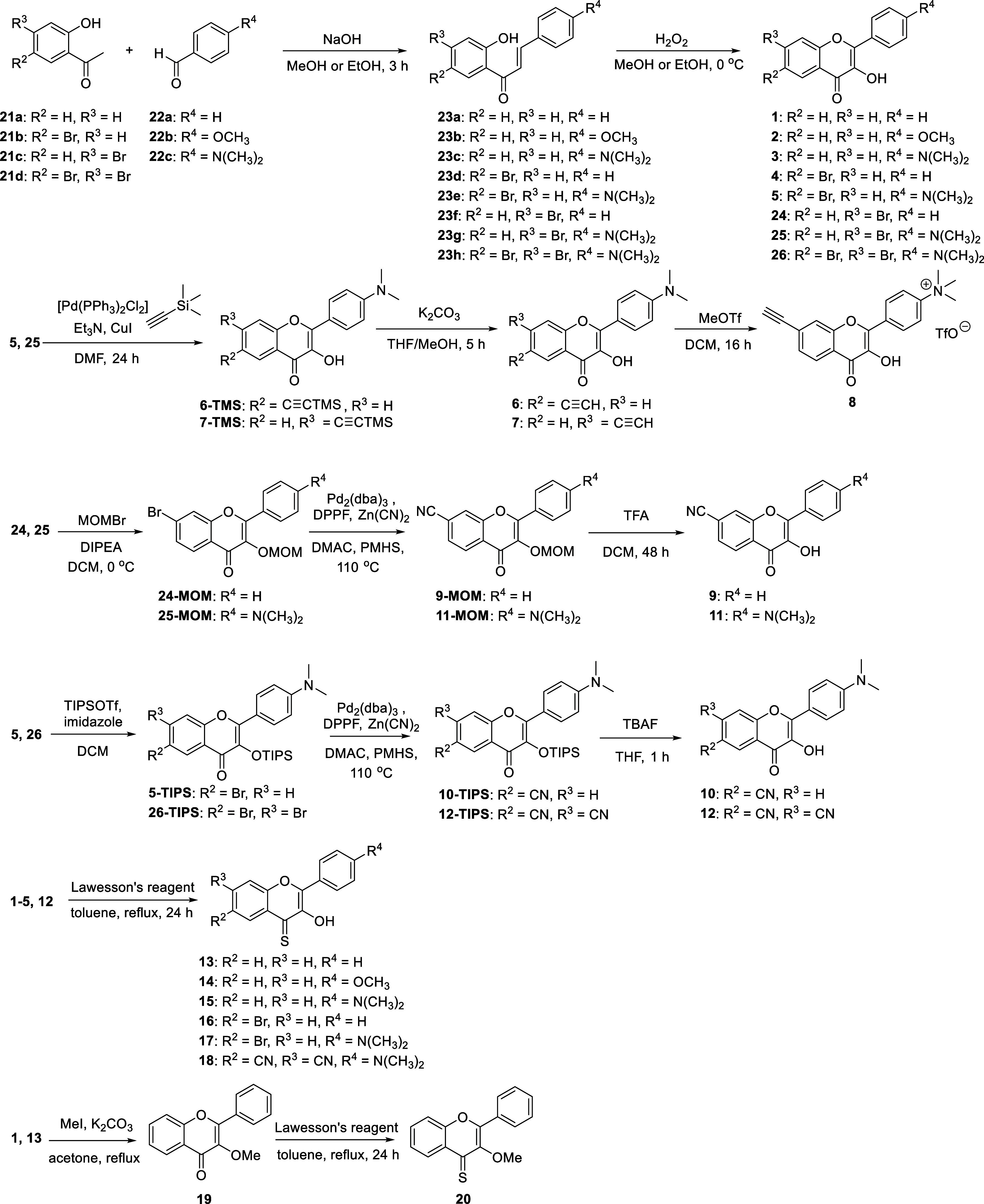
Synthesis of 3-Hydroxyflavone and 3-Hydroxyflavothione Derivatives

3-Hydroxyflavones **1**–**12** were synthesized via an aldolization reaction from commercially available 2-hydroxyacetophenone (**21a**–**d**) and benzaldehyde (**22a**–**c**) derivatives under basic conditions according to the reported methods^35,41^ to give the corresponding chalcone intermediates (**23a**–**h**, Scheme 2). The subsequent *in situ* Flynn–Algar–Oyamada condensation^42,43^ with hydrogen peroxide under basic conditions provided the title 3-hydroxyflavone derivatives (**1**–**5**). The ethynyl and nitrile groups were introduced by palladium-catalyzed coupling reactions^44,45^ from the corresponding brominated precursors to give compounds **6**, **7**, and **9**–**12**. The ability of flavonols to coordinate metal ions^46^ in these synthetic steps required the use of protecting groups. Methylation of compound **7** with methyl triflate as a methylating agent provided compound **8**. Compounds **1**–**5** and **12** were converted to 3-hydroxyflavothiones **13**–**18** using Lawesson’s reagent.^41,47^ 3-Methoxyflavone **19** was synthesized from **1** using methyl iodide and was converted to 3-methoxyflavothione (**20**) using Lawesson’s reagent.

### Physicochemical Properties

**Scheme 3:**
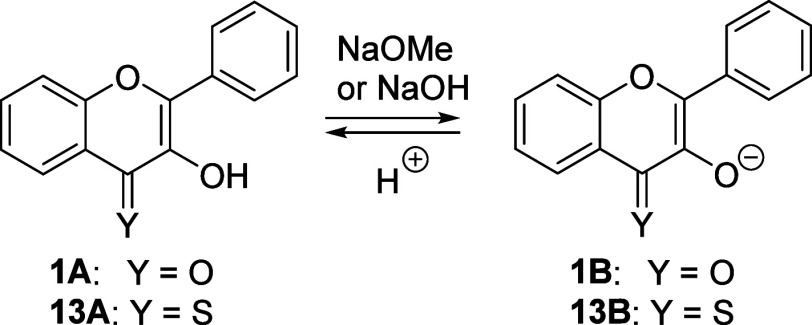
Acid–Base Forms of 3-Hydroxyflavone **1** and 3-Hydroxyflavothione **13**

3-Hydroxyflavones and their thione analogs exist in both acid (**1A**–**18A**) and base (**1B**–**18B**) forms^35^ in polar protic solvents (Scheme 3). A p*K*_a_ value of 8.7 in pure water for **1A** was reported.^48^ Because of its low solubility in water, the p*K*_a_ of 3-hydroxyflavothione (**13**) was determined in a water/methanol (50:50, v/v) mixture and recalculated to give a value of 10.07 ± 0.12 for pure water using the reported calibration method (Figure S160).^49^ Flavonols with electron-withdrawing groups (EWGs) on rings A and B (e.g., **8** and **9**) are partially deprotonated in pure methanol (Figures S108 and S110), while those with an additional electron-donating group (EDG) in position C4′ (**7** and **11**) are fully protonated (Figures S106 and S114, respectively).

The substituents considerably affect the absorption and other physical properties of hydroxyflavones and hydroxyflavothiones (Table 1). Two characteristic absorption bands of **1A** in the UV region are replaced by one major band bathochromically shifted by ∼50 nm in **3A** due to excited-state internal charge transfer (ESICT) introduced by an electron-donating 4′-dimethylamino (DMA) group (Figure S99).^50−52^ With moderately (ethynyl, **6A**, and **7A**) or more strongly (cyano, **10A**–**12A**) electron-withdrawing^53^ groups on C6 or C7, ESICT is manifested even more (Figures S104, S106, S112, S114, and S116). The absorption bands of 3-hydroxyflavothiones are bathochromically shifted^54^ compared to those of 3-hydroxyflavones; the absorption bands of base forms **1B**–**18B** are shifted by 30–80 nm for 3-hydroxyflavones and 30–70 nm for their thione analogs compared to those of the corresponding acid forms (Figures S96–S130). For example, **12B** exhibits absorption at λ_max_ = 510 nm, reaching 600 nm at the tail of the absorption band due to ESICT involving both EWGs and EDGs on rings A and B, respectively, as well as resonance effects (Figure S117).^53^ Such an absorption shift has only been possible for flavonol derivatives with a π-extended conjugated system.^30^ As a moderate EWG, the bromine atom^53^ in position C6 (**4**, **5**, **16**, **17**) does not affect the absorption spectra significantly (Figures S101, S102, S124, and S126).

**Table 1:** Physicochemical and Photochemical Properties of the Studied Flavone Derivatives^a^

cmpd	λ_max_ (nm)^b^	λ_em_ (nm)^b^	ε (M^–1^ cm^–1^)^b^	Φ_f^*,*^_^c^	CO yield (equiv)^d^	Φ_CO_^e^ (Φ_dec_)^f^	εΦ_CO_^g^
**1A**	345	403, 532	25,260	0.03^55^	0.08	(0.011 ± 0.0008)	
**1B**	405	500	21,620	0.03^56^	0.08	n.m.	
**2A**	356	436^57^	22,840^57^	n.m.	0.06	n.m.	
**2B**	419	486	19,660	n.m.	n.m.	n.m.	
**3A**^h^	402	535	30,300	0.26	0.46 ± 0.05	0.0031	94
**3B**^h^	434	542	22,400	0.17	0.57 ± 0.02	0.026	305
**4A**	350	540	13,815^58^	n.m.	0.18	(0.0147 ± 0.0001)	
**4B**	417	497	15,280	n.m.	n.m.	n.m.	
**5A**^i^	413	551	19,990	n.m.	0.82 ± 0.02	n.m.	
**5B**^i^	443	552	19,910	n.m.	0.77 ± 0.02	n.m.	
**6A**^j^	412	546	31,900	0.20	0.55 ± 0.05	0.0012 ± 0.0001	38
**6B**^j^	440	548	24,000	0.19	0.58 ± 0.07	0.049 ± 0.013	1200
**7A**^j^	418	566	32,200	0.20	0.83 ± 0.08	0.0033 ± 0.0003	110
**7B**^j^	455	577	24,800	0.21	0.89 ± 0.10	0.083 ± 0.011	2100
**8A**^j^	352	472, 553	9910	<0.02	0.04 ± 0.01	0.0016 ± 0.0004	16
**8B**^j^	430	563	9670	<0.02	0.08 ± 0.01	0.0013 ± 0.0004	13
**9A**^h^	363	560	6820	0.15	0.19 ± 0.01	0.021 ± 0.002	143
**9B**^h^	445	543	7360	0.18	0.28 ± 0.03	0.016 ± 0.003	118
**10A**^j^	420	561	35,300	0.06	0.88 ± 0.04	0.0007 ± 0.0004	25
**10B**^j^	456	574	24,700	0.07	0.86 ± 0.04	0.0254 ± 0.0045	630
**11A**^h^	435	614	26,800	<0.02	0.99 ± 0.05	0.0018 ± 0.0002	48
**11B**^h^	490	636	18,900	0.2	0.78 ± 0.03	0.0359 ± 0.0083	680
**12A**^j^	451	675	27,900	<0.02	0.84 ± 0.07	0.0004 ± 0.0001	11
**12B**^j^	510	699	17,400	0.03	0.84 ± 0.10	0.0114 ± 0.0017	200
**13A**	420	510^k^	21,700	<0.02^k^	0.29	(0.0232 ± 0.0008)	
**13B**	485	n.a	21,100	n.a.	0.06	decomposed	
**14A**	446	n.m.	37,250	n.a.	0.32	n.m.	
**14B**	498	n.m.	22,480	n.a.	0.03	n.m.	
**15A**	496	591	53,210	<0.02	0.35	(0.0168 ± 0.0006)	
**15B**	528	585	50,900	n.a.	0.06	n.m.	
**16A**	433	n.m.	25,370	n.a.	0.43	(0.0468 ± 0.0010)	
**16B**	501	n.m.	23,270	n.a.	0.02	n.m.	
**17A**^i^	513	602	11,250	<0.02	0.13	n.m.	
**17B**^i^	548	n.m.	13,840	n.a.	0.02	n.m.	
**18A**^j^	555	665	33,000	n.a.	0.04	n.m.	
**18B**^j^	615	n.a.	n.m.	n.a.	n.m.	n.m.	
**19**	300	430	21,600	0.02^l^^*,*^^59^	n.a.	n.m.	
**20**	398	n.a.	24,650	n.a.	n.a.	n.m.	

a n.m. = not measured. n.a. = below the detection limit.

b Absorption (λ_max_) and emission (λ_em_) maxima and molar absorption coefficients (ε) in methanol, unless stated otherwise; *c* = 5 × 10^–5^–1 × 10^–4^ M.

c Fluorescence quantum yields in methanol at 20 °C.

d Chemical yields (in equiv) of released CO measured by GC-headspace upon irradiation at the absorption maxima.

e Quantum yields of CO release upon irradiation at the absorption maxima.

f Quantum yields of the decomposition obtained upon irradiation at the absorption maxima.

g Uncaging cross section.

h In methanol/DMSO (90:10), *c* ≈ 5 × 10^–5^ M.

i In ethanol/DMSO (98:2), *c* ≈ 1 × 10^–4^ M.

j In methanol/DMSO (98:2), *c* ≈ 5 × 10^–5^ M.

k Measured at −80 °C.

l In ethanol, *c* ≈ 1 × 10^–6^–1 × 10^–4^ M.

Kasha and co-workers rationalized the spectroscopy of 3-hydroxyflavone (**1A**) by intramolecular ESIPT to form a singlet-excited phototautomer (Scheme 1),^39,40^ which is known to take place in less than 125 fs^60^ and possesses a lifetime of <10 ps (in ethanol).^61^ It explains why the emission spectra of 3-hydroxyflavone such as **1A** show an emission band from the locally excited state at shorter wavelengths in nonpolar solvents or a lower temperature^52^ and a new, bathochromically shifted band of the phototautomer in protic solvents such as methanol (Figure S96). On the other hand, emission spectra of 4′-dimethylamino derivatives in methanol have the bands bathochromically shifted (Table 1 and Figure S99) due to ESICT.^62^ Polar solvents stabilize a zwitterionic excited state, the energy of which may decrease to such an extent that the proton transfer (ESIPT) is no longer favorable.^51^ The compounds with electron-withdrawing cyano groups **9A**–**12A** were weakly fluorescent. The most bathochromically shifted emission maximum at 675 nm, corresponding to a very large Stokes shift of 224 nm (7358 cm^–1^), was observed for a dicyano derivative **12A**. To investigate the emissive states further, we measured the emission spectra of **1A**, **3A**, **7A**, **9A**, and **12A** in solvents of different polarities. ESIPT was dominant in flavonols without an EDG in position C4′ (**1A** and **9A**; Stokes shifts of ∼9568 cm^–1^), and the emission maxima were not affected by the solvent polarity (Figures S131 and S134). On the other hand, the derivatives exhibiting ESICT (**3A**, **7A**, and **12A**) showed a significant emission band of a charge-transfer character, which was bathochromically shifted with increased polarity of the solvent (Figures S132, S133, and S135). As reported before,^51^ these derivatives showed emission from the normal state in methanol. 3-Hydroxyflavothione derivatives (**13A**–**18A**) are either not emissive or show a very low emission^63^ (Figure S128) due to more efficient intersystem crossing related to the heavy atom effect of the sulfur atom.^64^ The observed Stokes shifts were always below 100 nm in polar methanol; thus, the emission was ascribed to the normal form emission, as also demonstrated by Chou and co-workers for **3A**.^65^ The base forms of 3-hydroxyflavones showed one emission band (Scheme 1; ESIPT is not possible) with moderate to large Stokes shifts ranging from 4073 cm^–1^ for **1B** to 5302 cm^–1^ for **12B**).

### Photochemistry of Flavonol Derivatives

Near-UV irradiation of **1A** in aerated methanol was reported to give CO and 2-(benzoyloxy)benzoic acid as a side product in relatively low yield (below 0.2 equiv; Scheme 1).^36^ We found that substituents in compounds **2A**–**12A** affected this photochemistry of flavonol significantly (Table 1). 4**′**-DMA substitution in **3A** increased CO yields to 0.5 equiv, but when combined with the cyano EWG at C6 and/or C7 positions (**10A**–**12A**), for example, the yield reached almost unity (up to 0.99 equiv for **11A**). Such high yields have only been reported in π-extended flavonols.^28,34^ The CO-photorelease enhancement was also significant in 6- (**6A**; 0.58 equiv) and especially 7-ethynyl (**7A**; 0.83 equiv) derivatives bearing the 4**′**-DMA group. On the other hand, when the electron-donating 4**′**-DMA group was replaced by trimethylammonium as an EWG in **8A**, the CO yield was negligible (no ESICT). 7-CN and 6-Br substitution alone (**9A** and **4A**, respectively) had no effect (∼0.2 equiv) but in connection with the 4**′**-DMA group in **5A** and **11A**, CO yields increased significantly. The high CO yield (∼0.8 equiv) in **5A** is attributed to the combination of ESICT and heavy atom effect, promoting the triplet excited state, which has a pivotal role in the photodecarbonylation mechanism.^36^ Interestingly, irradiating the corresponding base forms in methanol provided very similar CO yields. Quantum yields of CO release on the order of 0.001 for acid forms and 0.01 for base forms were not affected significantly by the substituents (Table 1). The corresponding uncaging cross-section values (Φ_CO_ε_max_) for the selected flavonols are listed in the same table. 3-Methoxyflavone (**19**) has a slightly hypsochromically shifted absorption maximum compared to **1A** due to the electron-donating methyl group and produces only trace amounts of CO upon irradiation (Table 1). The protection of the 3-OH group (from which CO originates; Scheme 1) by the methyl group suppresses CO release.^32^

To rationalize a significant increase in the CO yields in donor–acceptor-substituted flavonols, we irradiated representative derivatives **7A** and **12A** to compare their photochemistry with that of the parent compound **1A** and its 4′-DMA derivative **3A**. Compounds **7A** and **12A** were chosen as the most efficient photoCORMs from all studied derivatives; compound **12A** has the most bathochromically shifted absorption spectrum.

**Scheme 4:**
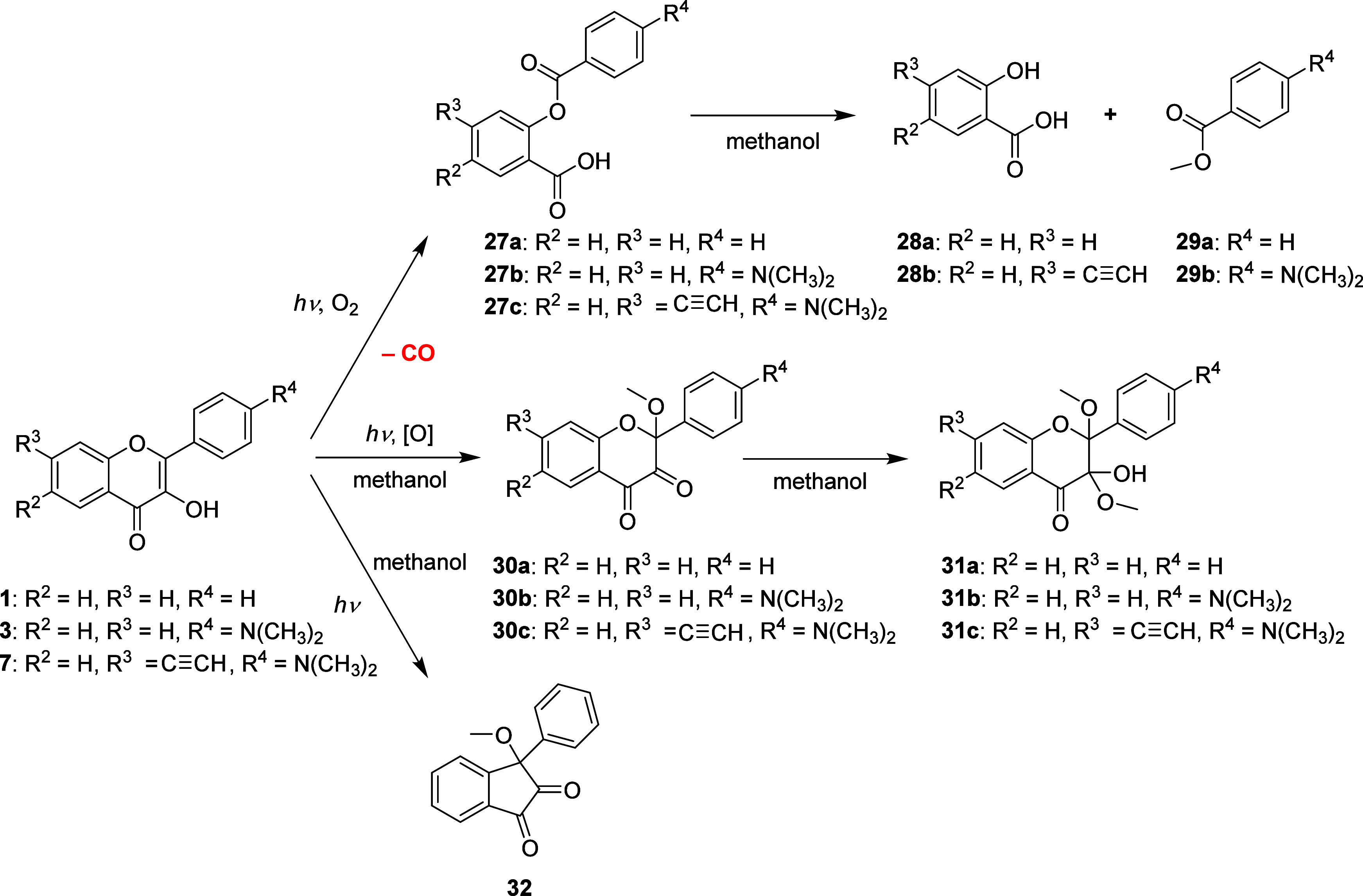
Photoproducts Detected upon Irradiation of Compounds **1A**, **3A**, and **7A**

HRMS analyses of the irradiated mixtures of **1A**, **3A**, and **7A** provided evidence for the formation of the corresponding derivatives of salicylic acid (**28a**–**b**) and methyl benzoate (**29a**–**b**) as the expected photoproducts formed during CO extrusion and subsequent methanolysis^28,33−36,66^ and two more side products, 2-methoxyflavo-3,4-dione (**30a**–**c**) and 3-aryl-3-methoxy-1,2-indandione (**32**) derivatives (Scheme 4, Figures S138–S143). For **1A**, both **30a** and **32** were identified as the main products, whereas only traces of 2-methoxyflavo-3,4-dione derivatives (**30b**–**c**) were found for **3A** and **7A** (Figures S140–S143). 2-Alkoxyflavo-3,4-diones were found to be the only identified product of quercetin irradiation in ethanol or methanol.^67^

**Figure 3:**
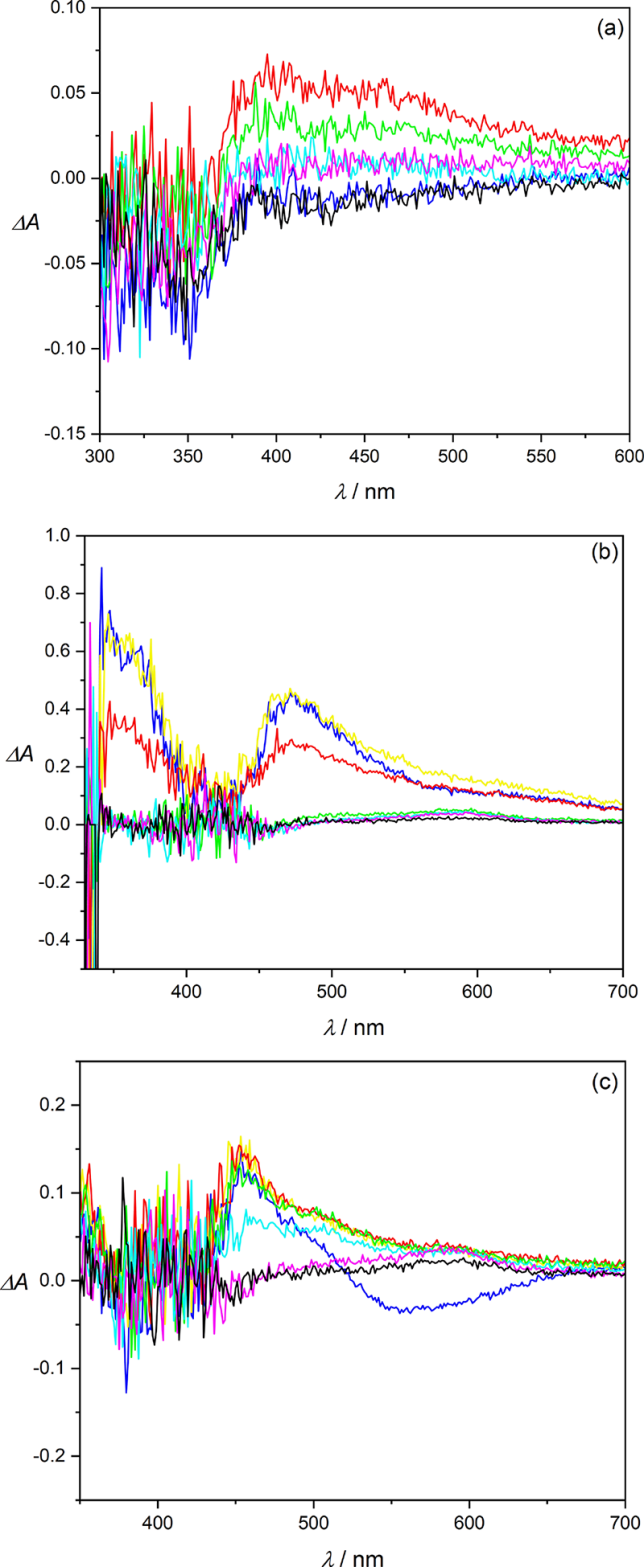
(a) Transient absorption spectra of **1A** (∼1 × 10^–5^ M, degassed methanol) taken after a 355 nm flash at various time delays (blue: 10 ns, red: 100 ns, green: 1 μs, cyan: 10 μs, magenta: 100 μs, black: 1 ms). (b) Transient absorption spectra for **7A** (∼2.5 × 10^–5^ M, degassed methanol/DMSO, 98:2) taken after a 355 nm flash at various time delays (blue: 10 ns, yellow: 50 ns, red: 100 ns, green: 1 μs, cyan: 10 μs, magenta: 100 μs, black: 1 ms). (c) Transient absorption spectra for **7A** (∼2.5 × 10^–5^ M, degassed acetonitrile) taken after a 355 nm flash at various time delays (blue: 10 ns, yellow: 50 ns, red: 100 ns, green: 1 μs, cyan: 10 μs, magenta: 100 μs, black: 1 ms). A broad negative signal (blue line) corresponds to the emission of **7A** (λ_max_ = 566 nm).

Because all photochemical processes observed in methanol were considerably suppressed in aerated solutions compared to degassed ones, we concluded that CO release originates from the triplet excited state and used nanosecond transient absorption spectroscopy to determine the lifetime of the triplet states of representative compounds **1A**, **7A**, and **12A** (Figure 3 and Figures S148–155).

Oxygen in both ground^33^ and excited^66^ states is a critical element in the photochemistry of flavonols and the production of CO and 3,4-flavandione, involving a triplet excited state as a productive common intermediate.^34,35,67^ Nanosecond transient spectroscopy of **1A** in degassed methanol showed a long-lived species possessing a lifetime of 12.5 ± 0.6 μs (Figure 3a). It is efficiently quenched by oxygen in an aerated solution (Figure S148), and we assigned it to a triplet state in agreement with previous observations.^33,35^ The transients formed from **7A** and **12A** exhibited significantly shorter lifetimes in degassed methanol (0.233 ± 0.007 and 0.071 ± 0.036 μs, Figure 3b and Figure S155, respectively) and bathochromically shifted signals. The lifetime of the former species in less polar degassed acetonitrile was found to be ∼200-fold longer (48 ± 2 μs, Figure 3c), while that of **1A** remains the same in nonpolar^33^ and polar protic solvents (Figure 3a). Because these transients were also quenched by triplet oxygen (Figures S151 and S154), we attributed them to a triplet state, possibly of a charge-/proton-transfer character.^50^ Despite their shortened lifetimes, partially due to their interaction with oxygen, oxygen is also indispensable for the oxidative formation of CO.

The shorter triplet lifetimes in the case of **7A** and **12A** correlate with the lower quantum yields of CO production and in particular with the formation of 2-methoxyflavo-3,4-dione (**30**) and 3-aryl-3-methoxy-1,2-indandione (**32**) as photoproducts (Scheme 4). The latter process involves a nucleophilic bimolecular attack of methanol, which, in principle, should be significantly suppressed when directly connected to the productive excited state. We found that the photodecomposition of these compounds in degassed methanol is very slow (Figure S136), and because it no longer competes with the oxidative production of CO, CO yield approaches 0.9 equiv (Table 1).

Self-sensitized photo-oxygenation is an important channel of CO production from flavonol base forms, while acid forms were reported to be unreactive.^35^ We determined the role of singlet oxygen in CO release from **7** and **12**. Photochemical CO yields (Table 2) from derivatives **1**, **3**, **7**, and **12** in their acid and base forms in the presence of singlet oxygen traps (NaN_3_ or furfuryl alcohol^68,69^) were found to be essentially the same or similar to those obtained in their absence. On the other hand, the affinities of **7** and **12** in their acid and base forms toward singlet oxygen (Table 2) are similar to or even higher than those reported for flavonol derivatives with a π-extended aromatic system.^34^ We concluded that the amount of singlet oxygen produced by the compounds is insufficient (quantum yield of singlet oxygen production (Φ_Δ_) is ∼1 × 10^–2^ for both forms) to compete with the reaction of triplet state with triplet oxygen (Scheme 1).

**Table 2:** Oxygenation by Singlet Oxygen^a^

cmpd	*k*_SO_/M^–1^ s^–1^^b^	Φ_Δ_^c^	CO yield/equiv^d^	CO yield/equiv^e^
**1A**^f^	1.42 × 10^6^	0.022	0.07 ± 0.01	0.074 ± 0.001
**1B**^f^	1.64 × 10^9^	n.m.	0.051 ± 0.001	0.018 ± 0.001
**3A**^g^	1.36 × 10^6^	n.m.	0.46 ± 0.02	0.41 ± 0.02
**3B**^g^	5.19 × 10^8^	n.m.	0.51 ± 0.05	0.48 ± 0.01
**7A**^h^	5.87 × 10^6^	0.0063	0.72 ± 0.03	0.78 ± 0.02
**7B**^h^	5.07 × 10^8^	0.013	0.79 ± 0.06	0.76 ± 0.04
**12A**^h^	2.91 × 10^6^	0.0044	0.82 ± 0.05	0.84 ± 0.13
**12B**^h^	n.m.	0.0077	0.83 ± 0.04	0.85 ± 0.11

a n.m. = not measured.

b Bimolecular rate constant of the reaction of a flavonol derivative with ^1^O_2_ produced by rose bengal sensitization (*c* = 1.0 × 10^–5^ M; irradiated at 572 nm) or methylene blue (for **12A**; *c* = 1.5 × 10^–5^, irradiated at 650 nm).

c Quantum yields of singlet oxygen production.

d Chemical yields of released CO upon irradiation at the absorption maxima in the presence of furfuryl alcohol (*c* ≈ 10 mM, ∼200 equiv; measured by GC-headspace).

e Chemical yields of released CO upon irradiation at the absorption maxima in the presence of NaN_3_ (*c* ≈ 10 mM, ∼200 equiv; measured by GC-headspace).

f In aerated methanol, *c* ≈ 5 × 10^–5^ M.

g In methanol/DMSO (90:10), *c* ≈ 5 × 10^–5^ M.

h In methanol/DMSO (98:2), *c* ≈ 5 × 10^–5^ M.

### Photochemistry of Flavothione Derivatives

**Figure 4:**
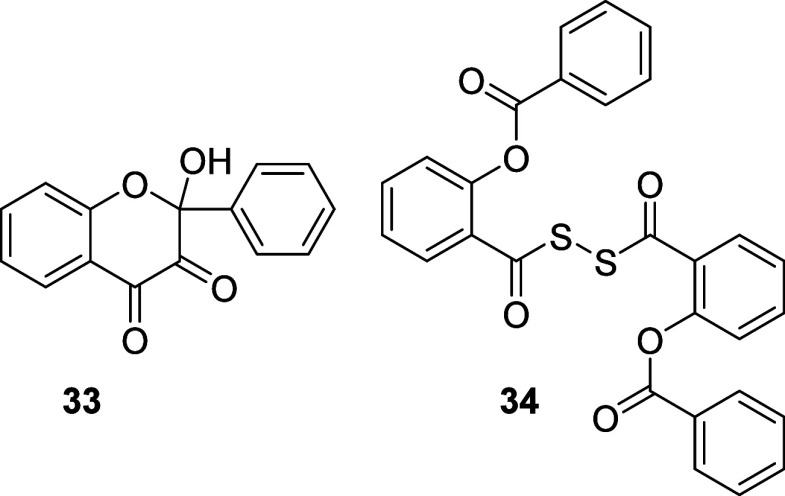
Main photoproducts of 3-hydroxyflavothione (**13A**) photodegradation.

Irradiation of the acid form of unsubstituted flavothione **13A** in aerated methanol at 420 nm (*c* ≈ 1.0 × 10^–4^ M; Φ_dec_ = 0.0232 ± 0.008) resulted in the formation of a mixture of photoproducts possessing absorption below 400 nm, 2-hydroxy-3,4-flavandione (**33**) and 2-(benzoyloxy)benzoic dithioperoxyanhydride (**34**) and carbon monoxide (Figures 4 and Figures S144 and S145; Table 1). In addition, the product of the thione → carbonyl conversion, 3-hydroxyflavone (**1A**), was found in trace amounts. (It is important to note that the photodegradation of all 3-hydroxyflavothione derivatives led to many other unidentified minor compounds (LC-HRMS, Figure S144), formed probably via secondary (photo)oxygenation processes.) Because **33** and **34** are the products of oxidation, the effect of O_2_ on the reaction was investigated. The photodegradation efficiency of **13A** was much lower in a pump-freeze-degassed methanolic solution, suggesting that oxygen is again essential for the transformations (Figure S137).

Flavothione derivatives **13A**–**17A** produced 0.1–0.4 equiv of carbon monoxide, which is up to 3 times lower than for analogous 3-hydroxyflavones (Table 1). An electron-donating group in position 4′ had only a small effect on the CO production yields. The bromine substituent in position 6 in 6-bromo-3-hydroxyflavothione (**16A**; 0.43 equiv) increased CO yield compared to that of **13A**, while a very low CO yield (0.13 equiv) was observed for 6-bromo-4′-DMA derivative **17A**. The photodegradation efficiency of **13A** was concomitant with that of CO production; therefore, CO was not formed in a secondary process. 6,7-Dicyano substitution, which enhanced the CO yield in the case of **12A**, resulted in poor yields for **18A** (0.04 equiv). (Note: the decomposition quantum yields of thiones were larger than those of the corresponding flavonols; Table 1.)

Nanosecond transient absorption (ns TA) spectroscopy of **13A** showed a long-lived species (λ_max_ ≈ 500 nm; Figure S158) with a lifetime of ∼110 ns in an aerated solution, which extends 40-fold to 5.2 μs in a degassed sample (Figure S159). Elisei and co-workers assigned this signal to a triplet excited state,^70^ which is not fully quenched by oxygen due to its relatively short lifetime. Still, the singlet oxygen production by 3-hydroxyflavothione is very high (Φ_Δ_ = 0.56^70^). Thus, the potential dual role of oxygen as a triplet quencher and oxidizing agent, facilitated in the later steps of the mechanism, was further explored. The photodegradation of **13A** in the presence of furfuryl alcohol and dimethyl furan used as a singlet oxygen trap did not change the CO yield or the degradation kinetics. Interestingly, all protonated flavothiones (**13A**–**17A**), bearing less electronegative sulfur, were found to be inert to singlet oxygen generated by the thermal decomposition of naphthalene 1,4-endoperoxide in the dark (Figure S161).

The photochemistry of **13B**–**17B** was more complicated to study because of their poor stability in the dark (Figure S163) and higher sensitivity to ambient light. For **13B**, photodegradation products were not fully identified but were different from those found in the case of **13A** (Figure S146). Unlike the acid forms, conjugate bases **13B**–**17B** react with singlet oxygen (generated from naphthalene 1,4-endoperoxide) to give the same photoproducts as those observed during direct irradiation (Figure S147). The presence of α-terpinene (1000 equiv, *c* ≈ 6.0 × 10^–2^ mol L^–1^) as a singlet oxygen trap during the photodecomposition of **13B** did not interfere with the photoreaction or with the nature of the photoproducts; thus, ^1^O_2_ did not play any significant role in photodegradation.

**Scheme 5:**
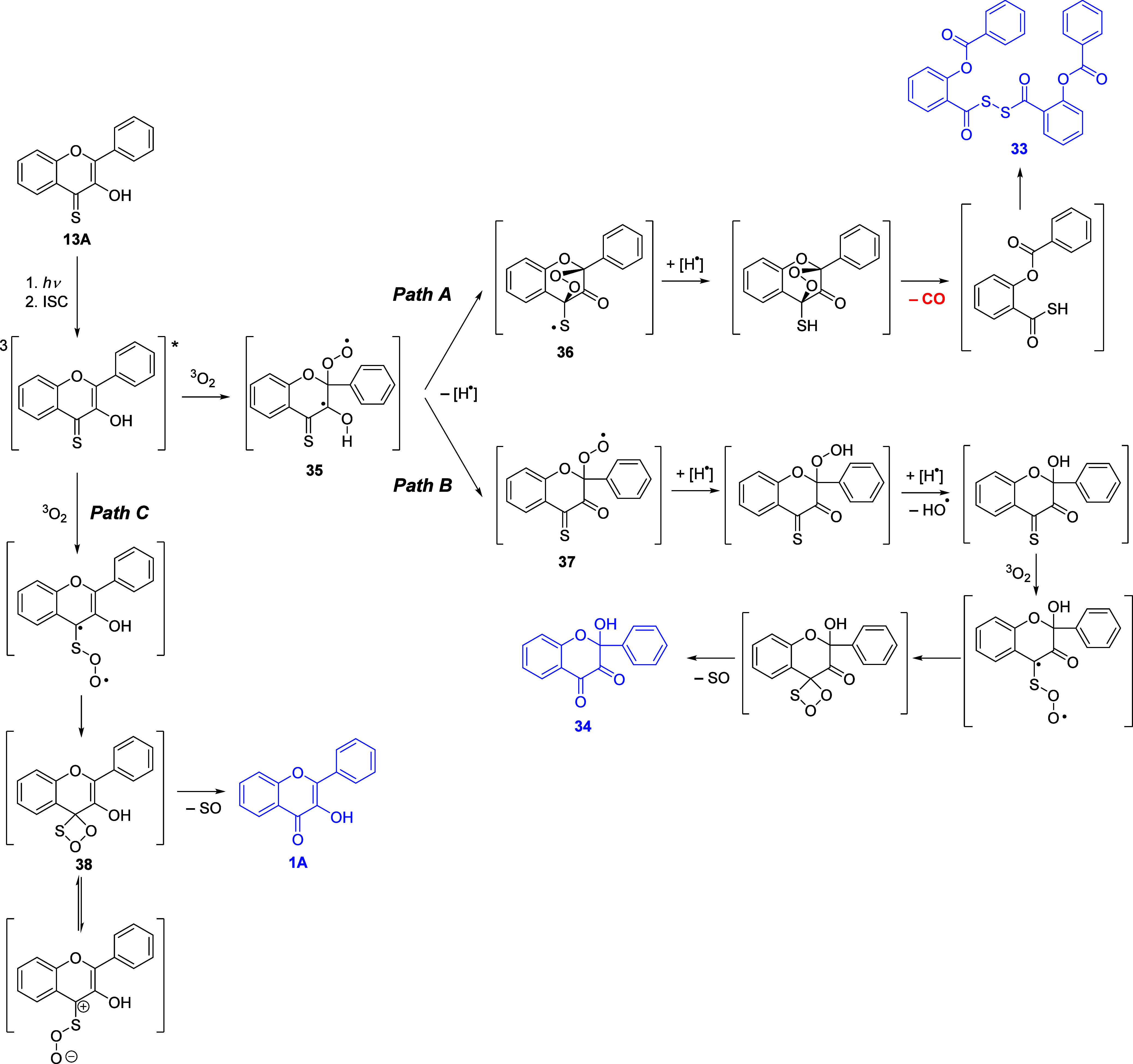
Proposed Photodegradation Mechanism of 3-Hydroxyflavothione (**13A**)

3-Hydroxyflavothione (**13A**) photodegradation occurs through the triplet excited state (Scheme 5), with a quantum yield of the triplet-state formation close to the unity.^70^ This triplet excited state reacts with triplet oxygen to form an adduct or sensitize it to singlet oxygen (Φ_Δ_ = 0.56 in ethanol^70^). Our experiments with singlet oxygen traps confirmed the absence of any significant involvement of singlet oxygen in the primary photochemical steps. This is unusual behavior for thiones, which are often reported to react with electrophilic ^1^O_2_ via its addition to the thione bond to form sulfine or ketone.^71,72^

Based on our photoproduct analyses and kinetic and quenching experiments described above, we propose that the triplet excited state of **13A** in methanol reacts via three pathways. Two of them (*paths A* and *B*, Scheme 5) share a common intermediate **35** formed by the reaction of the triplet excited state of **13A** with triplet oxygen.

In *path A*, the intermediate is converted to endoperoxide **36** that decarbonylates and forms unstable 2-(benzoyloxy)benzothioic *S*-acid, which is air-oxidized to 2-(benzoyloxy)benzoic dithioperoxyanhydride (**33**). This reaction is common for thioacids^73^ and analogous to the reported decarbonylation mechanism of 3-hydroxyflavone and their derivatives.^28,35,37^

*Path B* starts with **35**, which forms exoperoxide **37**, analogous to that suggested for 3-hydroxyflavone photodegradation.^33^ This exoperoxide undergoes autoxidation^74^ cleavage, turning the peroxide into the hydroxy group. The resulting intermediate, 2-hydroxy-2-phenyl-4-thioxochroman-3-one, undergoes another oxidation step on the sulfur atom with triplet oxygen via a 1,2,3-thiadioxetane intermediate,^75^ analogous to the reactions between thiones and ^1^O_2_.^71,72^ This mechanism finally leads to the formation of 2-hydroxy-3,4-flavandione (**34**), identified as a final product. 2-Hydroxy-3,4-flavandione derivatives have already been reported in the literature as the products obtained from 3-hydroxyflavone derivatives by chemical oxidation^76^ and photolysis.^67^ Photodegradation of **13A** in methanol in the presence of 5% labeled water D_2_(^18^O) did not show any sign of integrating the ^18^O atom into the structure of **34**.

*Path C* consists of the direct attack of triplet oxygen to the thione group of **13A** in the triplet excited state to give a 1,2,3-thiadioxetane intermediate **38** (shared by *path B*) and the subsequent loss of sulfur monoxide to yield to 3-hydroxyflavone (**1A**). This is a minor pathway, giving only traces of flavone. We initially assumed that **34** is produced by the degradation of **1A**. However, irradiation of a mixture of **13A** and **1A** (1:1) showed that the reaction of **13A** is not affected by the presence of **1A**; thus, we dismissed this assumption.

Photodegradation of 3-methoxyflavothione (**20**) produced only trace amounts of CO and gave **19**, probably via *path C*. Inertness of **20** toward the singlet oxygen attack confirmed that desulfuration in *paths A* and *B* involves the reaction of the triplet excited state with triplet oxygen. Sulfur monoxide (SO) is a probable side product of thione oxidation (*paths B* and *C*) formed upon photo-oxidation of thiones.^71,72,77^ Unfortunately, its short lifetime and high reactivity make its detection difficult.^78^

## Conclusions

Twenty 3-hydroxyflavone and 3-hydroxyflavothione derivatives were synthesized and subjected to a detailed investigation of their photophysical and photochemical properties. The strategically placed electron-donating and electron-withdrawing groups were used to fine-tune the charge-transfer character of the flavonol excited state, which showcases bathochromically shifted absorption bands up to 600 nm, high CO liberation yields (up to 0.99 equiv), and uncaging cross-section values. Such results, previously observed only for π-extended derivatives, were achieved for the simple (parent) 3-hydroxyflavone skeleton, which is easily functionalized and shows a significantly improved atom economy of CO release. Although 3-hydroxyflavothione substituents improved CO yields, its modification never reached the qualities of 3-hydroxyflavone as a photoCORM platform. With the considerably improved photoCORM properties and detailed understanding of the CO release mechanism, we demonstrate here that derivatives of 3-hydroxyflavone are a promising and potentially useful alternative to the more complex, π-extended flavonol derivatives.

## Experimental Section

### Synthesis

#### 1-(4,5-Dibromo-2-hydroxyphenyl)ethan-1-one (**21d**)

Anhydrous ZnCl_2_ (20 g, 146.8 mmol, 2.10 equiv), 4′-bromo-2′-hydroxyacetophenone (15 g, 69.8 mmol, 1.00 equiv), and CS_2_ (220 mL) were added to a 1 L round-bottom flask, and the mixture was cooled with an ice bath under strong magnetic stirring. After 15 min, Br_2_ (12 g, 75.0 mmol, 1.07 equiv; dissolved in 10 mL of CS_2_) was added, and the temperature was raised to 23 °C. The mixture was stirred for 5 days. Aqueous Na_2_S_2_O_3_ solution (5%; 200 mL) was poured into the mixture, and the organic phase was let to separate. The aqueous phase was extracted with CH_2_Cl_2_ (3 × 50 mL), and the combined organic phases were collected and dried over Na_2_SO_4_. The solvents were evaporated in vacuo, and the solid residue was washed with *i*-Pr_2_O (2 × 20 mL) and dried under reduced pressure. The crude product was recrystallized from hot acetone. Yield: 13.6 g (66%). Off-white crystals (dec < 82 °C). ^1^H NMR (500 MHz, CDCl_3_, Figure S52): δ (ppm) 12.13 (s, 1H), 7.95 (s, 1H), 7.34 (s, 1H), 2.63 (s, 3H). ^13^C NMR (125 MHz, CDCl_3_, Figure S53): δ (ppm) 203.1, 161.2, 134.4, 133.0, 123.8, 120.1, 113.4, 26.7. HRMS (APCI–, Figure S89): calcd. for C_8_H_5_Br_2_O_2_^–^ [M – H^+^]: 290.8662, found: 290.8664. This compound has also been characterized elsewhere.^79^

### General Procedure for the Synthesis of 3-Hydroxyflavone Derivatives **1–5**, **24**, and **25**

2-Hydroxyacetophenone derivative **22a**–**d** (5.00 g, 1.00 equiv) was dissolved in ethanol or methanol (500 mL) with benzaldehyde derivative **22a**–**c** (1.5−5.0 equiv). Then, sodium hydroxide (5.00 equiv) was added to the solution, and the reaction mixture was stirred at room temperature for 3 h. Subsequently, the solvent was evaporated followed by three cycles of redissolution and evaporation. The residue was dissolved in another portion of the solvent (500 mL), and the solution was cooled to 0 °C in an ice bath. Hydrogen peroxide (30%, 3.00 equiv) was added dropwise, and the solution was stirred overnight. The reaction mixture was acidified with aqueous HCl to reach a pH between 6 and 7, and the precipitate was filtered and washed once with water and twice with cold ethanol or methanol. The product was purified by recrystallization.

#### 3-Hydroxy-2-phenyl-4*H*-chromen-4-one (**1**)

The compound was purchased from a commercial supplier and used without further purification. Mp: 168.5–171.2 °C. ^1^H NMR (500 MHz, DMSO-*d*_6_, Figure S1): δ (ppm) 9.58 (s, 1H), 8.23 (d, *J* = 7.3 Hz, 2H), 8.12 (dd, *J*_*1*_ = 8.0 Hz, *J*_*2*_ = 1.4 Hz, 1H), 7.83–7.75 (m, 2H), 7.59–7.56 (m, 2H), 7.52–7.46 (m, 2H). ^13^C{^1^H} NMR (125 MHz, DMSO-*d*_6_, Figure S2): δ (ppm) 173.0, 154.6, 145.2, 139.0, 133.7, 131.3, 129.9, 128.5, 127.6, 124.8, 124.5, 121.3, 118.4.

#### 2-(4-(Hydroxy)phenyl)-3-hydroxy-4*H*-chromen-4-one (**2**)

The compound was prepared according to the general procedure with 2-hydroxyacetophenone (0.467 mL, 3.876 mmol, 1.00 equiv), 4-methoxybenzaldehyde (2.36 mL, 19.38 mmol, 5.00 equiv), sodium hydroxide (0.775 g, 19.38 mmol, 5.00 equiv), and hydrogen peroxide (1.00 mL, 30%) in ethanol as a solvent. The title compound was recrystallized from ethanol. Yield: 0.62 g (60%). Yellow solid. Mp: 228.5–234.6 °C. ^1^H NMR (500 MHz, DMSO-*d*_6_, Figure S3): δ (ppm) 9.42 (s, 1H), 8.21 (d, *J* = 9.1 Hz, 2H), 8.11 (dd, *J* = 8.0, 1.3 Hz, 1H), 7.82–7.73 (m, 2H), 7.49–7.43 (m, 1H), 7.14 (d, J = 9.1 Hz, 2H), 3.86 (s, 3H). ^13^C{^1^H} NMR (125 MHz, DMSO-*d*_6_, Figure S4): δ (ppm) 172.6, 160.4, 154.4, 145.6, 138.1, 133.4, 129.4, 124.7, 124.4, 123.6, 121.3, 118.3, 114.0, 55.3. HRMS (APCI+): calcd. for C_16_H_13_O_4_^+^ [M + H^+^]: 269.0808, found: 269.0807. This compound has also been characterized elsewhere.^80^

#### 2-(4-(Dimethylamino)phenyl)-3-hydroxy-4*H*-chromen-4-one (**3**)

The compound was prepared according to the general procedure from 2-hydroxyacetophenone (0.467 mL, 3.876 mmol, 1.00 equiv), 4-dimethylaminobenzaldehyde (2.89 g, 19.38 mmol, 5.00 equiv), and sodium hydroxide (0.775 g, 19.38 mmol, 5.00 equiv) with hydrogen peroxide (1.00 mL, 30%) in ethanol as a solvent. The title compound was recrystallized from ethanol. Yield: 0.64 g (59%). Orange solid. Mp 184.5–188.7 °C. ^1^H NMR (500 MHz, DMSO-*d*_6_, Figure S5): δ (ppm) 3.02 (s, 6H), 6.85 (d, 2H, *J* = 9.1 Hz), 7.44 (t, 1H, *J* = 8.0 Hz), 7.74 (m, 2H), 8.09 (d, 1H, *J* = 8.0 Hz), 8.13 (d, 2H, *J* = 9.1 Hz), 9.12 (s, 1H). ^13^C{^1^H} NMR (125 MHz, DMSO-*d*_6_, Figure S6): δ (ppm) 111.4, 117.9, 118.1, 121.4, 124.2, 124.6, 128.9, 133.0, 137.2, 146.8, 151.0, 154.2, 171.9. A carbon signal of the 4′-dimethylamino group overlaps with that of DMSO-*d*_6_. (Note: the same applies for all the following compounds bearing the 4′-dimethylamino group measured in DMSO-*d*_6_). HRMS (APCI+, Figure S66) *m*/*z* [M + H^+^] calculated for C_17_H_16_NO_3_^+^: 282.1125, found: 282.1128. This compound has also been reported elsewhere.^81^

#### 6-Bromo-3-hydroxy-2-phenyl-4*H*-chromen-4-one (**4**)

The compound was prepared according to the general procedure from 5-bromo-2-hydroxyacetophenone (0.467 g, 3.876 mmol, 1.00 equiv), benzaldehyde (2.0 mL, 19.38 mmol, 5.00 equiv), sodium hydroxide (0.775 g, 19.38 mmol, 5.00 equiv), and hydrogen peroxide (1.30 mL, 30%) in ethanol as a solvent. The title compound was recrystallized from ethanol. Yield: 0.73 g (59%). Yellow solid (dec < 125 °C). ^1^H NMR (500 MHz, CDCl_3,_Figure S7): δ (ppm) 8.39 (d, 1H, *J* = 2.4 Hz), 8.26–8.20 (m, 2H), 7.79 (dd, 1H, *J*_*1*_ = 9.0 Hz, *J*_*2*_ = 2.4 Hz), 7.58–7.46 (m, 4H), 6.95 (s, 1H). ^13^C{^1^H} NMR (75 MHz, CDCl_3_, Figure S8): δ (ppm) 172.5, 154.3, 145.6, 138.8, 136.8, 130.9, 130.7, 128.8, 128.1, 128.0, 122.2, 120.4, 118.1. HRMS (APCI+, Figure S67): calcd. for C_15_H_10_BrO_3_^+^ [M + H^+^]: 316.9808, found: 316.9806. This compound has also been reported elsewhere.^81^

#### 6-Bromo-2-(4-(dimethylamino)phenyl)-3-hydroxy-4*H*-chromen-4-one (**5**)

The compound was prepared according to the general procedure from 5-bromo-2-hydroxyacetophenone (2.0 g, 9.30 mmol, 1.00 equiv), 4-dimethylaminobenzaldehyde (6.94 g, 46.50 mmol, 5.00 equiv), and sodium hydroxide (1.86 g, 46.50 mmol, 5.00 equiv), with hydrogen peroxide (2.80 mL, 30%) in ethanol as a solvent. The title compound was recrystallized from ethanol. Yield: 2.28 g (68%). Orange solid (dec < 225 °C). ^1^H NMR (500 MHz, DMSO-*d*_6_, Figure S9): δ (ppm) 9.32 (s, 1H), 8.14 (d, *J* = 2.5 Hz, 1H), 8.12 (d, *J* = 9.5 Hz, 2H), 7.89 (dd, *J*_1_ = 2.5 Hz, *J*_2_ = 9.0 Hz, 1H), 7.73 (d, *J* = 9.0 Hz, 1H), 6.84 (d, *J* = 9.5 Hz, 2H), 3.02 (s, 6H). ^13^C{^1^H} NMR (125 MHz, DMSO-*d*_6_, Figure S10): δ (ppm) 170.6, 153.1, 151.1, 147.4, 137.4, 135.5, 129.0, 126.5, 123.0, 120.8, 117.5, 116.5, 111.3. HRMS (APCI+, Figure S68): calculated for C_17_H_15_BrNO_3_^+^ [M + H^+^]: 360.0230, found: 360.0233. This compound has also been reported elsewhere.^81^

#### 7-Bromo-3-hydroxy-2-phenyl-4*H*-chromen-4-one (**24**)

The compound was prepared according to the general procedure from 2-hydroxy-4-bromoacetophenone (0.70 g, 3.25 mmol, 1.00 equiv), and benzaldehyde (1.66 mL, 16.28 mmol, 5.00 equiv) in methanol as a solvent. Yield: 0.17 g (30%). Yellow solid. Mp: 183.2–188.6 °C. ^1^H NMR (500 MHz, DMSO-*d*_6_, Figure S54): δ (ppm) 7.49–7.53 (m, 1H), 7.54–7.59 (m, 2H), 7.63 (dd, 1H, *J*_*1*_ = 8.5 Hz, *J*_*2*_ = 1.7 Hz), 8.03 (d, 1H, *J* = 8.5 Hz), 8.12 (d, 1H, *J* = 1.7 Hz), 8.20–8.24 (m, 2H), 9.73 (s, 1H). ^13^C{^1^H} NMR (125 MHz, DMSO-*d*_6_, Figure S55): δ (ppm) 120.5, 121.3, 126.7, 126.7, 127.7, 127.8, 128.5, 130.0, 131.0, 139.3, 145.3, 154.7, 172.5. HRMS (APCI+, Figure S90) *m*/*z* [M + H^+^] calculated for C_15_H_10_BrO_3_^+^: 316.9808, found: 316.9811. This compound has also been documented elsewhere.^82^

#### 7-Bromo-2-(4-(dimethylamino)phenyl)-3-hydroxy-4*H*-chromen-4-one (**25**)

Prepared according to the general procedure from 4-bromo-2-hydroxyacetophenone (5.00 g, 23.25 mmol, 1.00 equiv) and 4-(dimethylamino)benzaldehyde (6.95 g, 46.50 mmol, 2.00 equiv) in methanol as a solvent. Yield: 5.27 g (63%). Yellow solid. Mp: 207.6–212.0 °C. ^1^H NMR (500 MHz, DMSO-*d*_6_, Figure S58): δ (ppm) 9.29 (s, 1H), 8.13 (d, *J* = 9.0 Hz, 2H), 8.08 (d, *J* = 1.8 Hz, 1H), 7.99 (d, *J* = 8.5 Hz, 1H), 7.60 (dd, *J*_1_ = 1.8 Hz, *J*_2_ = 8.5 Hz, 1H), 6.84 (d, *J* = 9.0 Hz, 2H), 3.02 (s, 6H). ^13^C{^1^H} NMR (125 MHz, DMSO-*d*_6_, Figure S59): δ (ppm) 171.4, 154.4, 151.1, 147.0, 137.4, 129.0, 127.5, 126.5, 126.0, 121.0, 120.7, 117. 6, 111.3. HRMS (APCI+, Figure S92): calculated for C_17_H_15_BrNO_3_^+^ [M + H^+^]: 360.0230, found: 360.0233. This compound was also reported elsewhere.^83^

#### 6,7-Dibromo-2-(4-(dimethylamino)phenyl)-3-hydroxy-4*H*-chromen-4-one (**26**)

4-(Dimethylamino)benzaldehyde (8.8 g, 60.1 mmol, 1.50 equiv), 3′,4′-dibromo-2′-hydroxyacetophenone **21d** (11.8 g, 40.1 mmol, 1.00 equiv), and sodium hydroxide (9.62 g, 240.5 mmol, 6.00 equiv) were dissolved in methanol (330 mL). The solution was stirred under reflux for 30 min. The solvent was evaporated, and the solid residue was redissolved in methanol (330 mL), refluxed, and evaporated again to shift the equilibrium in favor of the aldol condensation. Finally, an ultrasonic bath redissolved the solid in methanol (330 mL), sodium hydroxide (9.62 g, 240.5 mmol, 6.00 equiv) was added, and the mixture was stirred for 3 h at 90 °C using a heat block. The reaction mixture was then cooled down with an ice bath, and hydrogen peroxide (30%, 180 mmol, 20.4 mL, 4.50 equiv) was added dropwise. A dark orange precipitate formed immediately, and after 16 h of mechanical stirring (200 rpm), an aqueous HCl solution (10% v/v) was added until a pH of 6–7 was reached. The precipitate was filtered and washed several times with water and then with *i-*PrOH (1 × 20 mL). Yield: 8.8 g (63%). Orange solid (dec < 201 °C). ^1^H NMR (500 MHz, DMSO-*d*_6_, Figure S62): δ (ppm) 9.45 (br, 1H), 8.32 (s, 1H), 8.27 (s, 1H), 8.15 (d, *J* = 9.3 Hz, 2H), 6.86 (d, *J* = 9.3 Hz, 2H), 3.03 (s, 6H). ^13^C{^1^H} NMR (125 MHz, DMSO-*d*_6_, Figure S63): δ (ppm) 170.2, 152.9, 151.2, 147.6, 137.5, 129.1, 128.5, 128.1, 123.7, 122.2, 119.2, 117.3, 111.3 HRMS (APCI–, Figure S94): calcd. for C_17_H_12_Br_2_NO_3_^–^ [M – H^+^]: 435.9189, found: 435.9186.

### General Procedure for Sonogashira Coupling of Compounds **5** and **25**

Compound **5** or **25** (2.50 g, 6.940 mmol, 1.00 equiv) was dissolved in dry DMF (100 mL). The solution was purged with nitrogen and kept under an inert atmosphere. Gradually, bis(triphenylphosphine)palladium(II) dichloride (195 mg, 0.28 mmol, 0.04 equiv), copper iodide (106 mg, 0.56 mmol, 0.08 equiv), and triethylamine (1.45 mL, 10.41 mmol, 1.50 equiv) were added. Afterward, trimethylsilylacetylen (1.23 mL, 10.41 mmol, 1.50 equiv) was added dropwise, and the reaction mixture was stirred at room temperature. After 24 h, another portion of all reagents except the starting material was added to the reaction mixture. The reaction was monitored with TLC (*n*-hexane/ethyl acetate, 3:1) until no starting material was detected. After that, DMF was evaporated followed by three cycles of redissolution in toluene and evaporation. The dry residue was redissolved in dichloromethane and adsorbed on silica. The crude product was purified using column chromatography (*n*-hexane/ethyl acetate; a gradient from 6:1 to 1:1).

#### 2-(4-(Dimethylamino)phenyl)-3-hydroxy-6-((trimethylsilyl)ethynyl)-4*H*-chromen-4-one (**6-TMS**)

The compound was prepared according to the general procedure for Sonogashira coupling from 6-bromo-2-(4-(dimethylamino)phenyl)-3-hydroxy-4H-chromen-4-one (2.50 g, 6.940 mmol, 1.00 equiv). Yield: 154 mg (10%). Orange solid. ^1^H NMR (500 MHz, DMSO-*d*_6_, Figure S15): δ (ppm) 9.33 (s, 1H), 8.13 (d, *J* = 9.2 Hz, 2H), 8.07 (d, *J* = 2.0 Hz, 1H), 7.77 (dd, *J*_*1*_ = 2.0 Hz, *J*_*2*_ = 8.7 Hz, 1H), 7.73 (d, *J* = 8.7 Hz, 1H), 6.85 (d, *J* = 9.2 Hz, 2H), 3.02 (s, 6H), 0.26 (s, 9H). ^13^C NMR (125 MHz, DMSO-*d*_6_, Figure S16): δ (ppm) 171.0, 153.9, 151.1, 147.2, 137.4, 135.4, 129.0, 128.0, 121.5, 119.0, 118.1, 117.6, 111.4, 103.8, 94.9, −0.2. HRMS (APCI+, Figure S71): calculated for C_22_H_24_NO_3_Si^+^ [M + H^+^]: 378.1520, found: 378.1522.

#### 2-(4-(Dimethylamino)phenyl)-3-hydroxy-7-((trimethylsilyl)ethynyl)-4*H*-chromen-4-one (**7-TMS**)

Prepared according to the general procedure for Sonogashira coupling from 7-bromo-2-(4-(dimethylamino)phenyl)-3-hydroxy-4*H*-chromen-4-one (1.50 g, 4.164 mmol, 1.00 equiv). Yield: 1.98 g (76%). Red solid (dec < 179 °C). ^1^H NMR (500 MHz, DMSO-*d*_6_, Figure S17): δ (ppm) 9.28 (s, 1H), 8.14 (d, *J* = 9.0 Hz, 2H), 8.03 (d, *J* = 8.0 Hz, 1H), 7.86 (s, 1H), 7.44 (d, *J* = 8.0 Hz, 1H), 6.84 (d, *J* = 9.0 Hz, 2H), 3.02 (s, 6H), 0.27 (s, 9H). ^13^C{^1^H} NMR (125 MHz, DMSO-*d*_6_, Figure S18): δ (ppm) 171.2, 153.8, 151.1, 147.2, 137.6, 129.0, 127.1, 126.1, 125.0, 121.4, 121.2, 117.6, 111.3, 103.6, 98.1, −0.3. HRMS (APCI+, Figure S73): calculated for C_22_H_24_NO_3_Si^+^ [M + H^+^]: 378.1520, found: 378.1521.

### General Procedure for TMS Deprotection

A TMS-protected derivative, **6-TMS** or **7-TMS** (0.350 g, 0.927 mmol, 1.00 equiv), was dissolved in a mixture of THF and methanol (1:1–2:1, 40 mL). Potassium carbonate (192 mg, 1.39 mmol, 1.50 equiv) was added to the solution followed by a small amount of deionized water (100 μL). The reaction mixture was stirred at room temperature for 5 h until no starting material was visible on TLC (ethyl acetate/*n*-hexane 1:3). Then, 0.1 M HCl was added (70 mL), and the precipitate was filtered and washed three times with water (50 mL) and twice with methanol (50 mL).

#### 2-(4-(Dimethylamino)phenyl)-6-ethynyl-3-hydroxy-4*H*-chromen-4-one (**6**)

The compound was prepared according to the general procedure for TMS deprotection from 2-(4-(dimethylamino)phenyl)-3-hydroxy-6-((trimethylsilyl)ethynyl)-4*H*-chromen-4-one (110 mg, 0.2914 mmol, 1.00 equiv). Yield: 75 mg (84%). Yellow solid (dec < 233 °C). ^1^H NMR (500 MHz, DMSO-*d*_6_, Figure S13): δ (ppm) 9.31 (s, 1H), 8.13 (d, *J* = 9.0 Hz, 2H), 8.09 (d, *J* = 1.7 Hz, 1H), 7.80 (dd, *J*_1_ = 1.7 Hz, *J*_2_ = 8.7 Hz, 1H), 7.75 (d, *J* = 8.7 Hz, 1H), 6.85 (d, *J* = 9.0 Hz, 2H), 4.29 (s, 1H), 3.02 (s, 6H). ^13^C{^1^H} NMR (125 MHz, DMSO-*d*_6_, Figure S14): δ (ppm) 171.0, 153.9, 151.1, 147.2, 137.4, 135.6. 129.0, 127.9, 121.5, 119.0, 117.7, 117.6, 111.4, 82.2, 81.3. HRMS (APCI+, Figure S70): calculated for C_19_H_16_NO_3_^+^ [M + H^+^]: 306.1125, found: 306.1126.

#### 2-(4-(Dimethylamino)phenyl)-7-ethynyl-3-hydroxy-4*H*-chromen-4-one (**7**)

The compound was prepared according to the general procedure for TMS deprotection from 2-(4-(dimethylamino)phenyl)-3-hydroxy-7-((trimethylsilyl)ethynyl)-4*H*-chromen-4-one (1.50 g, 3.97 mmol, 1.00 equiv). Yield: 0.444 g (37%). Dark orange solid. Mp: 176.8–183.2 °C. ^1^H NMR (500 MHz, DMSO-*d*_6_, Figure S19): δ (ppm) 9.27 (s, 1H), 8.13 (d, *J* = 9.2 Hz, 2H), 8.05 (d, *J* = 8.2 Hz, 1H), 7.87 (d, *J* = 1.0 Hz, 1H), 7.47 (dd, *J*_1_ = 1.0 Hz, *J*_2_ = 8.2 Hz, 1H), 6.84 (d, *J* = 9.2 Hz, 2H), 4.53 (s, 1H), 3.02 (s, 6H). ^13^C{^1^H} NMR (125 MHz, DMSO-*d*_6_, Figure S20): δ (ppm) 171.2, 153.8, 151.1, 147.2, 137.6, 129.0, 127.2, 125.8, 125.1, 121.5, 121.3, 117.6, 111.3, 84.1, 82.2. HRMS (APCI+, Figure S72): calculated for C_19_H_16_NO_3_^+^ [M + H^+^]: 306.1125, found: 306.1128.

#### 4-(7-Ethynyl-3-hydroxy-4-oxo-4*H*-chromen-2-yl)-*N*,*N*,*N*-trimethylbenzenammonium Trifluoromethanesulfonate (**8**)

Flavonol **7** (500 mg, 1.64 mmol, 1.00 equiv) was dissolved in dry dichloromethane (40 mL), and the solution was purged with nitrogen. Methyl trifluoromethanesulfonate (371 μL, 3.28 mmol, 2.00 equiv) was added dropwise, and the reaction mixture was stirred overnight at room temperature. The precipitate was filtered and washed with dichloromethane (3 × 5 mL). The crude product was purified with reversed-phase chromatography (C18; methanol/water with 0.1% TfOH; gradient from 3:7 to pure methanol). Collected fractions were stirred overnight with polypyridine ion-exchange resin (230 mg) at room temperature. Afterward, the solution was filtered, and the solvent evaporated under reduced pressure. Yield: 0.38 g (50%). Pale solid. Mp: 274.4–278.3 °C. ^1^H NMR (500 MHz, DMSO-*d*_6_, Figure S21): δ (ppm) 10.14 (s, 1H), 8.42 (d, *J* = 9.0 Hz, 2H), 8.16 (d, *J* = 9.0 Hz, 2H), 8.11 (d, *J* = 8.2 Hz, 1H), 7.98 (s, 1H), 7.54 (d, *J* = 8.2 Hz, 1H), 4.61 (s, 1H), 3.67 (s, 9H). ^13^C{^1^H} NMR (125 MHz, DMSO-*d*_6_, Figure S22): δ (ppm) 172.7, 154.2, 147.5, 140.3, 132.7, 129.0, 127.6, 126.8, 125.4, 124.5 (TfO^–^), 121.9 (TfO^–^), 121.8, 121.3, 120.8, 119.4 (TfO^–^), 116.8 (TfO^–^), 84.8, 82.0, 56.4. ^19^F-NMR (470 MHz, DMSO-*d*_6_, Figure S23): δ (ppm) – 77.7 (s). HRMS (ESI+, Figure S74): calculated for C_20_H_18_NO_3_^+^ [M^+^]: 320.1281, found: 320.1284.

### General Procedure for MOM Protection

Compound **24** or **25** (1.34 g, 1.00 equiv) was suspended in dichloromethane (80 mL), and *N*,*N*-diisopropylethylamine (1.8 equiv) was added. The reaction mixture was cooled on an ice bath to 0 °C, and bromo(methoxy)methane (1.5 equiv) was added dropwise over 30 min. The reaction mixture was stirred overnight and then washed with a sat. Na_2_CO_3_ solution (2 × 30 mL), water (1 × 30 mL), and brine (1 × 30 mL). The organic phase was dried with anhydrous MgSO_4_ and filtered, and the solvent was removed under reduced pressure.

#### 7-Bromo-3-(methoxymethoxy)-2-phenyl-4*H*-chromen-4-one (**24-MOM**)

The compound was prepared according to the general procedure for MOM protection from 7-bromo-3-hydroxy-2-phenyl-4*H*-chromen-4-one (0.15 g, 0.47 mmol, 1.00 equiv). Yield: 0.12 g (67%). Light-green solid. Mp: 114.4–117.6 °C. ^1^H NMR (500 MHz, DMSO-*d*_6_, Figure S56): δ (ppm) 3.05 (s, 3H), 5.16 (s, 2H), 7.58–7.63 (m, 3H), 7.69 (dd, 1H, *J*_*1*_ = 8.5 Hz, *J*_*2*_ = 1.8 Hz), 8.02 (d, 1H, *J* = 8.5 Hz), 8.05–8.09 (m, 2H), 8.12 (d, 1H, *J* = 1.8 Hz). ^13^C{^1^H} NMR (125 MHz, DMSO-*d*_6_, Figure S57): δ (ppm) 57.3, 97.4, 121.9, 123.0, 127.3, 127.7, 129.0, 129.0, 129.3, 130.7, 131.5, 137.9, 155.5, 156.5, 173.8. HRMS (APCI+, Figure S91) *m*/*z* [M + H^+^] calculated for C_17_H_14_BrO_4_^+^: 361.0070, found: 361.0072.

#### 7-Bromo-2-(4-(dimethylamino)phenyl)-3-(methoxymethoxy)-4*H*-chromen-4-one (**25-MOM**)

The compound was prepared according to the general procedure for MOM protection from 7-bromo-2-(4-(dimethylamino)phenyl)-3-hydroxy-4*H*-chromen-4-one (1.34 g, 3.72 mmol, 1.00 equiv). Yield: 1.41 g (94%). Dark orange solid. Mp: 139.4–143.2 °C. ^1^H NMR (500 MHz, DMSO-*d*_6_, Figure S60): δ (ppm) 8.07 (d, *J* = 1.8 Hz, 1H), 8.05 (d, *J* = 9.2 Hz, 2H), 7.97 (d, *J* = 8.5 Hz, 1H), 7.63 (dd, J_1_ = 1.8 Hz, J_2_ = 8.5 Hz, 1H), 6.85 (d, J = 9.2 Hz, 2H), 5.18 (s, 2H), 3.20 (s, 3H), 3.04 (s, 6H). ^13^C{^1^H} NMR (125 MHz, DMSO-*d*_6_, Figure S61): δ (ppm) 57.1, 96.6, 111.1, 116.2, 121.0, 122.5, 126.5, 126.7, 128.0, 130.0, 135.8, 151.7, 154.6, 156.5, 172.4. HRMS (APCI+, Figure S93) *m*/*z* [M + H^+^] calculated for C_19_H_19_BrNO_4_^+^: 404.0492, found: 404.0496.

#### 3-Hydroxy-4-oxo-2-phenyl-4*H*-chromene-7-carbonitrile (**9**)

A 50 mL two-necked round-bottom flask was charged with **24-MOM** (0.469 g, 1.298 mmol, 1.00 equiv) in dry dimethylacetamide (20 mL), and polymethylhydrosiloxane (100 μL) was added at room temperature. The solution was purged with N_2_ for 30 min. Then, the reaction mixture was heated up to 110 °C (heat block), and Pd_2_(dba)_3_ (12 mg, 0.013 mmol, 0.010 equiv) and DPPF (10 mg, 0.018 mmol, 0.014 equiv) were added. Afterward, Zn(CN)_2_ (0.27 g, 2.337 mmol, 1.80 equiv) was added in four portions. The reaction was monitored with TLC (dichloromethane/ethyl acetate, 39:1). The reaction mixture was cooled down, diluted with dichloromethane (70 mL), and filtered. The filtrate was washed with water (8 × 100 mL), dried with anhydrous MgSO_4_, filtered, and concentrated under reduced pressure. The crude product was purified by column chromatography (dichloromethane/ethyl acetate, a gradient from 39:1 to 19:1) to afford the MOM-deprotected product. Yield: 0.306 g (78%). Dark orange solid (dec < 307 °C). ^1^H NMR (500 MHz, 2.5% *d*_6_-DMSO/CD_2_Cl_2_ v/v, Figure S24): δ (ppm) 7.34–7.69 (m, 4H), 8.16 (s, 1H), 8.39 (brs, 1H), 8.71 (s, 2H). ^13^C{^1^H} NMR (125 MHz, 2.5% *d*_6_-DMSO/CD_2_Cl_2_ v/v, Figure S25): δ (ppm) 114.1, 117.3, 121.2, 123.0, 125.1, 126.0, 126.7, 127.8, 128.6, 132.5, 146.7, 149.5, 152.2, 177.1. HRMS (APCI+, Figure S75) *m*/*z* [M + H^+^] calculated for C_16_H_10_NO_3_^+^: 264.0655, found: 264.0652.

#### 2-(4-(Dimethylamino)phenyl)-3-hydroxy-4-oxo-4*H*-chromene-7-carbonitrile (**11**)

A 50 mL two-necked round-bottom flask was charged with **25-MOM** (0.500 g, 1.237 mmol, 1.00 equiv) in dry dimethylacetamide (20 mL), and polymethylhydrosiloxane (100 μL) was added at room temperature. The solution was purged with N_2_ for 30 min. The reaction mixture was heated up to 110 °C (heat block), and Pd_2_(dba)_3_ (11 mg, 0.012 mmol, 0.010 equiv) and DPPF (10 mg, 0.017 mmol, 0.014 equiv) were added. Afterward, Zn(CN)_2_ (0.218 g, 1.855 mmol, 1.50 equiv) was added to the heterogeneous mixture in four portions. After TLC (dichloromethane/methanol, 96.8:3.2) indicated the completion of the reaction, the reaction mixture was cooled down to room temperature, diluted with 70 mL of dichloromethane, and filtered. The filtrate was washed with water (8 × 100 mL), dried with anhydrous MgSO_4_, filtered, and concentrated under reduced pressure. The obtained residue was purified by flash column chromatography (dichloromethane/ethyl acetate, a gradient from 99.5:0.5 to 19:1) to afford a partially MOM-protected product (yield: 0.165 g, 38%). Combined products were dissolved in dichloromethane (10 mL), and trifluoroacetic acid (0.180 mL, 2.355 mmol, 5.00 equiv) was added to an ice-bath-cooled solution. The reaction mixture was stirred overnight. After TLC (dichloromethane/ethyl acetate, 10:1) indicated the end of the reaction, Amberlyst A21 (Alfa Aesar) as an ion-exchange resin was added (0.5 g). The mixture was filtered, and the solvents were evaporated under reduced pressure. The resulting residue was precipitated from dichloromethane with methanol. Yield: 0.12 g (32%). Dark-red solid (dec < 288.6 °C). ^1^H NMR (500 MHz, DMSO-*d*_6_, Figure S30): δ (ppm) 3.03 (s, 6H), 6.86 (d, 2H, *J* = 8.5 Hz), 7.80 (d, 1H, *J* = 8.2 Hz), 8.15 (d, *J* = 8.5 Hz, 2H), 8.20 (d, *J* = 8.2 Hz, 1H), 8.39 (s, 1H), 9.51 (s, 1H). ^13^C{^1^H} NMR (125 MHz, DMSO-*d*_6_, Figure S31): δ (ppm) 111.4, 114.4, 117.2, 117.7, 123.4, 124.4, 126.0, 126.6, 129.2, 138.1, 148.0, 151.3, 153.3, 170.8. HRMS (APCI+, Figure S78) *m*/*z* [M + H^+^] calculated for C_18_H_15_N_2_O_3_^+^: 307.1077, found: 307.1080.

### General Procedure for TIPS Protection

Compounds **5** or **26** (0.50 g, 1.00 equiv) and imidazole (3.00 equiv) were dissolved in dry dichloromethane (80 mL) and stirred for 25 min. Afterward, TIPSOTf (3.00 equiv) was added dropwise, and the reaction mixture was stirred at room temperature. After 2 h (completion of the reaction was followed with TLC – *n*-hexane/ethyl acetate, 3:1), the reaction mixture was diluted with dichloromethane (70 mL) and washed with water (2 × 75 mL). The organic phase was dried with anhydrous MgSO_4_ and filtered, and the solvent was evaporated under reduced pressure. The obtained residue was suspended in *n*-hexane (5 mL), sonicated, filtered, and washed twice with *n*-hexane.

#### 6-Bromo-2-(4-(dimethylamino)phenyl)-3-(triisopropylsilyl)oxy-4*H*-chromen-4-one (**5-TIPS**)

The compound was prepared according to the general procedure for TIPS protection from 6-bromo-2-(4-(dimethylamino)phenyl)-3-hydroxy-4*H*-chromen-4-one (0.50 g, 1.388 mmol, 1.00 equiv). Yield: 0.41 g (57%). Ocher solid. ^1^H NMR (500 MHz, DMSO-*d*_6_, Figure S11): δ (ppm) 8.15 (d, *J* = 2.5 Hz, 1H), 8.02 (d, *J* = 9.2 Hz, 2H), 7.91 (dd, *J*_1_ = 2.5 Hz, *J*_2_ = 8.9 Hz, 1H), 7.71 (d, *J* = 8.9 Hz, 1H), 6.82 (d, *J* = 9.2 Hz, 2H), 3.03 (s, 6H), 1.36 (m, *J* = 7.5 Hz, 3H), 1.03 (d, *J* = 7.5 Hz, 18H). ^13^C{^1^H} NMR (125 MHz, DMSO-*d*_6_, Figure S12): δ (ppm) 171.4, 153.0, 151.9, 151.5, 136.6, 135.8, 129.6, 126.8, 123.7, 120.8, 117.0, 116.8, 111.0, 18.00, 14.0. HRMS (APCI+, Figure S69): calculated for C_26_H_35_BrNO_3_Si^+^ [M + H^+^]: 516.1564, found: 516.1568.

#### 6,7-Dibromo-2-(4-(dimethylamino)phenyl)-3-(triisopropylsilyl)oxy-4*H*-chromen-4-one (**26-TIPS**)

The compound was prepared according to the general procedure for TIPS protection from 6-bromo-2-(4-(dimethylamino)phenyl)-3-hydroxy-4*H*-chromen-4-one (1.00 g, 2.277 mmol, 1.00 equiv). Yield: 1.07 g (50%). Ocher solid. Mp: 194.8–198.9 °C. ^1^H NMR (500 MHz, CDCl_3_, Figure S64): δ (ppm) 8.46 (s, 1H) 8.06 (d, *J* = 9.0 Hz, 2H), 7.85 (s, 1H), 6.78 (d, *J* = 9.0 Hz, 2H), 3.10 (s, 6H), 1.43 (m, *J* = 7.5 Hz, 3H), 1.10 (d, *J* = 7.5 Hz, 18H). ^13^C{^1^H} NMR (125 MHz, CDCl_3_, Figure S65): δ (ppm) 172.0, 153.2, 152.6, 151.4, 137.8, 130.0, 130.0, 128.8, 123.4, 122.9, 120.1, 111.2, 40.2, 18.2, 14.7, 14.4. HRMS (APCI+, Figure S95): calculated for C_26_H_34_Br_2_NO_3_Si^+^ [M + H^+^]: 594.0675, found: 594.0677.

#### 2-(4-(Dimethylamino)phenyl)-3-(triisopropylsilyl)oxy-4-oxo-4*H*-chromene-7-carbonitrile (**10-TIPS**)

Compound **5-TIPS** (0.40 g, 0.774 mmol, 1.00 equiv) was dissolved in dry dimethylacetamide (20 mL), and polymethylhydrosiloxane (100 μL) was added at room temperature. The reaction mixture was heated up to 80 °C to afford complete dissolution of the starting material and purged with N_2_ for 20 min. Then, Pd_2_(dba)_3_ (7.1 mg, 0.0077 mmol, 0.010 equiv) and DPPF (5.8 mg, 0.0104 mmol, 0.014 equiv) were added, and the reaction mixture was heated up to 110 °C (heat block). Afterward, Zn(CN)_2_ (0.164 g, 1.394 mmol, 1.80 equiv) was added in four portions. The reaction mixture was stirred overnight at 110 °C; the completion was indicated by TLC (dichloromethane). Afterward, the reaction mixture was cooled down, diluted with ethyl acetate (40 mL), and washed with brine (4 × 40 mL). The organic phase was dried with anhydrous MgSO_4_, filtered, and evaporated under reduced pressure. The resulting residue was purified with column chromatography (dichloromethane/*n*-hexane, a gradient from 3:7 to 4:1). Yield: 0.25 g (70%). Ocher solid. Mp: 202.3–204.1 °C. ^1^H NMR (500 MHz, DMSO-*d*_6_, Figure S28): δ (ppm) 8.49 (d, *J* = 2.0 Hz, 1H), 8.15 (dd, *J*_1_ = 2.0 Hz, *J*_2_ = 8.7 Hz, 1H), 8.04 (d, *J* = 9.2 Hz, 2H), 7.92 (d, *J* = 8.7 Hz, 1H), 6.83 (d, *J* = 9.2 Hz, 2H), 3.04 (s, 6H), 1.37 (m, *J* = 7.5 Hz, 3H), 1.04 (d, *J* = 7.5 Hz, 18H). ^13^C{^1^H} NMR (125 MHz, DMSO-*d*_6_, Figure S29): δ (ppm) 171.3, 156.0, 152.2, 151.6, 136.9, 135.6, 130.6, 129.7, 122.5, 120.0, 117.9, 116.6, 111.0, 107.5, 18.0, 14.0. HRMS (APCI+, Figure S77): C_27_H_35_N_2_O_3_Si^+^ [M + H^+^]: 463.2411, found: 463.2413.

#### 2-(4-(Dimethylamino)phenyl)-3-(triisopropylsilyl)oxy-4-oxo-4*H*-chromene-6,7-dicarbonitrile (**12-TIPS**)

Compound **26-TIPS** (0.91 g, 1.534 mmol, 1.00 equiv) was dissolved in dry dimethylacetamide (45 mL), and polymethylhydrosiloxane (250 μL) was added at room temperature. The reaction mixture was heated up to 80 °C to dissolve the starting material and purged with N_2_ for 20 min. Then, Pd_2_(dba)_3_ (28 mg, 0.0307 mmol, 0.020 equiv) and DPPF (23 mg, 0.0414 mmol, 0.027 equiv) were added, and the reaction mixture was heated up to 110 °C (heat block). Afterward, Zn(CN)_2_ (0.648 g, 5.522 mmol, 3.60 equiv) was added in four portions. The reaction mixture was stirred overnight at 110 °C; the completion was indicated by TLC (dichloromethane). Afterward, the reaction mixture was cooled down, diluted with ethyl acetate (90 mL), and washed with brine (4 × 90 mL). The organic phase was dried with anhydrous Na_2_SO_4_, filtered, and evaporated under reduced pressure. The resulting residue was purified with column chromatography (dichloromethane/*n*-hexane, a gradient from 3:7 to 4:1). Yield: 0.48 g (64%). Orange solid. Mp: 202.7–208.3 °C. ^1^H NMR (500 MHz, CDCl_3_, Figure S34): δ (ppm) 8.69 (s, 1H) 8.09 (d, *J* = 9.0 Hz, 2H), 7.97 (s, 1H), 6.81 (d, *J* = 9.0 Hz, 2H), 3.13 (s, 6H), 1.44 (m, *J* = 7.5 Hz, 3H), 1.11 (d, *J* = 7.5 Hz, 18H). ^13^C{^1^H} NMR (125 MHz, CD_2_Cl_2_, Figure S35): δ (ppm) 170.9, 155.7, 154.2, 152.5, 139.2, 133.4, 130.5, 126.3, 125.0, 117.9, 117.2, 115.5, 115.2, 111.5, 110.5, 40.3, 18.3, 15.0. HRMS (APCI+, Figure S80): C_28_H_34_N_3_O_3_Si^+^ [M + H^+^]: 488.2369, found: 488.2363.

#### 2-(4-(Dimethylamino)phenyl)-3-hydroxy-4-oxo-4*H*-chromene-7-carbonitrile (**10**)

TIPS-protected compound **10-TIPS** (0.10 g, 0.2161 mmol, 1.00 equiv) was dissolved in THF (10 mL), and tetrabutylammonium chloride (1 M in THF, 0.65 mL, 0.6484 mmol, 3.00 equiv) was added dropwise at room temperature. TLC (dichloromethane) showed completion after 30 min, and the reaction was quenched with 20 mL of a saturated NH_4_Cl solution. The organic phase was washed with water (15 mL) and brine (15 mL), dried with anhydrous MgSO_4_, filtered, and evaporated under reduced pressure. The resulting residue was suspended in dichloromethane (2 mL), sonicated, diluted with *n*-hexane (10 mL), filtered, and washed twice with *n*-hexane. Yield: 19 mg (29%). Brown solid (dec < 274 °C). ^1^H NMR (500 MHz, DMSO-*d*_6_, Figure S26): δ (ppm) 9.52 (s, 1H), 8.47 (d, *J* = 2.0 Hz, 1H), 8.13 (m, 3H), 7.93 (d, *J* = 8.7 Hz, 1H), 6.85 (d, *J* = 8.9 Hz, 2H), 3.03 (s, 6H). ^13^C{^1^H} NMR (125 MHz, DMSO-*d*_6_, Figure S27): δ (ppm) 171.1, 156.6, 151.7, 148.2, 138.2, 135.7, 130.9, 129.6, 122.4, 120.6, 118.5, 117.7, 111.9, 107.6. HRMS (APCI+, Figure S76): calculated for C_18_H_15_N_2_O_3_^+^ [M + H^+^]: 307.1077, found: 307.1075.

#### 2-(4-(Dimethylamino)phenyl)-3-hydroxy-4-oxo-4*H*-chromene-6,7-dicarbonitrile (**12**)

TIPS-protected compound **12-TIPS** (0.47 g, 0.9646 mmol, 1.00 equiv) was dissolved in THF (45 mL), and tetrabutylammonium chloride (1 M in THF, 2.89 mL, 2.8938 mmol, 3.00 equiv) was added dropwise at room temperature. TLC (dichloromethane) showed completion after 50 min, and the reaction was quenched with 70 mL of a saturated NH_4_Cl solution while a precipitate formed. The precipitate was filtered and washed three times with water and twice with *n*-hexane. Yield: 230 mg (72%). Red solid (dec < 259 °C). ^1^H NMR (500 MHz, DMSO-*d*_6_, Figure S32): δ (ppm) 9.89 (s, 1H), 8.71 (s, 1H), 8.70 (s, 1H), 8.15 (d, *J* = 9.0 Hz, 2H), 6.87 (d, *J* = 9.0 Hz, 2H), 3.05 (s, 6H). ^13^C{^1^H} NMR (125 MHz, DMSO-*d*_6_, Figure S33): δ (ppm) 169.5, 155.0, 151.4, 148.5, 138.7, 132.1, 129.3, 125.9, 124.4, 116.6, 116.4, 115.4, 115.2, 111.4, 109.0. HRMS (APCI–, Figure S79): calculated for C_19_H_12_N_3_O_3_^–^ [M – H^+^]: 330.0884, found: 330.0883.

#### 3-Methoxyflavone (**19**)

3-Methoxyflavone (**19**) was synthesized from 3-hydroxyflavone (**1**) (2.0 g, 8.395 mmol, 1.00 equiv) and sodium hydroxide (0.369 g, 9.234 mmol, 1.10 equiv). Reagents were dissolved in acetonitrile (75 mL), and methyl iodide (5.23 mL, 83.95 mmol, 10.00 equiv) was added. The mixture was stirred for 48 h. The solvent was evaporated under vacuum, and the remaining solid was recrystallized in water to give the title compound. Yield: 1.354 g (64%). Pale yellow crystals. Mp: 113.2–116.8 °C. ^1^H NMR (500 MHz, DMSO-*d*_6_, Figure S48): δ (ppm) 8.10 (d, 1H, *J* = 9.2 Hz), 8.08–8.03 (m, 2H), 7.83 (m, 1H), 7.76 (d, 1H, *J* = 8.4 Hz), 7.63–7.57 (m, 3H), 7.50 (m, 1H), 3.83 (s, 3H). ^13^C{^1^H} NMR (125 MHz, DMSO-*d*_6_, Figure S49): δ (ppm) 173.9, 155.0, 154.7, 140.8, 134.1, 130.5, 128.7, 128.3, 125.0, 124.9, 123.5, 118.4, 59.7. HRMS (APCI+, Figure S87): calcd. for C_16_H_13_O_3_^+^ [M + H^+^]: 253.0859, found: 253.0858. This compound was also reported elsewhere.^84^

### General Method of the Synthesis of 3-Hydroxyflavothione Derivatives (**13–18** and **20**)

A 3-hydroxyflavone derivative (1 equiv) was dissolved in dry toluene, Lawesson’s reagent (1.2 equiv) was added, and the reaction was refluxed (oil bath or heat block) for 24 h under a nitrogen atmosphere in the dark. The reaction mixture was filtered on a frit glass filter to remove insoluble impurities, and the filtrate was evaporated under reduced pressure. The crude material was then purified on a silica column and recrystallized to yield the corresponding 3-hydroxyflavothione derivative.

#### 3-Hydroxyflavothione (**13**)

Synthesis of **13** was performed according to the general method of the synthesis of 3-hydroxyflavothione derivatives with 3-hydroxyflavone **1** (0.500 g, 2.01 mmol, 1.00 equiv) and Lawesson’s reagent (0.971 g, 2.4 mmol, 1.20 equiv). The crude product was purified by column chromatography (*n*-hexane/dichloromethane, 2:1) and recrystallization from ethanol. Yield: 0.50 (93%). Bright red crystals (dec < 76 °C). ^1^H NMR (500 MHz, DMSO-*d*_6_, Figure S36): δ (ppm) 8.98 (s, 1H), 8.46 (dd, 1H, *J*_*1*_ = 8.2 Hz, *J*_*2*_ = 1.4 Hz), 8.36 (m, 2H), 7.93 (m, 2H), 7.63 (m, 4H). ^13^C{^1^H} NMR (125 MHz, DMSO-*d*_6_, Figure S37): δ (ppm) 187.8, 149.9, 146.1, 141.6, 133.9, 131.2, 130.5, 128.9, 128.7, 127.7, 127.6, 126.4, 119.0. HRMS (APCI+, Figure S81): calcd. for C_15_H_11_O_2_S^+^ [M + H^+^]: 255.0474, found: 255.0476. This compound was also reported elsewhere.^85^

#### 3-Hydroxy-4′-methoxyflavothione (**14**)

The synthesis of **14** was performed according to the general method of the synthesis of 3-hydroxyflavothione derivatives with 3-hydroxy-4′-methoxyflavone (**2**; 0.62 g, 2.31 mmol, 1.00 equiv) and Lawesson’s reagent (1.12 g, 2.77 mmol, 1.20 equiv). The crude product was purified by column chromatography (*n*-hexane/dichloromethane, 2:1) and recrystallization from ethanol. Yield: 0.44g (67%). Orange solid (dec < 125 °C). ^1^H NMR (500 MHz, DMSO-*d*_6_, Figure S38): δ (ppm) 8.93 (s, 1H), 8.45 (d, 1H, *J* = 8.2 Hz), 8.39 (d, 2H, *J* = 9.1 Hz), 7.90 (m, 2H), 7.60 (m, 1H), 7.21 (d, 2H, *J* = 9.1 Hz), 3.89 (s, 3H). ^13^C{^1^H} NMR (125 MHz, DMSO-*d*_6_, Figure S39): δ (ppm) 186.0, 161.7,149.7, 145.6, 142.2, 133.5, 130.8, 127.7, 127.3, 126.2, 122.5, 118.9, 114.6, 55.6. HRMS (APCI+, Figure S82): calcd. for C_16_H_13_O_3_S^+^ [M + H^+^]: 285.0580, found: 285.0580. This compound was also reported elsewhere.^86^

#### 4′-Dimethylamino-3-hydroxyflavothione (**15**)

The synthesis of **15** was performed according to the general method of the synthesis of 3-hydroxyflavothione derivatives with 4′-dimethylamino-3-hydroxyflavone (**3**; 0.39 g, 1.39 mmol, 1.00 equiv) and Lawesson’s reagent (0.673 g, 1.66 mmol, 1.20 equiv). The crude product was purified by column chromatography (*n*-hexane/dichloromethane, 2:1) and recrystallization from ethanol. Yield: 0.23g (55%). Purple solid (dec < 125 °C). ^1^H NMR (500 MHz, DMSO-*d*_6_, Figure S40): δ (ppm) 8.88 (s, 1H), 8.40 (d, 1H, *J* = 9.5 Hz), 8.34 (d, 2H, *J* = 9.3 Hz), 7.88 (m, 1H), 7.81 (m, 1H), 7.56 (m, 1H), 6.92 (d, 2H, *J* = 9.4 Hz), 3.09 (s, 6H).^13^C{^1^H} NMR (125 MHz, DMSO-*d*_6_, Figure S41): δ (ppm) 181.7, 152.2, 149.5, 145.0, 144.0, 132.7, 130.6, 127.6, 126.8, 125.9, 118.4, 115.9, 111.7. HRMS (APCI+, Figure S83): calcd. for C_17_H_16_NO_2_S^+^ [M + H^+^]: 298.0896, found: 298.0899.

#### 6-Bromo-3-hydroxyflavothione (**16**)

The synthesis of **16** was performed according to the general method of the synthesis of 3-hydroxyflavothione derivatives with 6-bromo-3-hydroxyflavone (**4**; 0.500 g, 1.58 mmol, 1.00 equiv) and Lawesson’s reagent (0.765 g, 1.89 mmol, 1.20 equiv). The crude was purified by column chromatography (*n*-hexane/dichloromethane, 2:1) and recrystallization from ethanol. Yield: 0.37g (70%). Brown solid (dec < 136 °C). ^1^H NMR (500 MHz, DMSO-*d*_6_, Figure S42): δ (ppm) 9.07 (s, 1H), 8.53 (d, 1H, *J* = 2.4 Hz), 8.35 (m, 2H), 8.04 (dd, 1H, *J*_*1*_ = 9.0 Hz, *J*_*2*_ = 2.4 Hz), 7.94 (d, 1H, *J* = 9.0 Hz), 7.63 (m, 3H). ^13^C{^1^H} NMR (125 MHz, DMSO-*d*_6_, Figure S43): δ (ppm) 186.3, 148.9, 146.8, 142.0, 136.1, 131.3, 130.3, 129.4, 129.2, 128.9, 128.8, 121.8, 119.0. HRMS (APCI+, Figure S84): calcd. for C_15_H_10_BrO_2_S^+^ [M + H^+^]: 332.9579, found: 332.9582.

#### 6-Bromo-4′-dimethylamino-3-hydroxyflavothione (**17**)

The synthesis of 6-bromo-4′-dimethylamino-3-hydroxyflavothione (**17**) was performed according to the general method of the synthesis of 3-hydroxyflavothione derivatives with 6-bromo-2-(4-(dimethylamino)phenyl)-3-hydroxy-4*H*-chromen-4-one (**5**; 2.18 g, 6.04 mmol, 1.00 equiv) and Lawesson’s reagent (2.93 g, 7.25 mmol, 1.20 equiv). The crude product was purified by column chromatography (*n*-hexane/dichloromethane, 2:1) and then recrystallized from ethanol, chloroform, and ethanol. Yield: 0.95g (42%). Violet solid (dec < 245 °C). ^1^H NMR (500 MHz, CD_2_Cl_2_, Figure S44): δ (ppm) 8.72 (s, 1H), 8.67 (d, 1H, *J* = 2.4 Hz), 8.37 (d, 2H, *J* = 9.2 Hz), 7.74 (dd, 1H, *J*_*1*_ = 8.9 Hz, *J*_*2*_ = 2.4 Hz), 7.54 (d, 1H, *J* = 8.9 Hz), 6.83 (d, 2H, *J* = 9.3 Hz), 3.11 (s, 6H).^13^C{^1^H} NMR (125 MHz, CD_2_Cl_2_, Figure S45): δ (ppm) 181.5, 153.0, 149.5, 146. 6, 145.0, 135.4, 131.5, 131.2, 129.4, 120.5, 119.5, 117.2, 112.2, 40.4. HRMS (APCI+, Figure S85): calcd. for C_17_H_15_BrNO_2_S^+^ [M + H^+^]: 376.0001, found: 376.0000.

#### 2-(4-(Dimethylamino)phenyl)-3-hydroxy-4-thioxo-4*H*-chromene-6,7-dicarbonitrile (**18**)

The compound was prepared according to the general procedure for the synthesis of 3-hydroxyflavothione derivatives from compound **12** (25 mg, 0.075 mmol, 1.00 equiv) and Lawesson’s reagent (37 mg, 0.091 mmol, 1.20 equiv). The mixture was refluxed in dry toluene for 30 min. The reaction progress was monitored with TLC (dichloromethane/methanol, 95:5). The crude product was purified by column chromatography (dichloromethane/*n*-hexane from 1:1 to pure dichloromethane). Yield: 9 mg (35%). Dark solid (dec < 181 °C). ^1^H NMR (500 MHz, CD_2_Cl_2_, Figure S46): δ (ppm) 8.96 (s, 1H), 8.39 (d, *J* = 9.5 Hz, 2H), 8.10 (s, 1H), 8.06 (s, 1H), 6.85 (d, *J* = 9.5 Hz, 2H), 3.15 (s, 6H). ^13^C{^1^H} NMR (125 MHz, CD_2_Cl_2_, Figure S47): δ (ppm) 178.6, 153.6, 151.0, 148.3, 145.7, 135.5, 132.0, 130.1, 125.1, 166.6, 115.6, 115.4, 115.2, 112.4, 11.8, 40.3. HRMS (APCI+, Figure S86): calculated for C_19_H_14_N_3_O_2_S^+^ [M + H^+^]: 348.0801, 348.0802.

#### 3-Methoxyflavothione (**20**)

The synthesis of **20** was performed according to the general method of the synthesis of 3-hydroxyflavothione from **19** (0.125 g, 0.49 mmol, 1.00 equiv) with Lawesson’s reagent (0.240 g, 0.59 mmol, 1.20 equiv). The crude product was purified on a silica column with dichloromethane as an eluent and recrystallized from ethanol. Yield: 0.077g (58%). Red crystalline solid (dec < 117 °C). ^1^H NMR (500 MHz, DMSO-*d*_6_, Figure S50): δ (ppm) 8.51 (dd, 1H, *J*_*1*_ = 8.2 Hz, *J*_*2*_ = 1.3 Hz), 8.19 (m, 2H), 7.86 (m, 2H), 7.65 (m, 3H), 7.56 (m, 1H), 3.71 (s, 3H) ^13^C{^1^H} NMR (125 MHz, DMSO-*d*_6_, Figure S51): δ (ppm) 195.3, 150. 6, 150.0, 149.9, 134.1, 131.5, 130.3, 130.2, 128.9, 127. 9, 126.4, 119.0, 58.7. HRMS (APCI+, Figure S88): calcd. for C_16_H_13_O_2_S^+^ [M + H^+^]: 269.0631, found: 269.0634. This compound was also reported elsewhere.^87^

### Preparation of Flavonolate and Flavothionolate Anions

3-Hydroxyflavone (**1**–**12**) or 3-hydroxyflavothione (**13**–**18**) derivatives were dissolved in dry methanol or ethanol and titrated with a solution of sodium methoxide (**1**–**5** and **13**–**17**; *c*(NaOCH_3_) ≈ 0.025 M) or sodium hydroxide (**3, 6–12, 17**–**19**; *c*(NaOH) ≈ 0.05 M) in methanol, monitored by UV–vis spectroscopy (the deprotonated form). The titration continued until the total disappearance of the band of the acid form.

## Data Availability

The data underlying this study are available in the published article and its Supporting Information.

## Supporting Information Available

Materials and methods; experimental procedures; kinetic studies; analyses of photoproducts; transient spectroscopy data; determination of p*K*_a_; and NMR, optical, transient, and HRMS spectra (PDF)
