# Supplementary material for: Structure–Photoreactivity Relationship Study of Substituted 3-Hydroxyflavones and 3-Hydroxyflavothiones for Improving Carbon Monoxide Photorelease
Source: J Org Chem. 2024 Mar 22;89(7):4888–903. doi: 10.1021/acs.joc.4c00070 (PMC11002828; doi:10.1021/acs.joc.4c00070)
Supplement: Supplementary file 1 — jo4c00070_si_001.pdf [file jo4c00070_si_001.pdf]

SUPPORTING INFORMATION

Structure-Photoreactivity Relationship Study of Substituted 3-Hydroxyflavones and 3-Hydroxyflavothiones for Improving Carbon Monoxide Photorelease

Yann A. Jézéquel,[†,‡,$] Filip Svěrák,[†,$] Andrea Ramundo,[†,‡] Vojtěch Orel,[†] Marek Martínek,[†,‡] Petr Klán[†,‡,*]

[†] Department of Chemistry, Faculty of Science, Masaryk University, Kamenice 5, 625 00, Brno, Czech Republic.

[‡] RECETOX, Faculty of Science, Masaryk University, Kamenice 5, 625 00, Brno, Czech Republic.

[$]Those authors contributed equally to this work.

* klan@sci.muni.cz

Table of Content

^{1}H NMR and ^{13}C NMR Spectra S2
HRMS Spectra S35
Absorption and Emission Spectra S45
Experimental Procedures S63
Emission Spectra in Solvents of Different Polarity S66
Kinetic of Degradation in Degassed and Aerated Solutions S69
Analysis of Photoproducts S70
Transient Spectroscopy S74
Spectroscopic Determination of the pK_a S80
Sensitivity of Flavothione **13** Toward Singlet Oxygen S81
References S83

^{1}H NMR and ^{13}C NMR Spectra

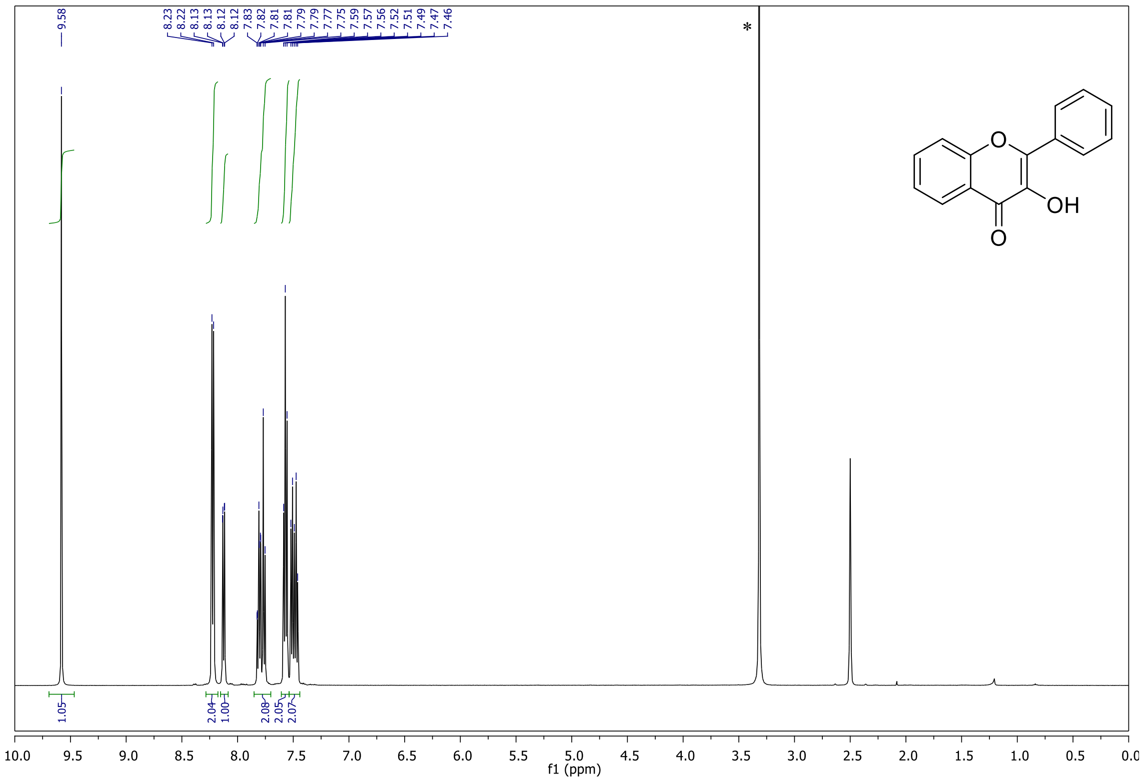

Figure S1. ^{1}H NMR (500 MHz, DMSO-d_6): **1**. Residual signal of water is marked with an asterisk.

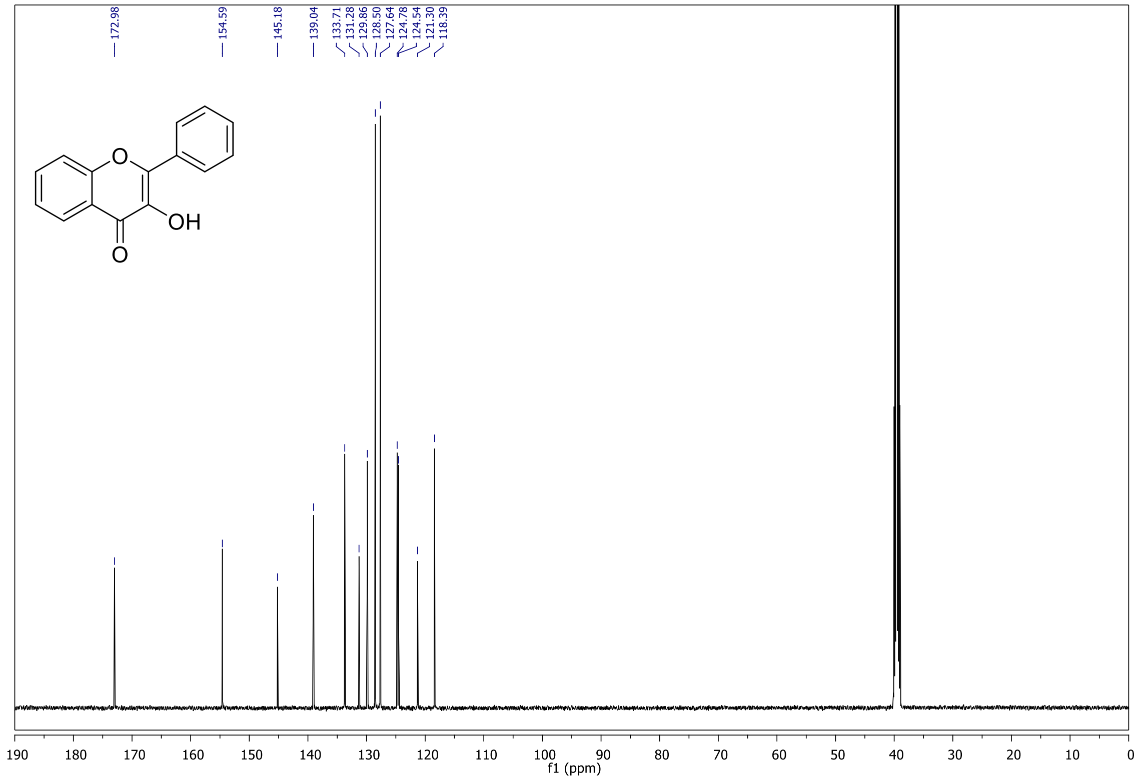

Figure S2. $^{13}C\{^{1}H\}$ NMR (125 MHz, DMSO-d_6): **1**.

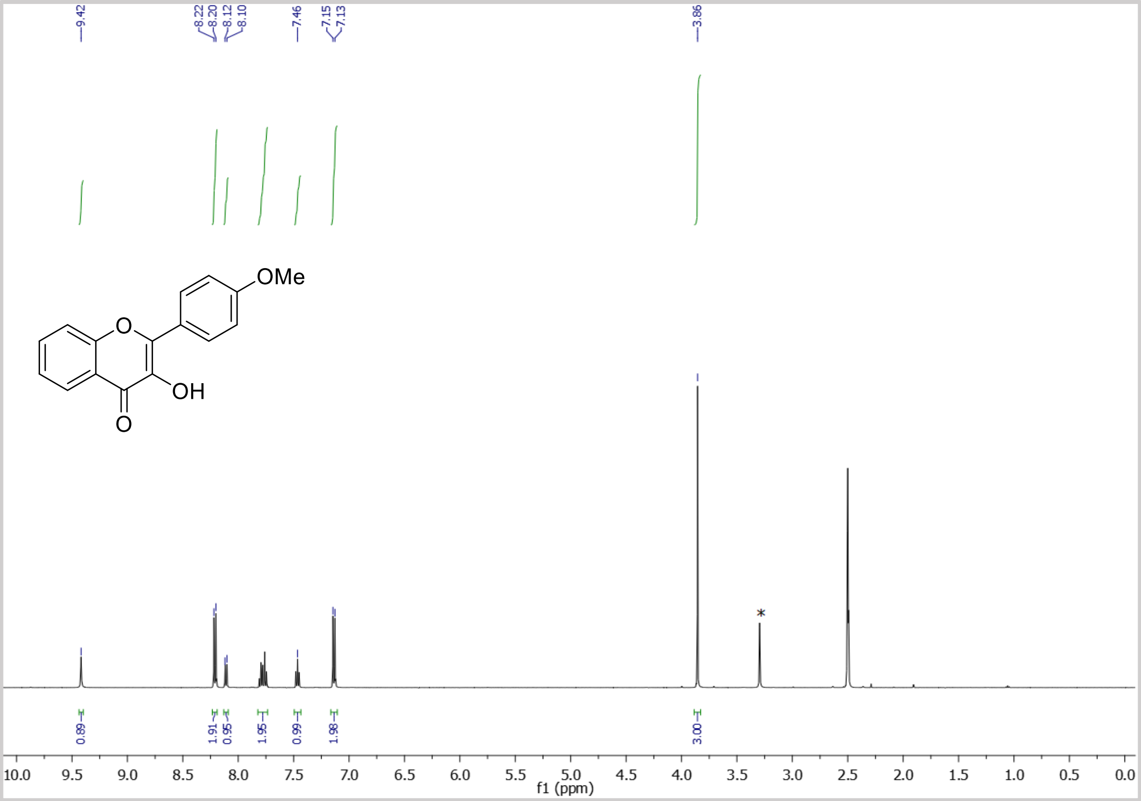

Figure S3. ^{1}H NMR (500 MHz, DMSO-d_6): **2**. Residual signal of water is marked with an asterisk.

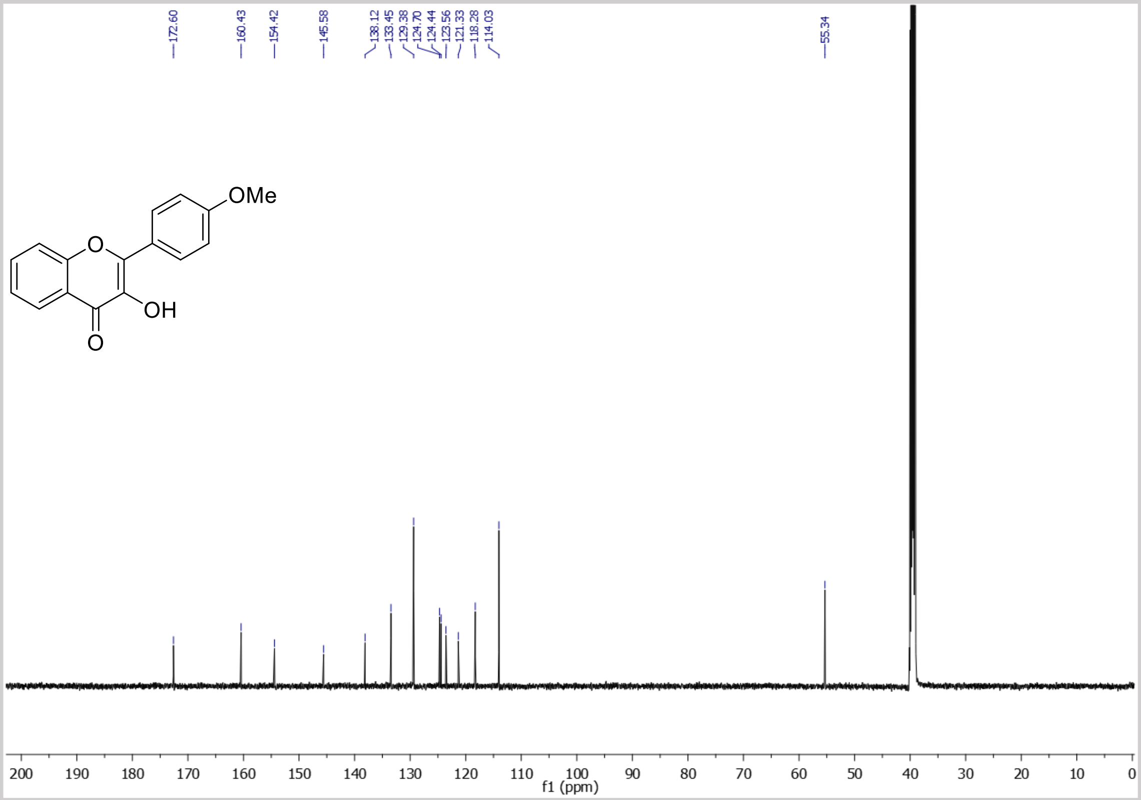

Figure S4. . $^{13}C\{^{1}H\}$ NMR (125 MHz, DMSO-d_6): **2**.

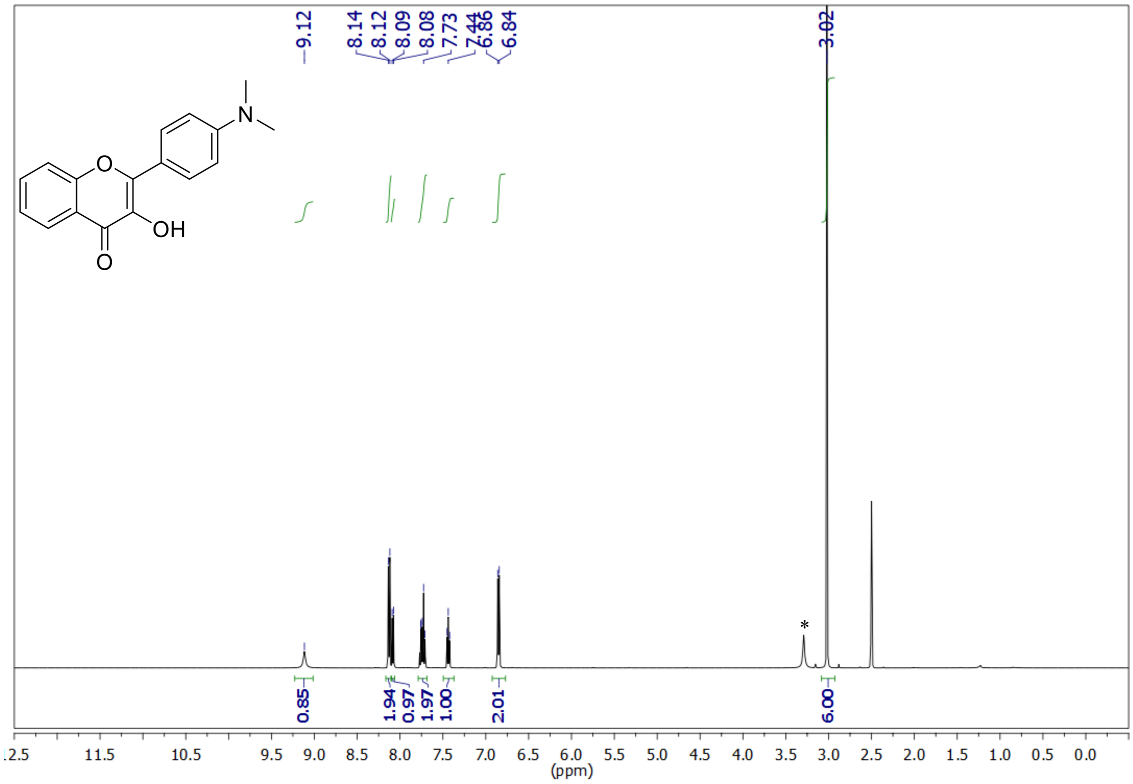

Figure S5. ^{1}H NMR (500 MHz, DMSO-d_6): **3**. Residual signal of water is marked with an asterisk.

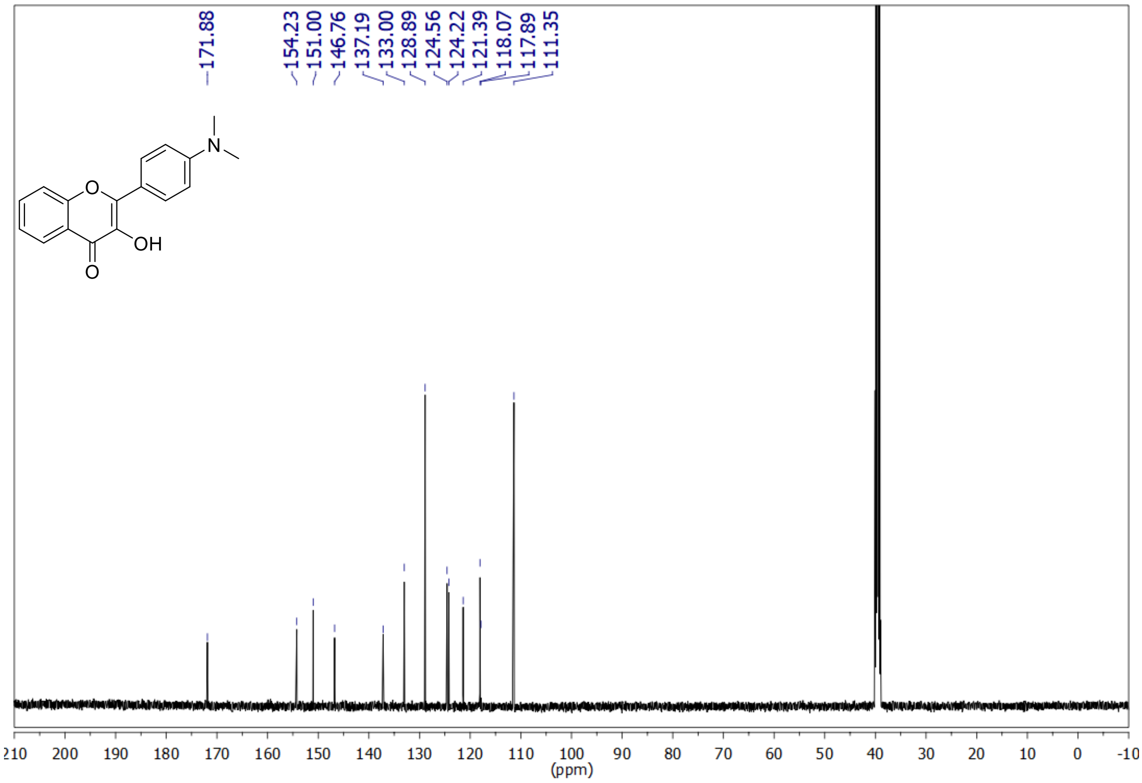

Figure S6. $^{13}C\{^{1}H\}$ NMR (125 MHz, DMSO-d_6): **3**.

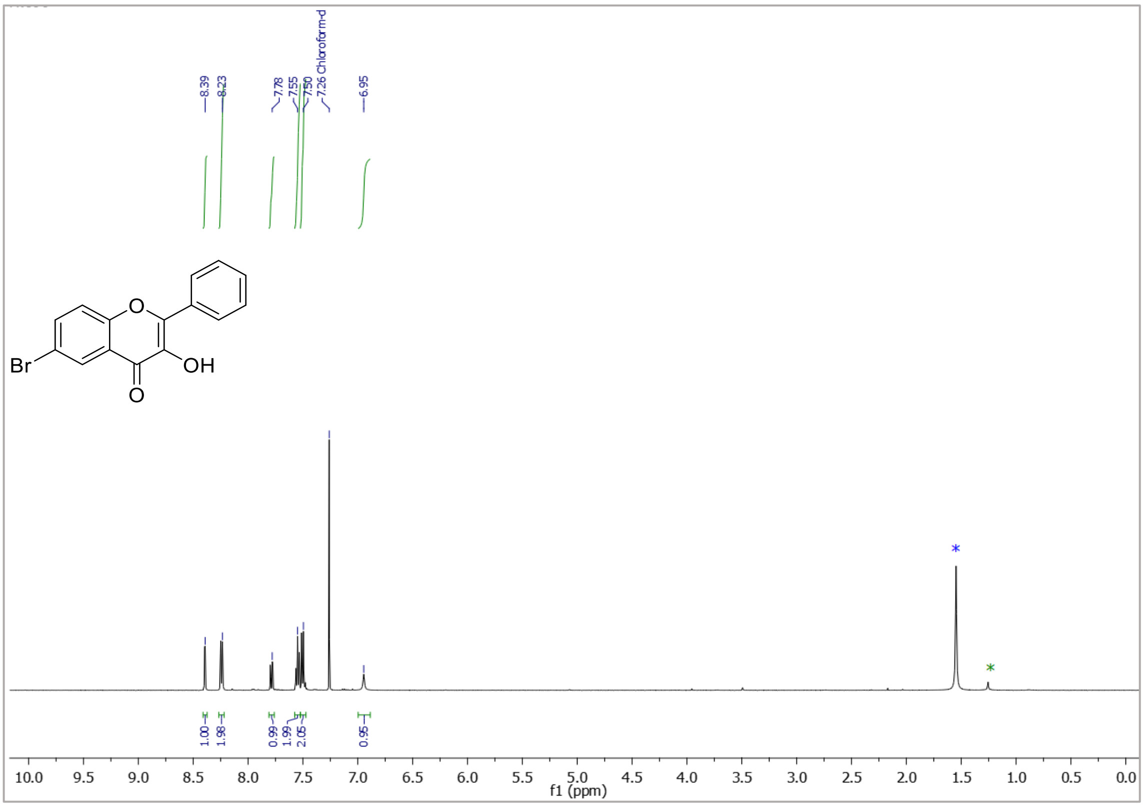

Figure S7. ^{1}H NMR (500 MHz, $CDCl_3$): **4**. Residual signals of water (blue) and grease (green) are marked with asterisks.

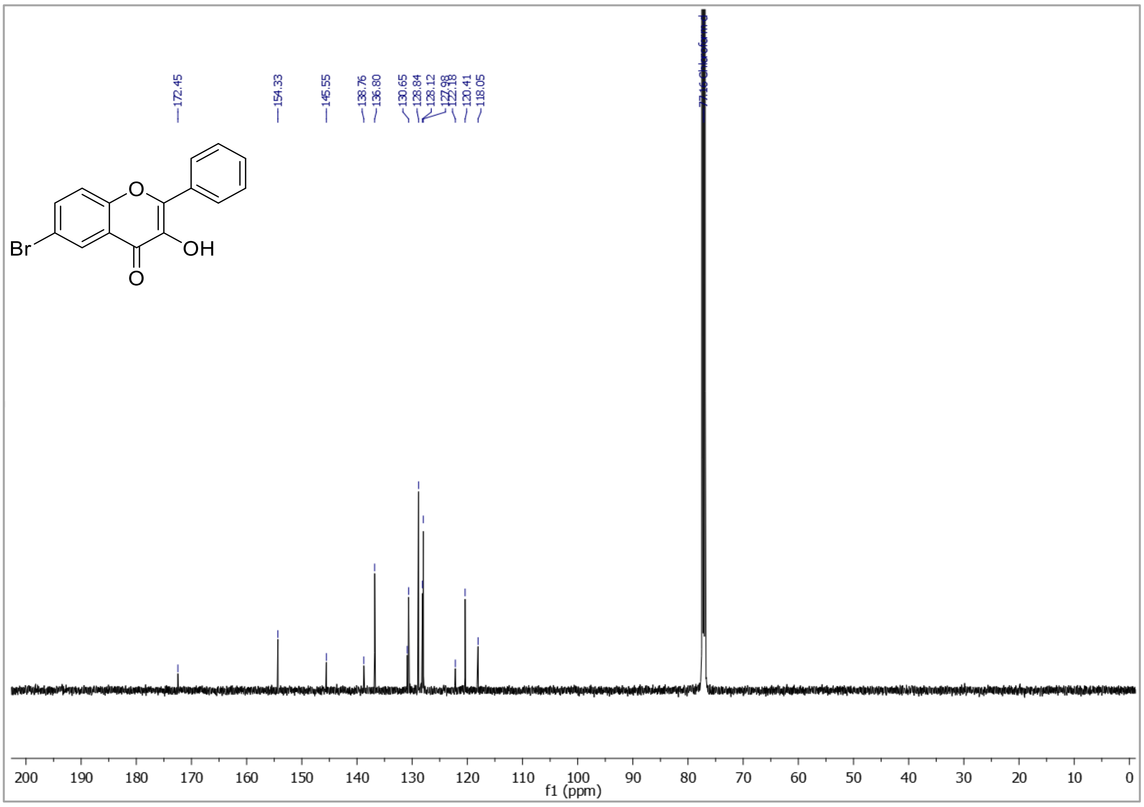

Figure S8. $^{13}C\{^{1}H\}$ NMR (125 MHz, $CDCl_3$): **4**.

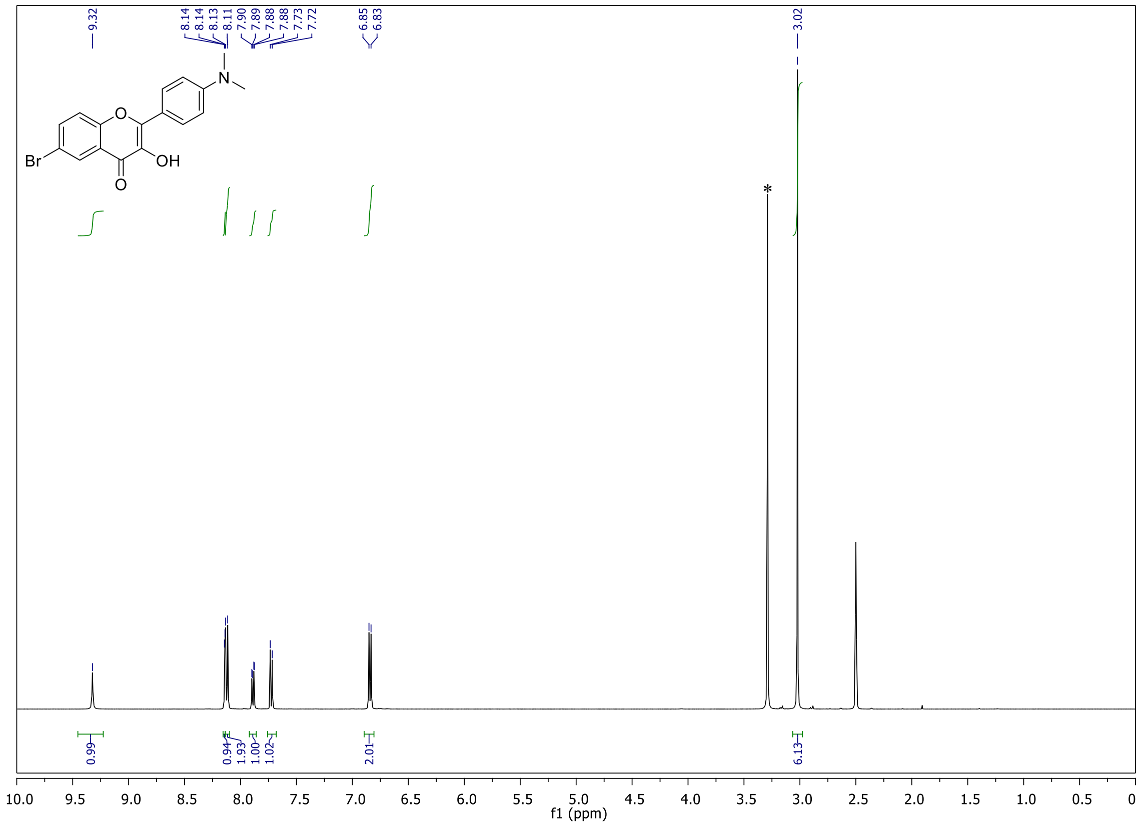

Figure S9. ^{1}H NMR (500 MHz, DMSO-d_6): **5**. Residual signal of water is marked with an asterisk.

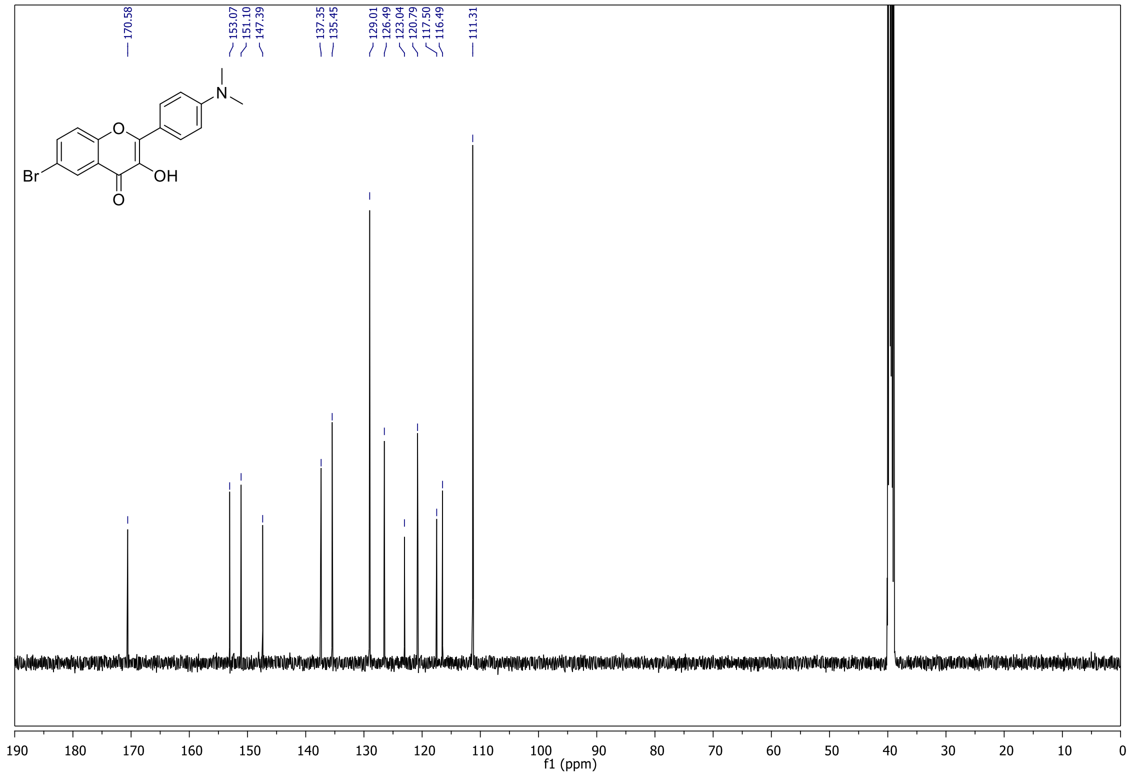

Figure S10. ^{13}C{^{1}H} NMR (125 MHz, DMSO-d_6): **5**.

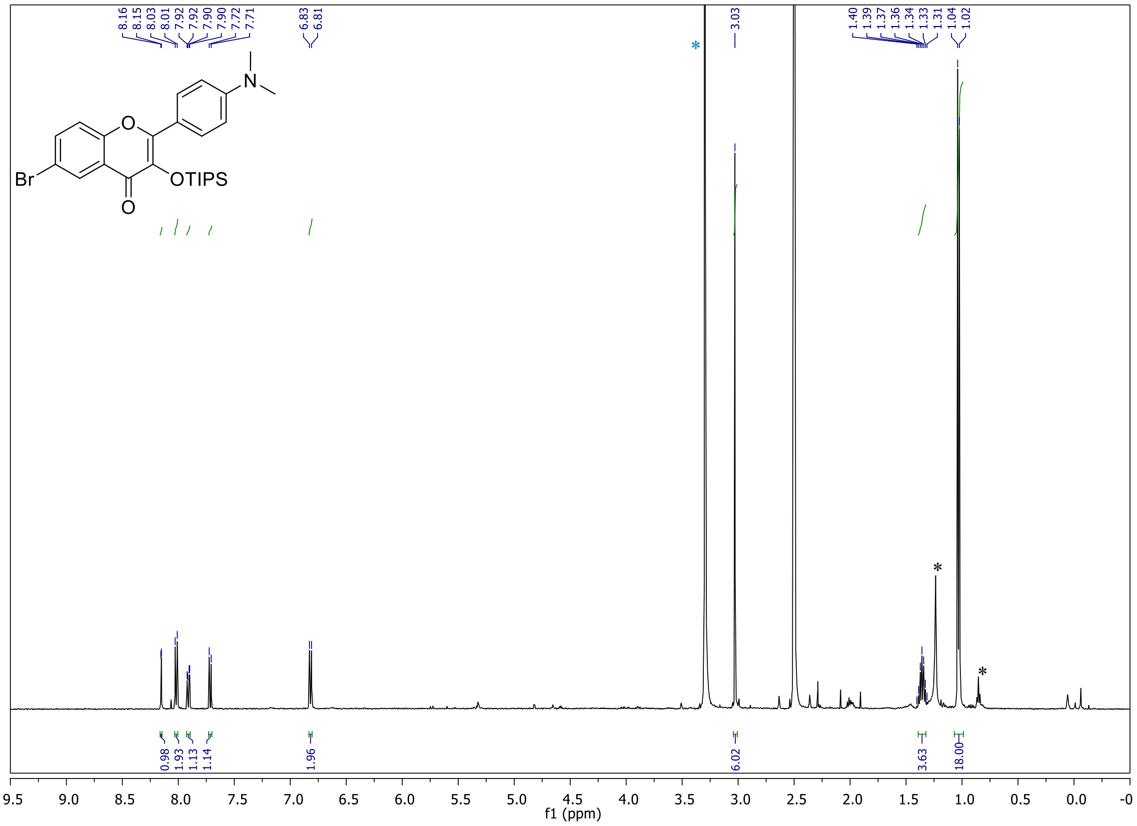

Figure S11. ^{1}H NMR (500 MHz, DMSO-d_6): **5-TIPS**. Residual signals of water (blue) and *n*-hexane (black) are marked with asterisks.

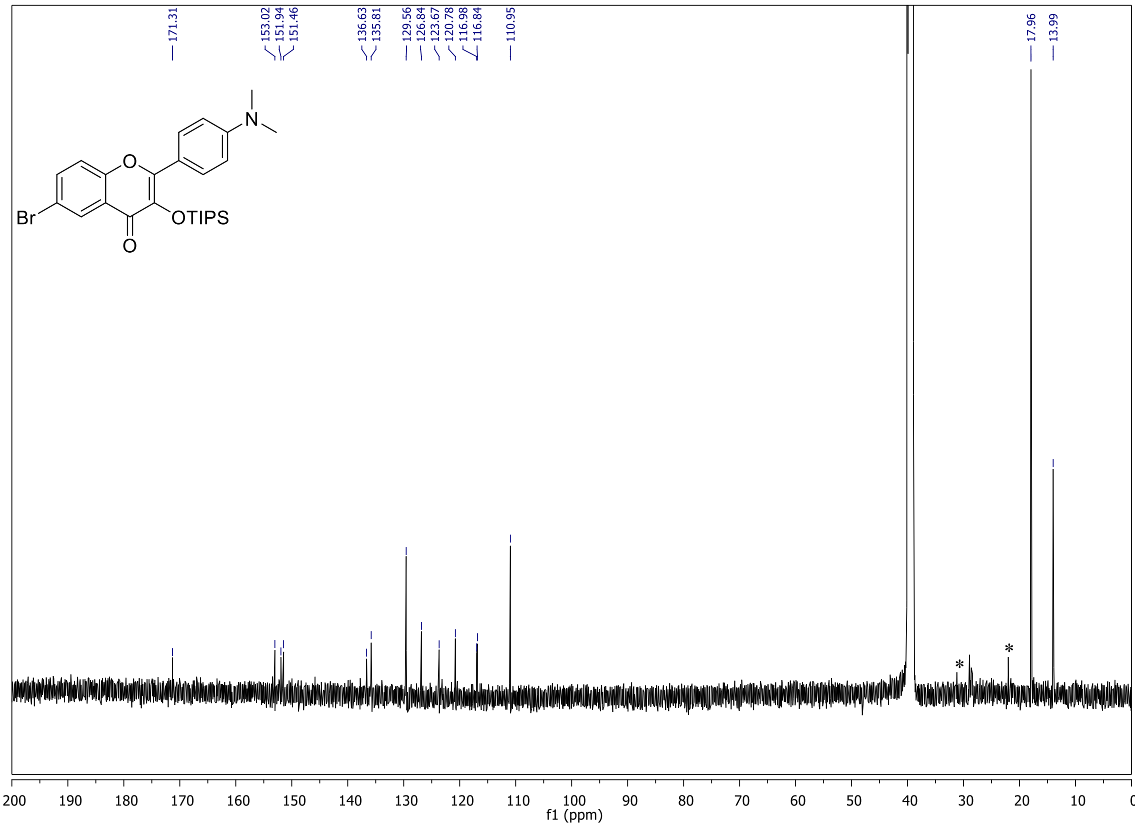

Figure S12. $^{13}C\{^{1}H\}$ NMR (125 MHz, DMSO-d_6): **5-TIPS**. Residual signals of *n*-hexane are marked with an asterisk.

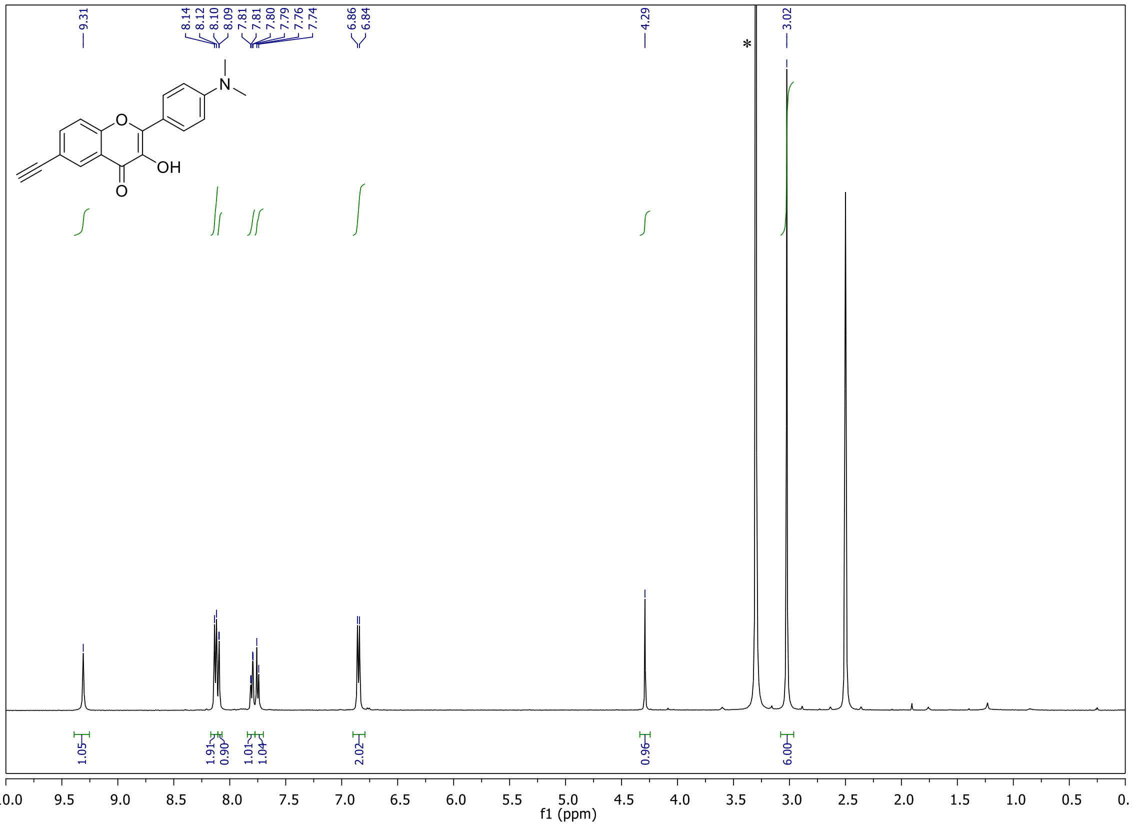

Figure S13. ^{1}H NMR (500 MHz, DMSO-d_6): **6**. Residual signal of water is marked with an asterisk.

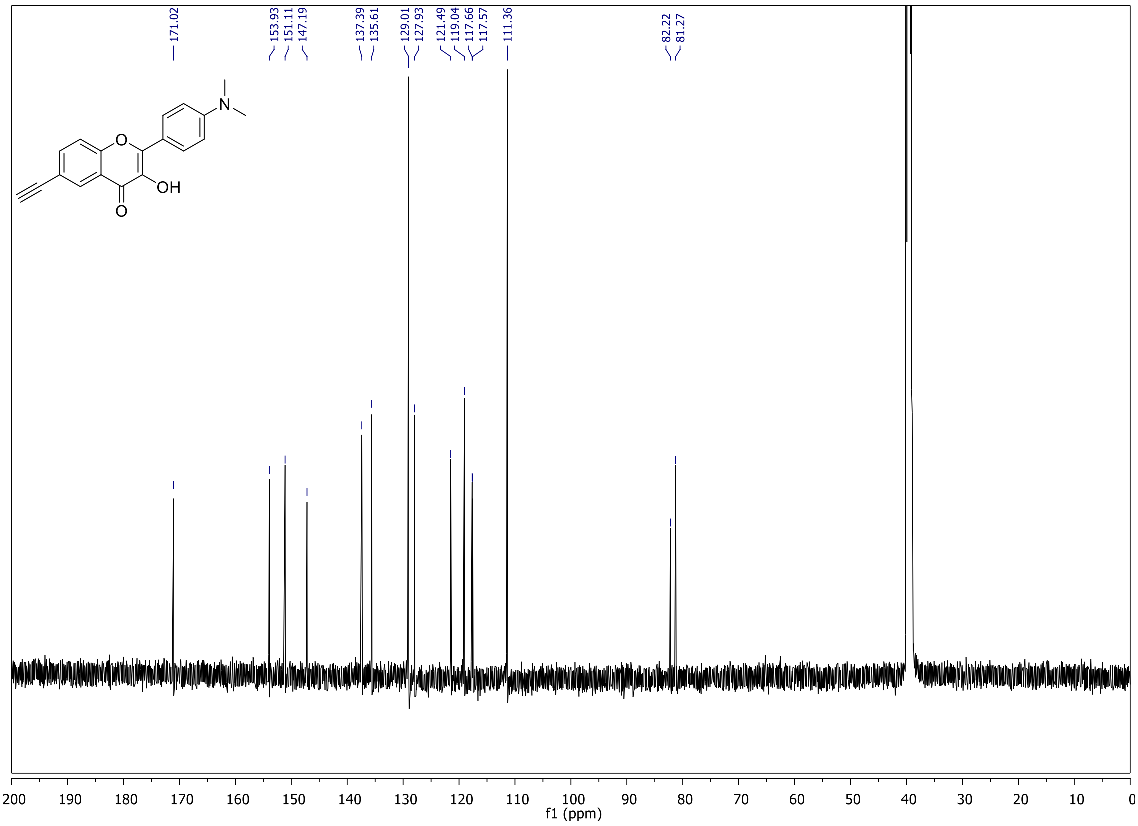

Figure S14. ^{13}C{^{1}H} NMR (125 MHz, DMSO-d_6): **6**.

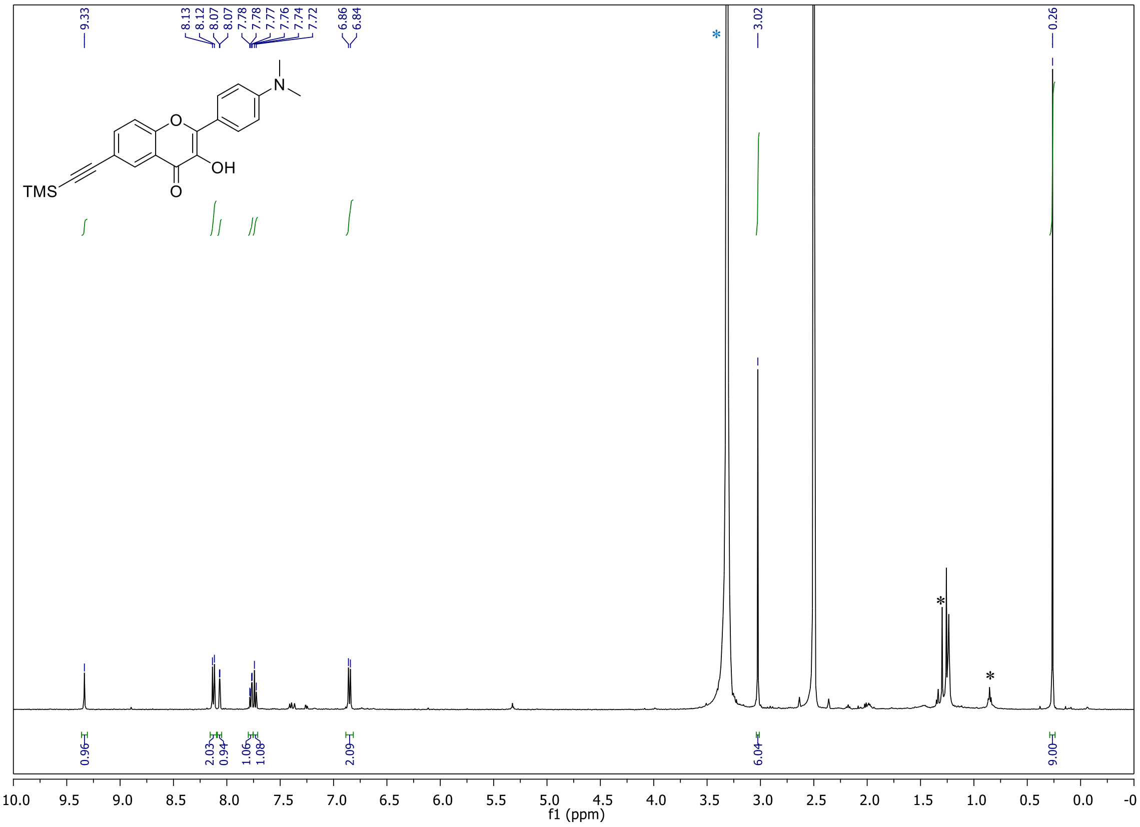

Figure S15. ^{1}H NMR (500 MHz, DMSO-d_6): **6-TMS**. Asterisks denote residual signals of *n*-hexane (black) and water (blue).

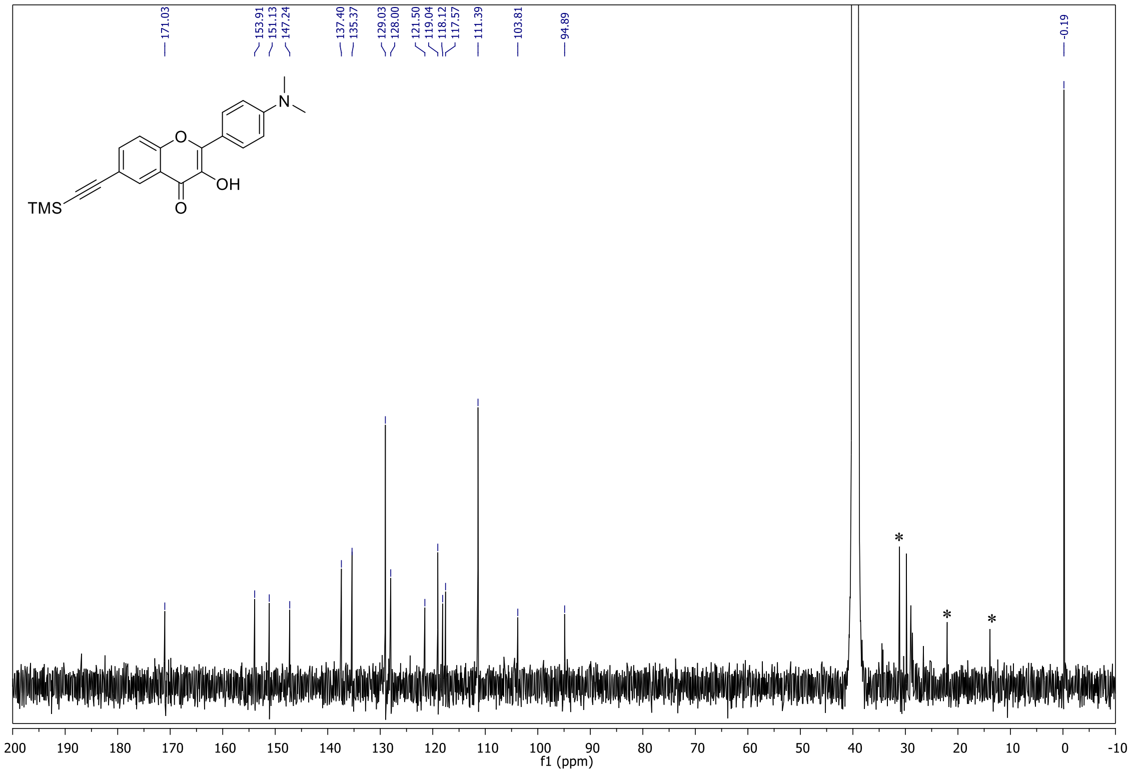

Figure S16. $^{13}C\{^{1}H\}$ NMR (125 MHz, DMSO-d_6): **6-TMS**. The asterisk denotes residual signals of *n*-hexane.

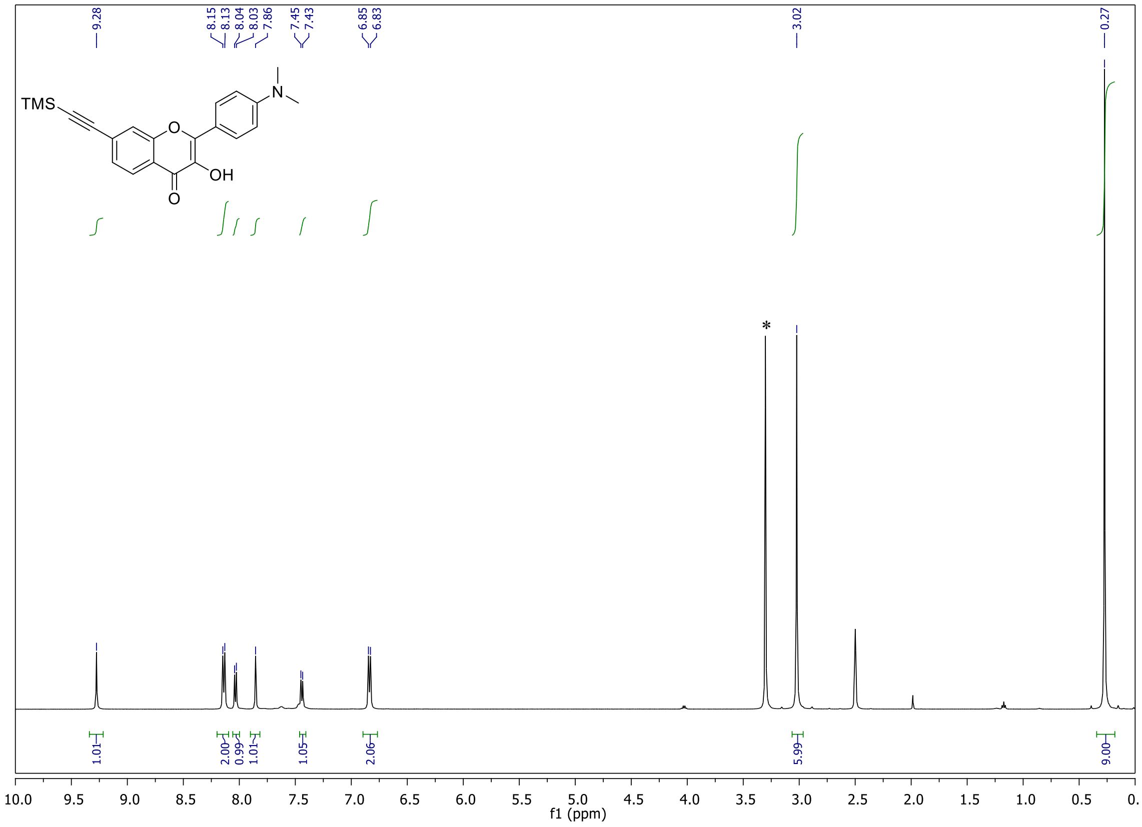

Figure S17. ^{1}H NMR (500 MHz, DMSO-d_6): **7-TMS**. The residual signal of water is marked with an asterisk.

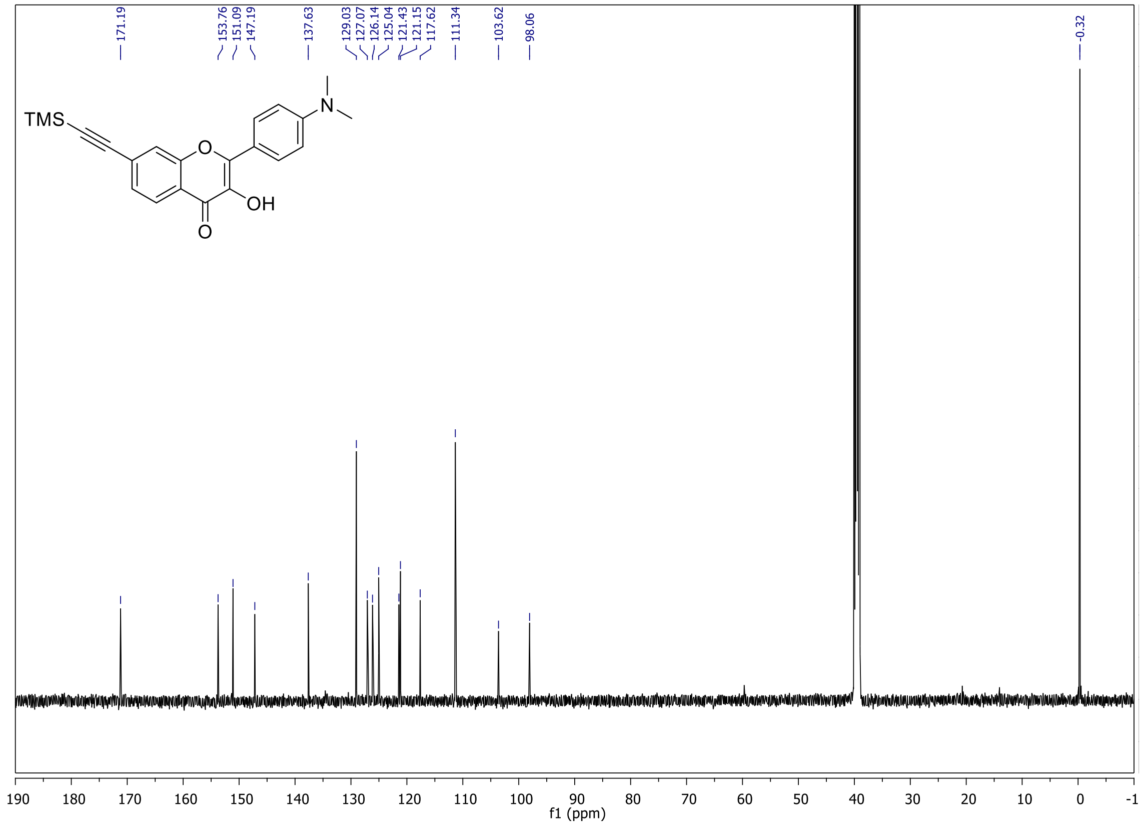

Figure S18. ^{13}C{^{1}H} NMR (125 MHz, DMSO-d_6): **7-TMS**.

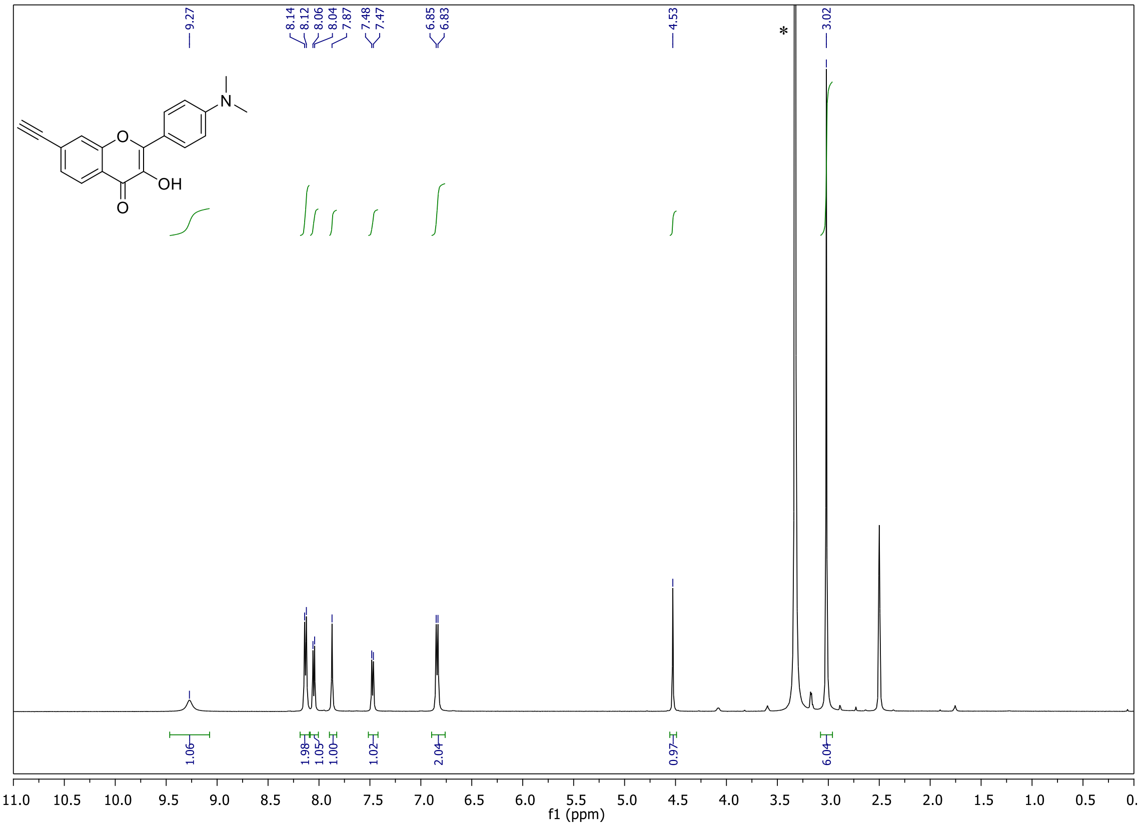

Figure S19. ^{1}H NMR (500 MHz, DMSO-d_6): **7**. Residual signal of water is marked with an asterisk.

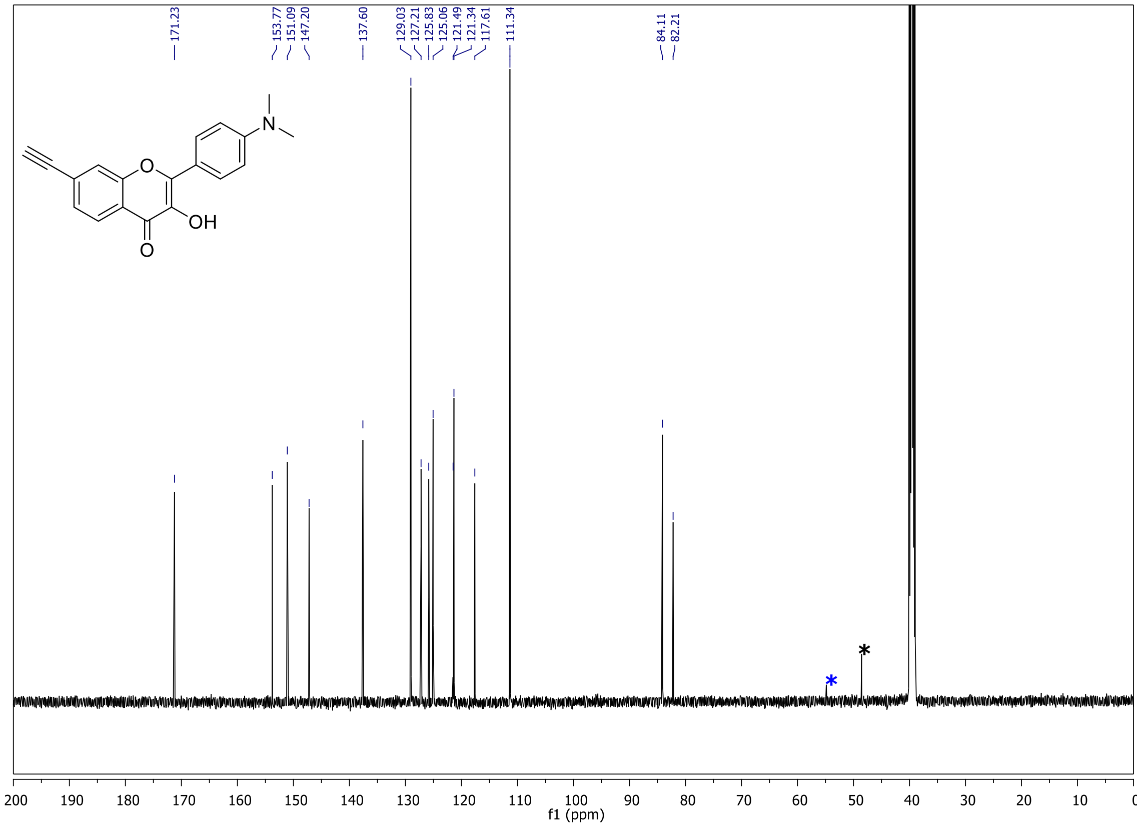

Figure S20. ^{13}C{^{1}H} NMR (125 MHz, DMSO-d_6): **7**. Residual signals of dichloromethane (blue) and methanol (black) are marked with asterisks.

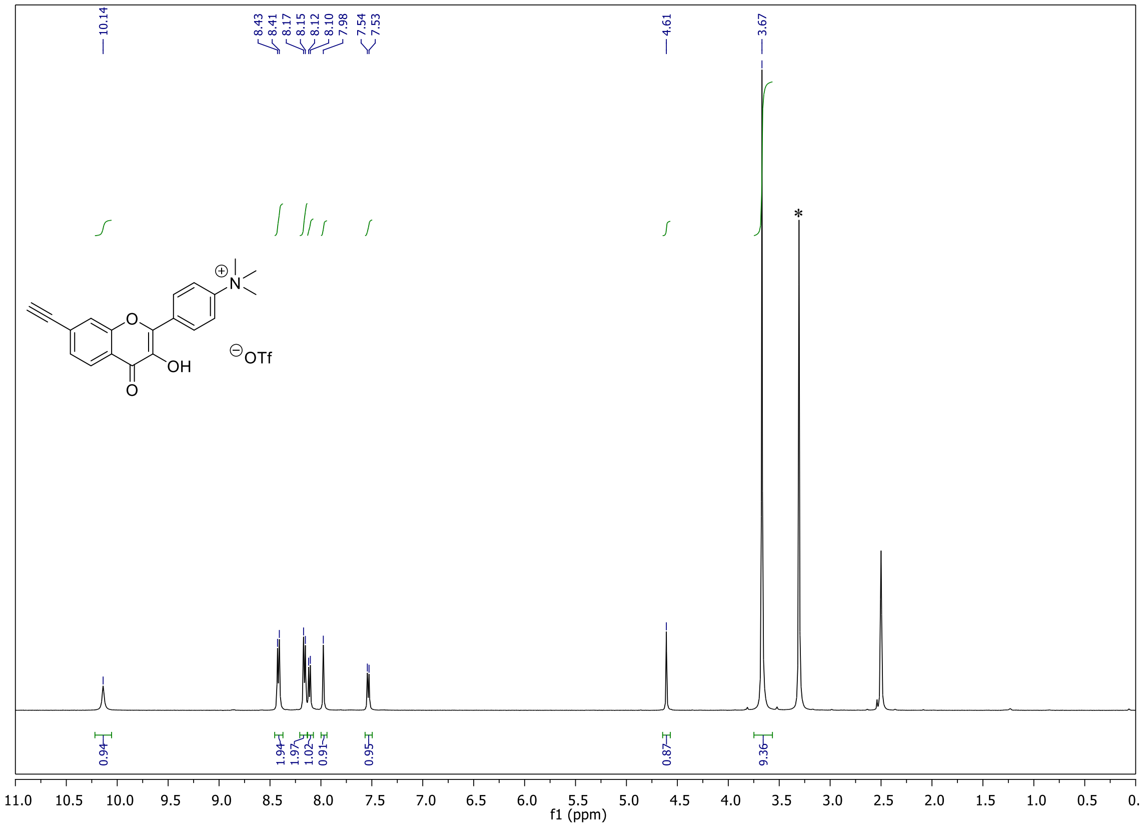

Figure S21. ^{1}H NMR (500 MHz, DMSO-d_6): **8**. The Residual signal of water is marked with an asterisk.

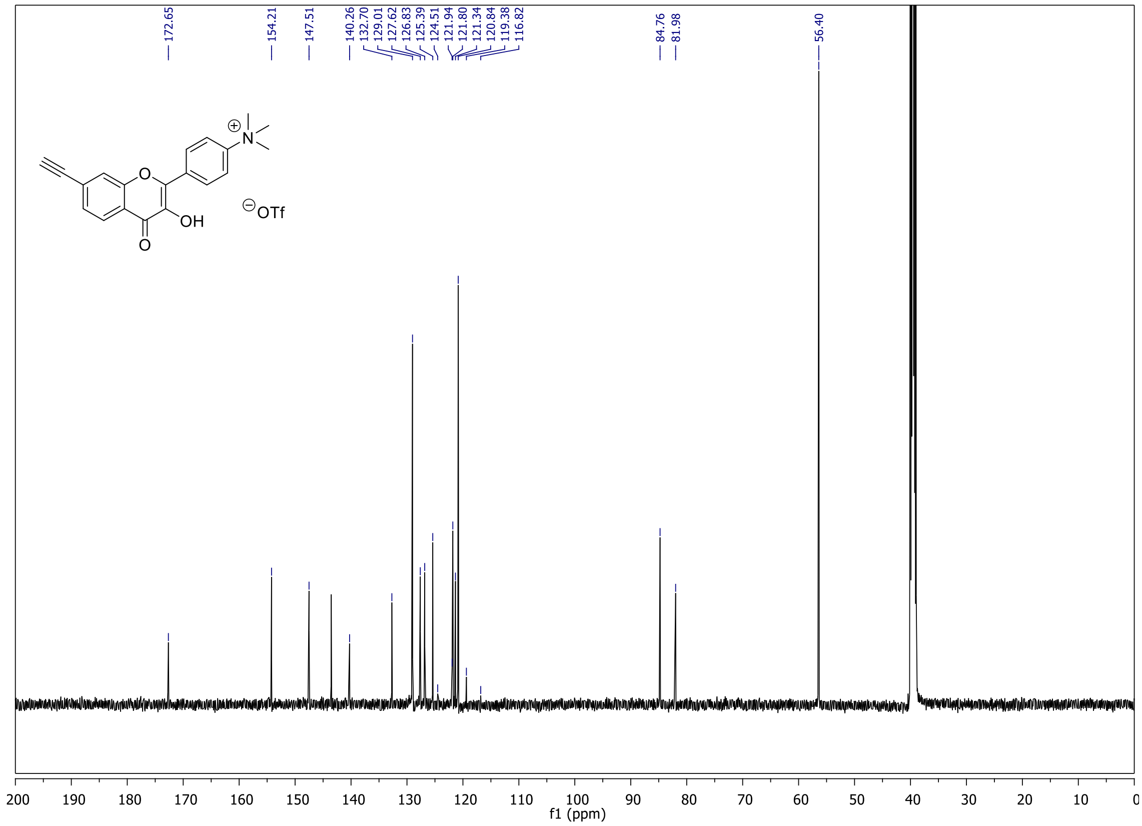

Figure S22. $^{13}C\{^{1}H\}$ NMR (125 MHz, DMSO-d_6): **8**.

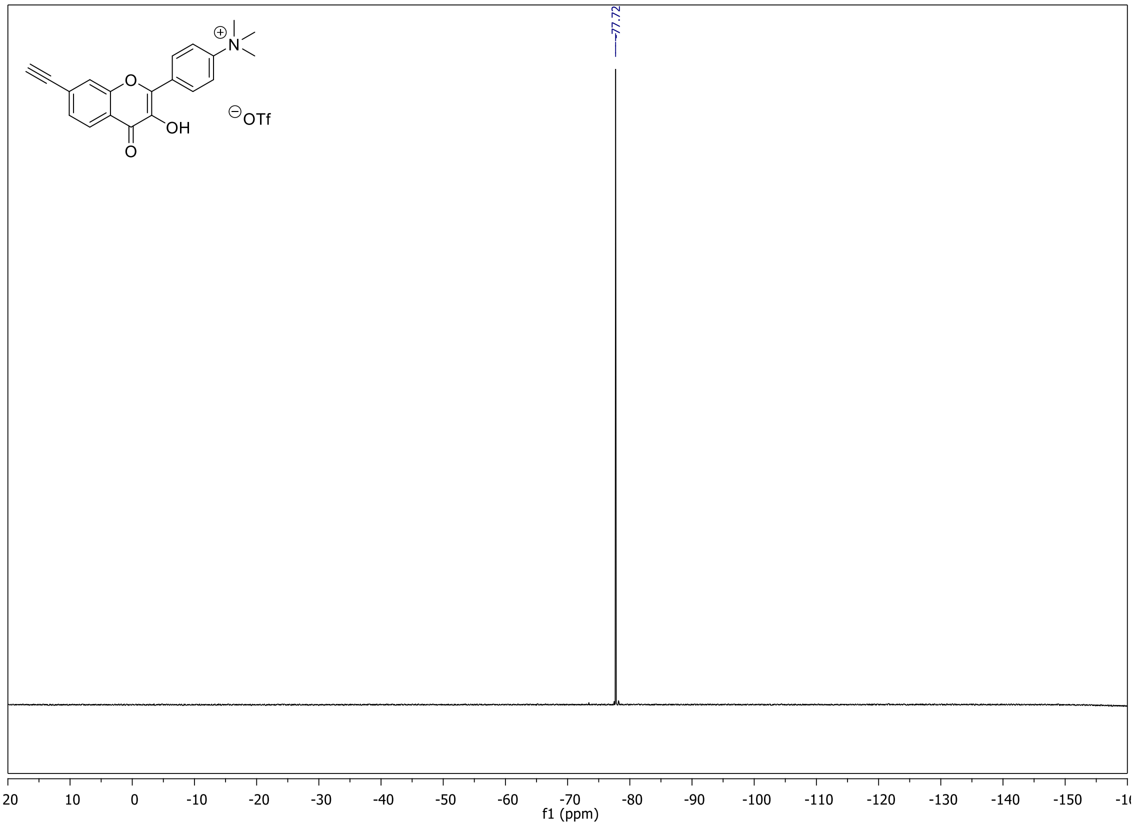

Figure S23. ^{19}F NMR (470 MHz, DMSO-d_6): **8**.

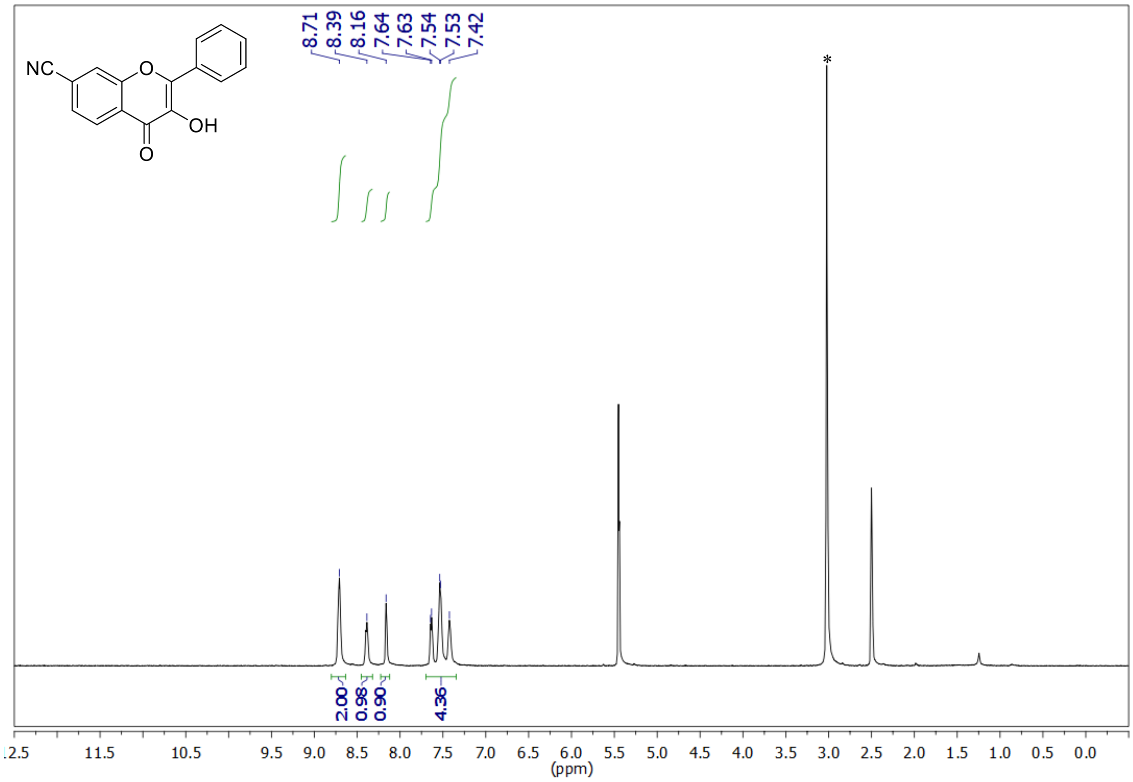

Figure S24. ^{1}H NMR (500 MHz, DMSO-d_6/CD_2Cl_2 2.5:97.5): **9**. Residual signal of water is marked with an asterisk.

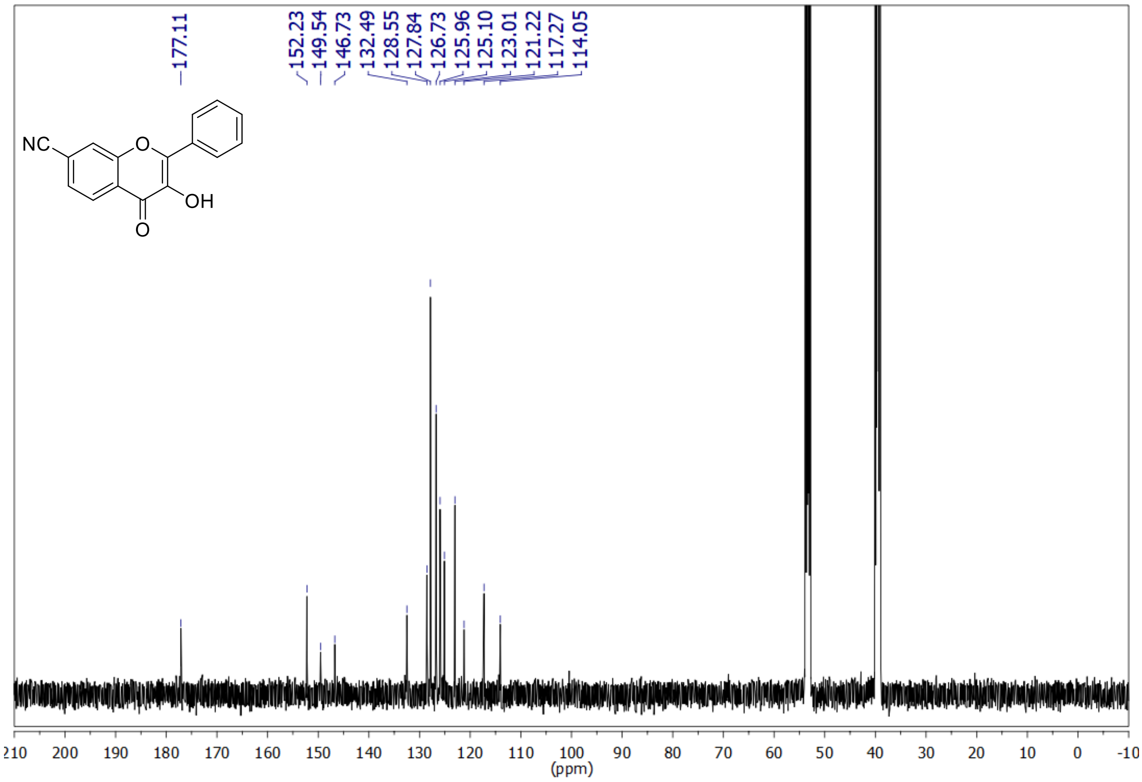

Figure S25. $^{13}C\{^{1}H\}$ NMR (125 MHz, DMSO-d_6/CD_2Cl_2 2.5:97.5): **9**.

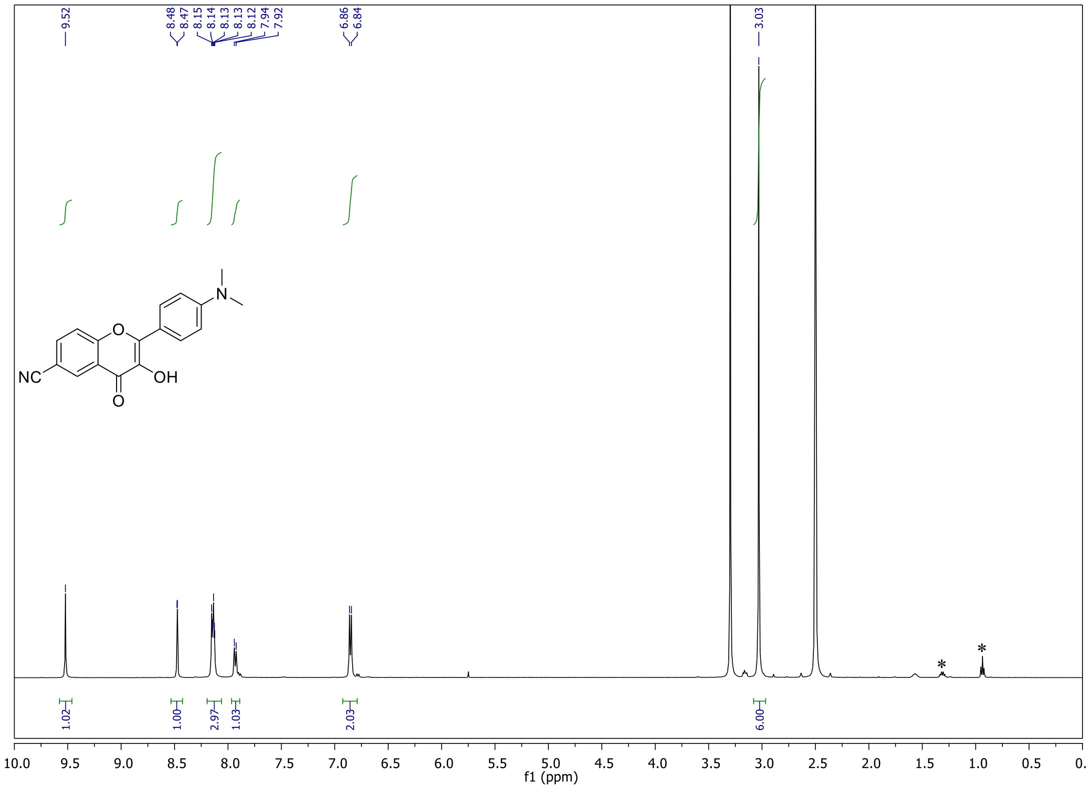

Figure S26. ^{1}H NMR (500 MHz, DMSO-d_6): **10**. Residual signals of *n*-hexane are marked with an asterisk.

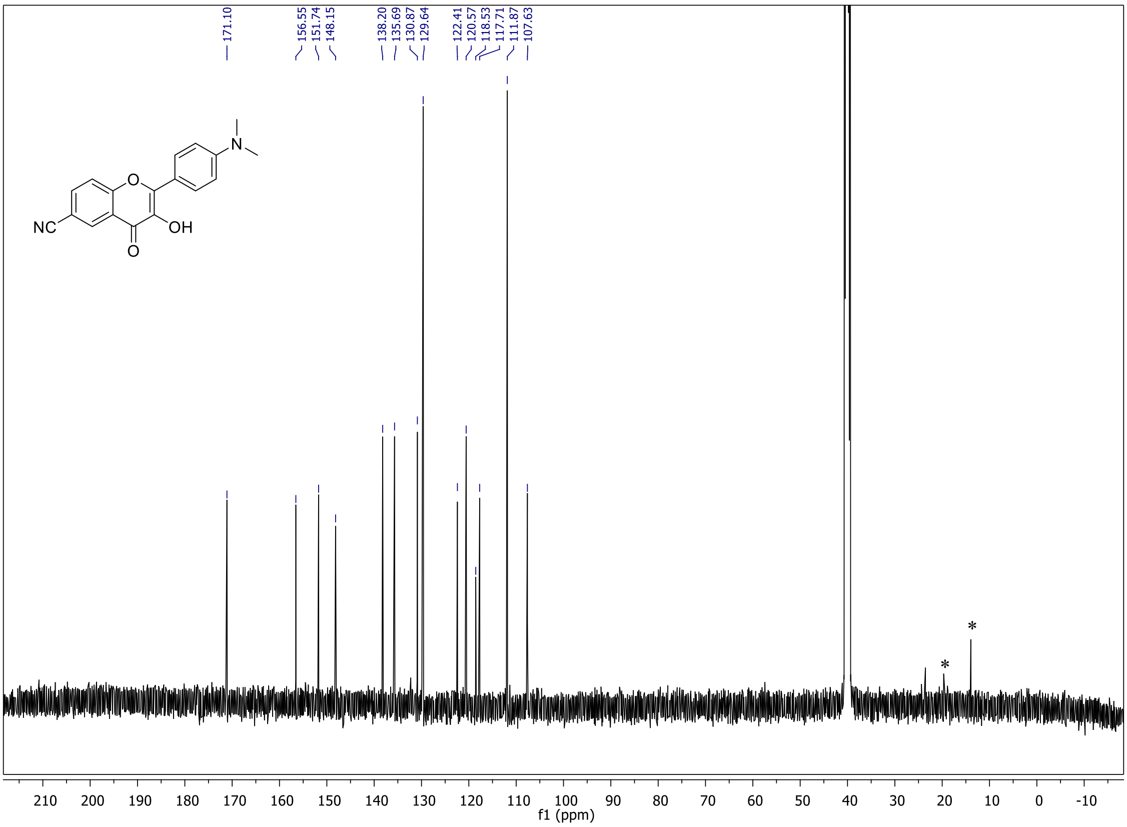

Figure S27. $^{13}C\{^{1}H\}$ NMR (125 MHz, DMSO-d_6):**10**. Residual signals of *n*-hexane are marked with an asterisk.

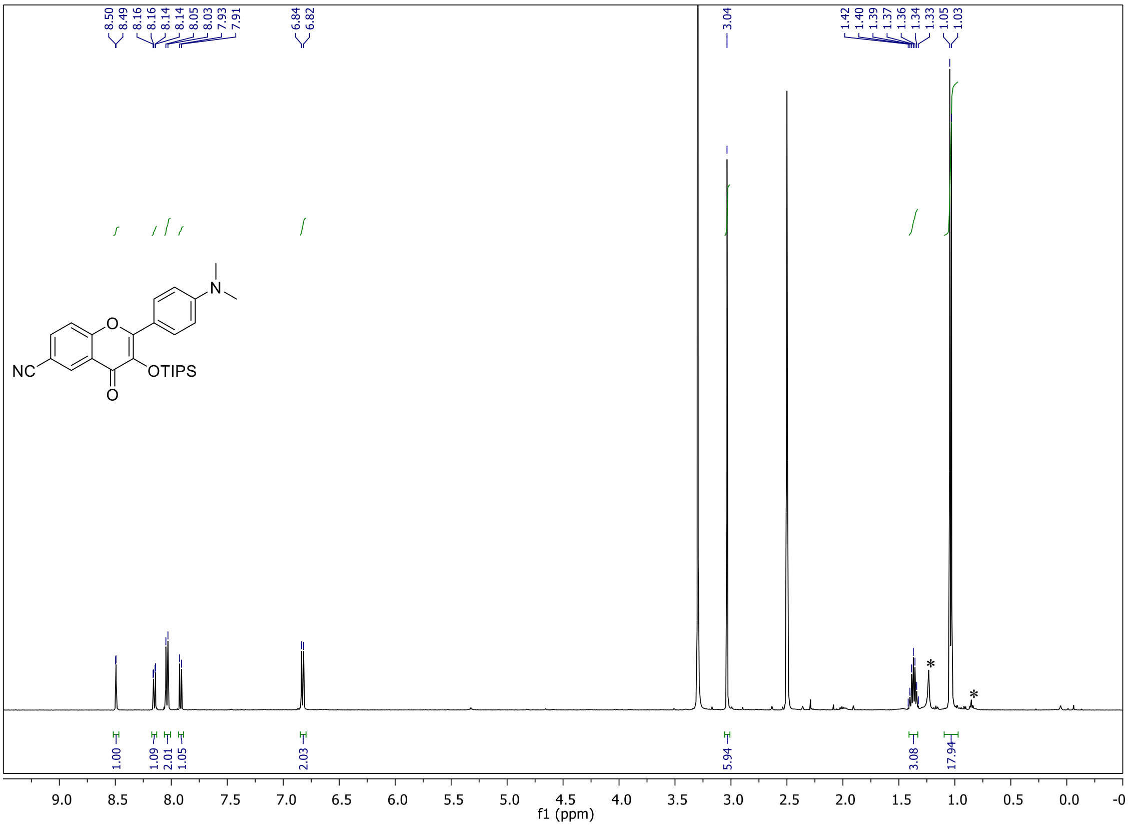

Figure S28. ^{1}H NMR (500 MHz, DMSO-d_6): **10-TIPS**. Residual signals of hexane are *n*-marked with an asterisk.

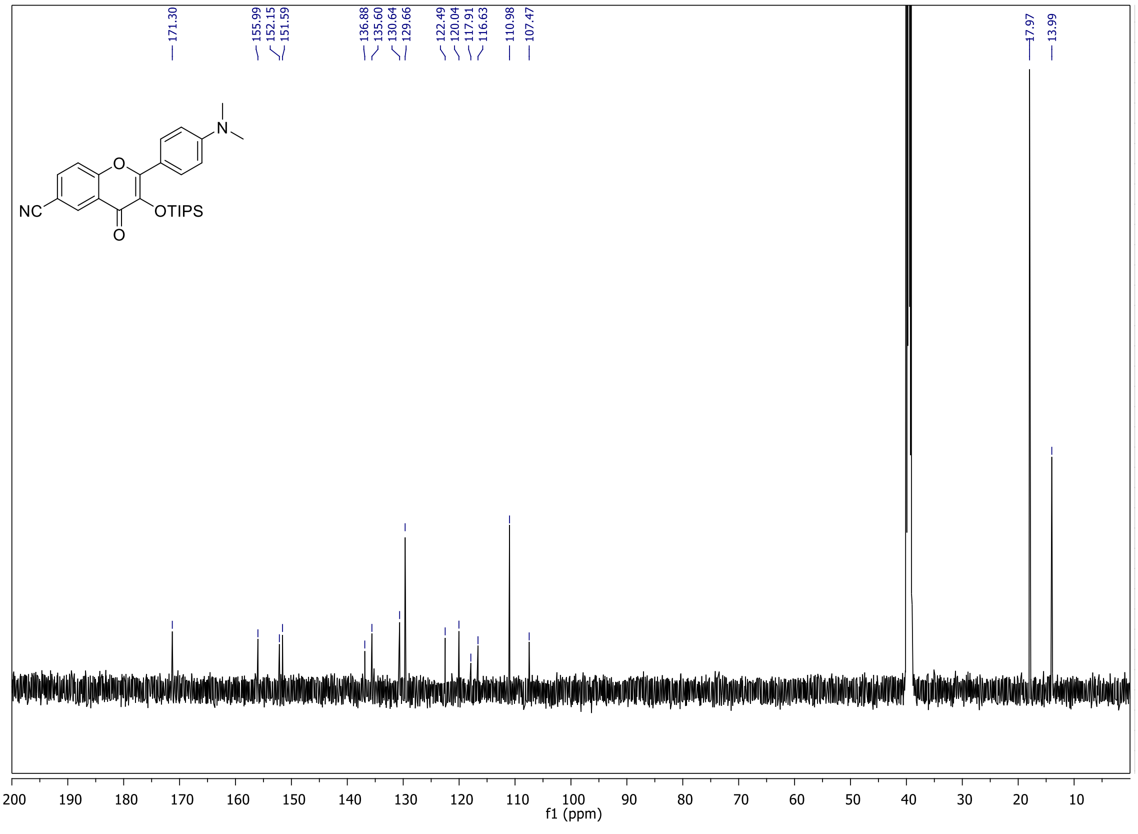

Figure S29. $^{13}C\{^{1}H\}$ NMR (125 MHz, DMSO-d_6): **10-TIPS**.

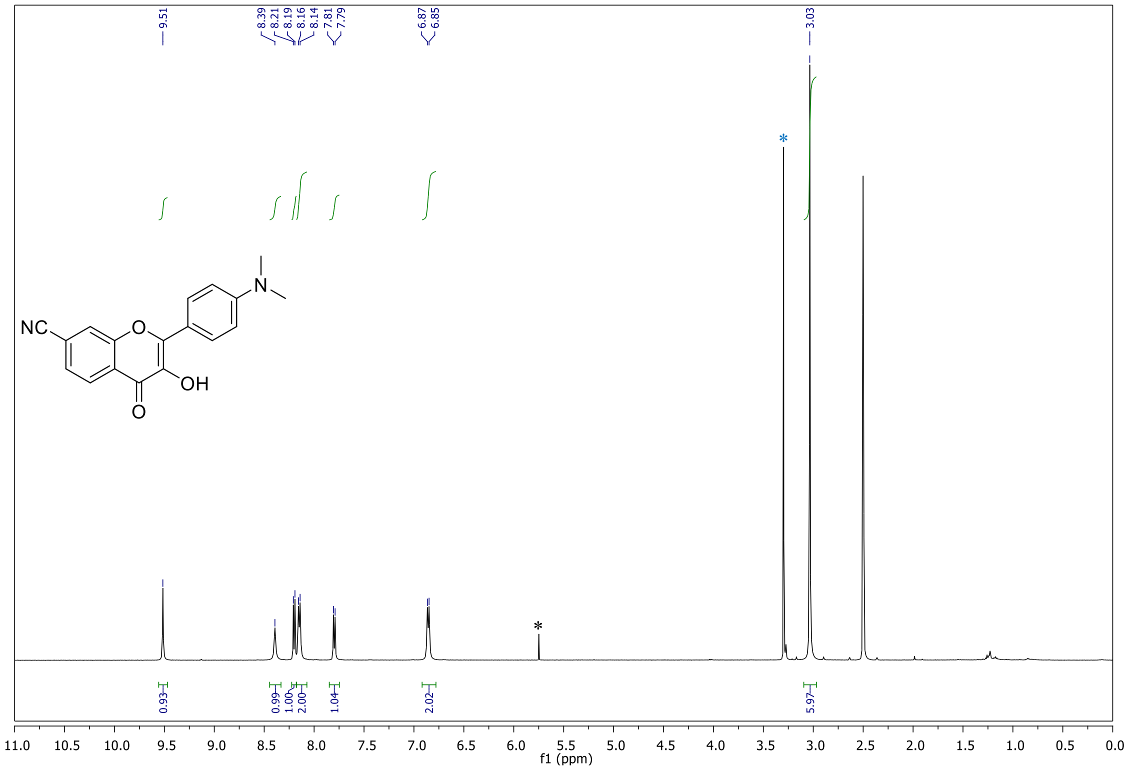

Figure S30. ^{1}H NMR (500 MHz, DMSO-d_6): **11**. Asterisk denotes residual signals of dichloromethane (black) and water (blue).

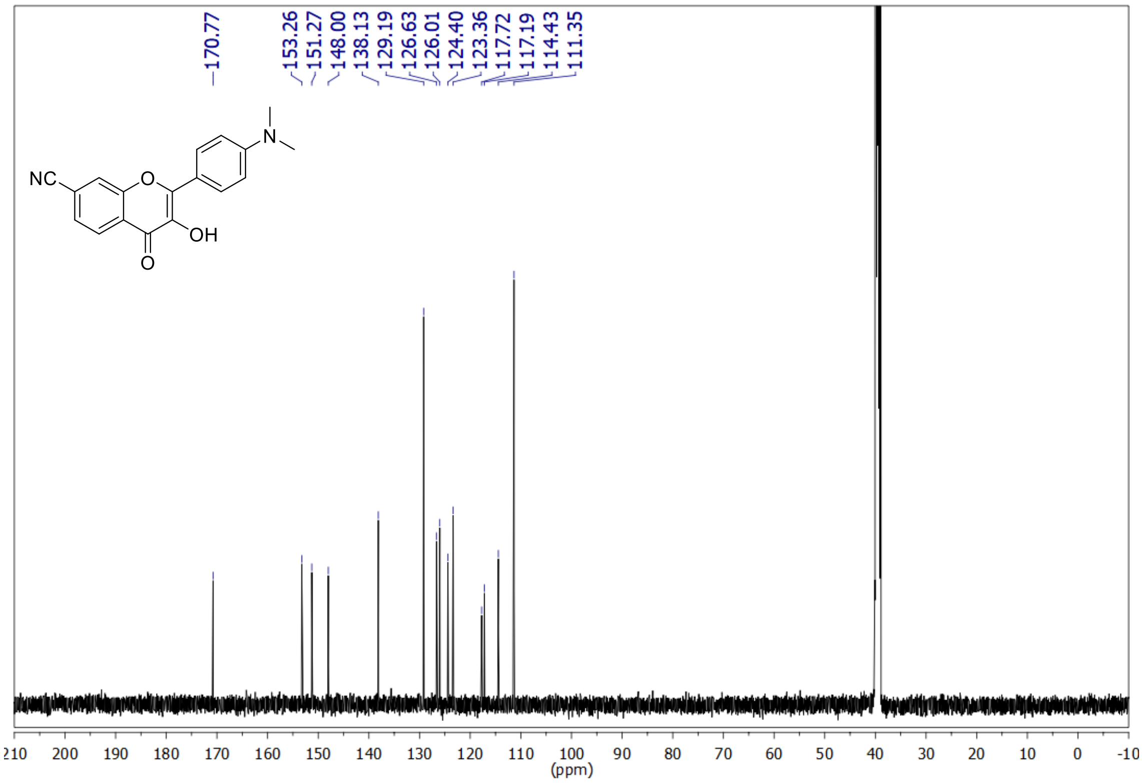

Figure S31. ^{13}C{^{1}H} NMR (125 MHz, DMSO-d_6): **11**.

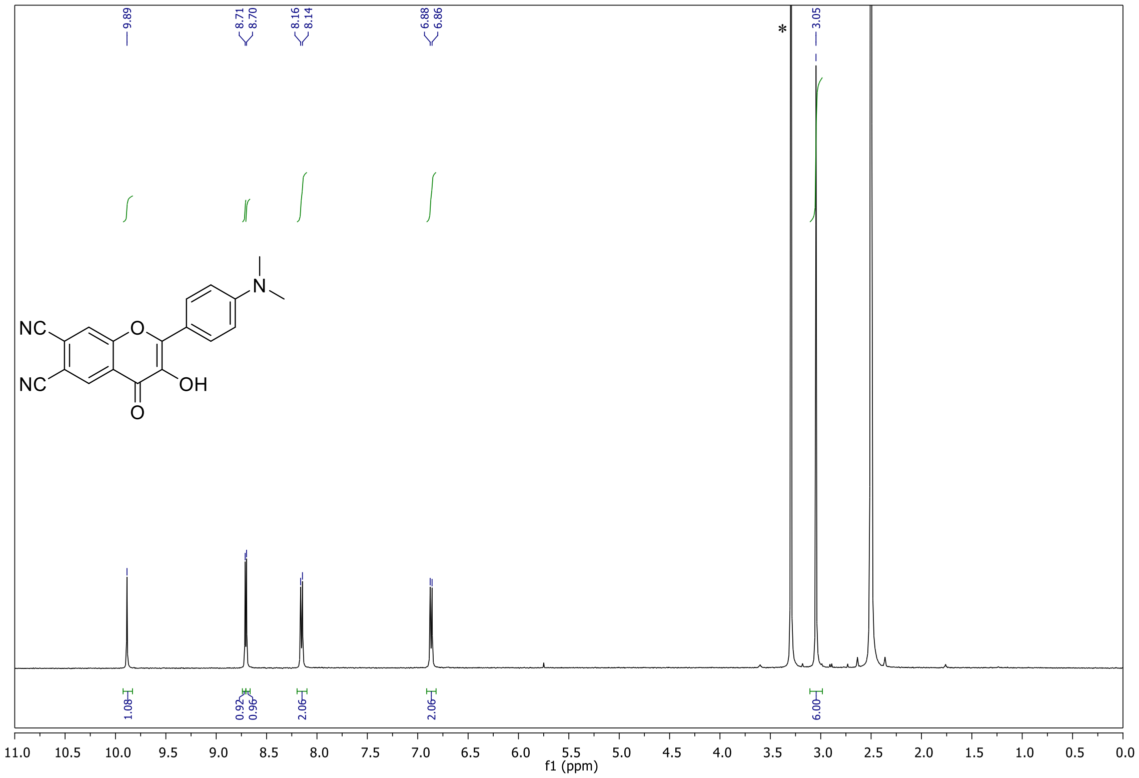

Figure S32. ^{1}H NMR (500 MHz, DMSO-d_6): **12**. Residual signal of water is marked with an asterisk.

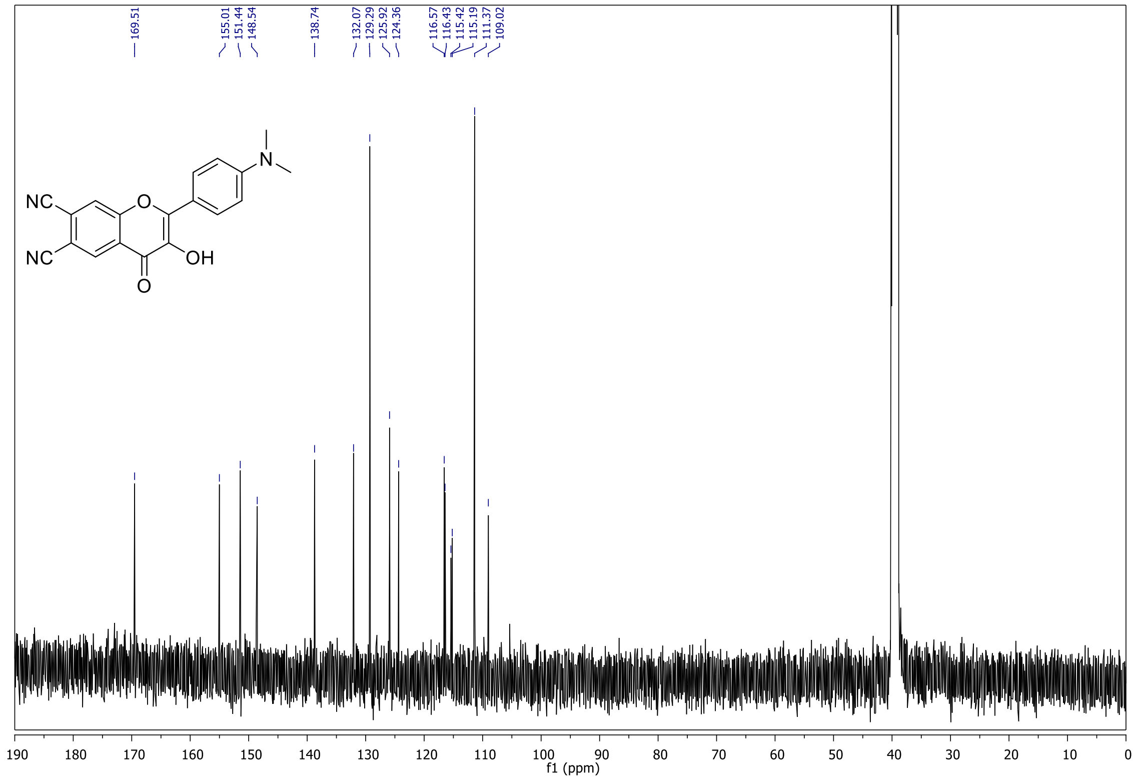

Figure S33. $^{13}C\{^{1}H\}$ NMR (125 MHz, DMSO-d_6): **12**.

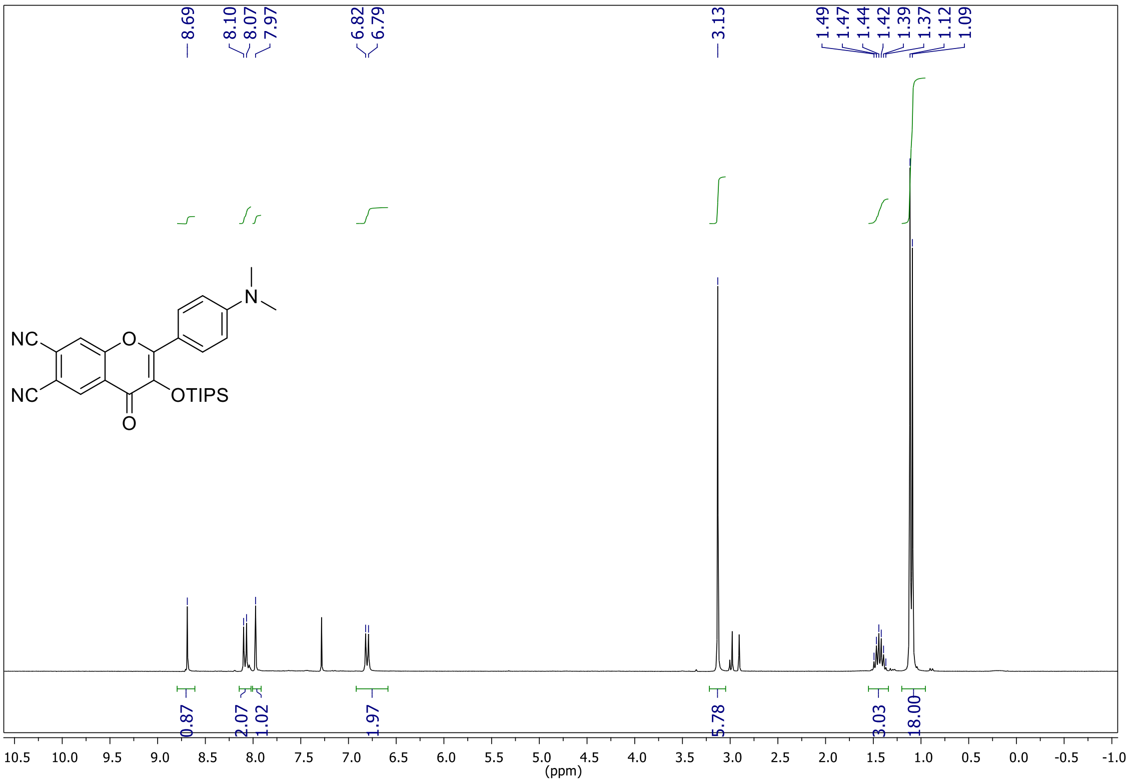

Figure S34. ^{1}H NMR (500 MHz, $CDCl_3$): **12-TIPS**.

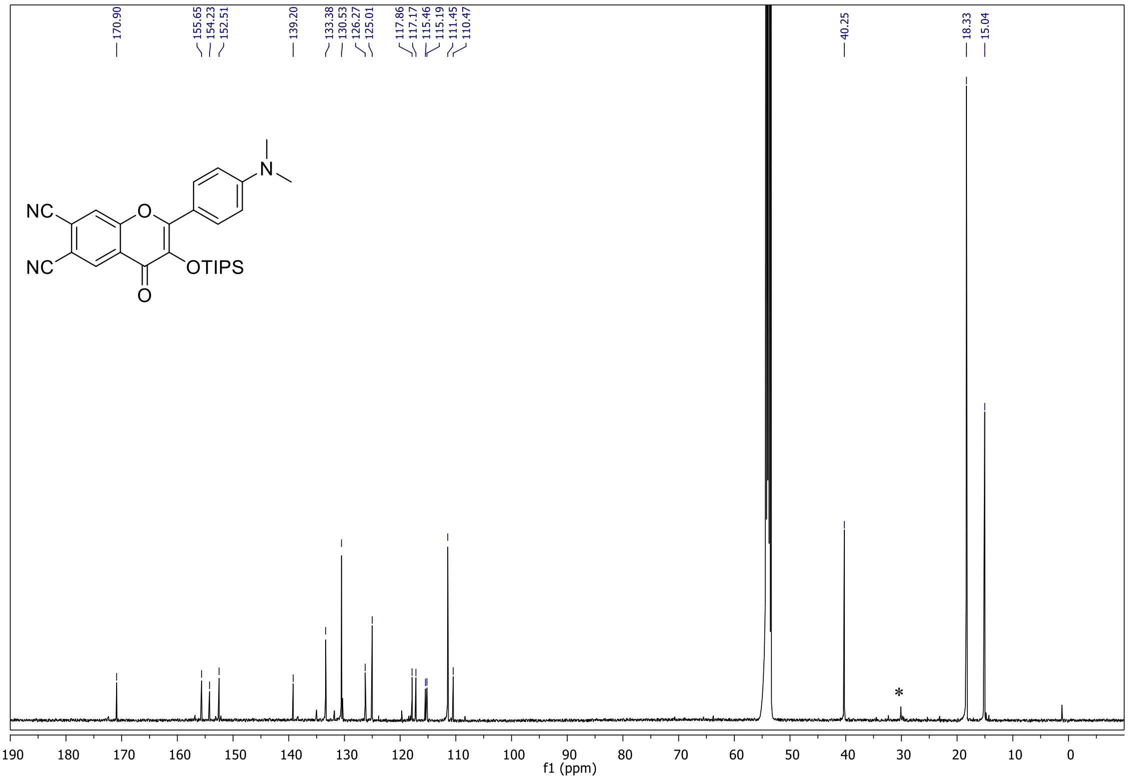

Figure S35. $^{13}C\{^{1}H\}$ NMR (125 MHz, CD_2Cl_2): **12-TIPS**. Asterisk denotes residual signal of *n*-hexane.

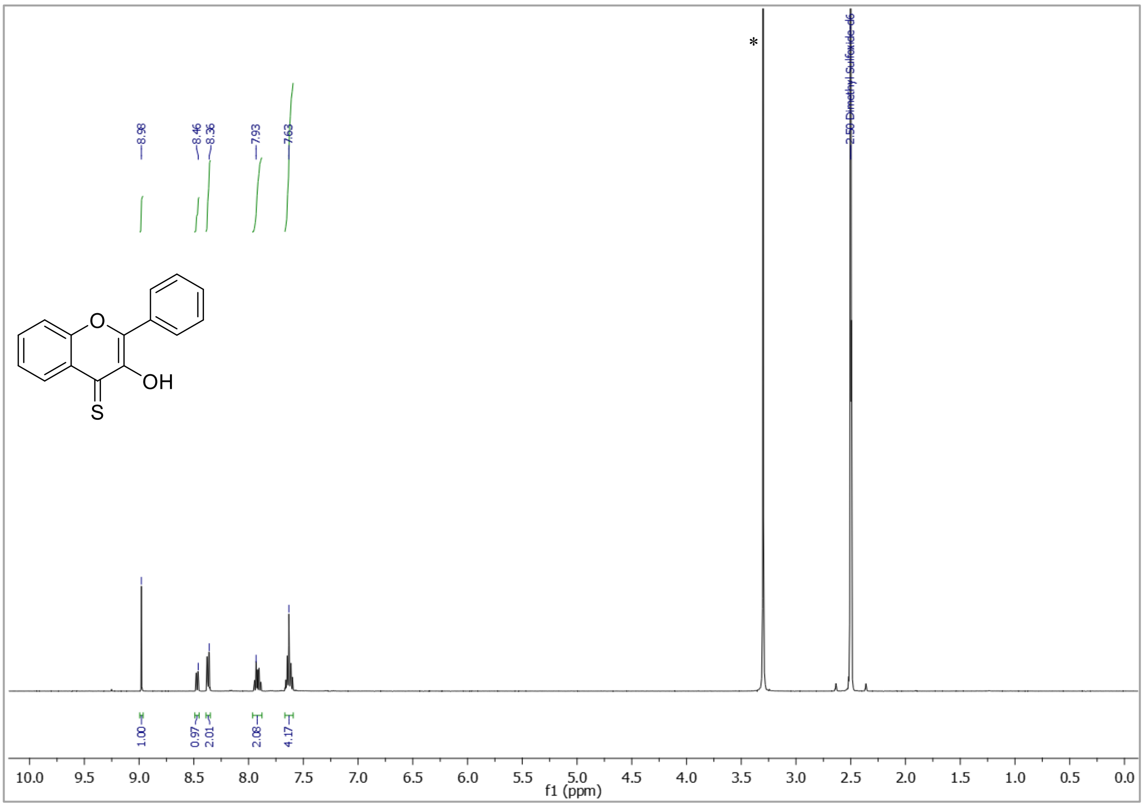

Figure S36. ^{1}H NMR (500 MHz, DMSO-d_6): **13**. Asterisk denotes residual signal of water.

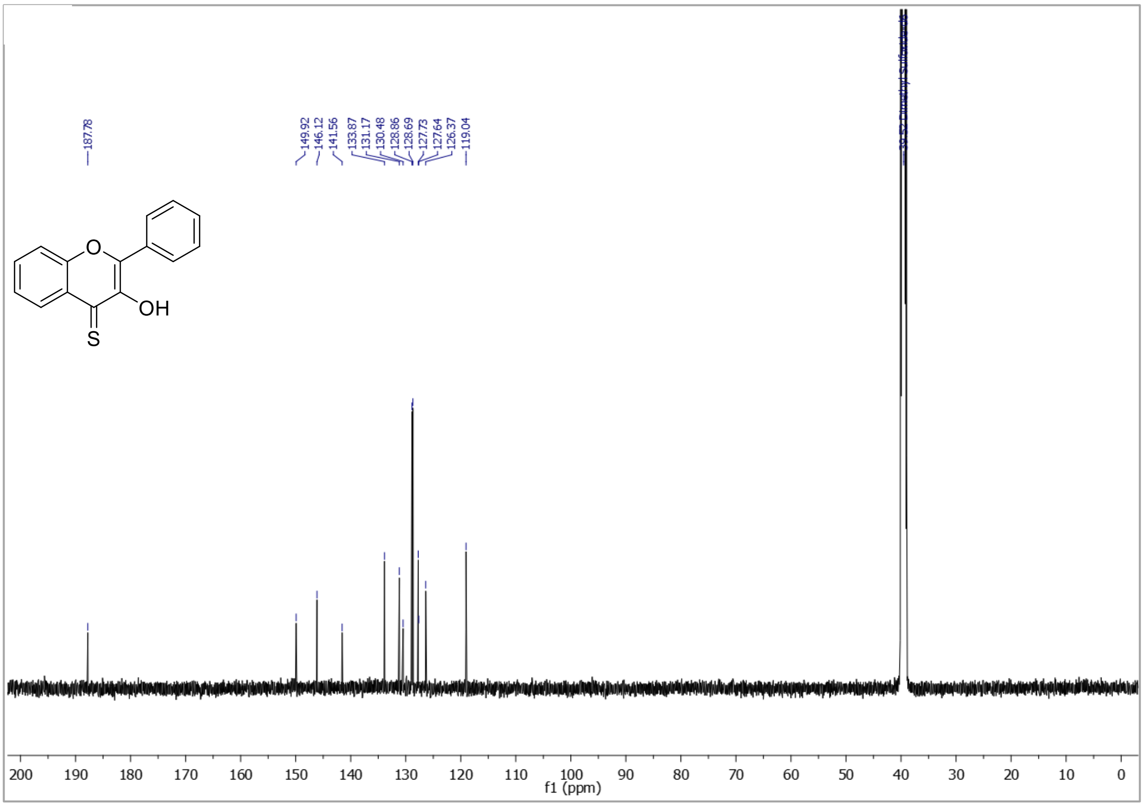

Figure S37. $^{13}C\{^{1}H\}$ NMR (125 MHz, DMSO-d_6): **13**.

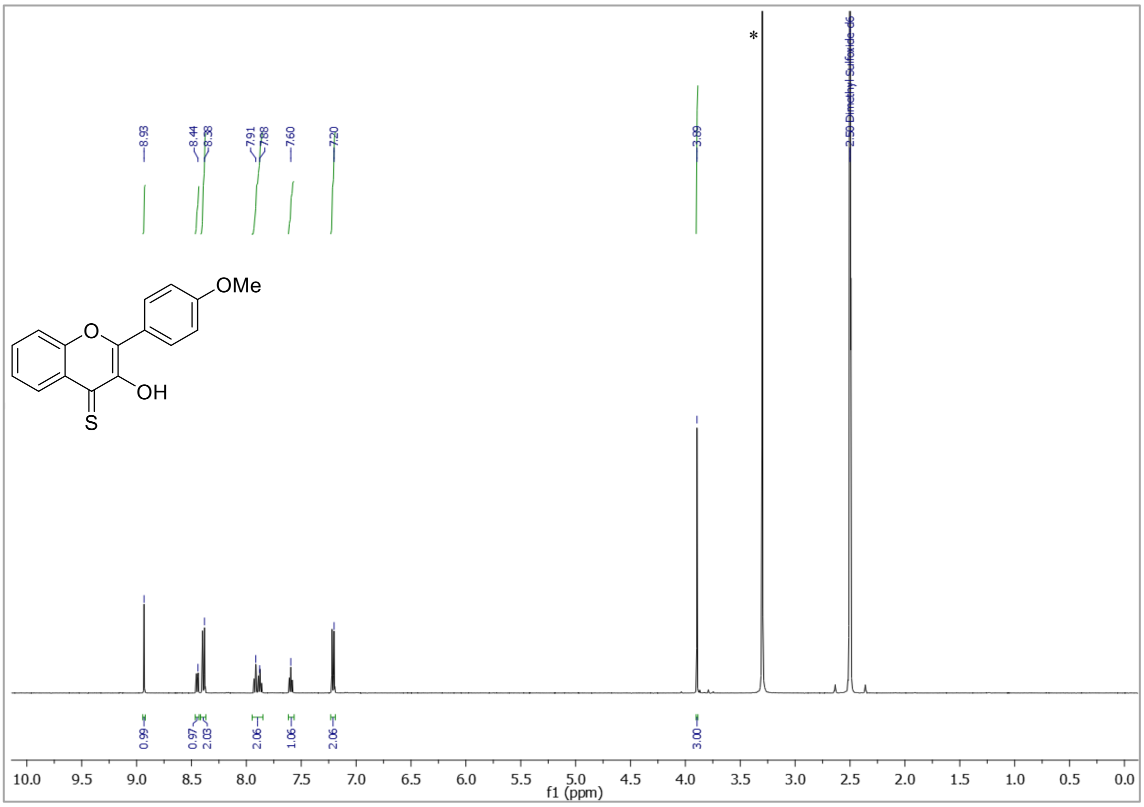

Figure S38. ^{1}H NMR (500 MHz, DMSO-d_6): **14**. Asterisk denotes residual signal of water.

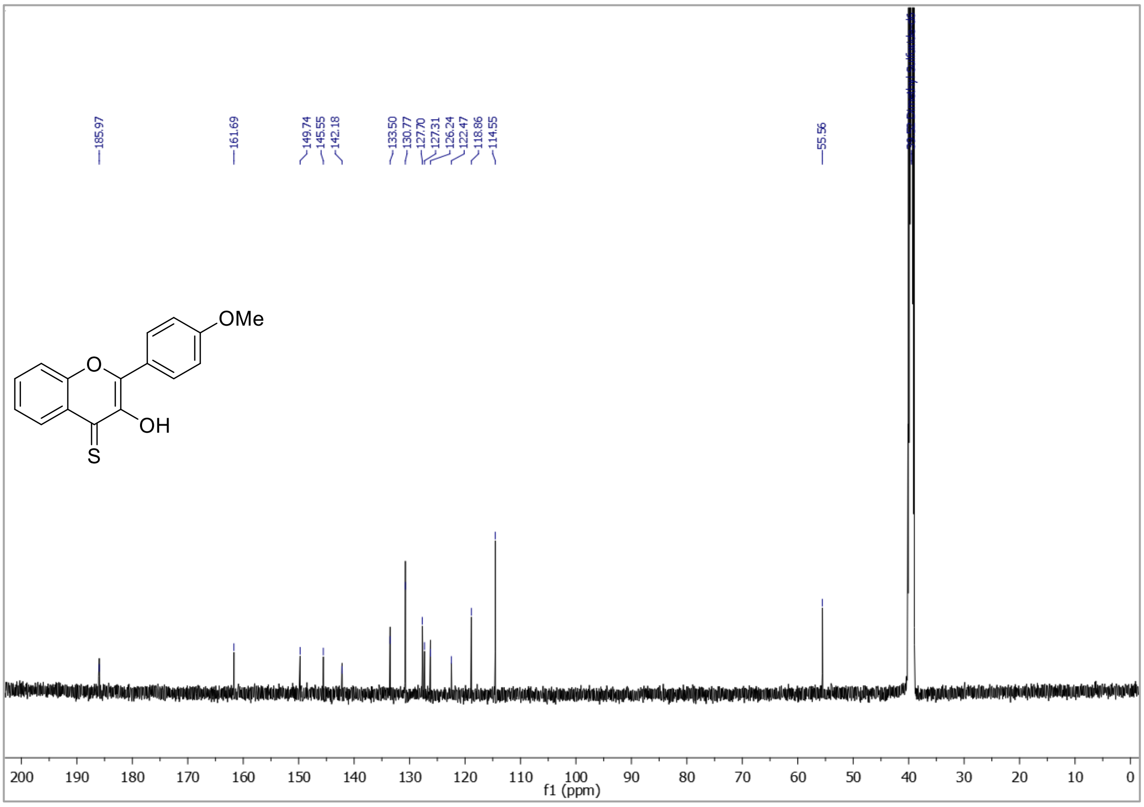

Figure S39. $^{13}C\{^{1}H\}$ NMR (125 MHz, DMSO-d_6): **14**.

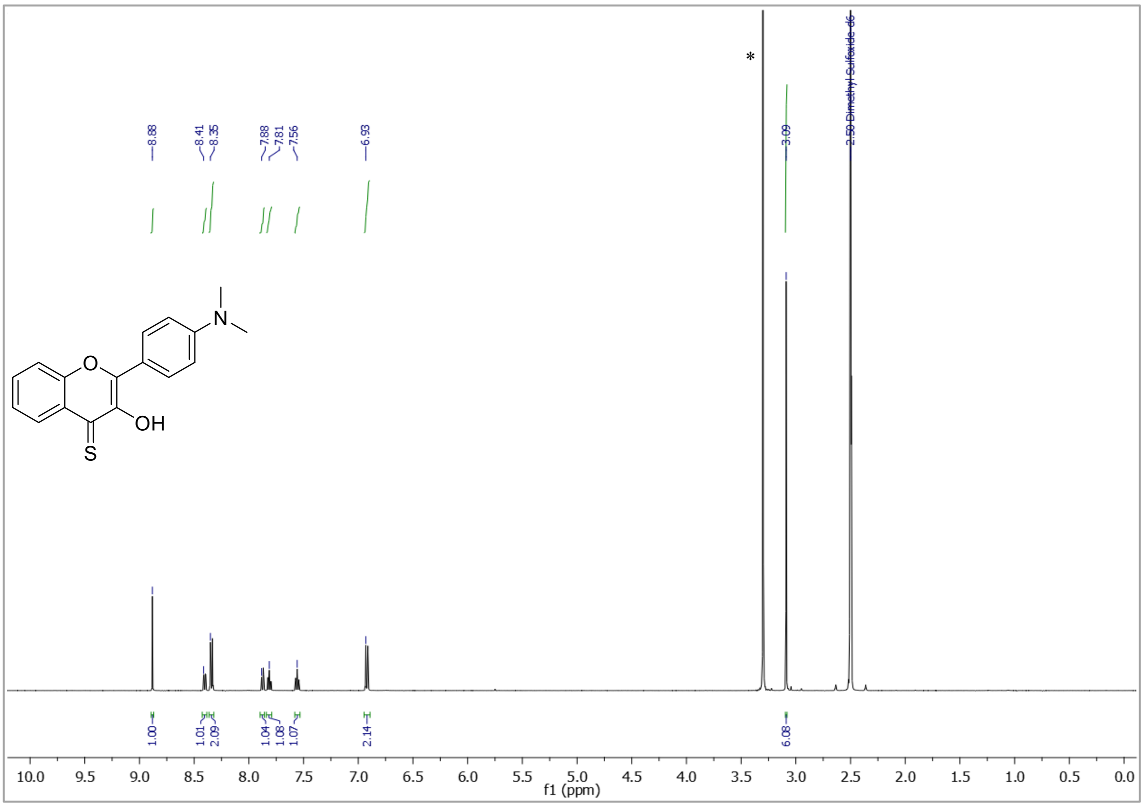

Figure S40. ^{1}H NMR (500 MHz, DMSO-d_6): **15**. Residual signal of water is marked with an asterisk.

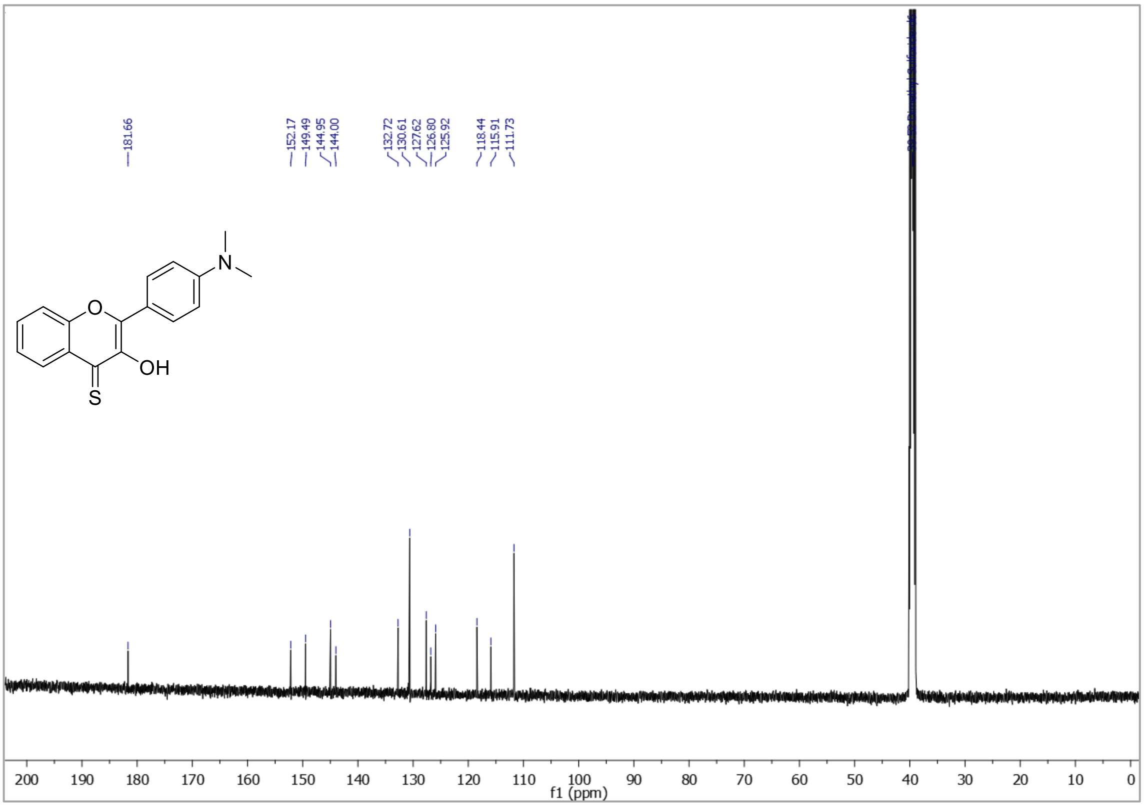

Figure S41. $^{13}C\{^{1}H\}$ NMR (125 MHz, DMSO-d_6): **15**.

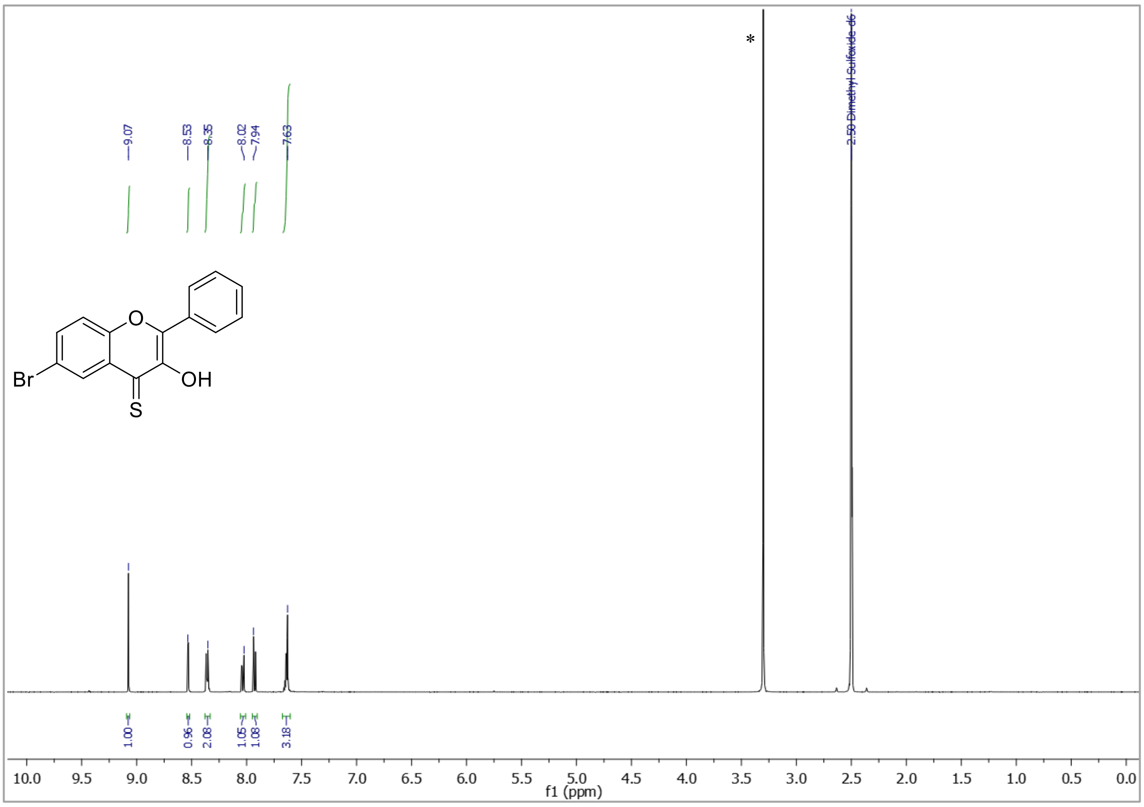

Figure S42. ^{1}H NMR (500 MHz, DMSO-d_6): **16**. Residual signal of water is marked with an asterisk.

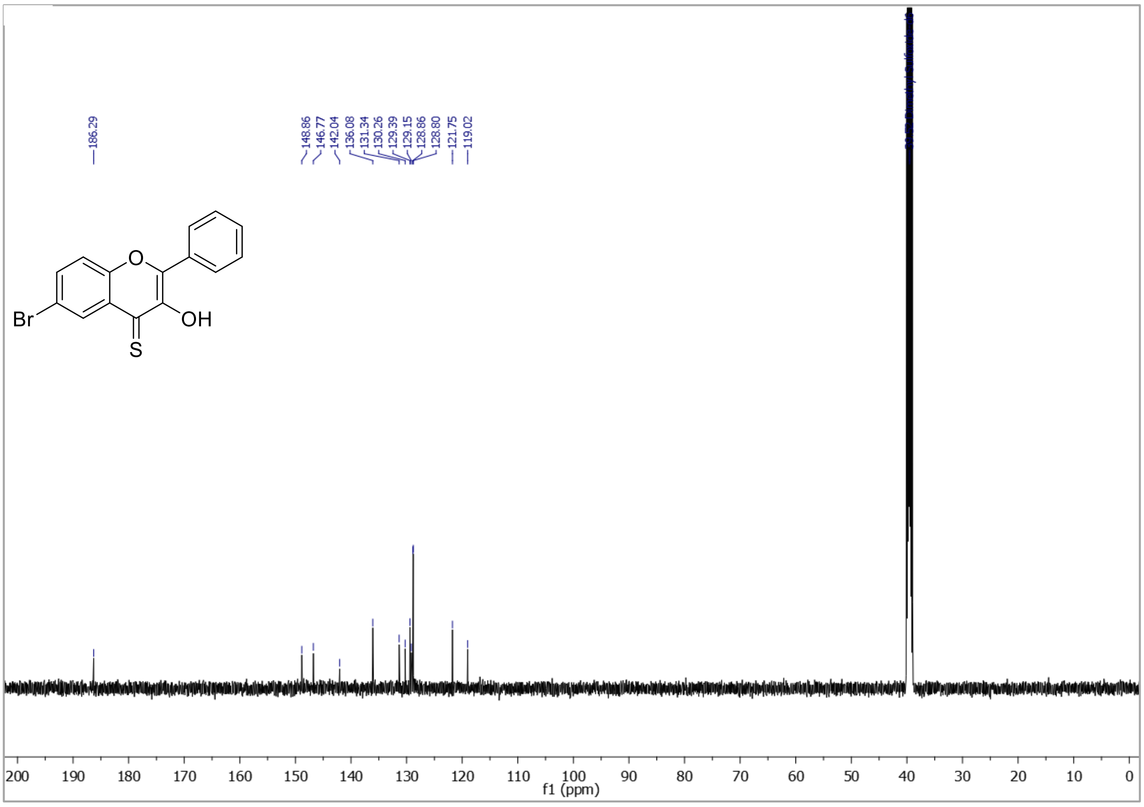

Figure S43. $^{13}C\{^{1}H\}$ NMR (125 MHz, DMSO-d_6): **16**.

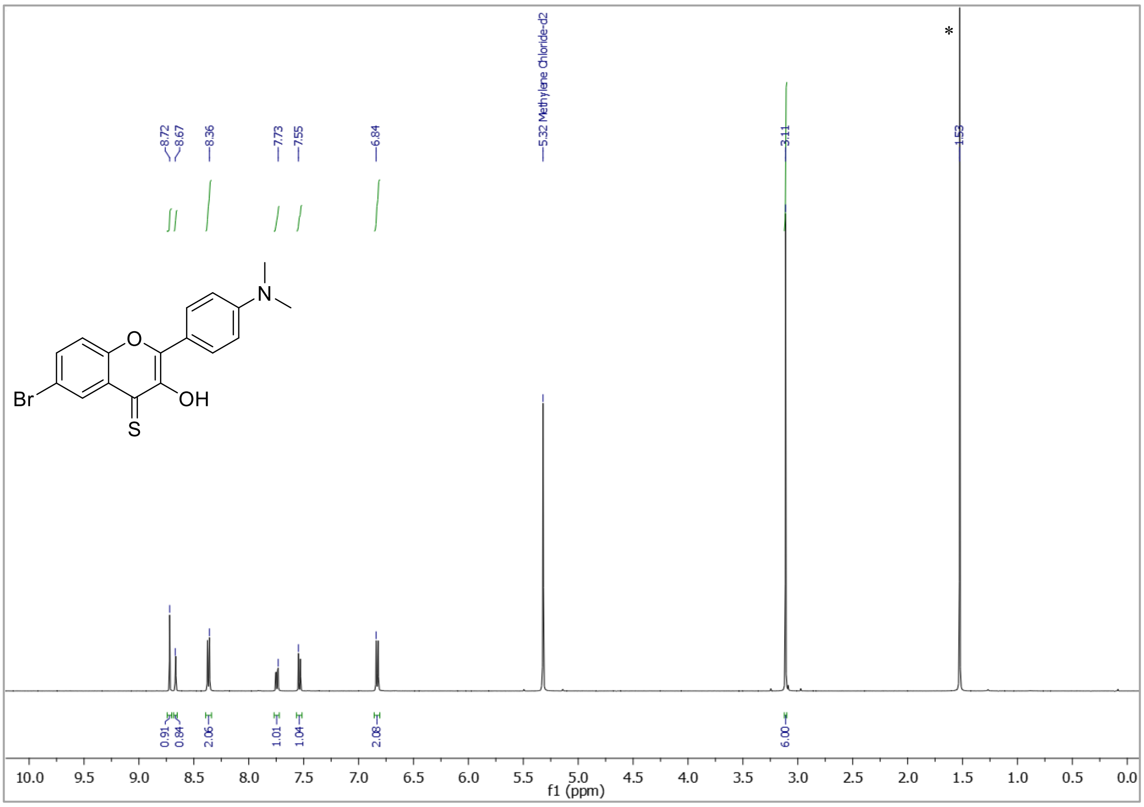

Figure S44. ^{1}H NMR (500 MHz, CD_2Cl_2): **17**. Residual signal of water is marked with an asterisk.

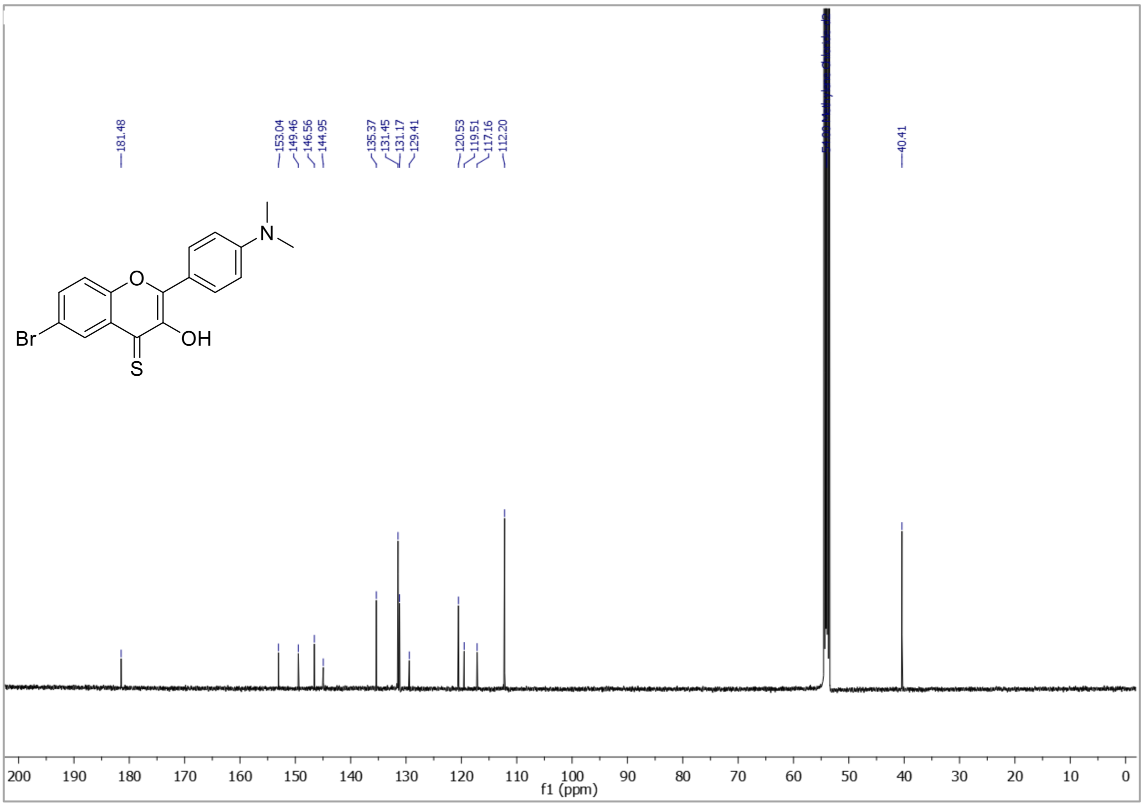

Figure S45. $^{13}C\{^{1}H\}$ NMR (125 MHz, CD_2Cl_2): **17**.

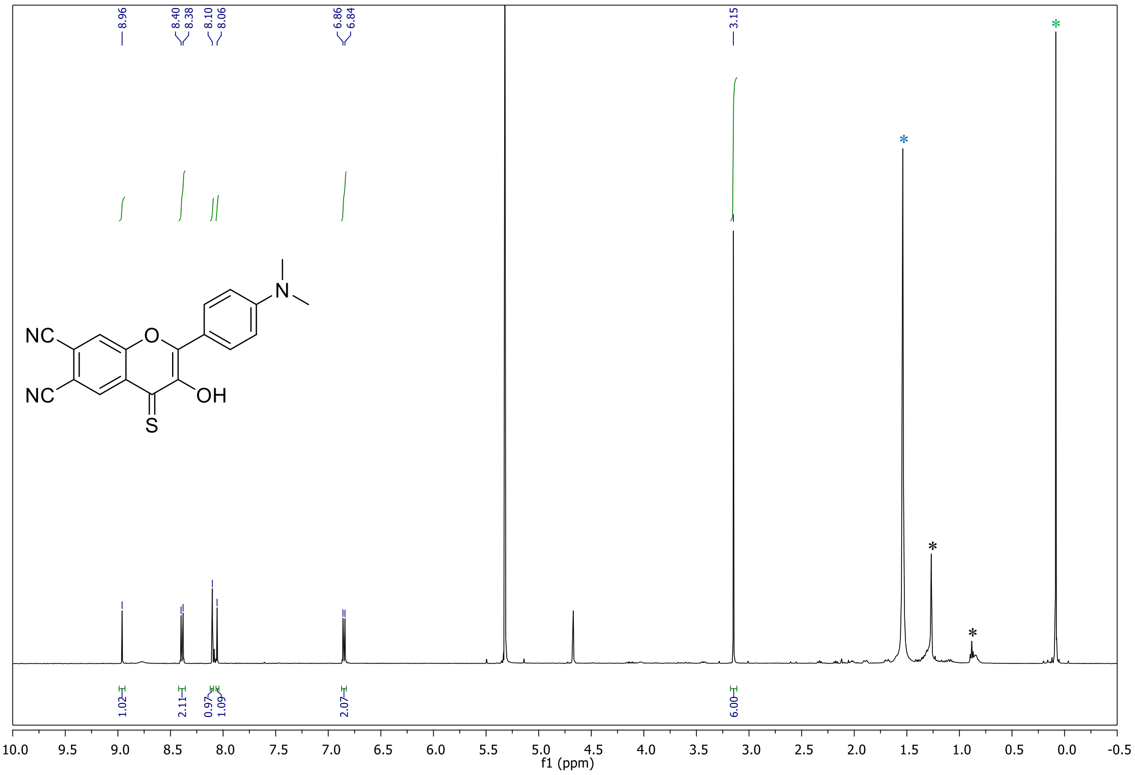

Figure S46. ^{1}H NMR (500 MHz, CD_2Cl_2): **18**. Residual signals of *n*-hexane (black), water (blue) and grease (green) are marked with asterisks.

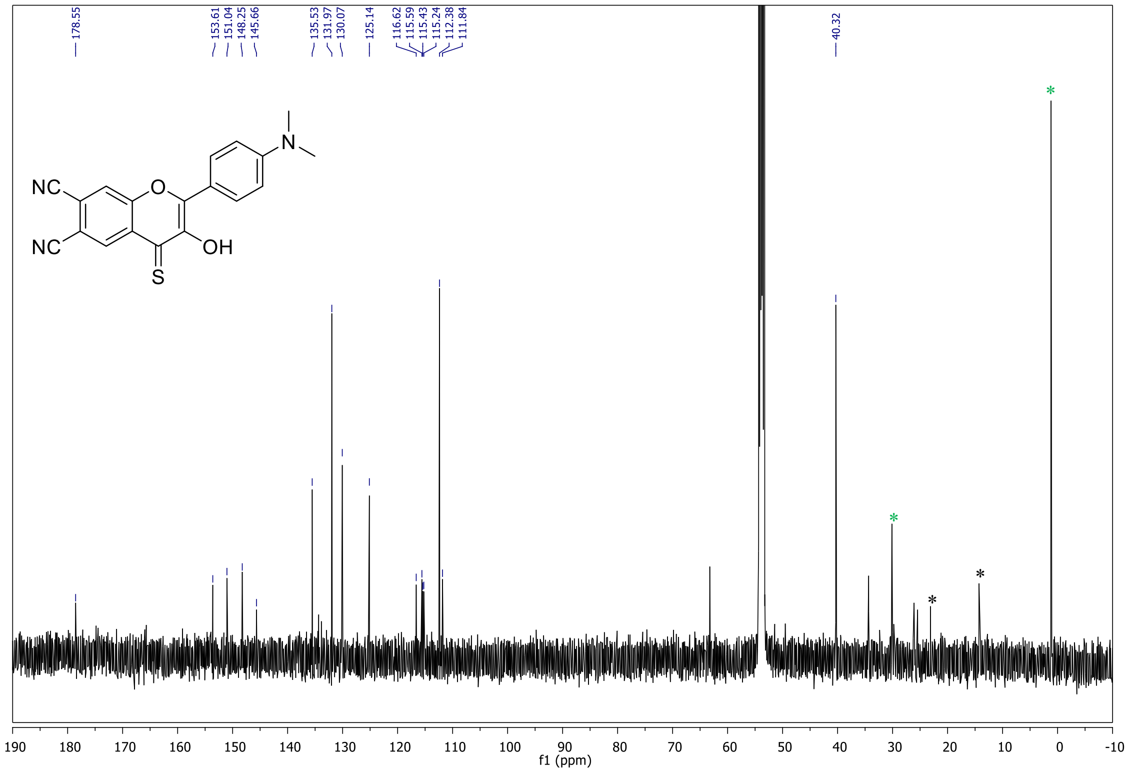

Figure S47. $^{13}C\{^{1}H\}$ NMR (125 MHz, CD_2Cl_2): **18**. Residual signals of *n*-hexane (black) and grease (green) are marked with asterisks.

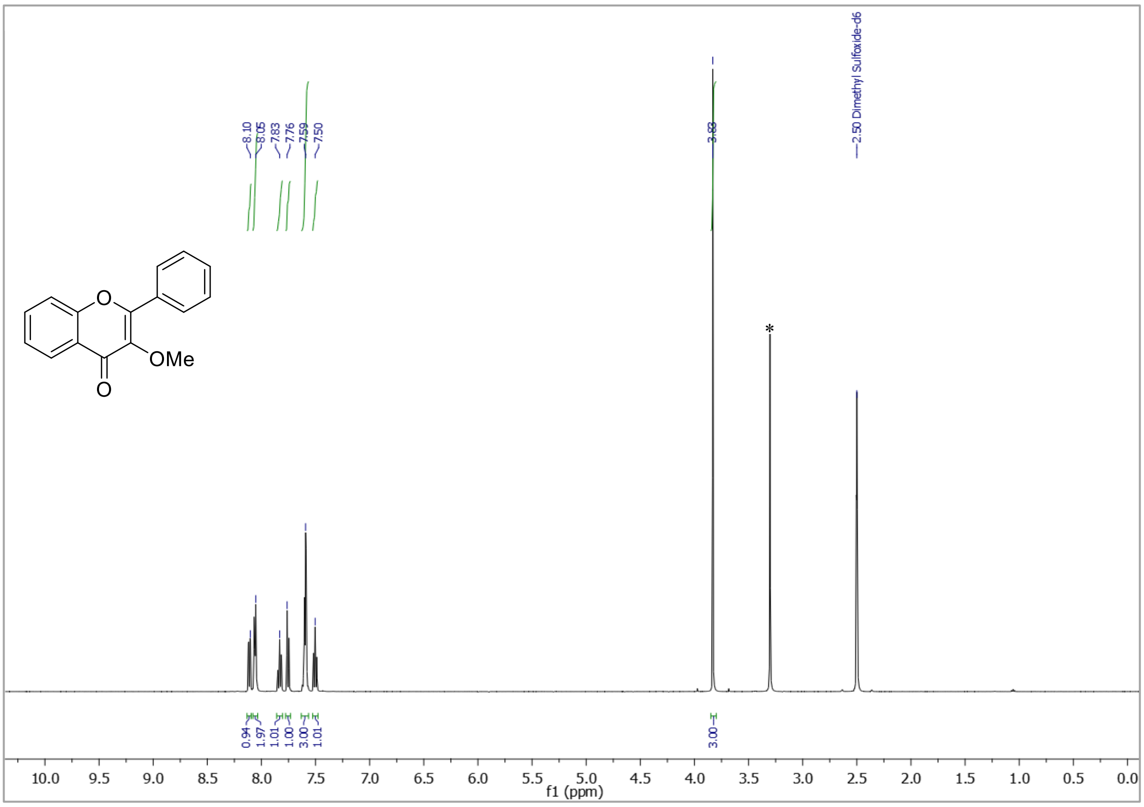

Figure S48. ^{1}H NMR (500 MHz, DMSO-d_6): **19**. Residual signal of water is marked with an asterisk.

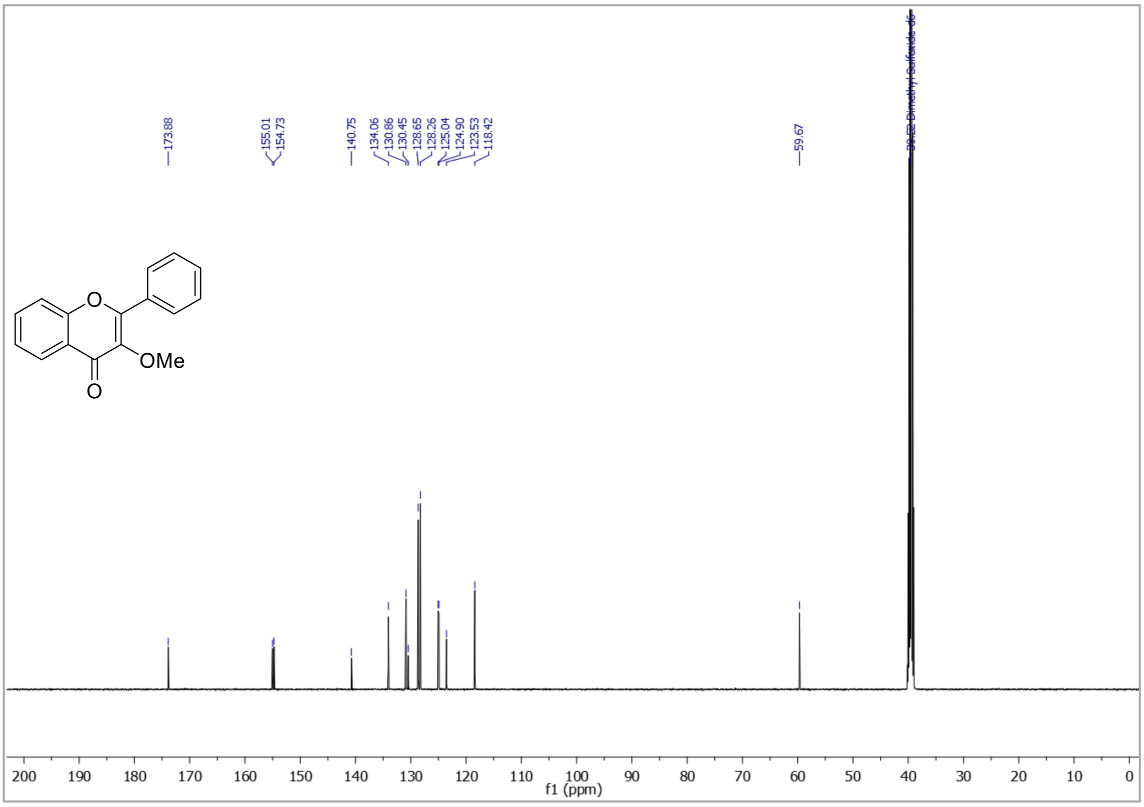

Figure S49. $^{13}C\{^{1}H\}$ NMR (125 MHz, DMSO-d_6): **19**.

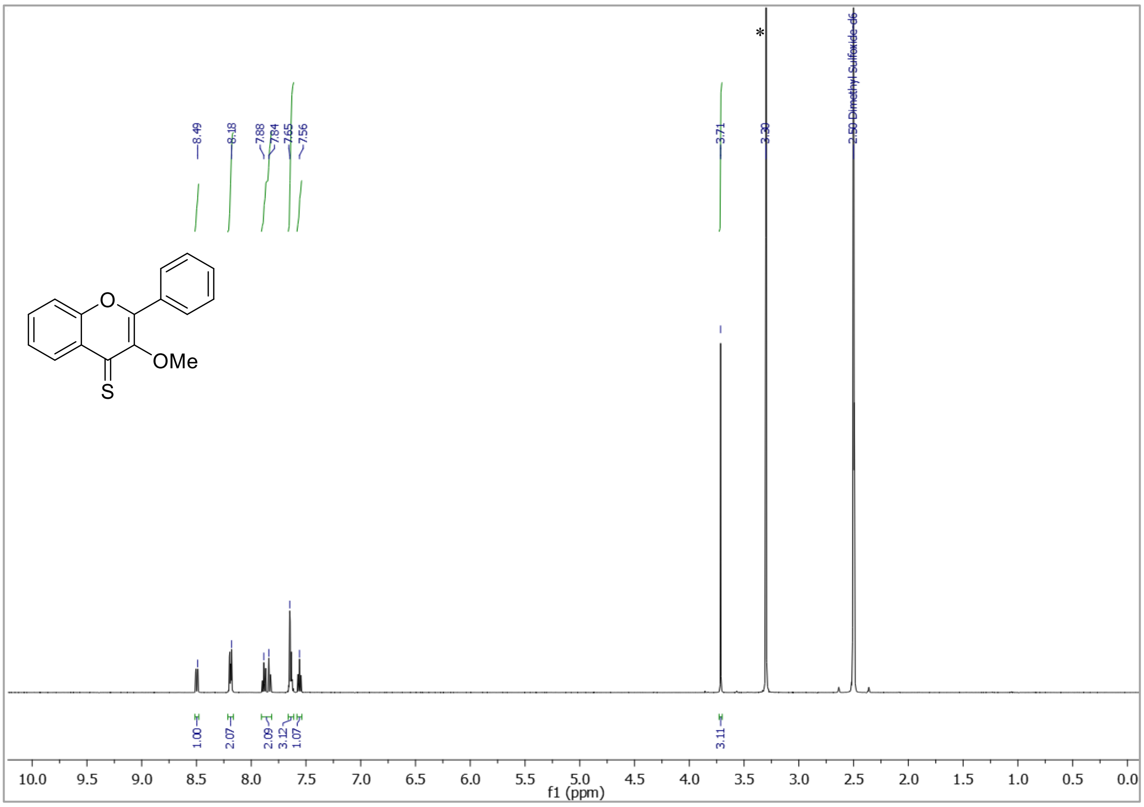

Figure S50. ^{1}H NMR (500 MHz, DMSO-d_6): **20**. Residual signal of water is marked with an asterisk.

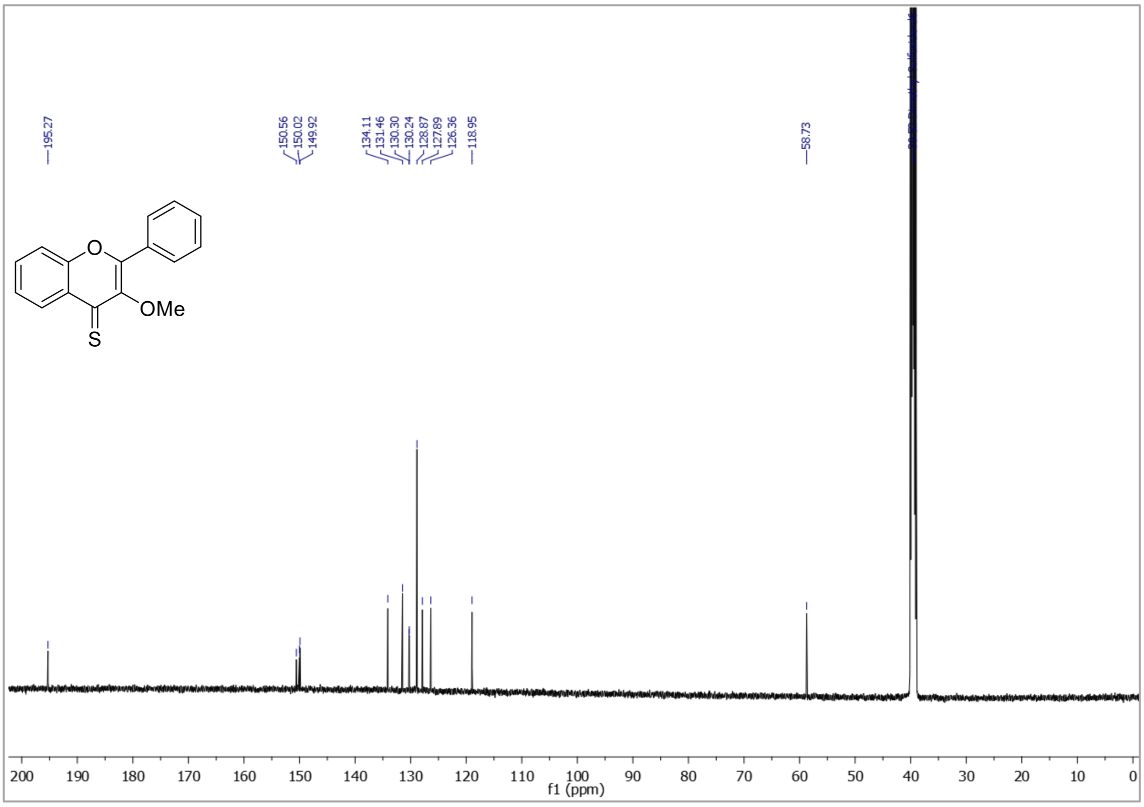

Figure S51. ^{13}C{^{1}H} NMR (125 MHz, DMSO-d_6): **20**.

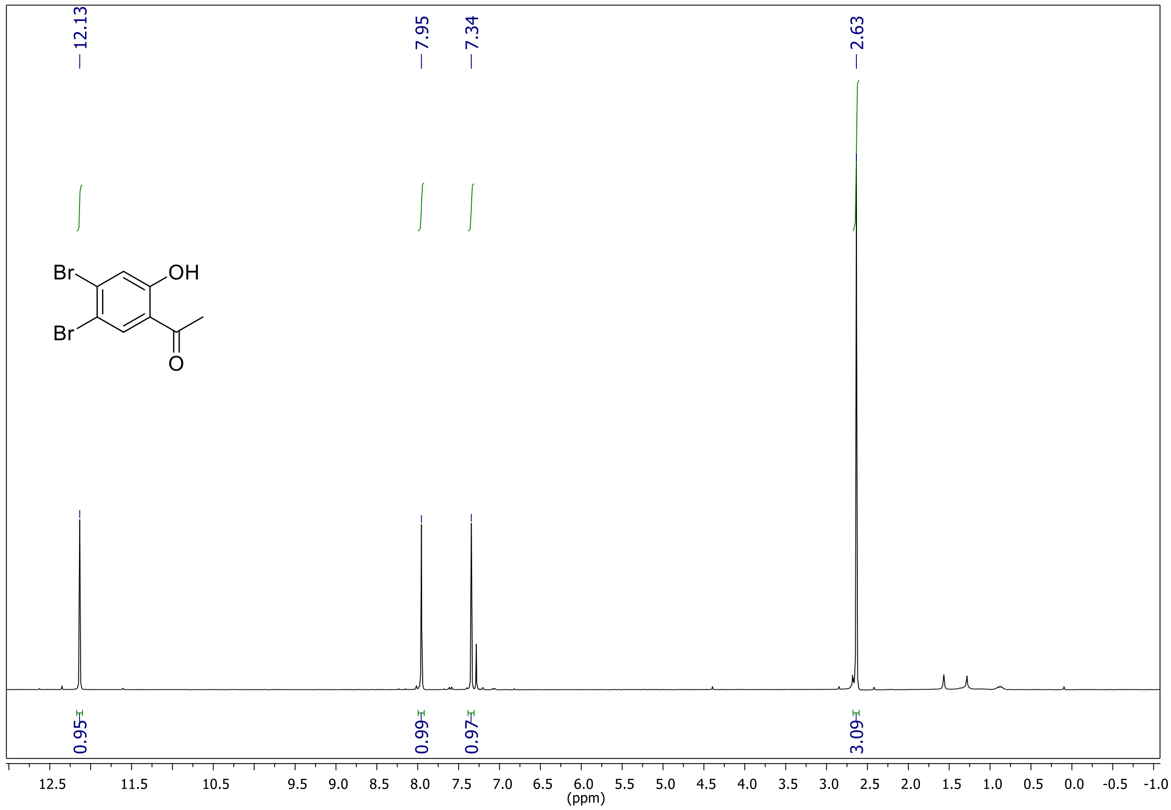

Figure S52. ^{1}H NMR (500 MHz, $CDCl_3$): **21d**.

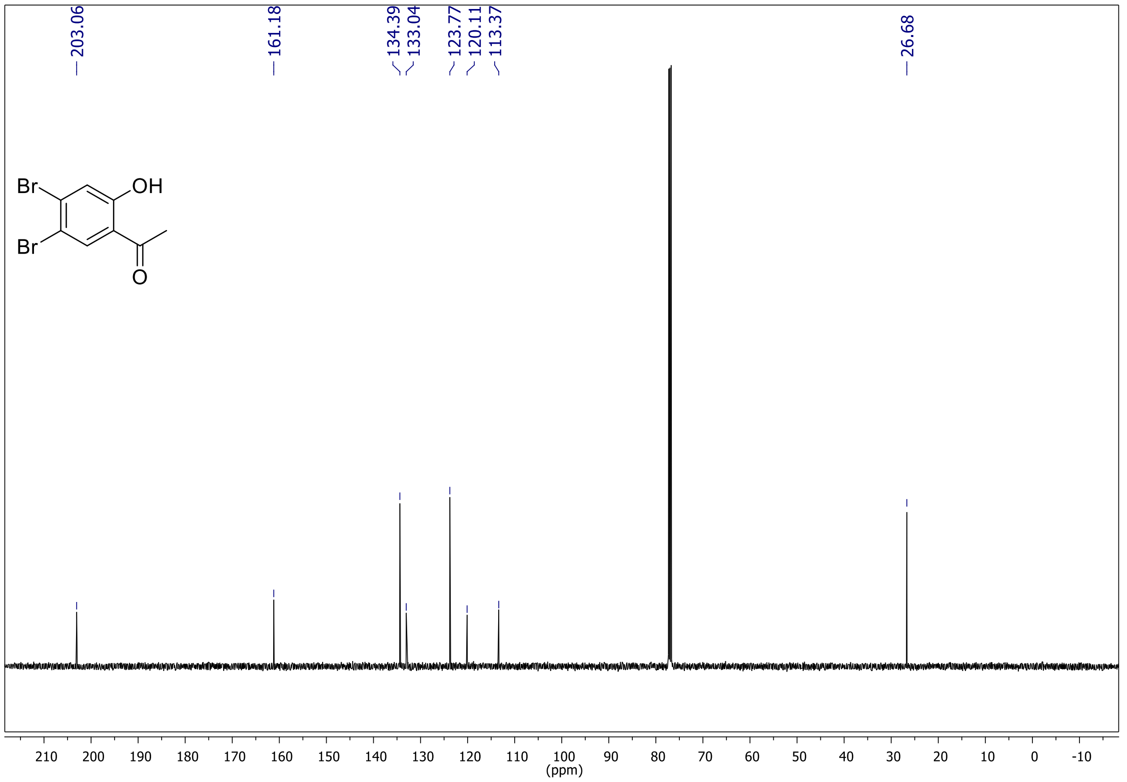

Figure S53. $^{13}C\{^{1}H\}$ NMR (125 MHz, $CDCl_3$): **21d**.

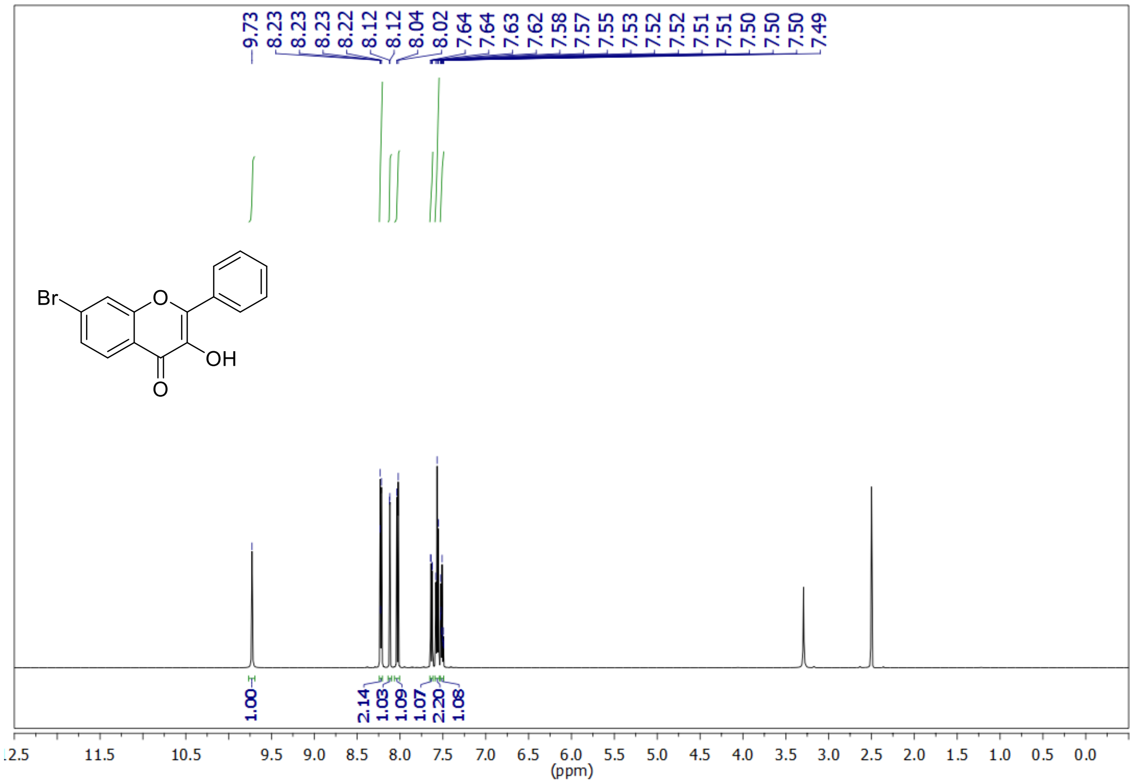

Figure S54. ^{1}H NMR (500 MHz, DMSO-d_6): **24**. Asterisk denotes residual signal of water.

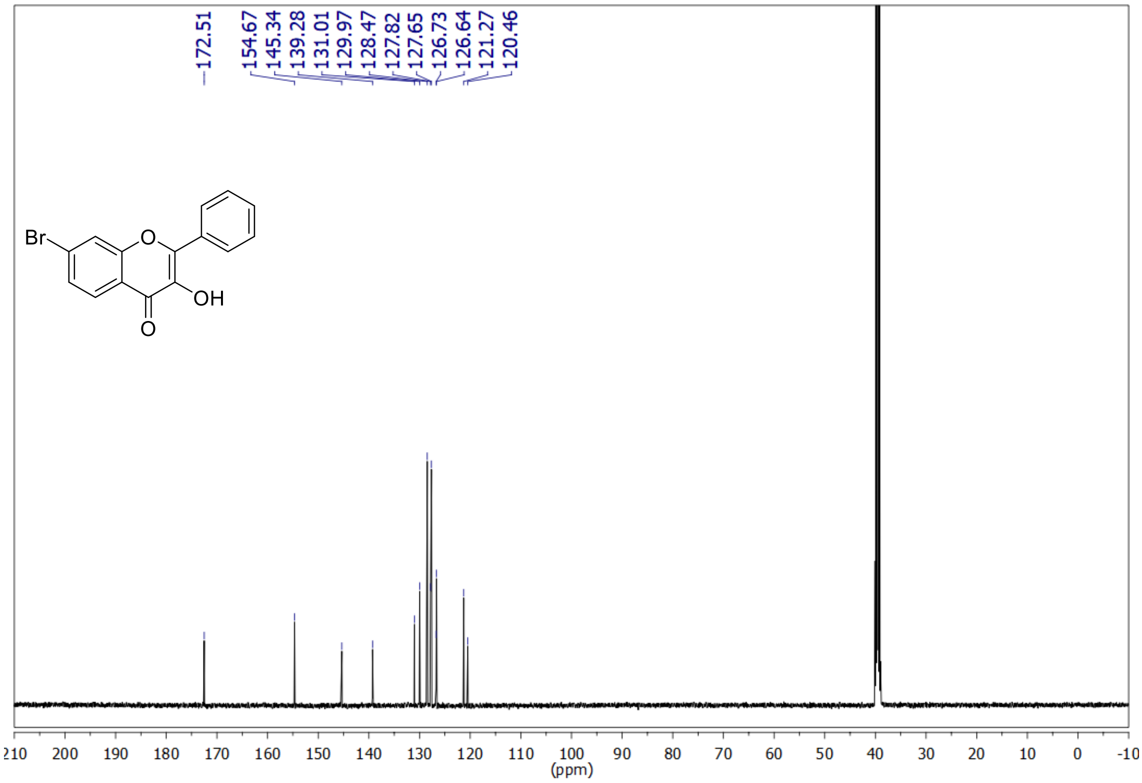

Figure S55. $^{13}C\{^{1}H\}$ NMR (125 MHz, DMSO-d_6): **24**.

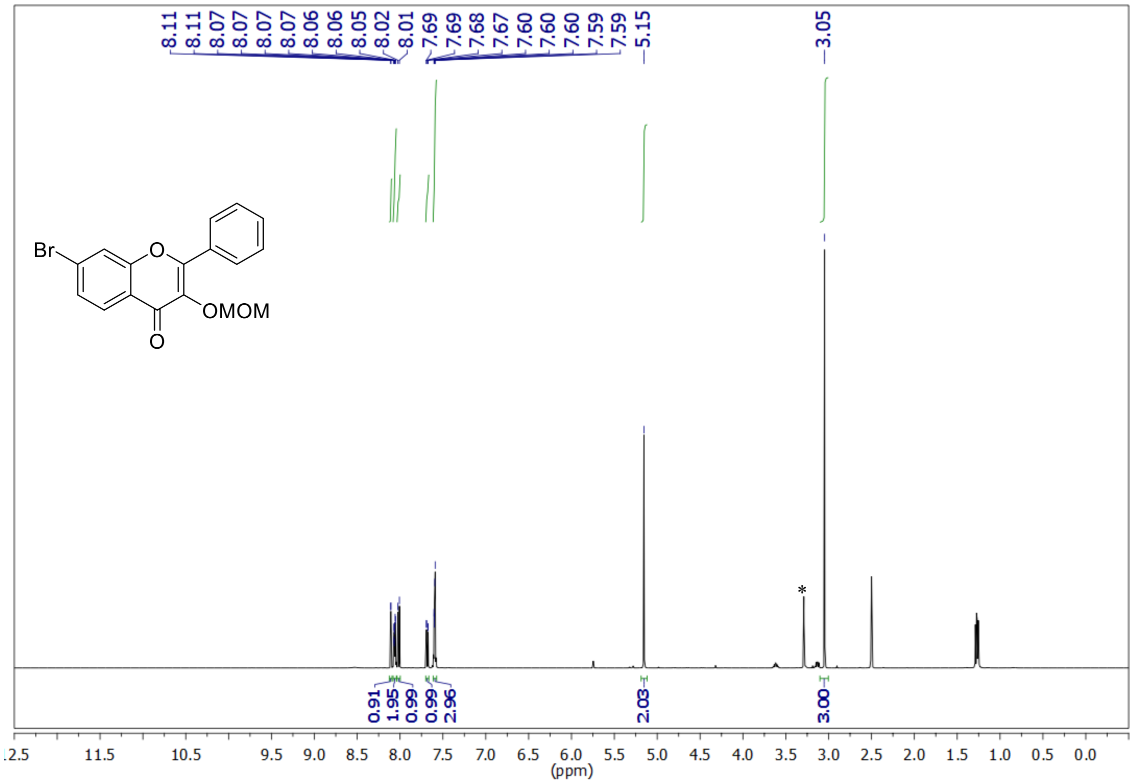

Figure S56. ^{1}H NMR (500 MHz, DMSO-d_6): **24-MOM**. The asterisk denotes the residual signal of water.

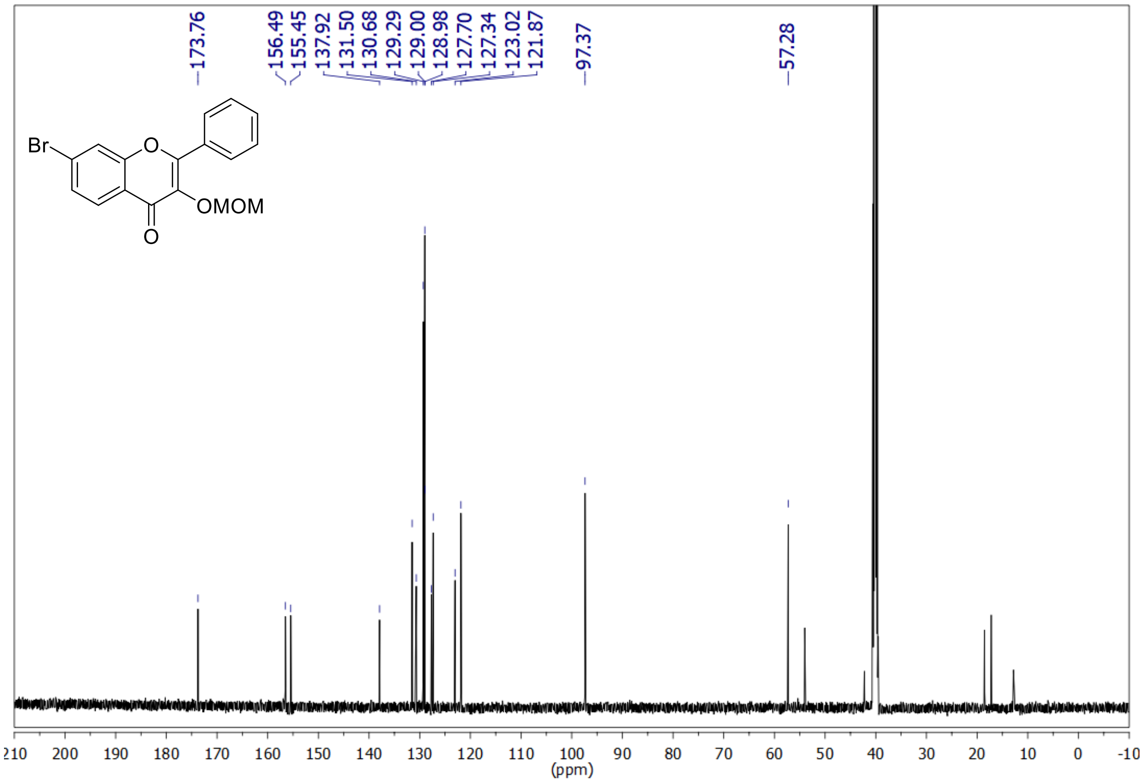

Figure S57. $^{13}C\{^{1}H\}$ NMR (125 MHz, DMSO-d_6): **24-MOM**.

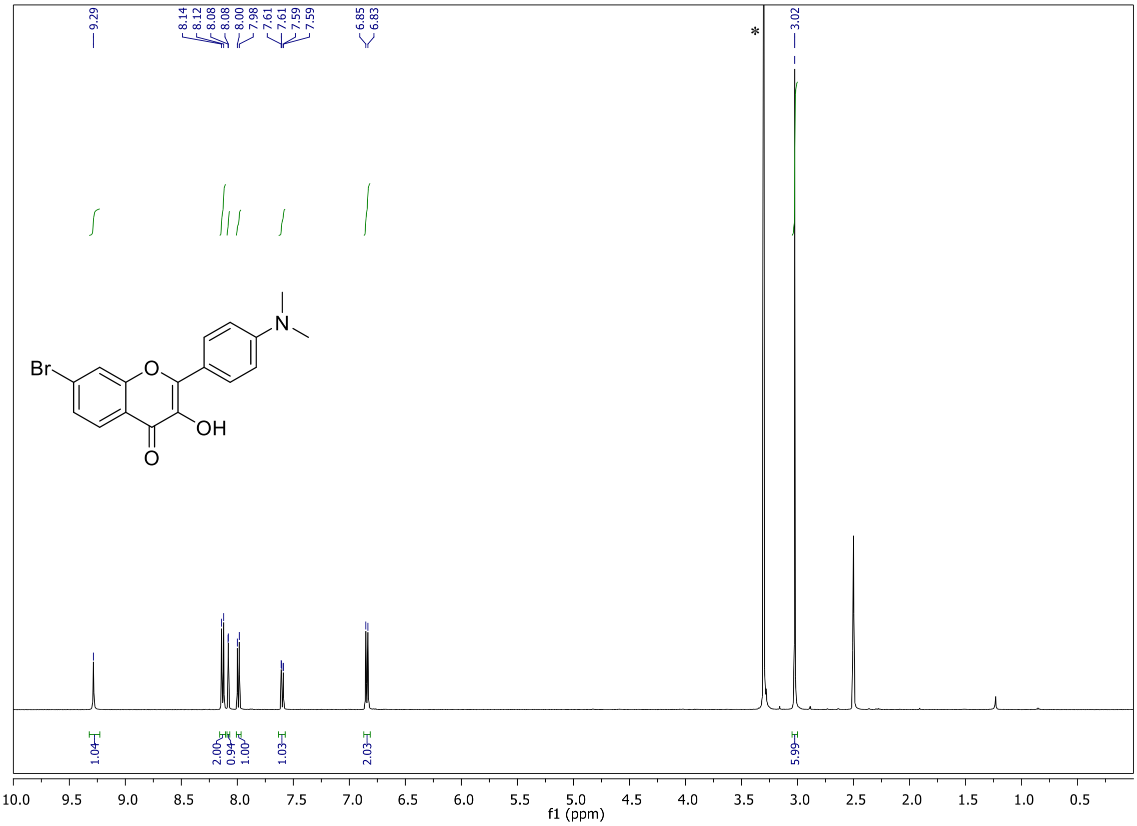

Figure S58. ^{1}H NMR (500 MHz, DMSO-d_6): **25**. Residual signal of water is marked with an asterisk.

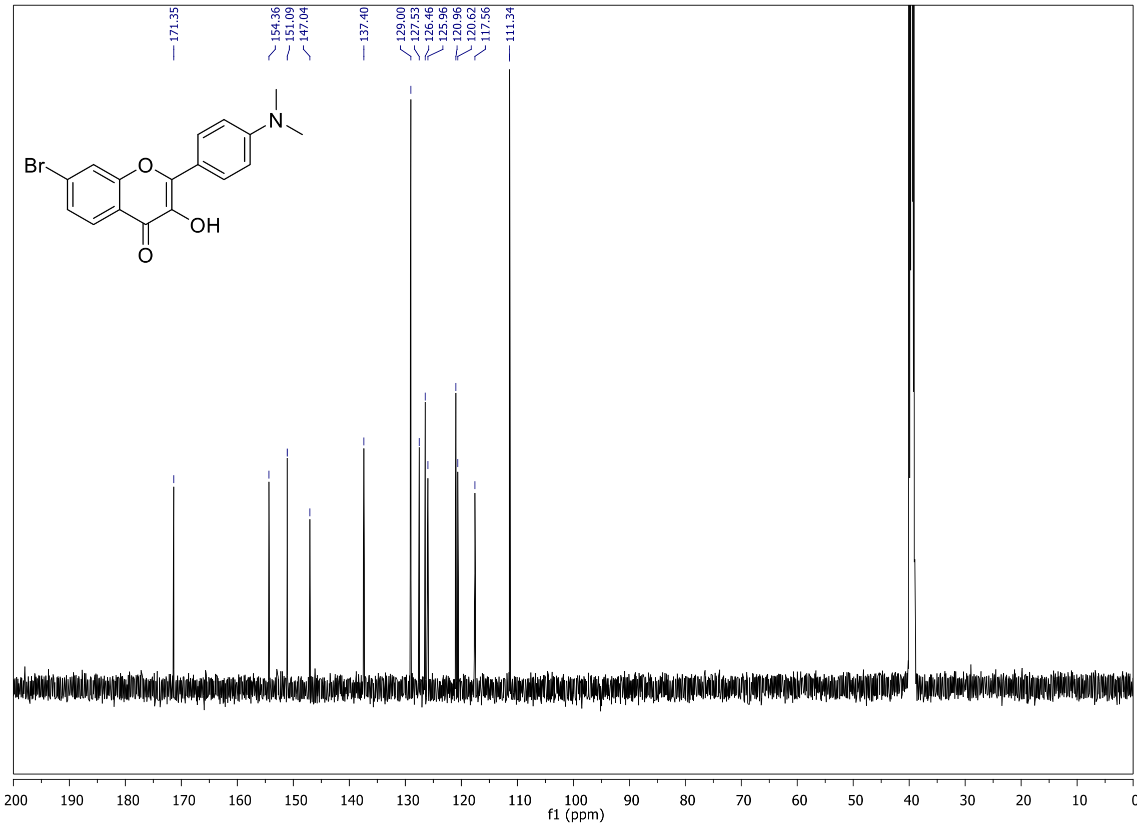

Figure S59. $^{13}C\{^{1}H\}$ NMR (125 MHz, DMSO-d_6): **25**.

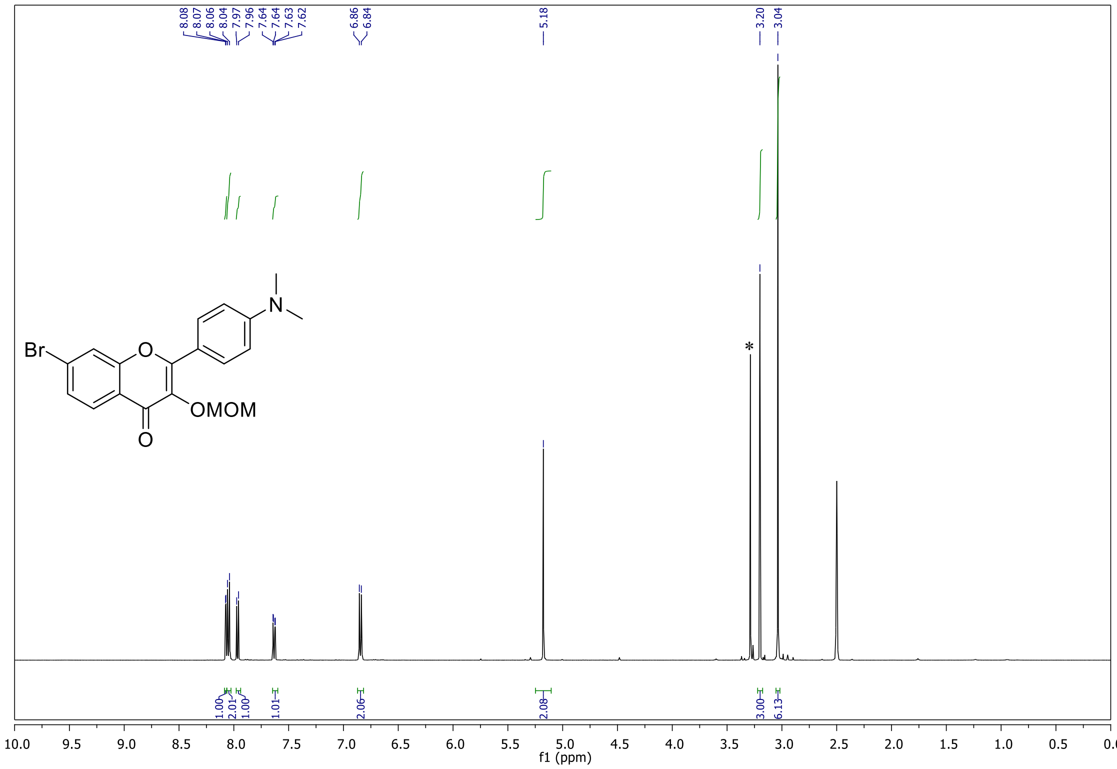

Figure S60. ^{1}H NMR (500 MHz, DMSO-d_6): **25-MOM**. Asterisk denotes the residual signal of water.

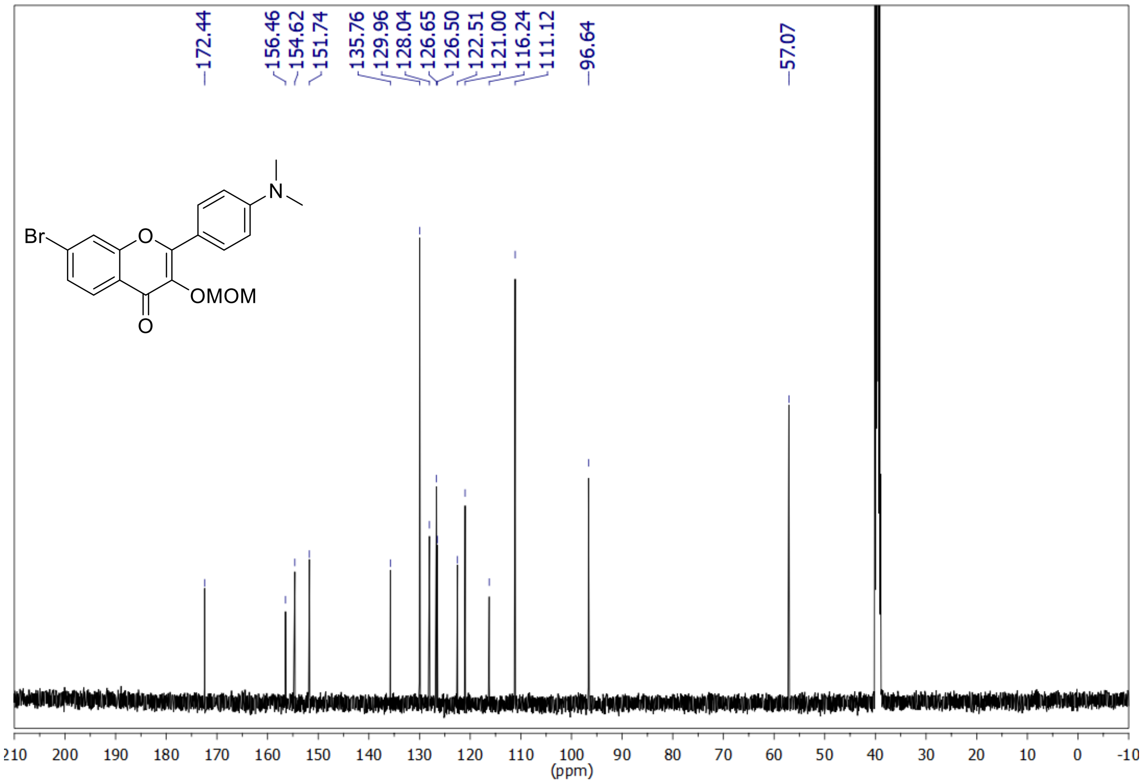

Figure S61. ^{13}C{^{1}H} NMR (125 MHz, DMSO-d_6): **25-MOM**.

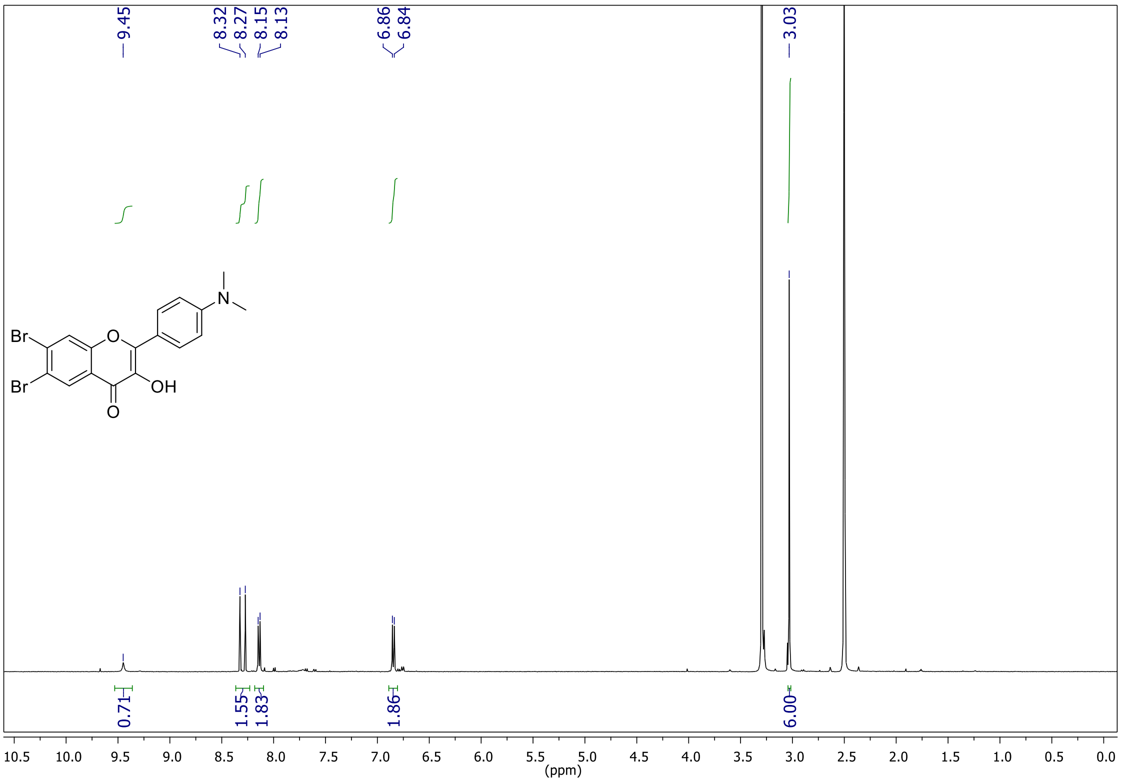

Figure S62. ^{1}H NMR (500 MHz, DMSO-d_6): **26**.

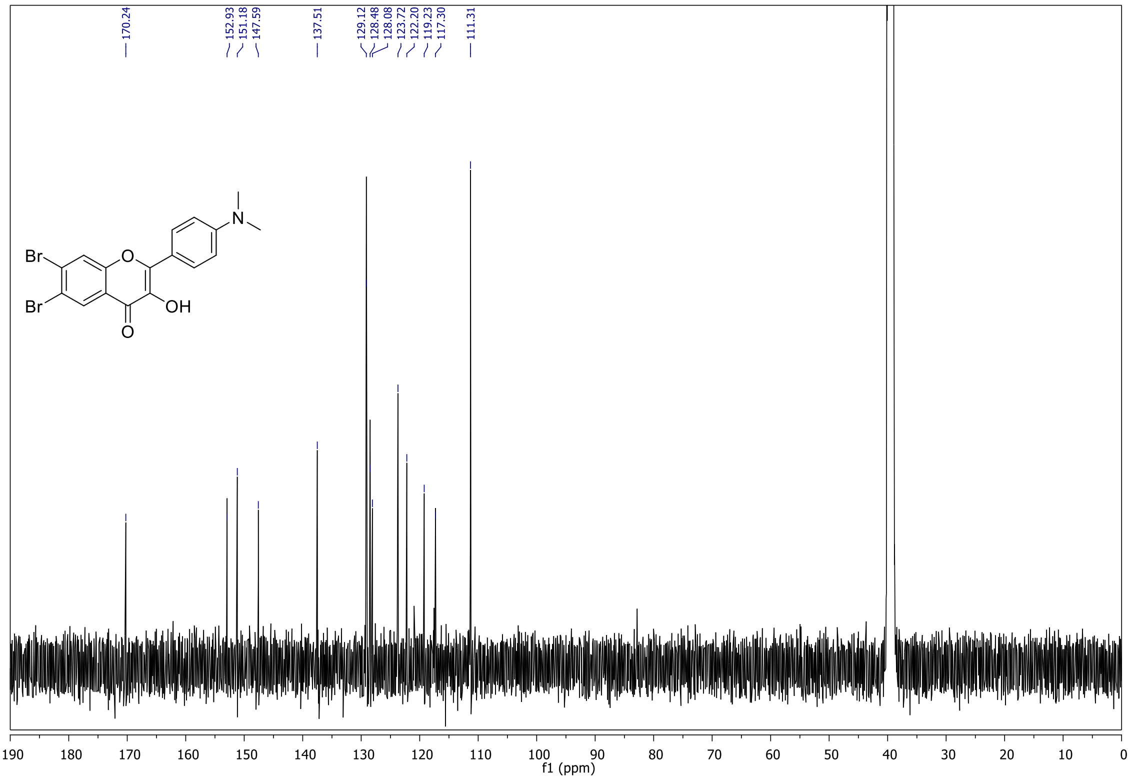

Figure S63. ^{13}C{^{1}H} NMR (125 MHz, DMSO-d_6): **26**.

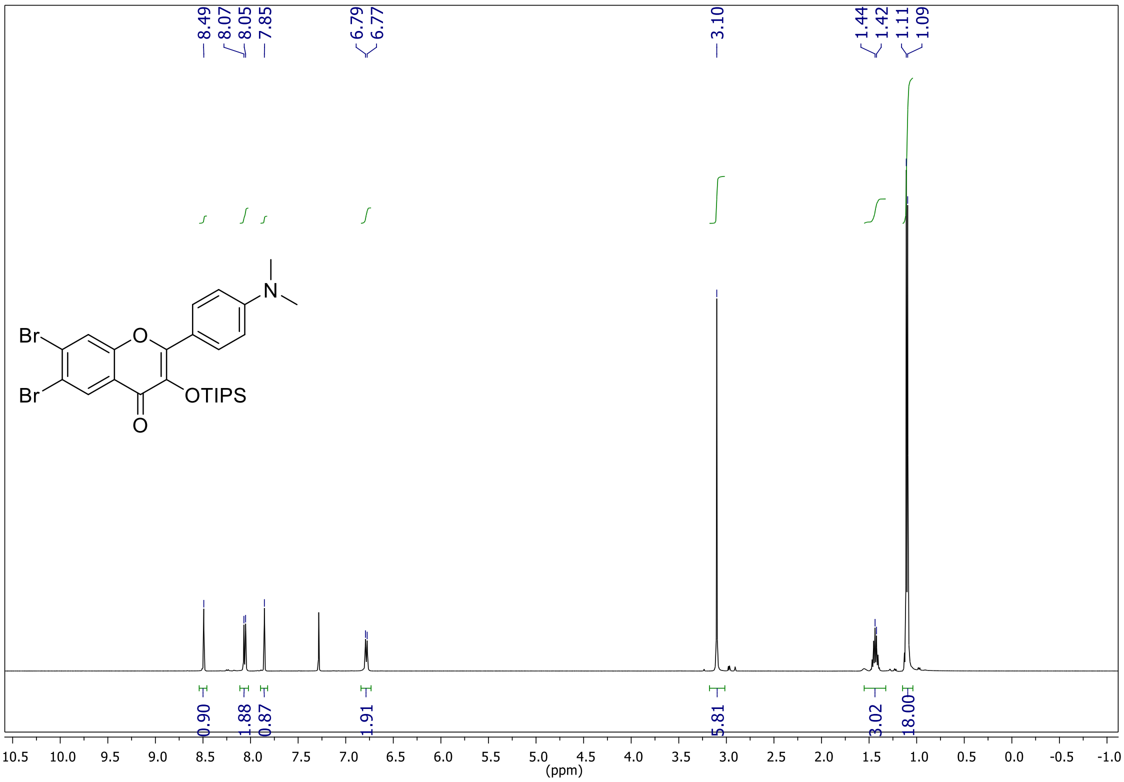

Figure S64. ^{1}H NMR (500 MHz, $CDCl_3$): **26-TIPS**.

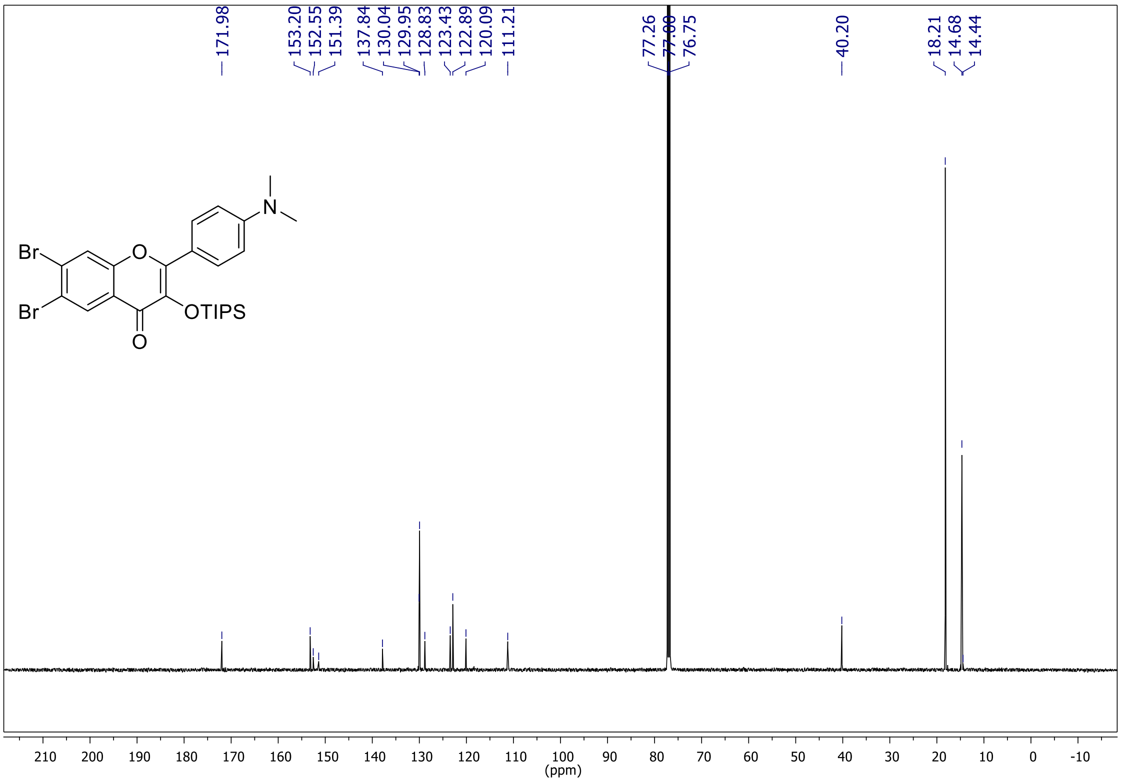

Figure S65. $^{13}C\{^{1}H\}$ NMR (125 MHz, $CDCl_3$): **26-TIPS**.

HRMS Spectra

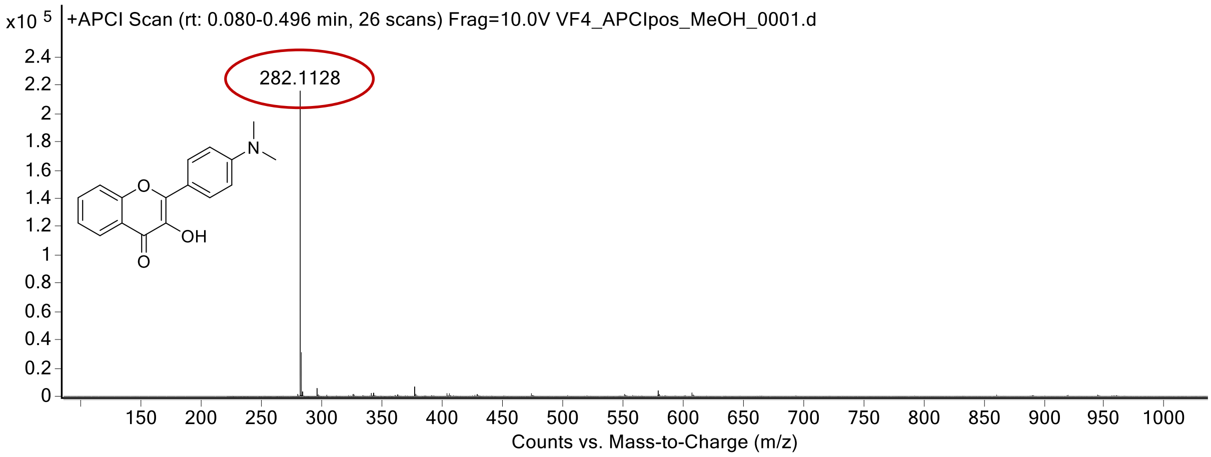

Figure S66. HRMS: **3**. APCI+ (MMI): nitrogen flow 5 L min^{-1}, gas temperature 325 °C, nebulizer 45 psig, skimmer 65 V, vaporizer 200 °C, fragmentor 10 V.

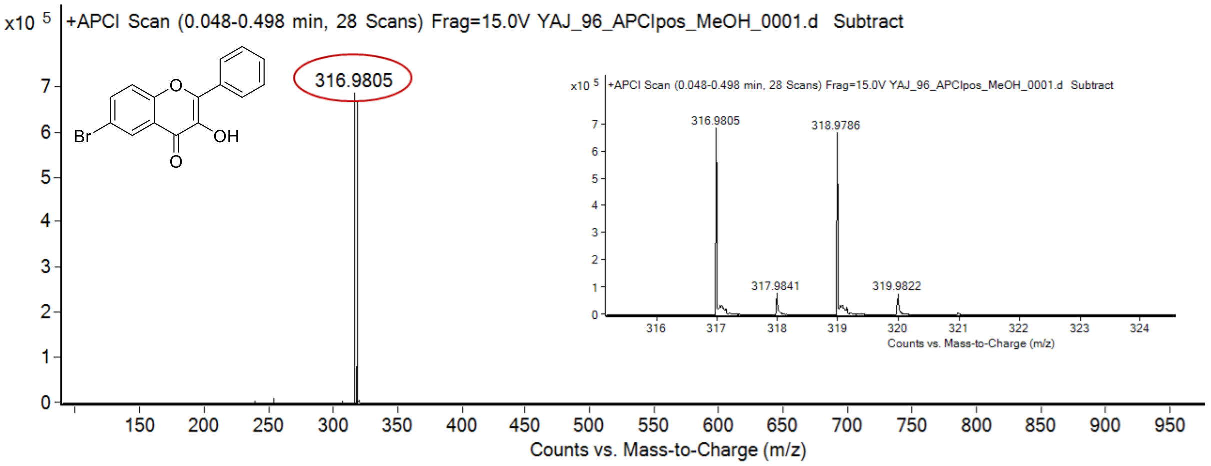

Figure S67. HRMS: **4**. APCI+ (MMI): nitrogen flow 3 L min^{-1}, gas temperature 325 °C, nebulizer 45 psig, skimmer 65 V, vaporizer 200 °C, fragmentor 15 V.

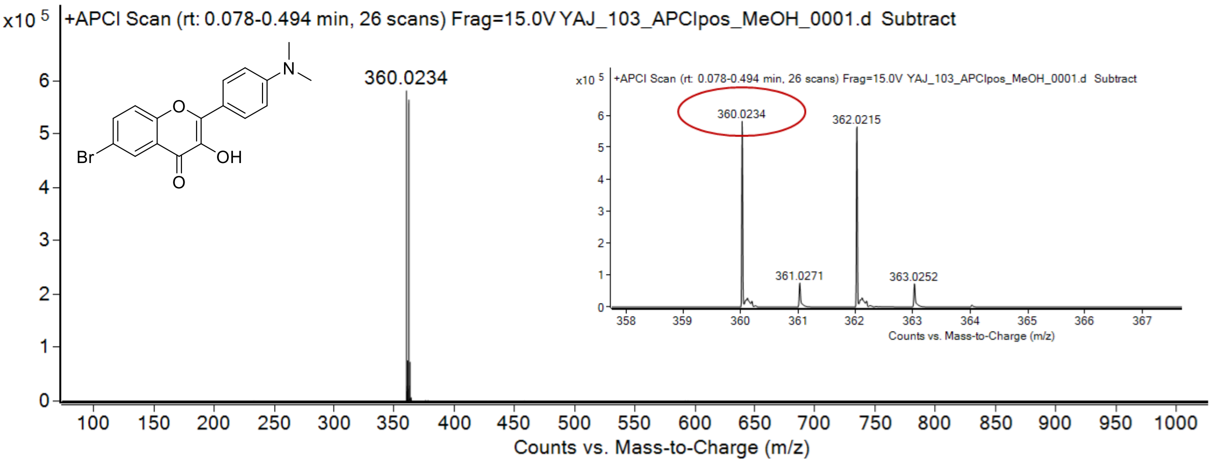

Figure S68. HRMS: **5**. APCI+ (MMI): nitrogen flow 5 L min^{-1}, gas temperature 325 °C, nebulizer 45 psig, skimmer 65 V, vaporizer 200 °C, fragmentor 15 V.

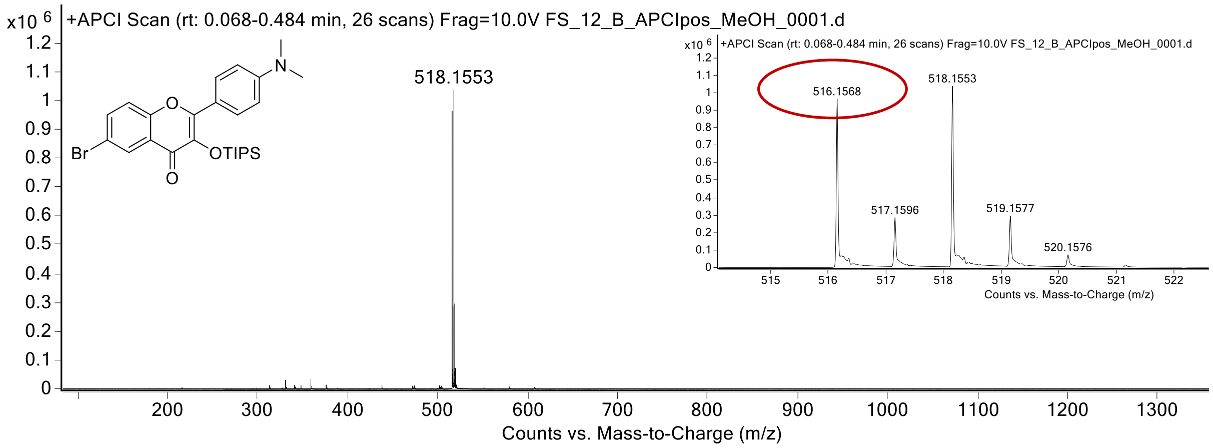

Figure S69. HRMS: **5-TIPS**. APCI+ (MMI): nitrogen flow 5 L min^{-1}, gas temperature 325 °C, nebulizer 45 psig, skimmer 65 V, vaporizer 200 °C, fragmentor 10 V.

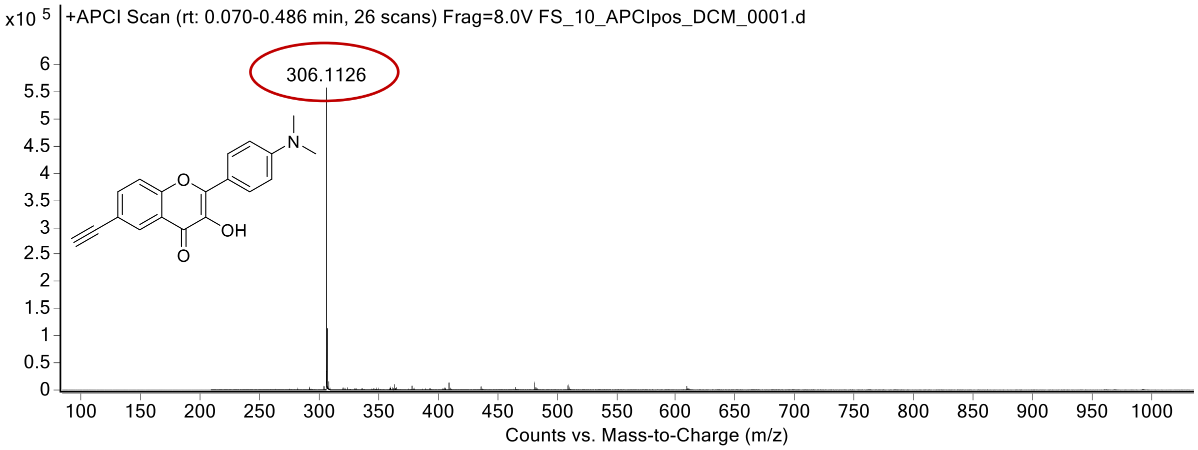

Figure S70. HRMS: **6**. APCI+ (MMI): nitrogen flow 5 L min^{-1}, gas temperature 325 °C, nebulizer 45 psig, skimmer 65 V, vaporizer 200 °C, fragmentor 8 V.

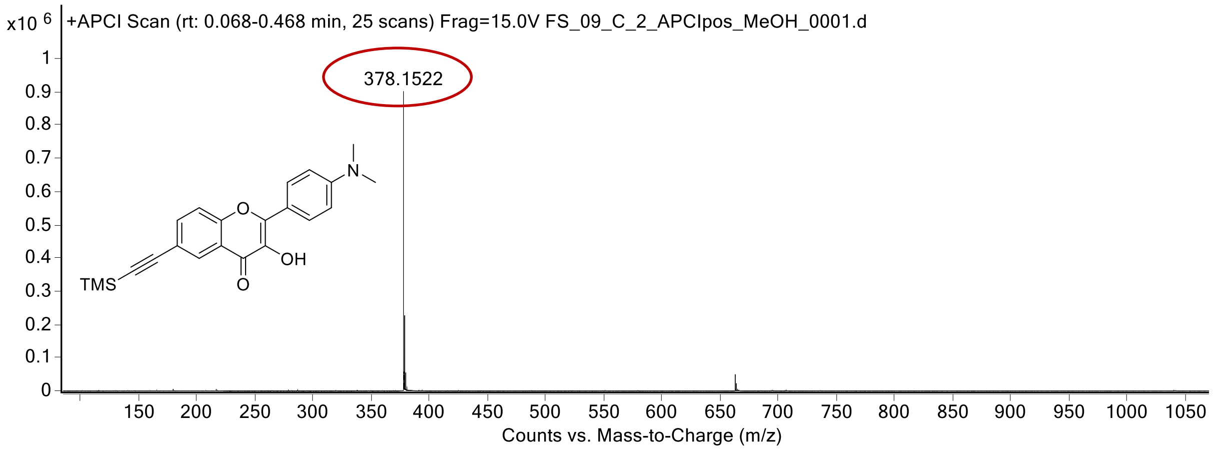

Figure S71. HRMS: **6-TMS**. APCI+ (MMI): nitrogen flow 5 L min^{-1}, gas temperature 325 °C, nebulizer 45 psig, skimmer 65 V, vaporizer 200 °C, fragmentor 15 V.

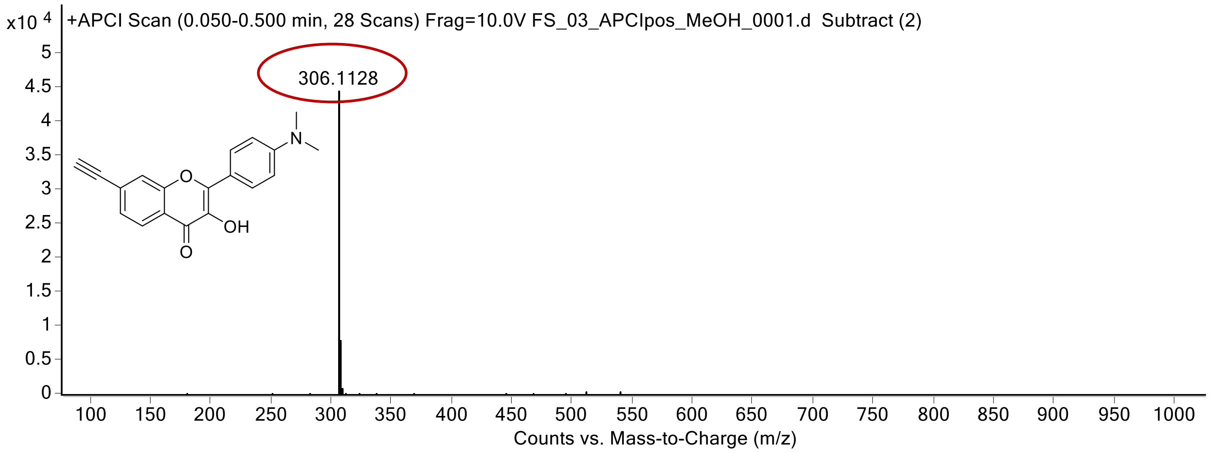

Figure S72. HRMS: **7**. APCI+ (MMI): nitrogen flow 5 L min^{-1}, gas temperature 325 °C, nebulizer 45 psig, skimmer 65 V, vaporizer 200 °C, fragmentor 10 V.

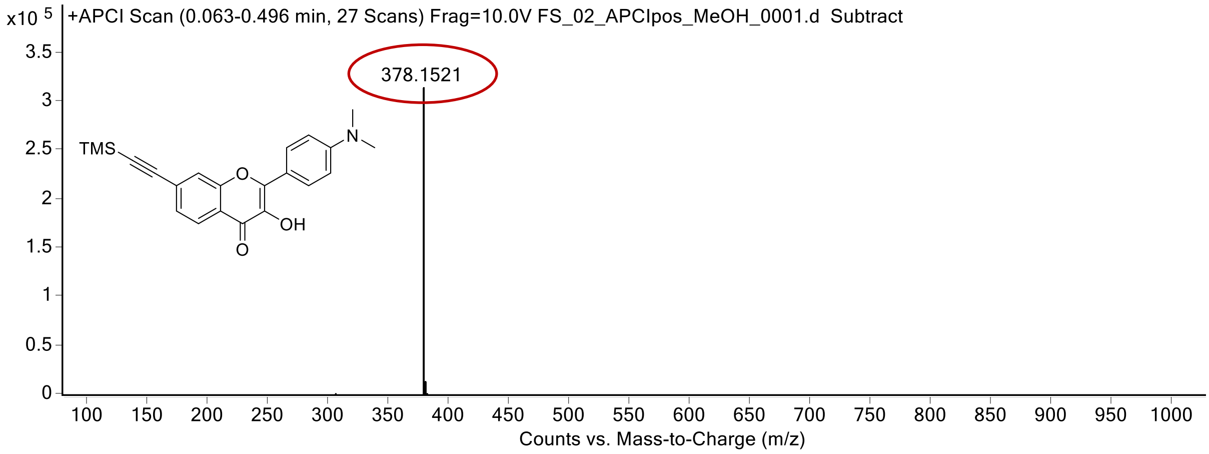

Figure S73. HRMS: **7-TMS**. APCI+ (MMI): nitrogen flow 5 L min^{-1}, gas temperature 325 °C, nebulizer 45 psig, skimmer 65 V, vaporizer 200 °C, fragmentor 10 V.

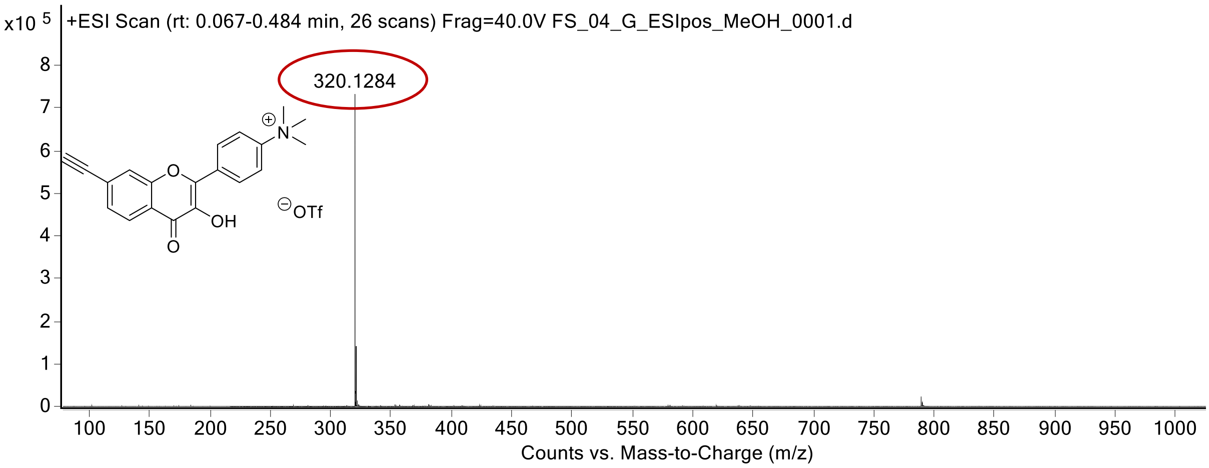

Figure S74. HRMS: **8**. ESI+ (MMI): nitrogen flow 5 L min^{-1}, gas temperature 325 °C, nebulizer 45 psig, V_{cap} – 2500 V, vaporizer 200 °C, fragmentor 40 V.

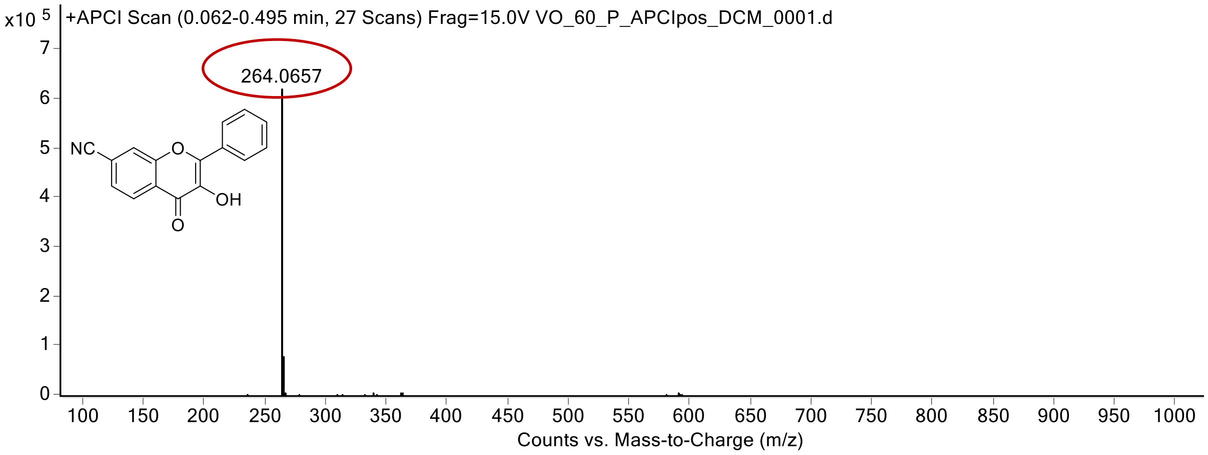

Figure S75. HRMS: **9**. APCI+ (MMI): nitrogen flow 5 L min^{-1}, gas temperature 325 °C, nebulizer 45 psig, skimmer 65 V, vaporizer 200 °C, fragmentor 15 V.

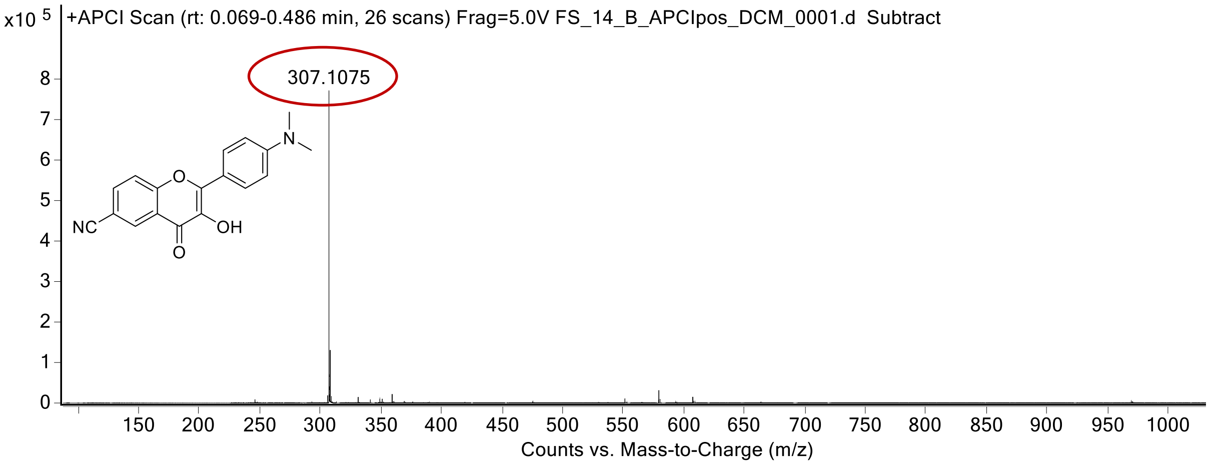

Figure S76. HRMS: **10**. APCI+ (MMI): nitrogen flow 5 L min^{-1}, gas temperature 325 °C, nebulizer 45 psig, skimmer 65 V, vaporizer 200 °C, fragmentor 5 V.

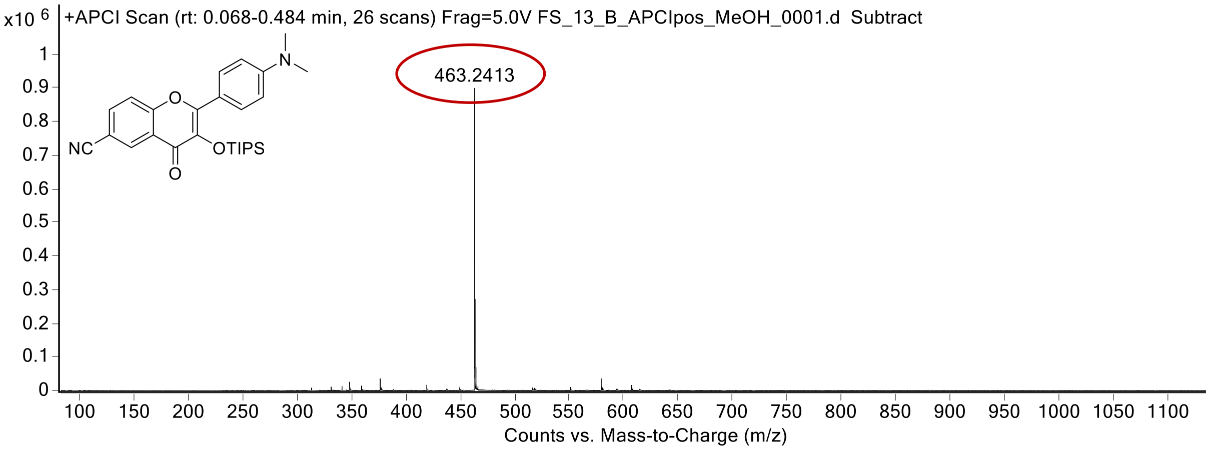

Figure S77. HRMS: **10-TIPS**. APCI+ (MMI): nitrogen flow 5 L min^{-1}, gas temperature 325 °C, nebulizer 45 psig, skimmer 65 V, vaporizer 200 °C, fragmentor 5 V.

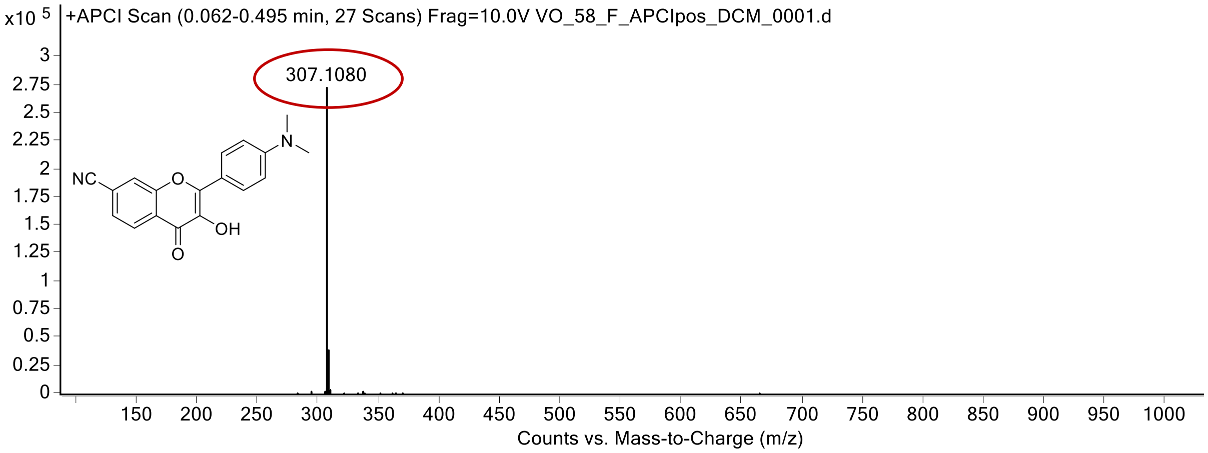

Figure S78. HRMS: **11**. APCI+ (MMI): nitrogen flow 5 L min^{-1}, gas temperature 325 °C, nebulizer 45 psig, skimmer 65 V, vaporizer 200 °C, fragmentor 10 V.

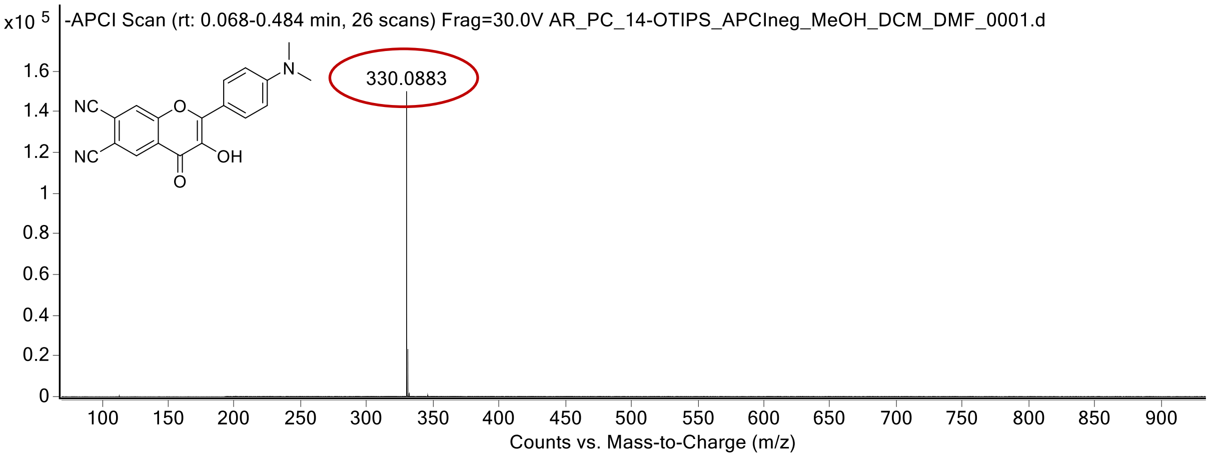

Figure S79. HRMS: **12**. APCI– (MMI): nitrogen flow 5 L min^{-1}, gas temperature 325 °C, nebulizer 45 psig, skimmer –65 V, vaporizer 200 °C, fragmentor –30 V.

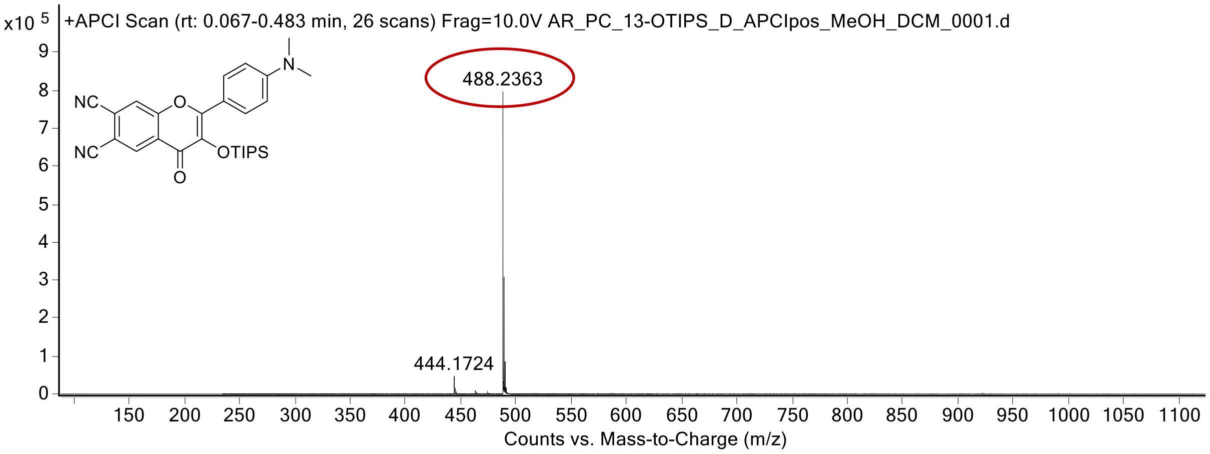

Figure S80. HRMS: **12-TIPS**. APCI+ (MMI): nitrogen flow 5 L min^{-1}, gas temperature 325 °C, nebulizer 45 psig, skimmer 65 V, vaporizer 200 °C, fragmentor 10 V.

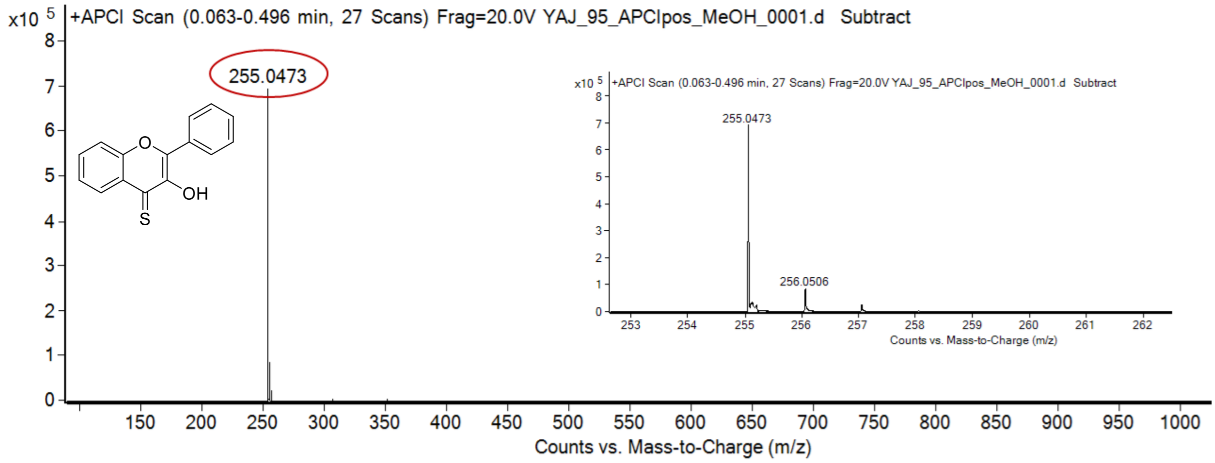

Figure S81. HRMS: **13**. APCI+ (MMI): nitrogen flow 3 L min^{-1}, gas temperature 325 °C, nebulizer 45 psig, skimmer 65 V, vaporizer 200 °C, fragmentor 20 V.

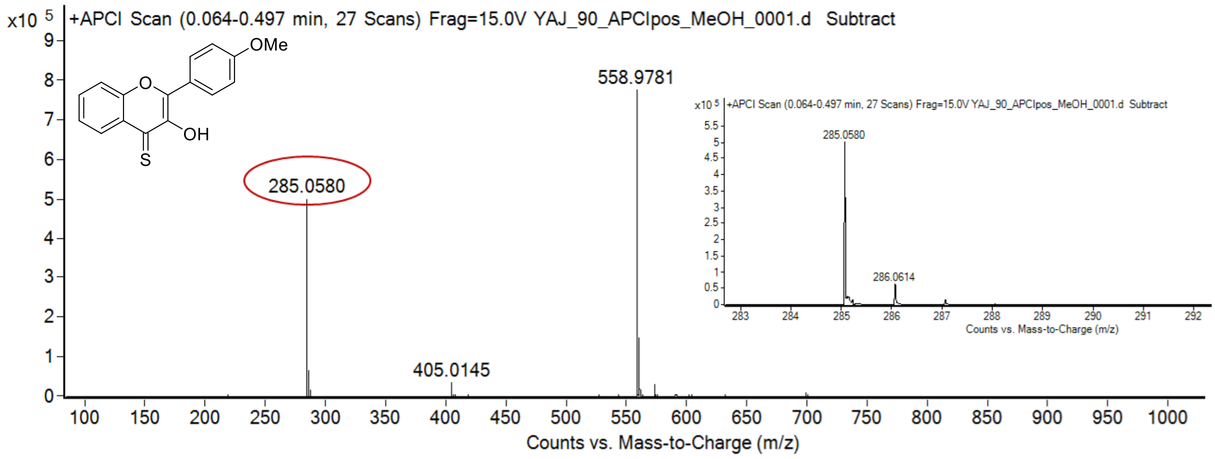

Figure S82. HRMS: **14**. APCI+ (MMI): nitrogen flow 3 L min^{-1}, gas temperature 325 °C, nebulizer 45 psig, skimmer 65 V, vaporizer 200 °C, fragmentor 15 V.

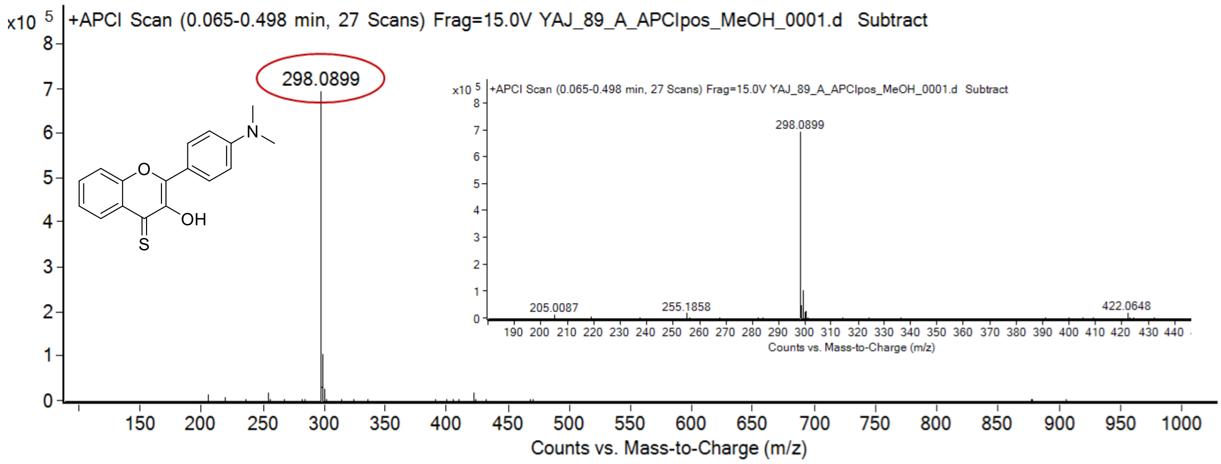

Figure S83. HRMS: **15**. APCI+ (MMI): nitrogen flow 3 L min^{-1}, gas temperature 325 °C, nebulizer 45 psig, skimmer 65 V, vaporizer 200 °C, fragmentor 15 V.

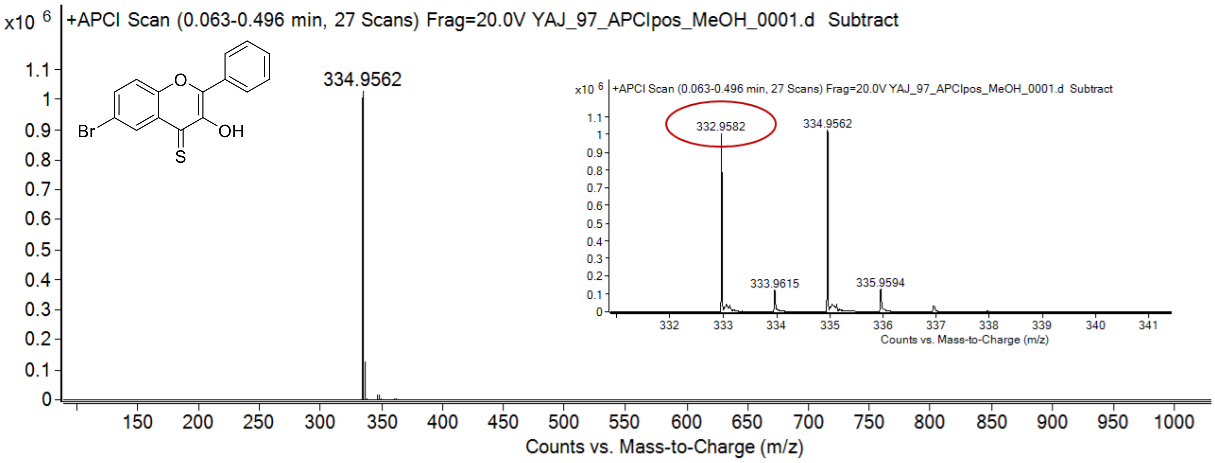

Figure S84. HRMS: **16**. APCI+ (MMI): nitrogen flow 3 L min^{-1}, gas temperature 325 °C, nebulizer 45 psig, skimmer 65 V, vaporizer 200 °C, fragmentor 20 V.

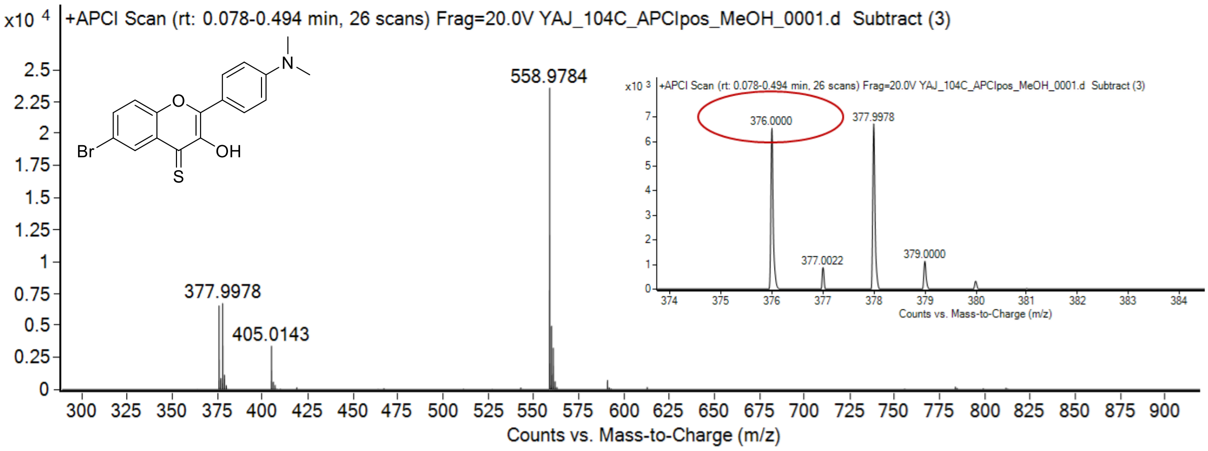

Figure S85. HRMS: **17**. APCI+ (MMI): nitrogen flow 5 L min^{-1}, gas temperature 325 °C, nebulizer 45 psig, skimmer 65 V, vaporizer 200 °C, fragmentor 20 V.

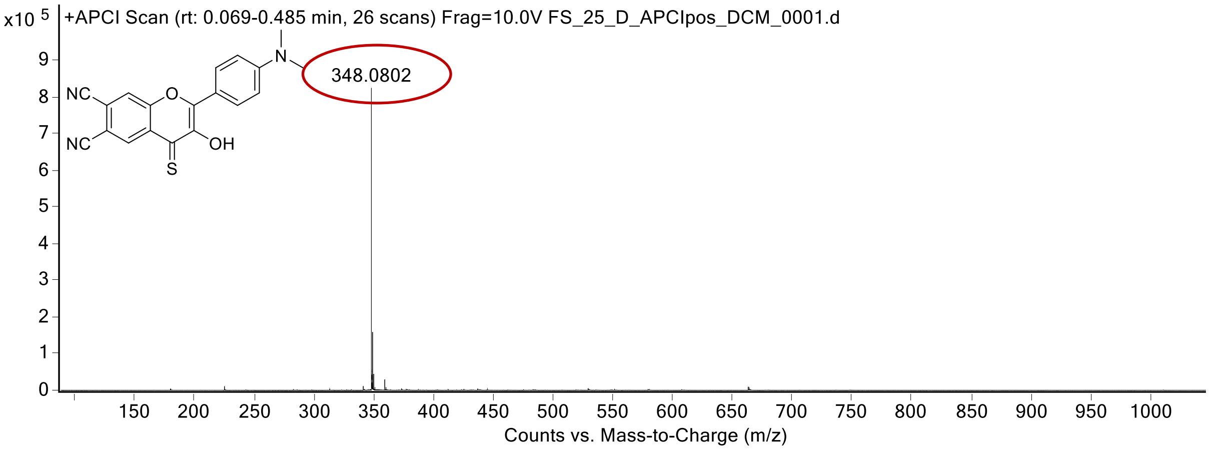

Figure S86. HRMS: **18**. APCI+ (MMI): nitrogen flow 5 L min^{-1}, gas temperature 325 °C, nebulizer 45 psig, skimmer 65 V, vaporizer 200 °C, fragmentor 10 V.

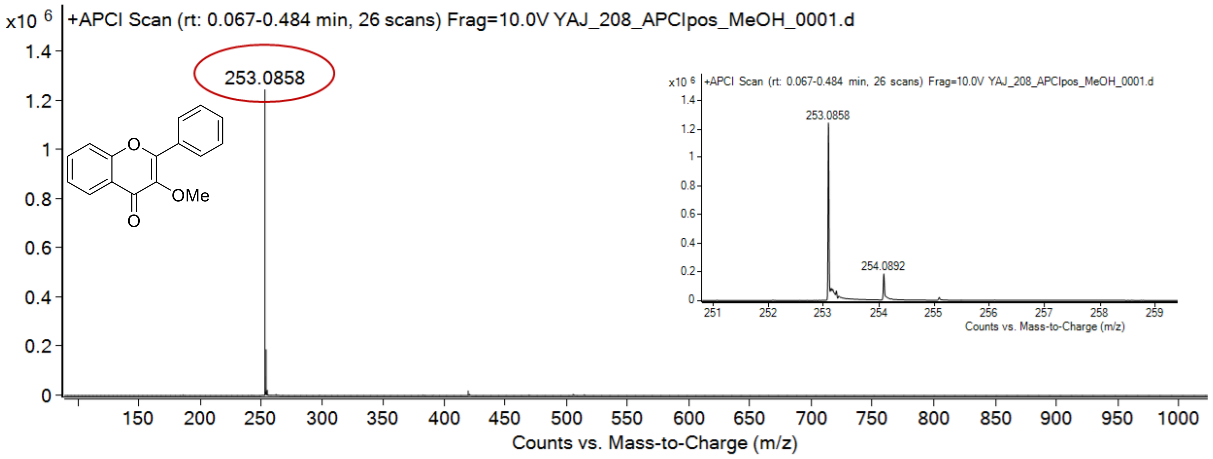

Figure S87. HRMS: **19**. APCI+ (MMI): nitrogen flow 5 L min^{-1}, gas temperature 325 °C, nebulizer 45 psig, skimmer 65 V, vaporizer 200 °C, fragmentor 10 V.

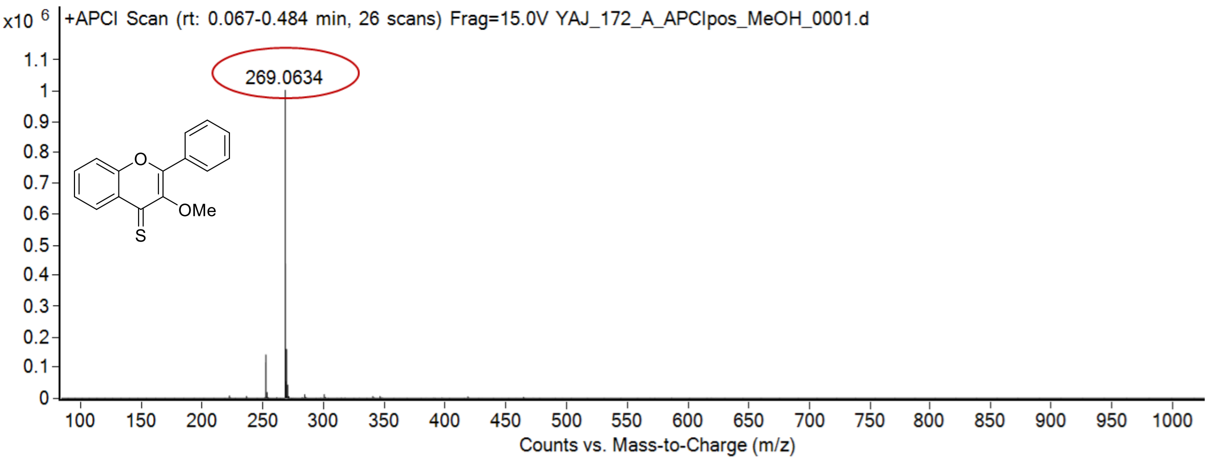

Figure S88. HRMS: **20**. APCI+ (MMI): nitrogen flow 5 L min^{-1}, gas temperature 325 °C, nebulizer 45 psig, skimmer 65 V, vaporizer 200 °C, fragmentor 15 V.

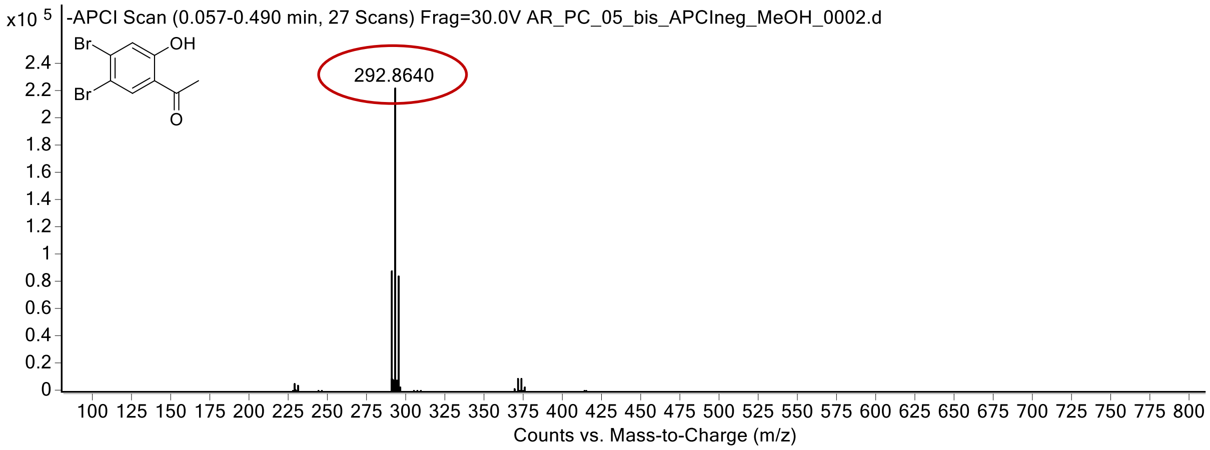

Figure S89. HRMS: **21d**. APCI– (MMI): nitrogen flow 5 L min^{-1}, gas temperature 325 °C, nebulizer 45 psig, skimmer –65 V, vaporizer 200 °C, fragmentor –30 V.

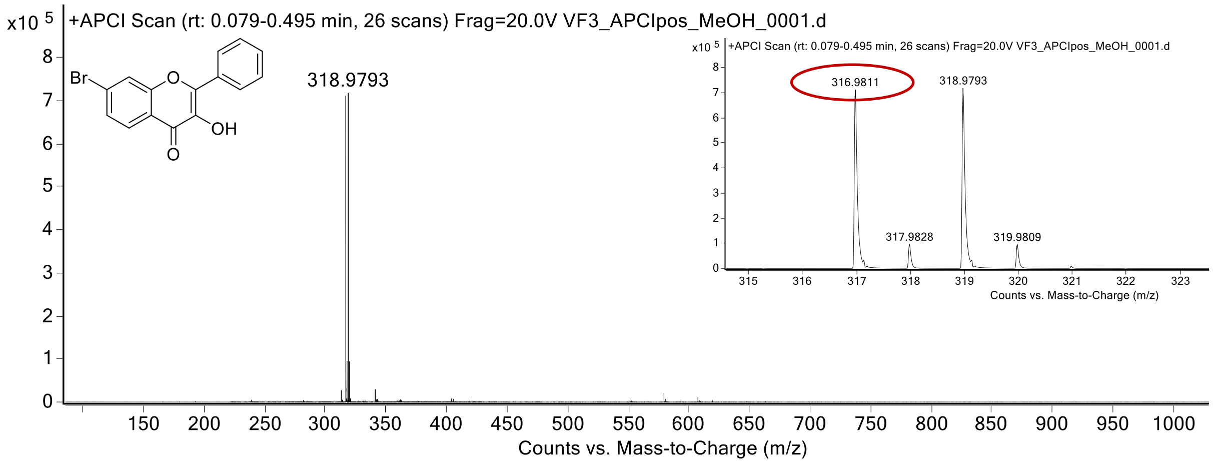

Figure S90. HRMS: **24**. APCI+ (MMI): nitrogen flow 5 L min^{-1}, gas temperature 325 °C, nebulizer 45 psig, skimmer 65 V, vaporizer 200 °C, fragmentor 20 V.

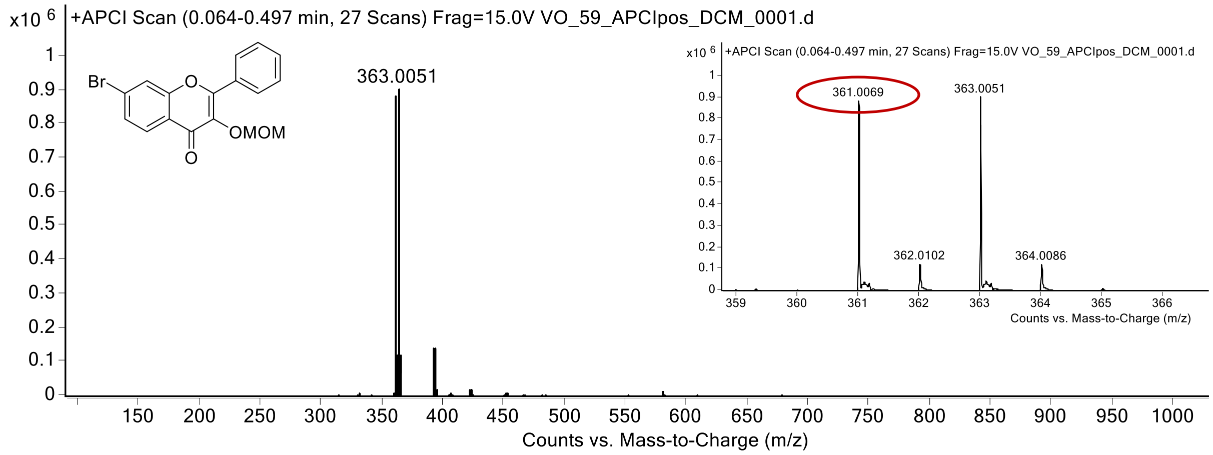

Figure S91. HRMS: **24-MOM**. APCI+ (MMI): nitrogen flow 5 L min^{-1}, gas temperature 325 °C, nebulizer 45 psig, skimmer 65 V, vaporizer 200 °C, fragmentor 15 V.

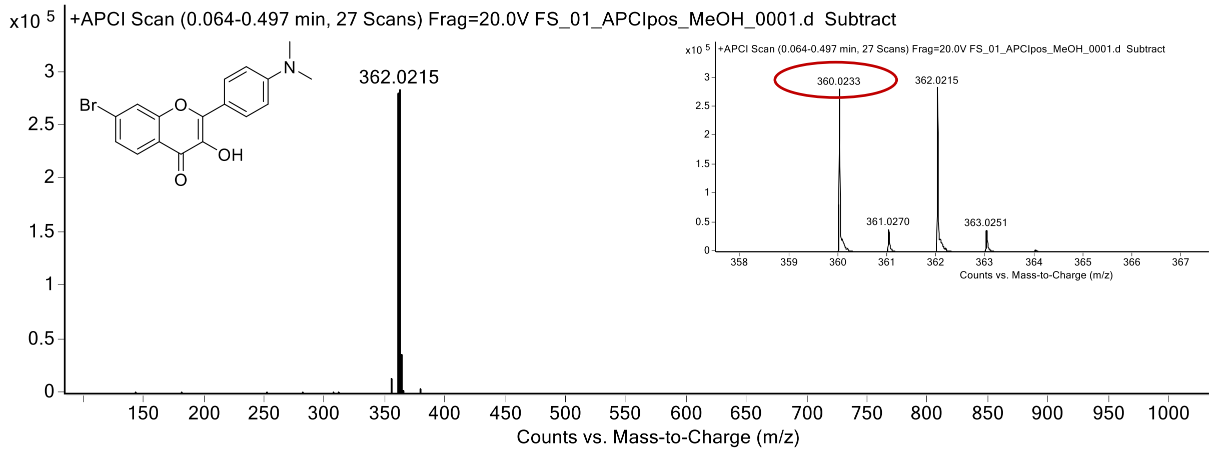

Figure S92. HRMS: **25**. APCI+ (MMI): nitrogen flow 5 L min^{-1}, gas temperature 325 °C, nebulizer 45 psig, skimmer 65 V, vaporizer 200 °C, fragmentor 20 V.

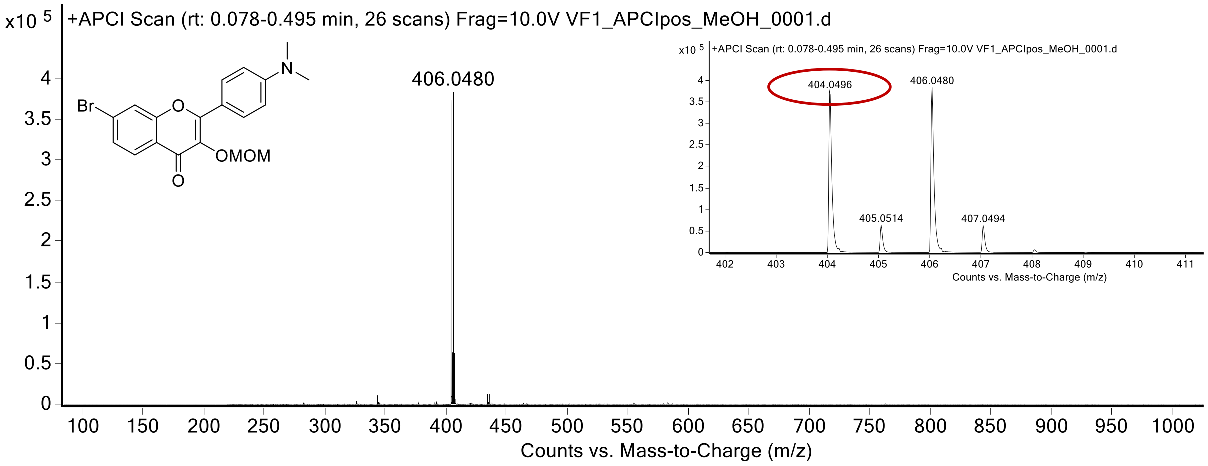

Figure S93. HRMS: **25-MOM**. APCI+ (MMI): nitrogen flow 5 L min^{-1}, gas temperature 325 °C, nebulizer 45 psig, skimmer 65 V, vaporizer 200 °C, fragmentor 10 V.

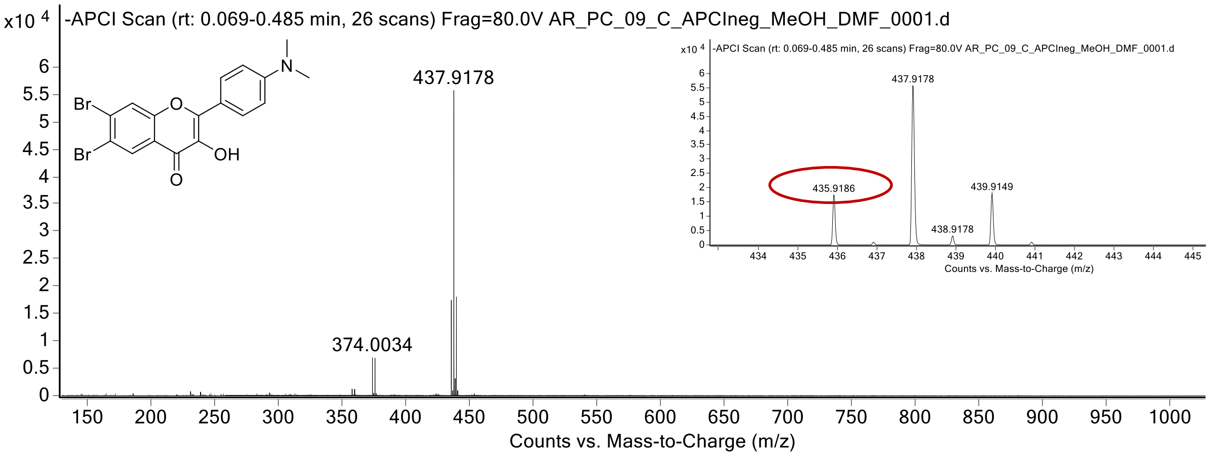

Figure S94. HRMS: **26**. APCI– (MMI): nitrogen flow 5 L min^{-1}, gas temperature 325 °C, nebulizer 45 psig, skimmer –65 V, vaporizer 200 °C, fragmentor –80 V.

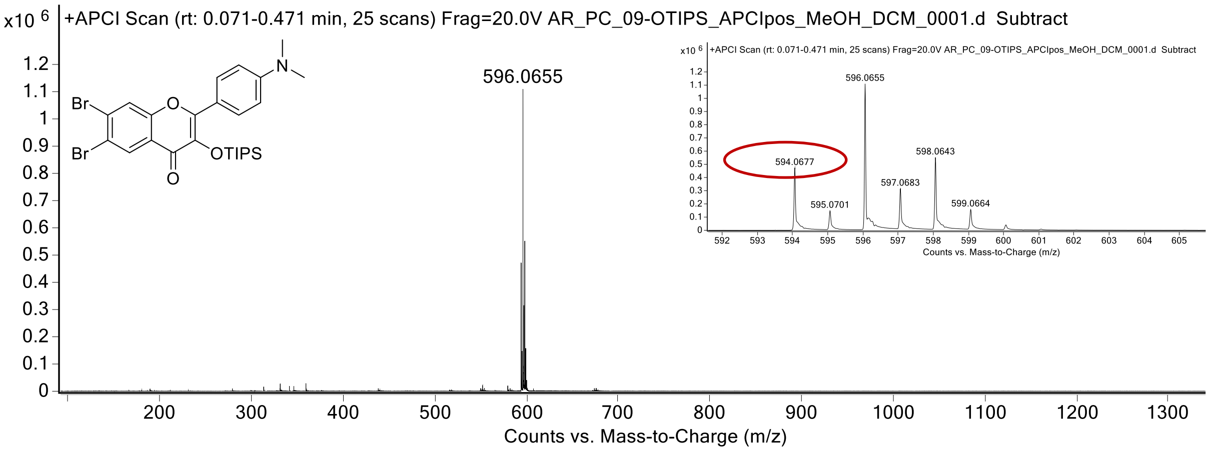

Figure S95. HRMS: **26-TIPS**. APCI+ (MMI): nitrogen flow 5 L min^{-1}, gas temperature 325 °C, nebulizer 45 psig, skimmer 65 V, vaporizer 200 °C, fragmentor 20 V.

Absorption and Emission Spectra

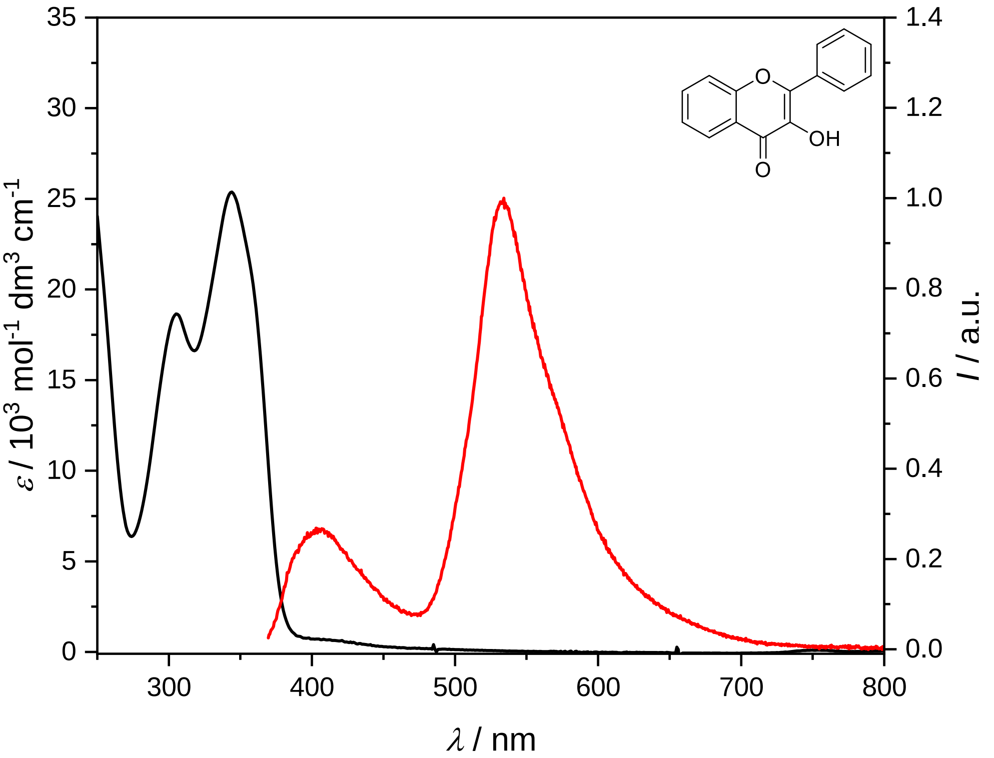

Figure S96. UV-VIS absorption (black line) and normalized emission (red line) of **1A** (c ~10^{-5} M, methanol).

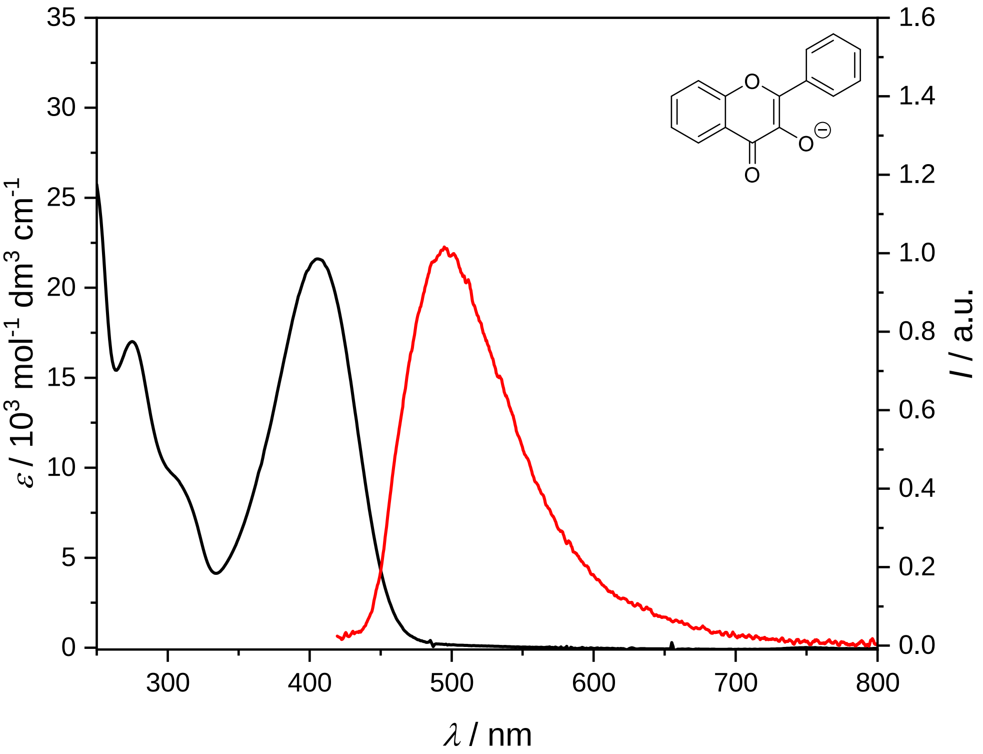

Figure S97. UV-VIS absorption (black line) and normalized emission (red line) of **1B** (c ~10^{-5} M, methanol, ~10 equiv. of NaOH).

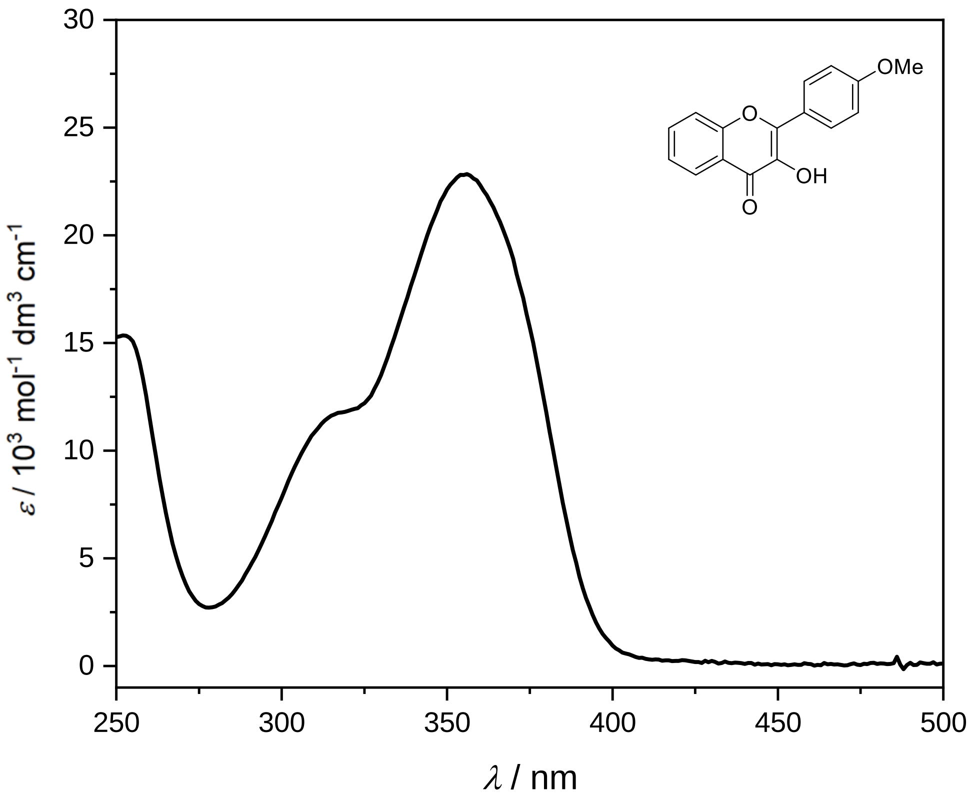

Figure S98. UV-VIS absorption of **2A** (c ~10^{-5} M, methanol).

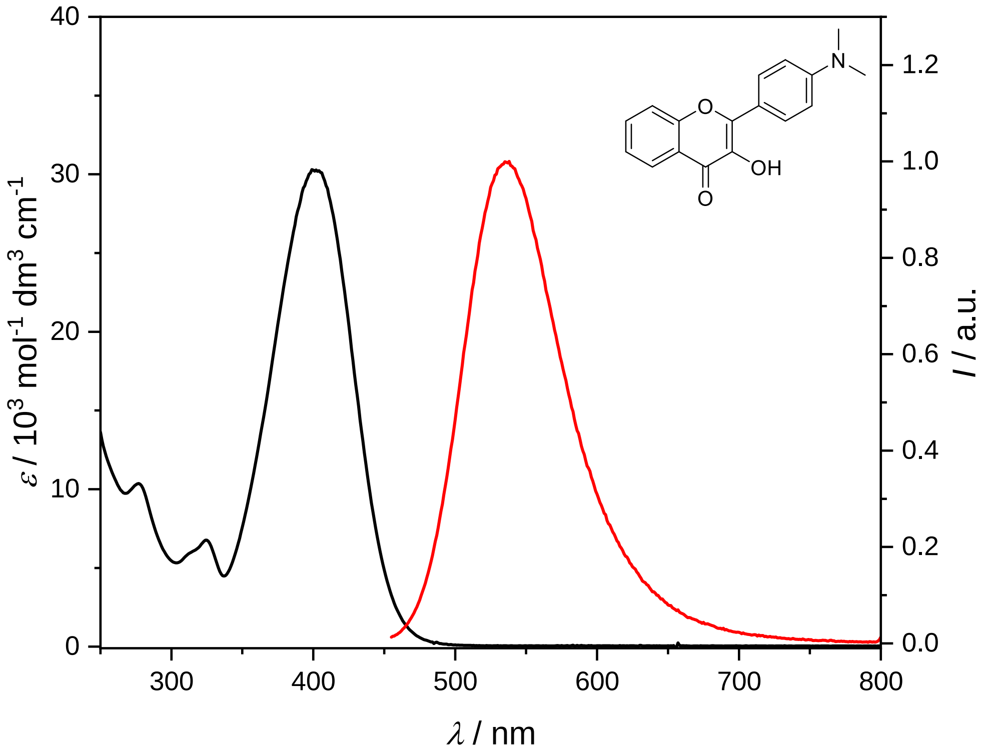

Figure S99. UV-VIS absorption (black line) and normalized emission (red line) of **3A** (c ~10^{-5} M, methanol/DMSO, 90:10).

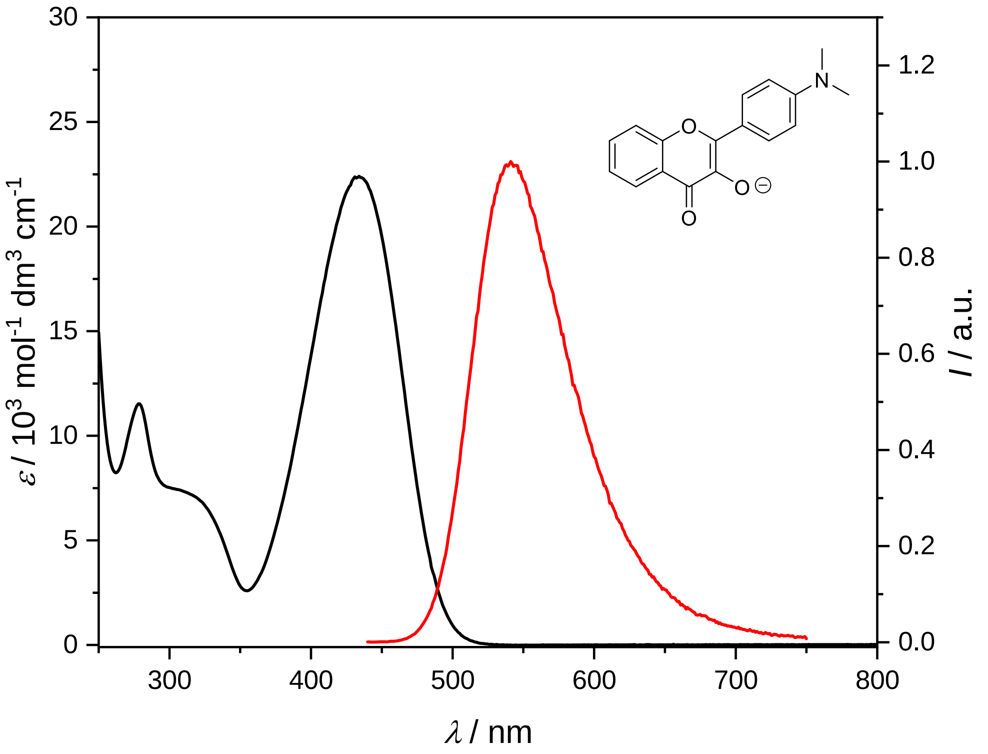

Figure S100. UV-VIS absorption (black line) and normalized emission (red line) of **3B** (c ~10^{-5} M, methanol/DMSO 90:10, ~400 equiv. of NaOH).

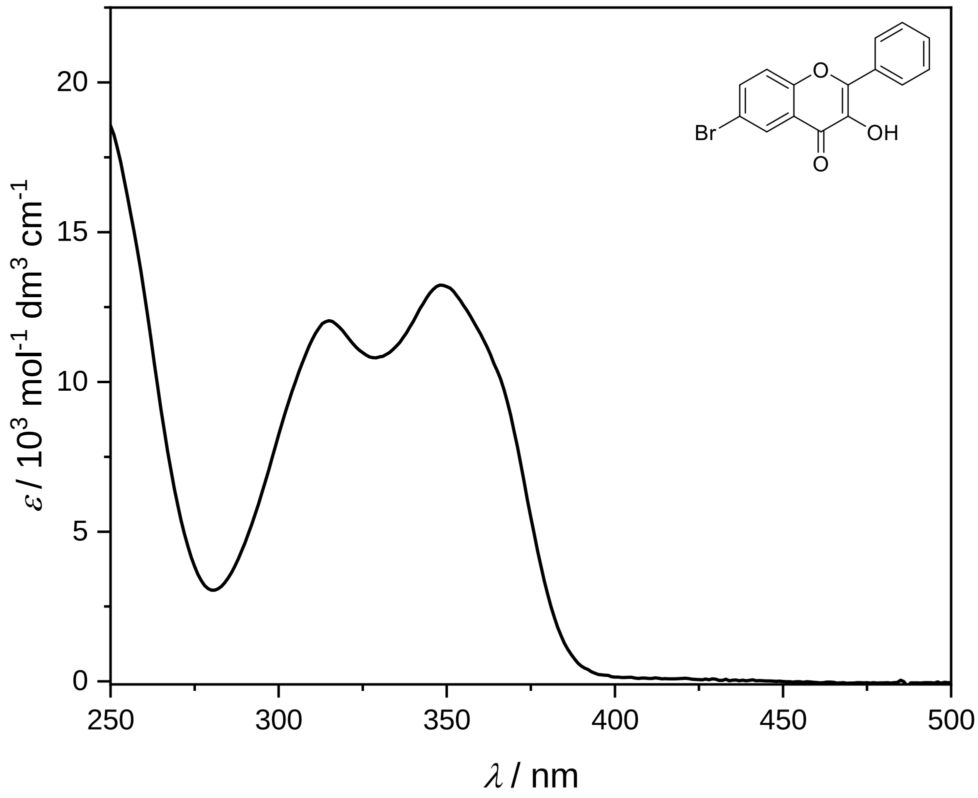

Figure S101. UV-VIS absorption of **4A** (c ~10^{-5} M, methanol).

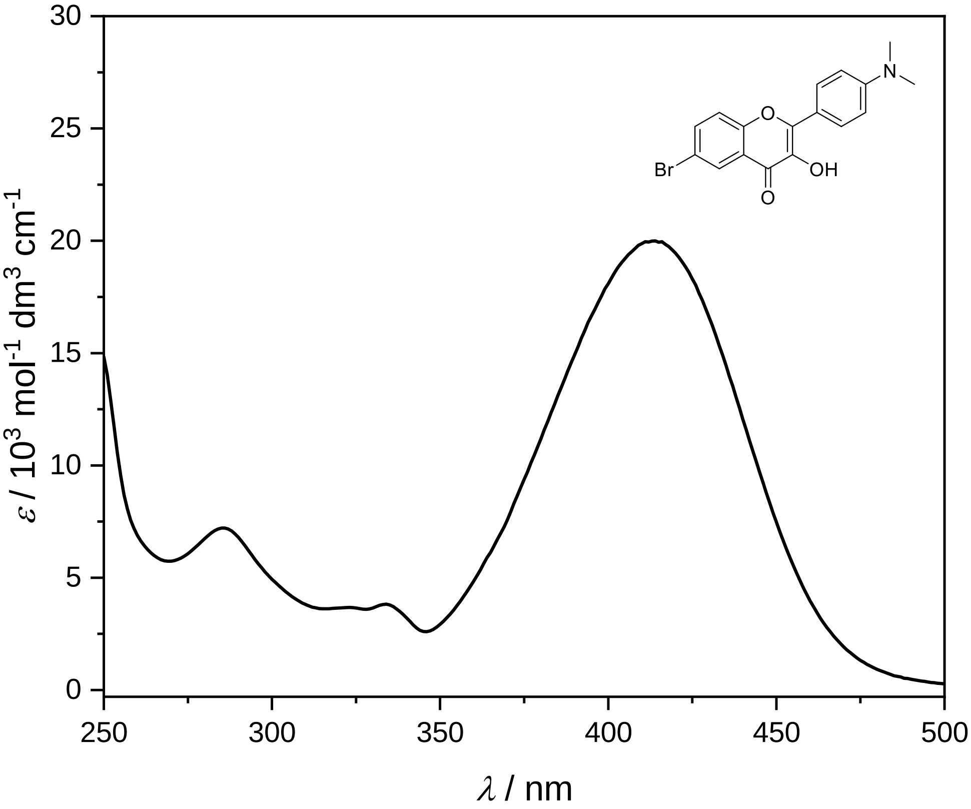

Figure S102. UV-VIS absorption of **5A** (c ~7 10^{-5} M, ethanol/DMSO, 98:2).

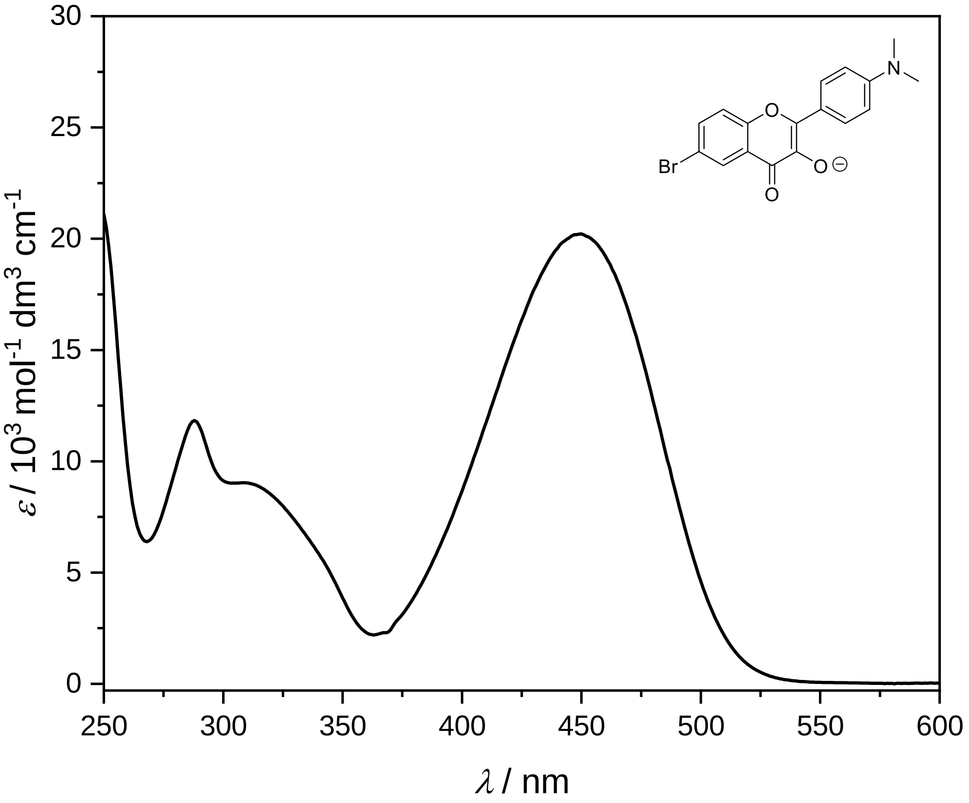

Figure S103. UV-VIS absorption of **5B** (c ~4 × 10^{-5} M, ethanol/DMSO, 98:2, ~200 equiv. of $NaOCH_3$).

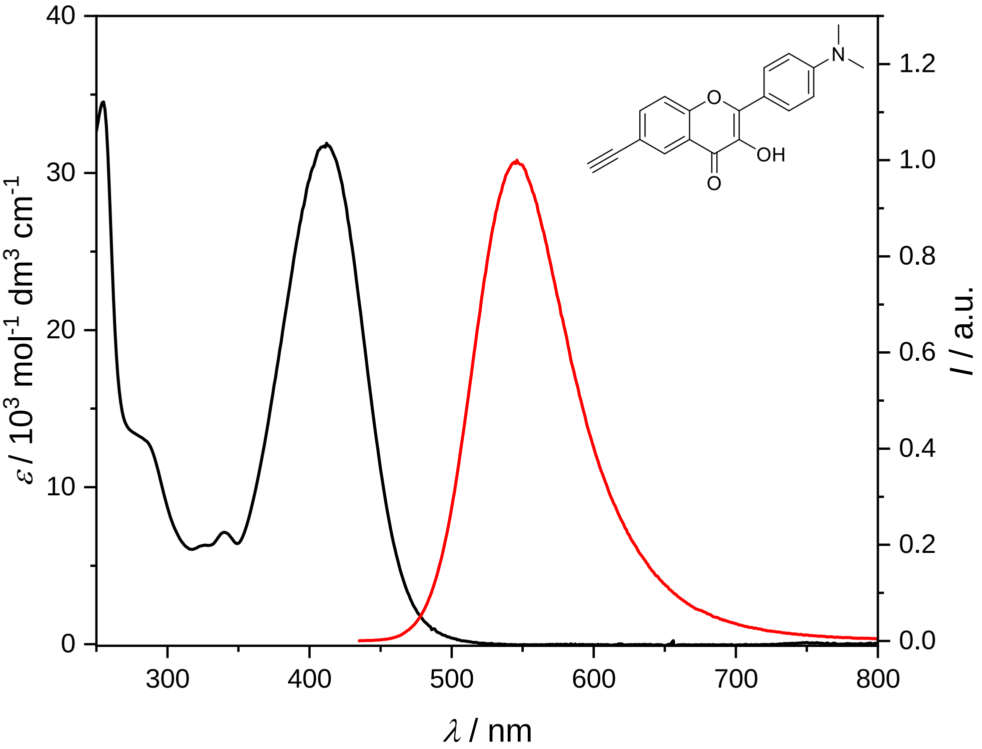

Figure S104. UV-VIS absorption (black line) and normalized emission (red line) of **6A** (c ~10^{-5} M, methanol/DMSO, 98:2).

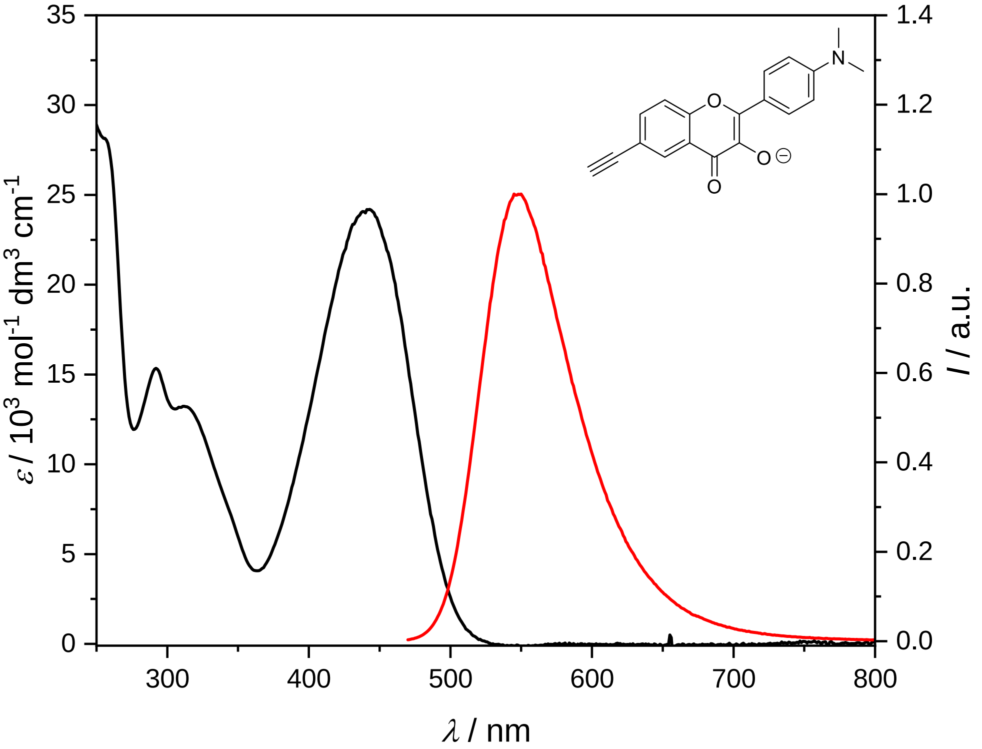

Figure S105. UV-VIS absorption (black line) and normalized emission (red line) of **6B** (c ~10^{-5} M, methanol/DMSO, 98:2, ~200 equiv. of NaOH).

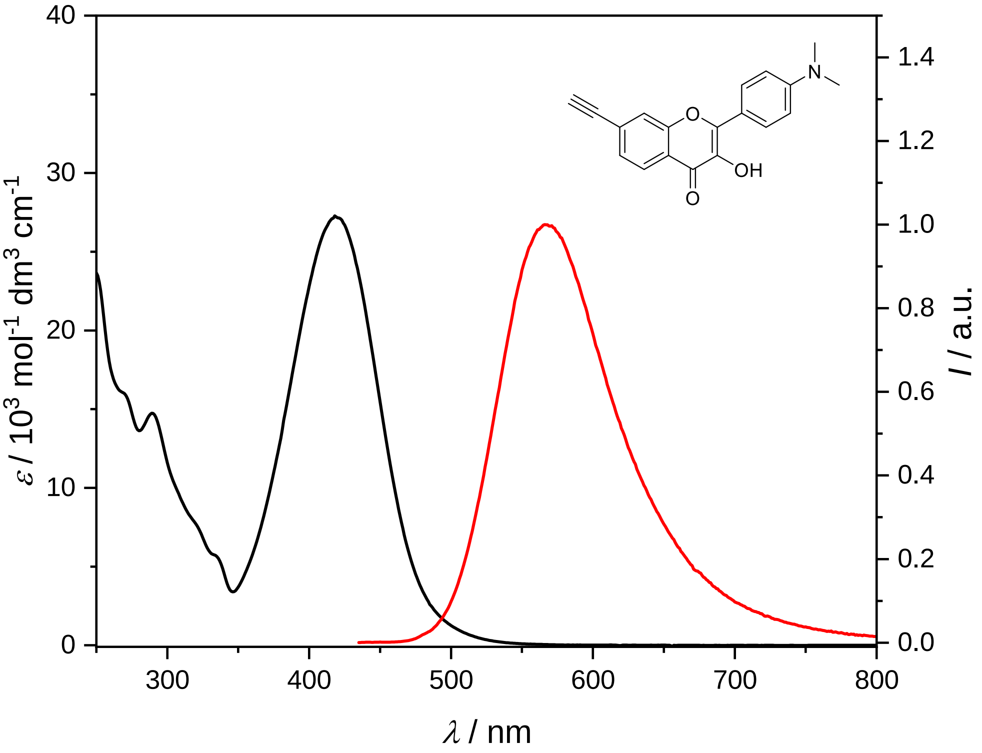

Figure S106. UV-VIS absorption (black line) and normalized emission (red line) of **7A** (c ~10^{-5} M, methanol/DMSO, 98:2).

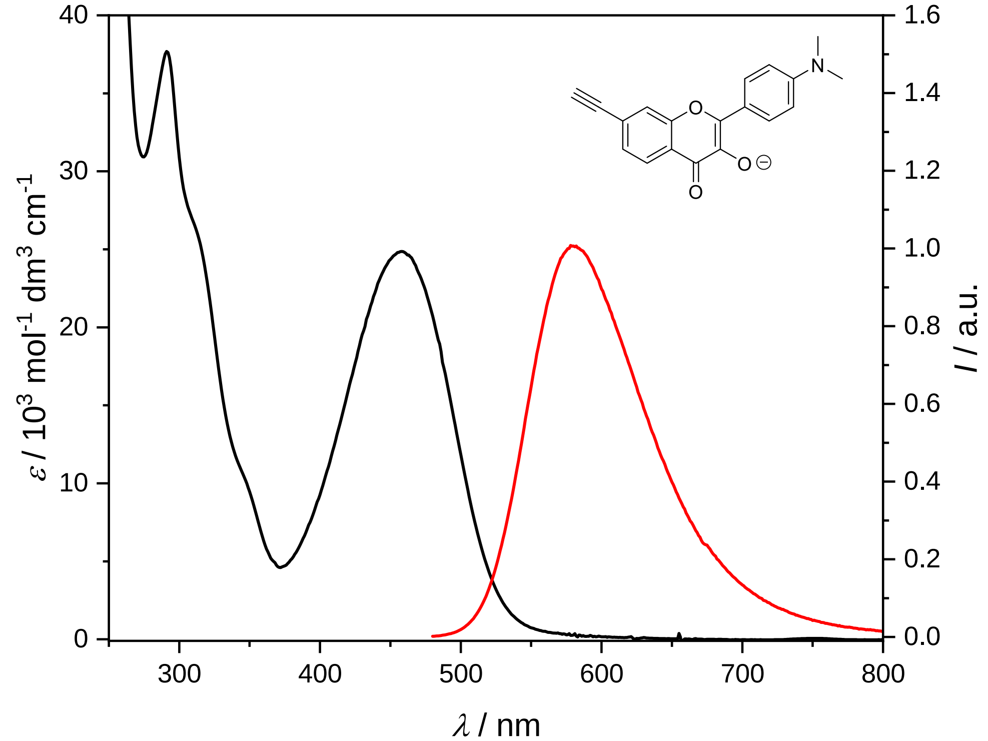

Figure S107. UV-VIS absorption (black line) and normalized emission (red line) of **7B** (c ~10^{-5} M, methanol/DMSO, 98:2, ~1000 equiv. of NaOH).

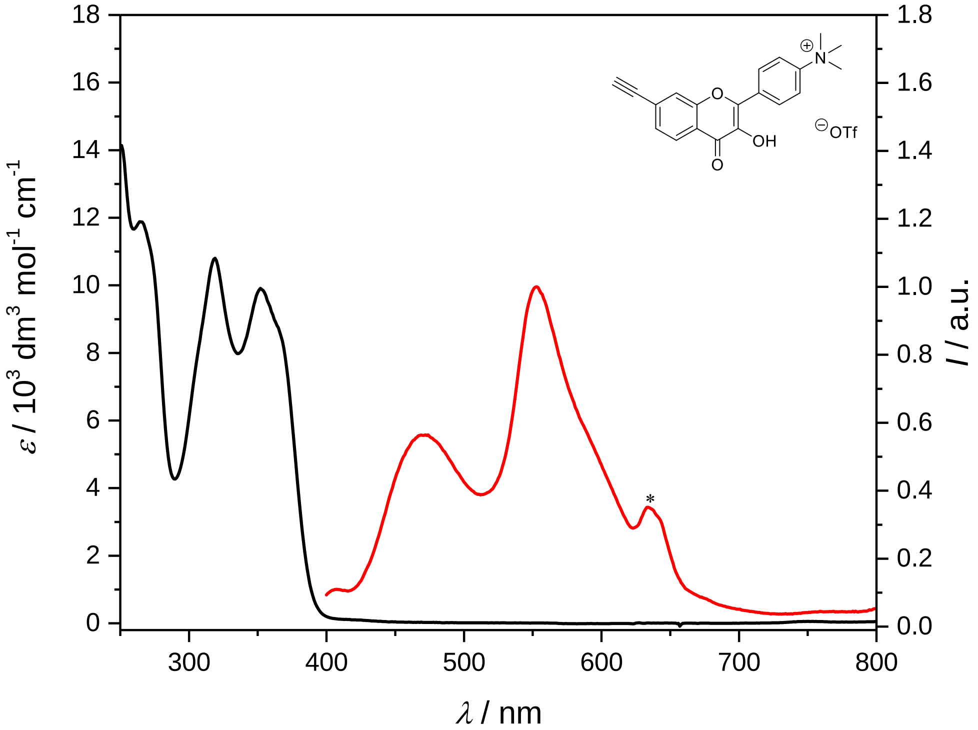

Figure S108. UV-VIS absorption (black line) and normalized emission (red line) of **8A** (*c* ~10^{-5} M, methanol/DMSO, 98:2, ~1 equiv. of HCl). The signal from the second-order diffraction from the lamp is denoted by an asterisk.

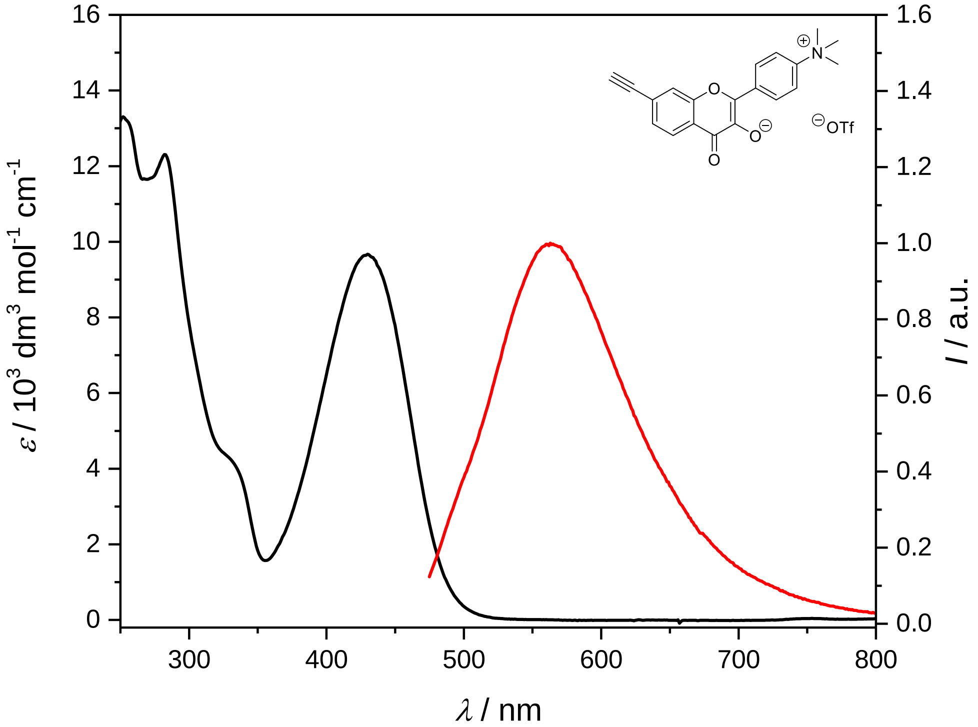

Figure S109. UV-VIS absorption (black line) and normalized emission (red line) of **8B** (*c* ~10^{-5} M, methanol/DMSO, 98:2, ~10 equiv. of NaOH).

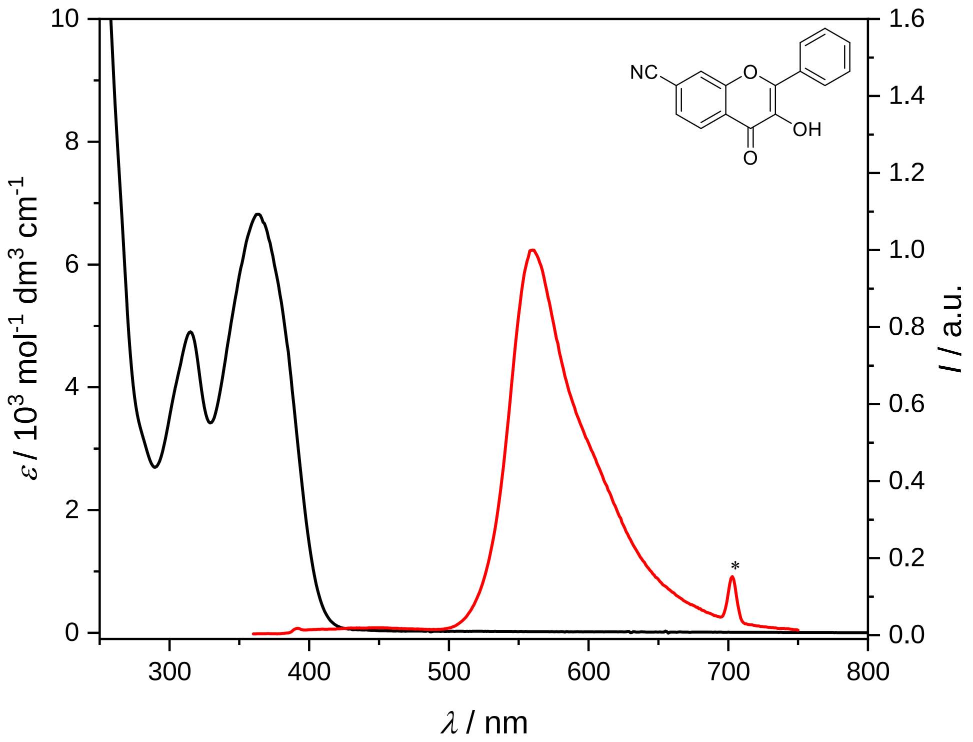

Figure S110. UV-VIS absorption (black line) and normalized emission (red line) of **9A** (*c* ~10^{-5} M, methanol/DMSO, 90:10, ~50 equiv. of HCl). The signal from the second-order diffraction from the lamp is denoted by an asterisk.

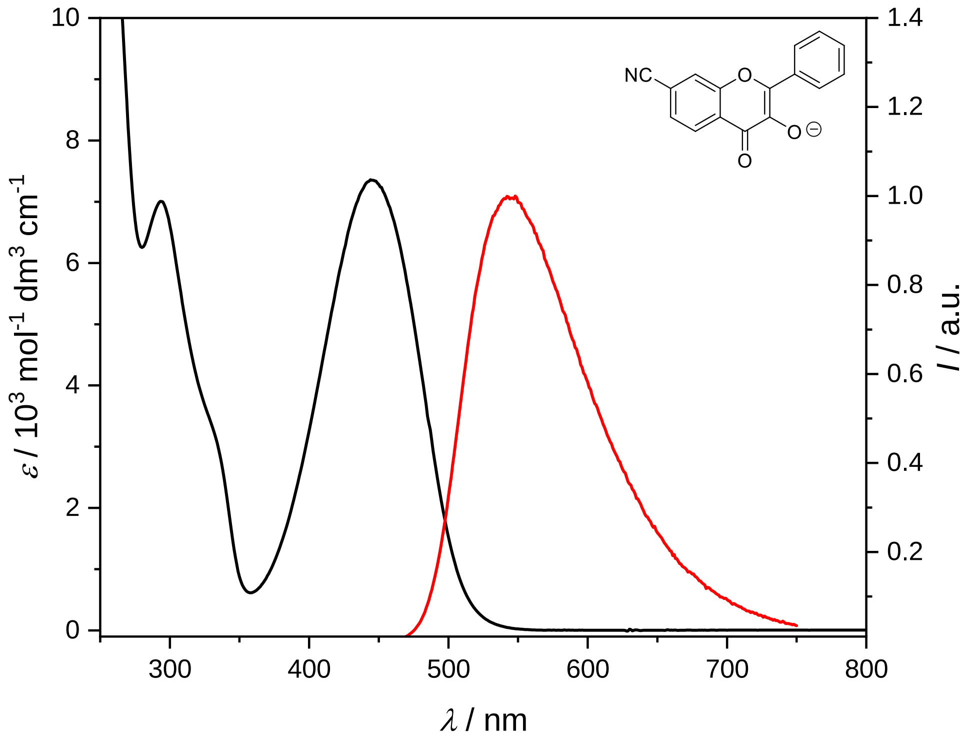

Figure S111. UV-VIS absorption (black line) and normalized emission (red line) of **9B** (*c* ~10^{-5} M, methanol/DMSO, 90:10, ~50 equiv. of NaOH).

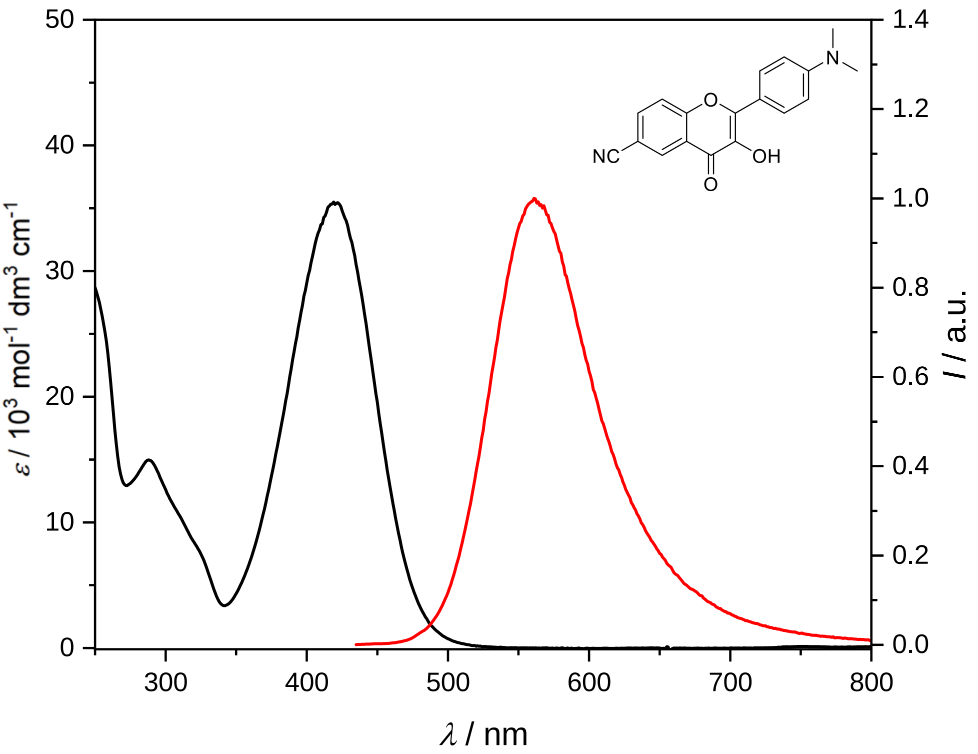

Figure S112. UV-VIS absorption (black line) and normalized emission (red line) of **10A** (c ~10^{-5} M, methanol/DMSO, 98:2).

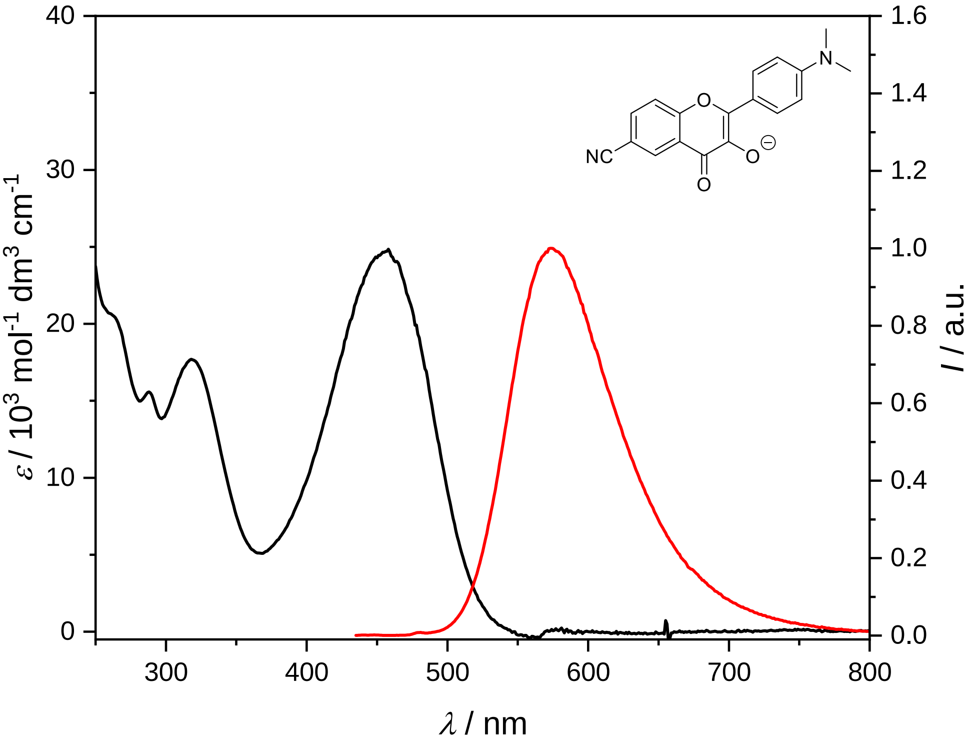

Figure S113. UV-VIS absorption (black line) and normalized emission (red line) of **10B** (c ~10^{-5} M, methanol/DMSO, 98:2, ~200 equiv. of NaOH).

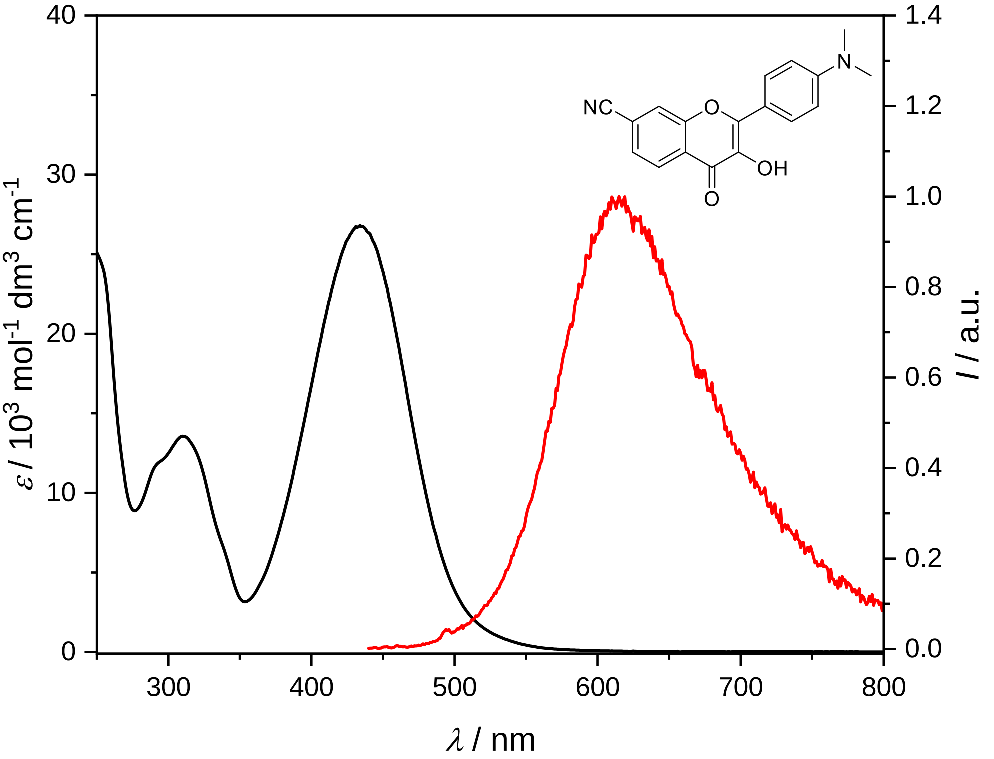

Figure S114. UV-VIS absorption (black line) and normalized emission (red line) of **11A** (c ~10^{-5} M, methanol/DMSO, 90:10).

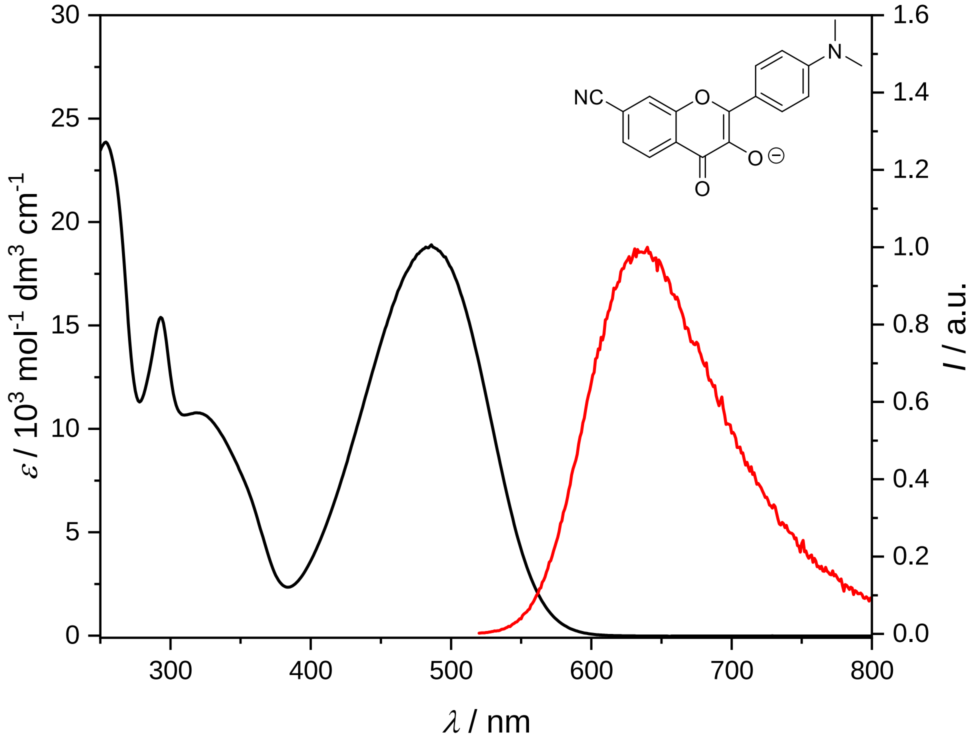

Figure S115. UV-VIS absorption (black line) and normalized emission (red line) of **11B** (c ~10^{-5} M, methanol/DMSO, 90:10, ~400 equiv. of NaOH).

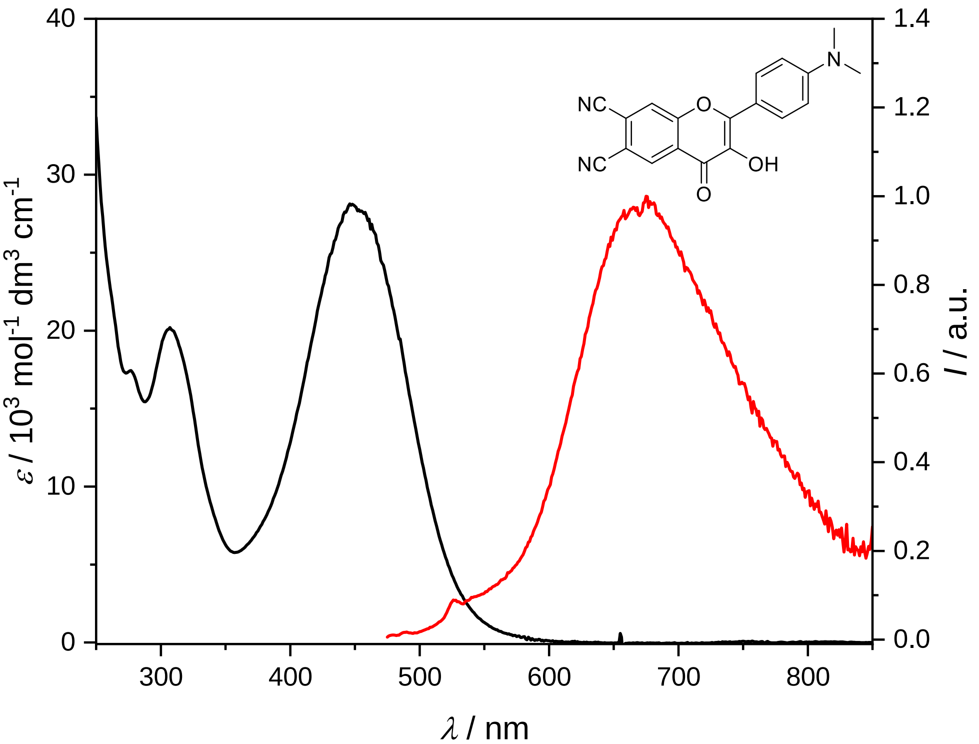

Figure S116. UV-VIS absorption (black line) and normalized emission (red line) of **12A** (c ~10^{-5} M, methanol/DMSO, 98:2).

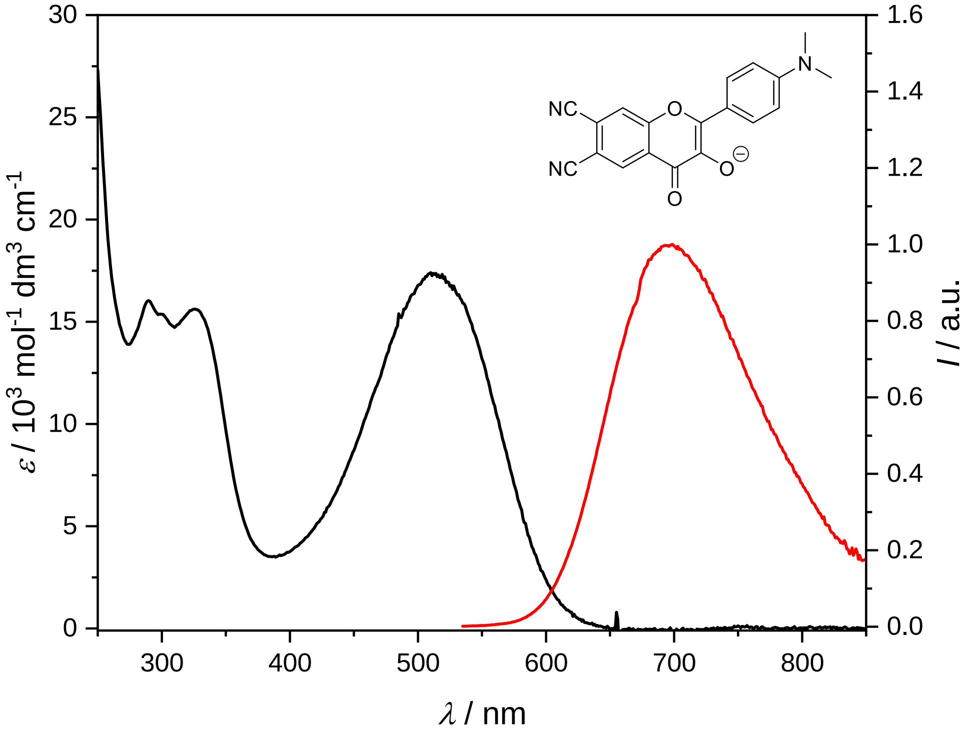

Figure S117. UV-VIS absorption (black line) and normalized emission (red line) of **12B** (c ~10^{-5} M, methanol/DMSO, 98:2, ~200 equiv. of NaOH).

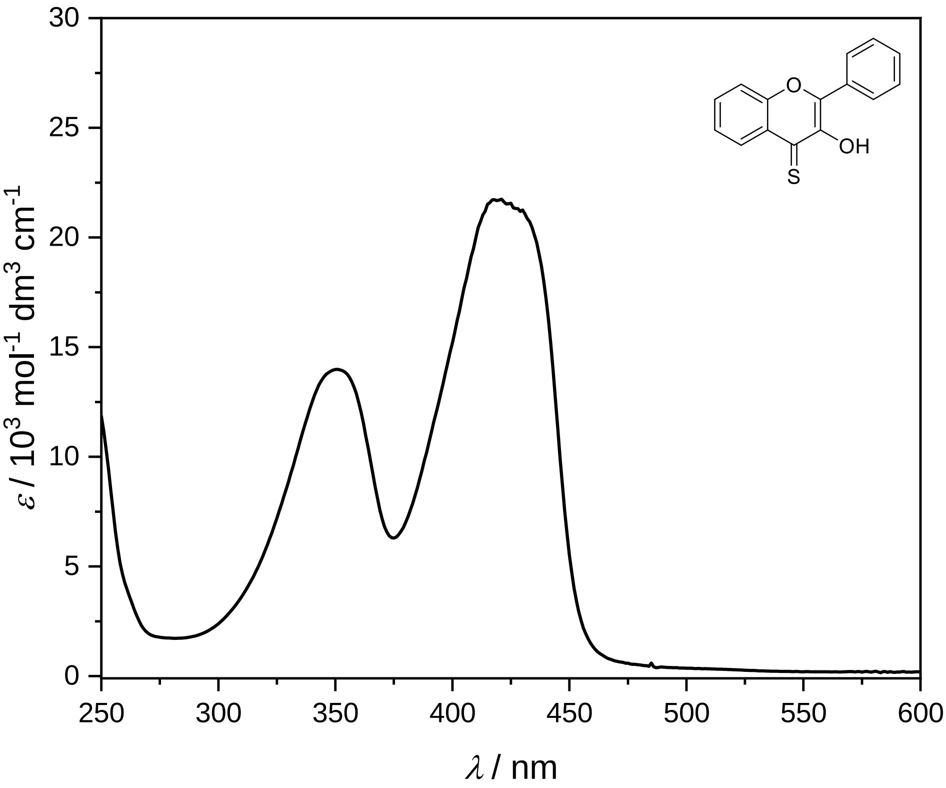

Figure S118. UV-VIS absorption of **13A** (c ~8 × 10^{-5} M, methanol).

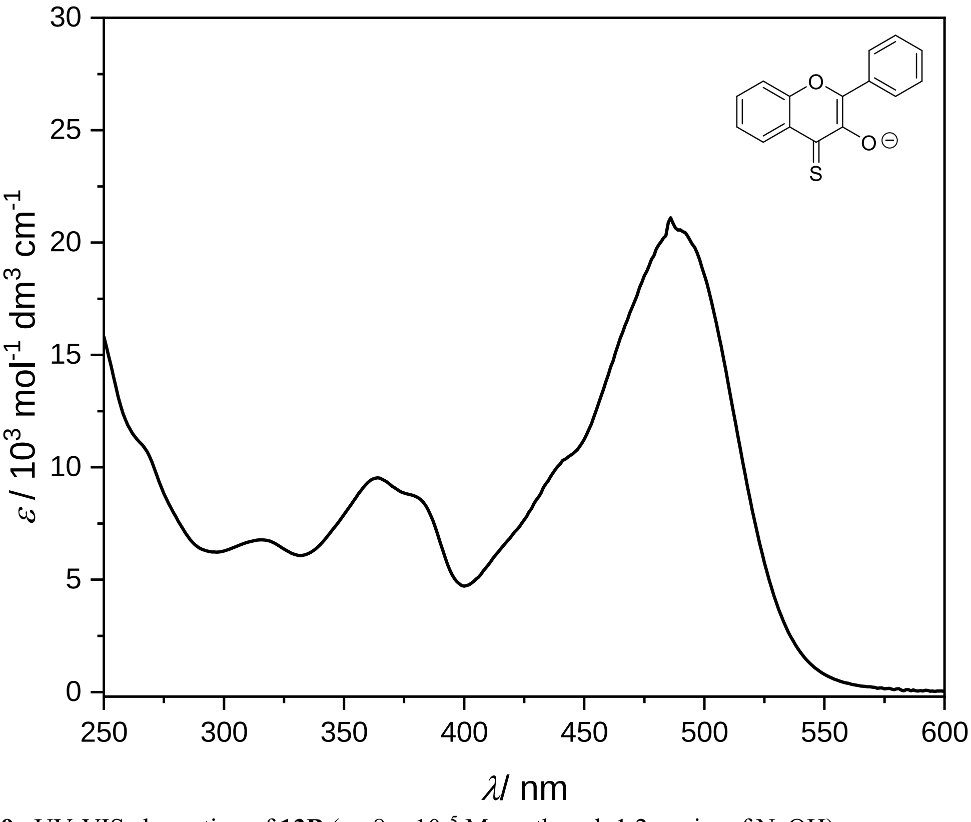

Figure S119. UV-VIS absorption of **13B** (c ~8 × 10^{-5} M, methanol, 1.2 equiv. of NaOH).

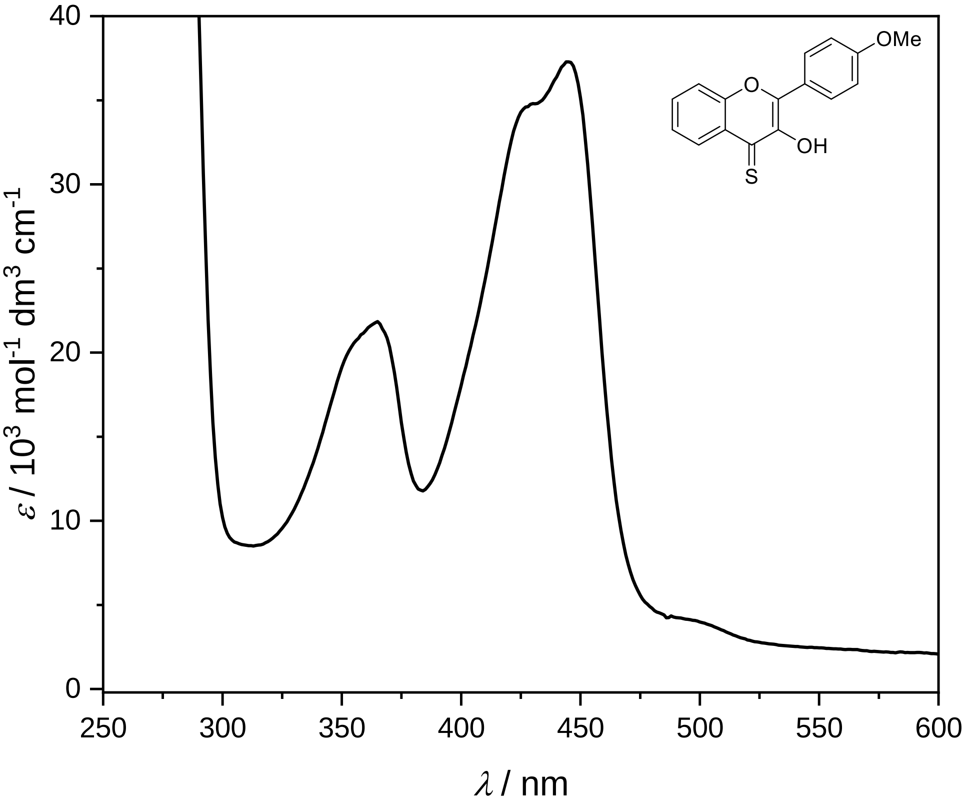

Figure S120. UV-VIS absorption of 1**4A** (c ~10^{-5} M, methanol).

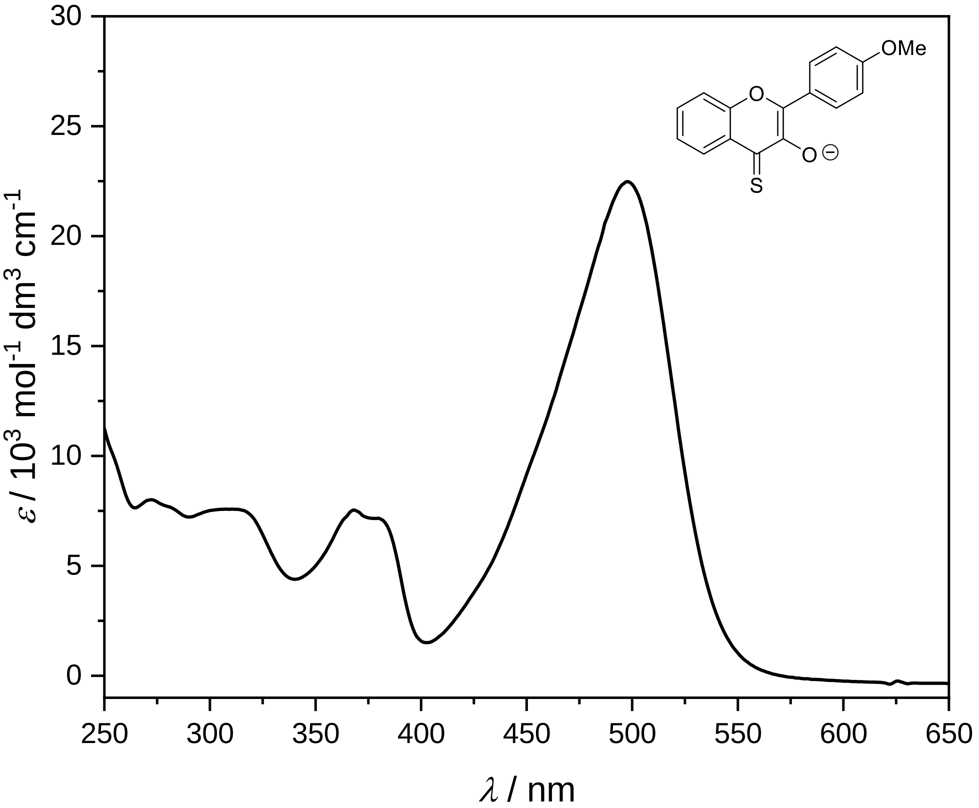

Figure S121. UV-VIS absorption of 1**4B** (c ~3 × 10^{-5} M, methanol, ~200 equiv. of $NaOCH_3$).

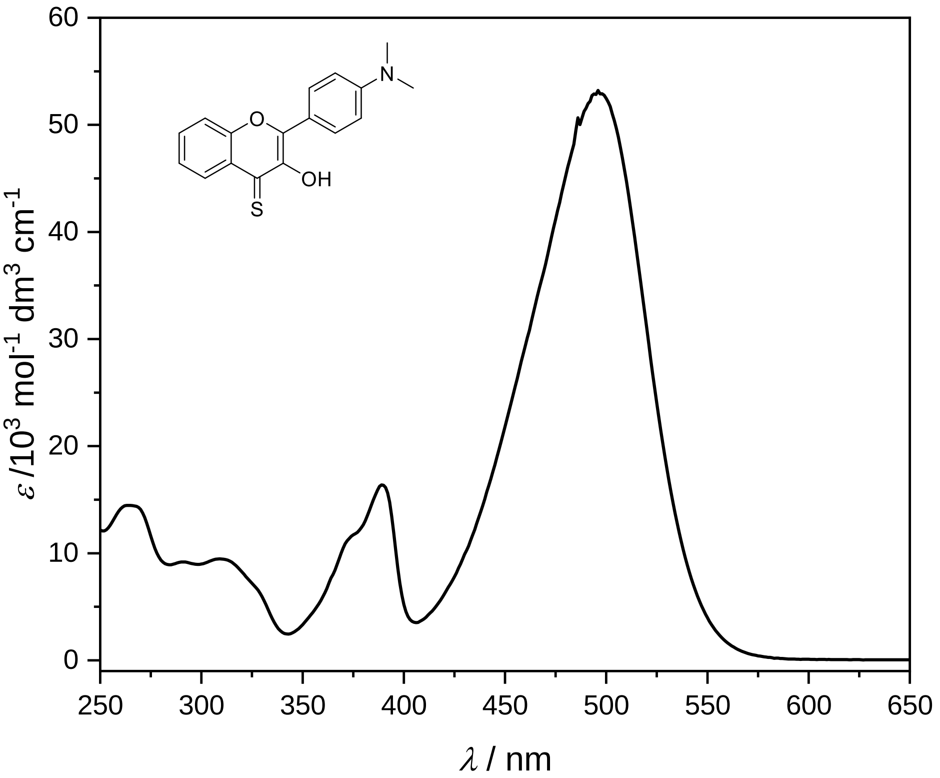

Figure S122. UV-VIS absorption of **15A** (c ~3 × 10^{-5} M, methanol).

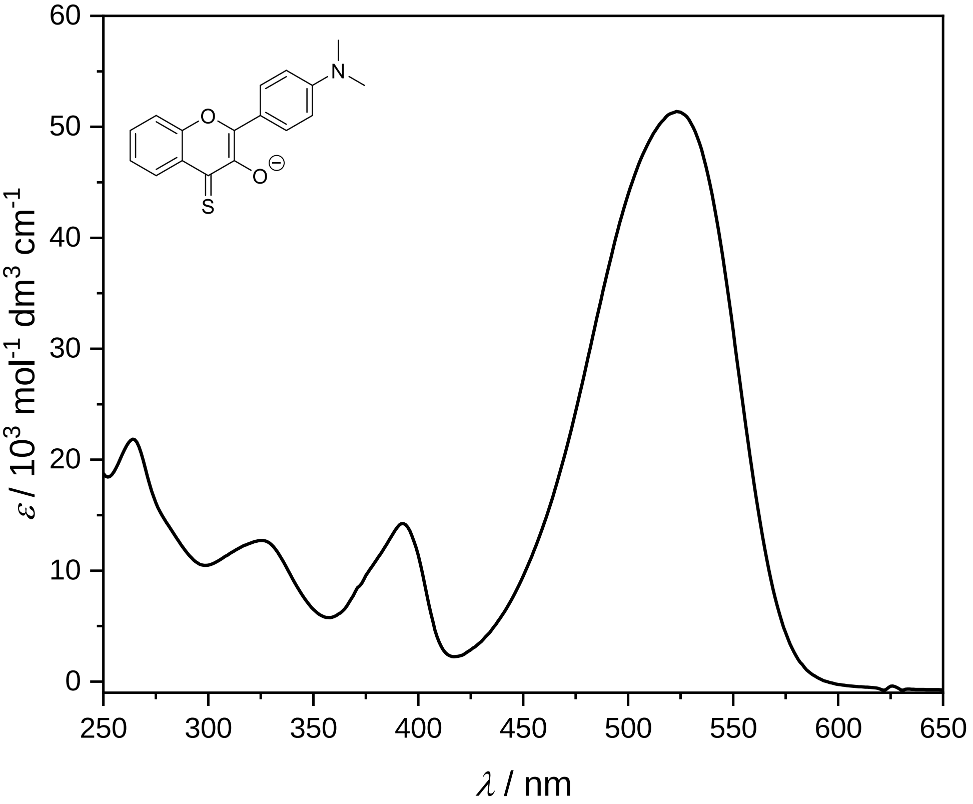

Figure S123. UV-VIS absorption of **15B** (c ~2 × 10^{-5} M, methanol, ~200 equiv. of $NaOCH_3$).

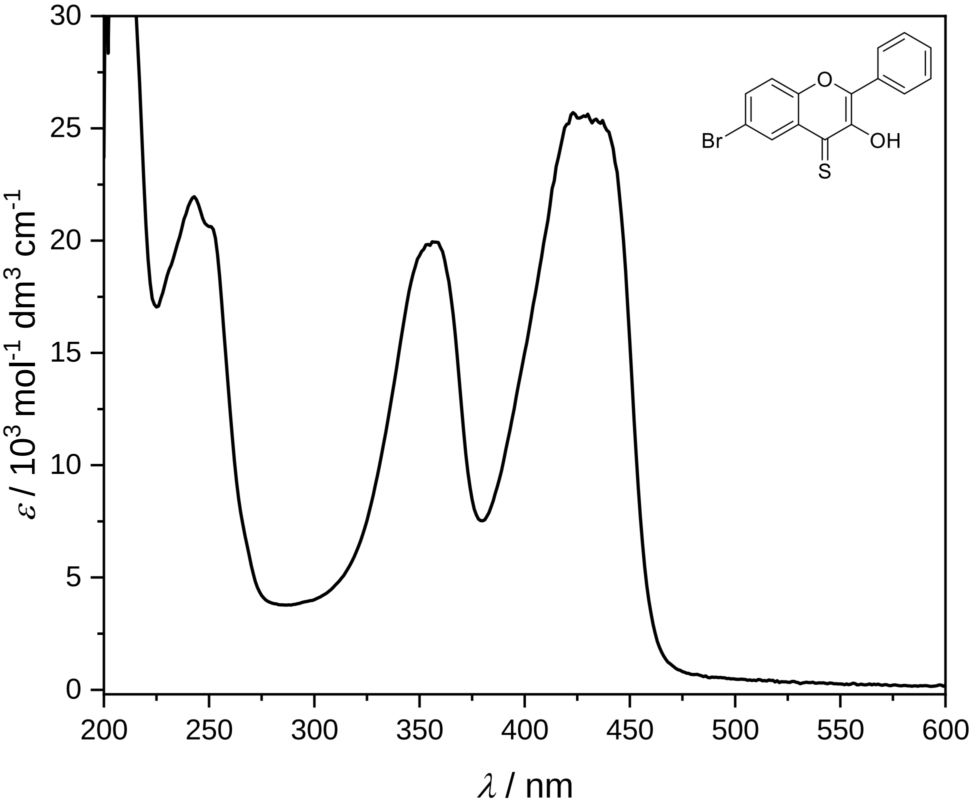

Figure S124. UV-VIS absorption of **16A** (c ~5 × 10^{-5} M, methanol).

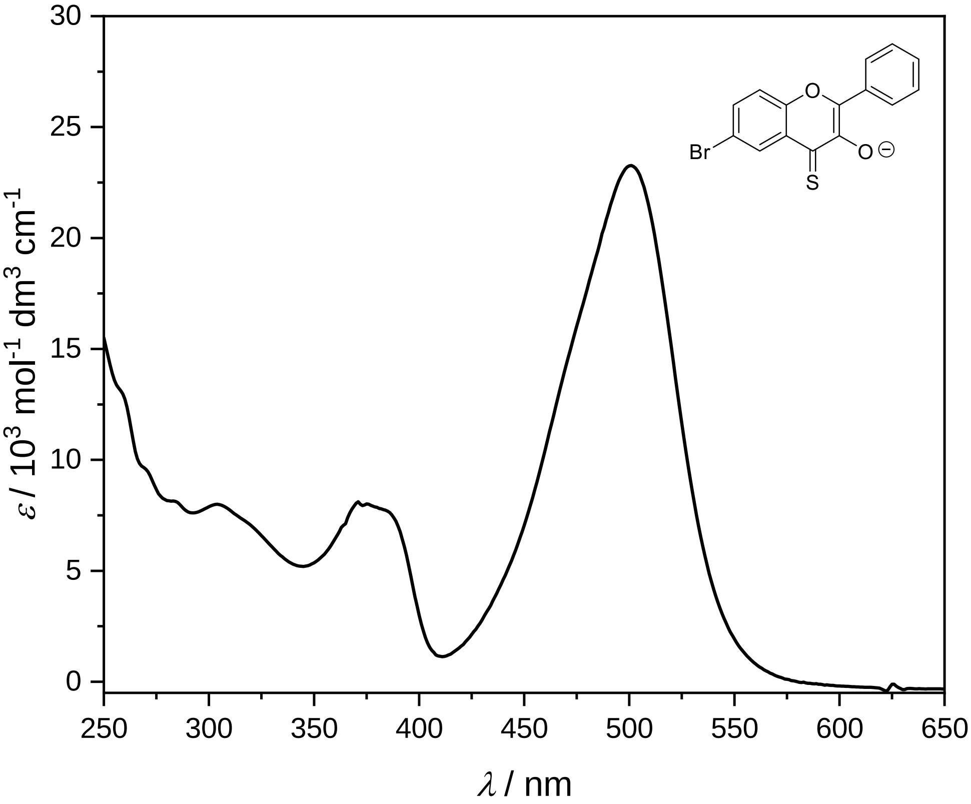

Figure S125. UV-VIS absorption of **16B** (c ~2 × 10^{-5} M, methanol, ~200 equiv. of $NaOCH_3$).

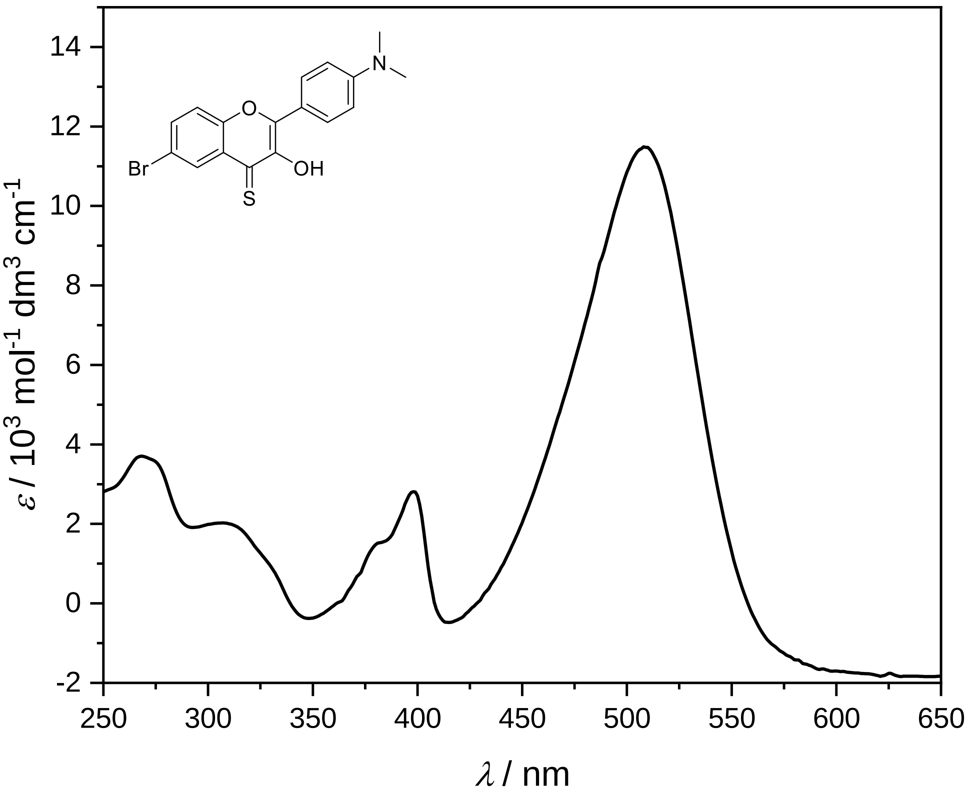

Figure S126. UV-VIS absorption of **17A** (c ~6 × 10^{-5} M, ethanol/DMSO, 98:2).

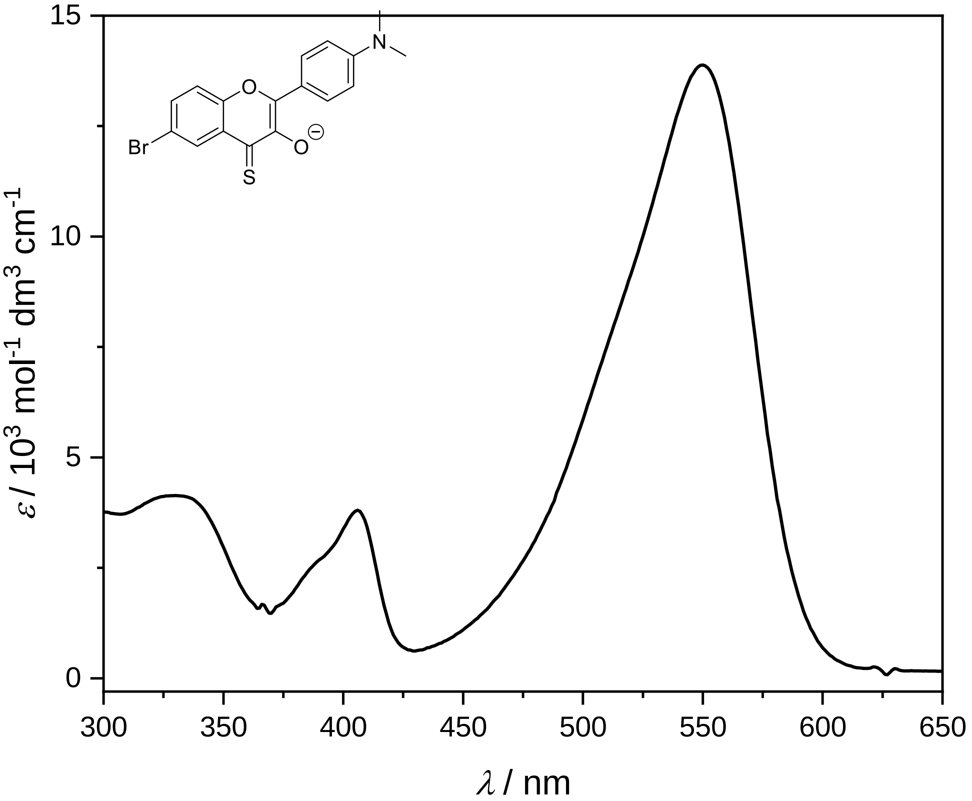

Figure S127. UV-VIS absorption of **17B** (c ~3 × 10^{-5} M, ethanol/DMSO, 98:2, ~200 equiv. of $NaOCH_3$).

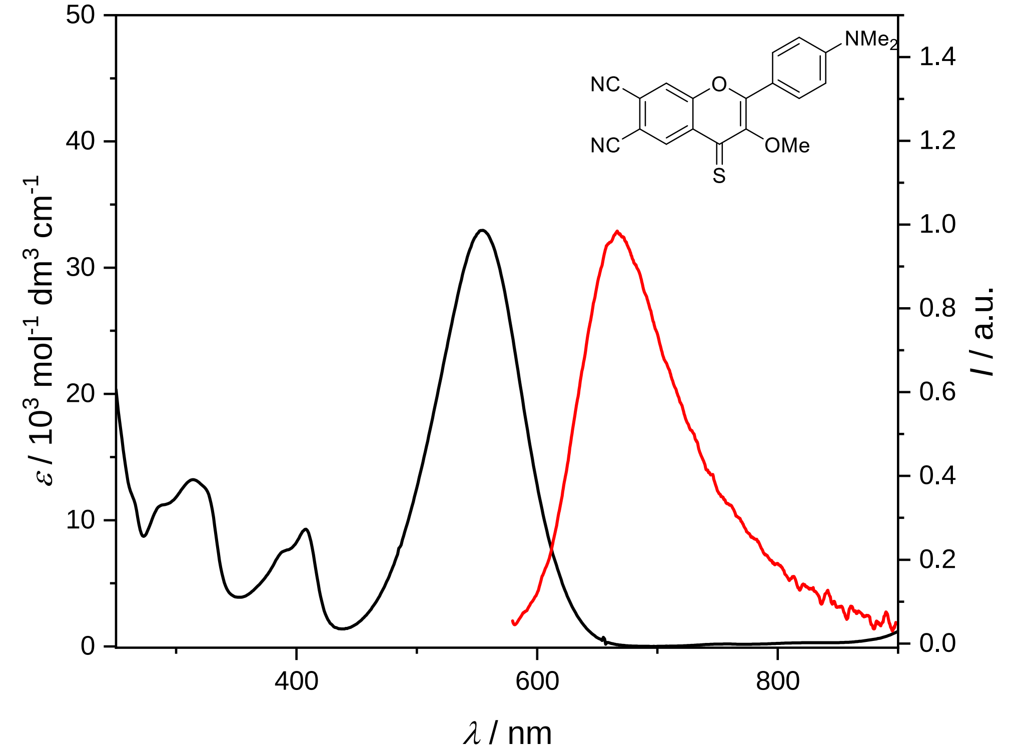

Figure S128. UV-VIS absorption (black line) and normalized emission (red line) of **18A** (c ~10^{-5} M, methanol/DMSO, 98:2).

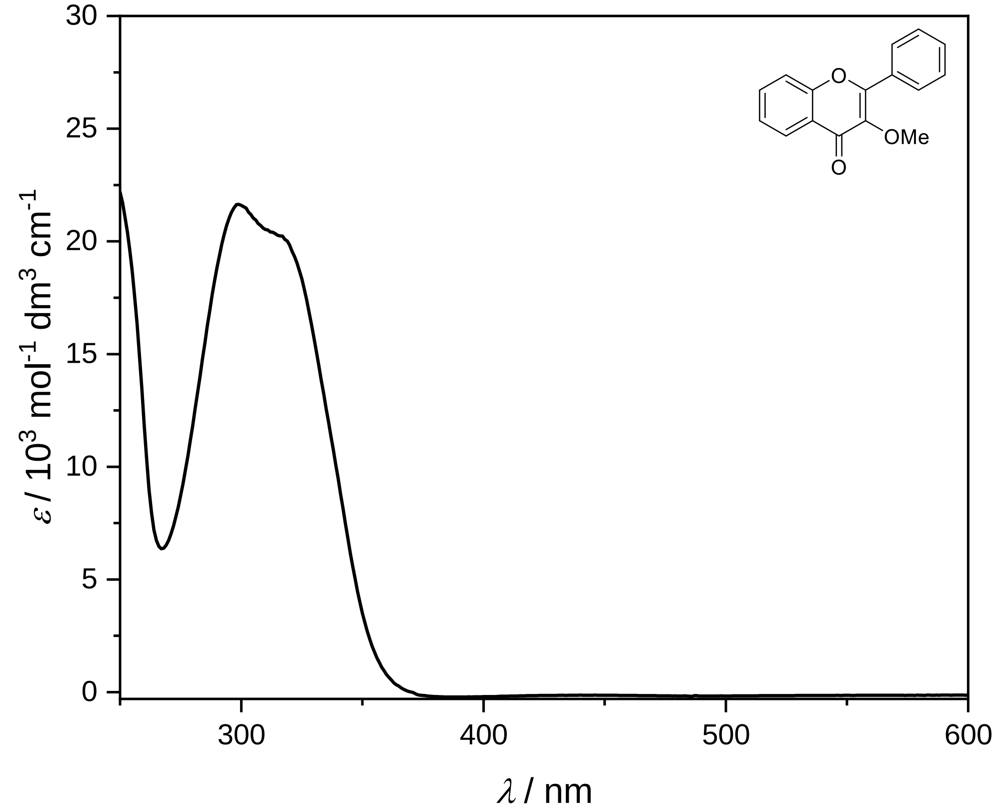

Figure S129. UV-VIS absorption of **19** (c ~9×10^{-5} M, methanol).

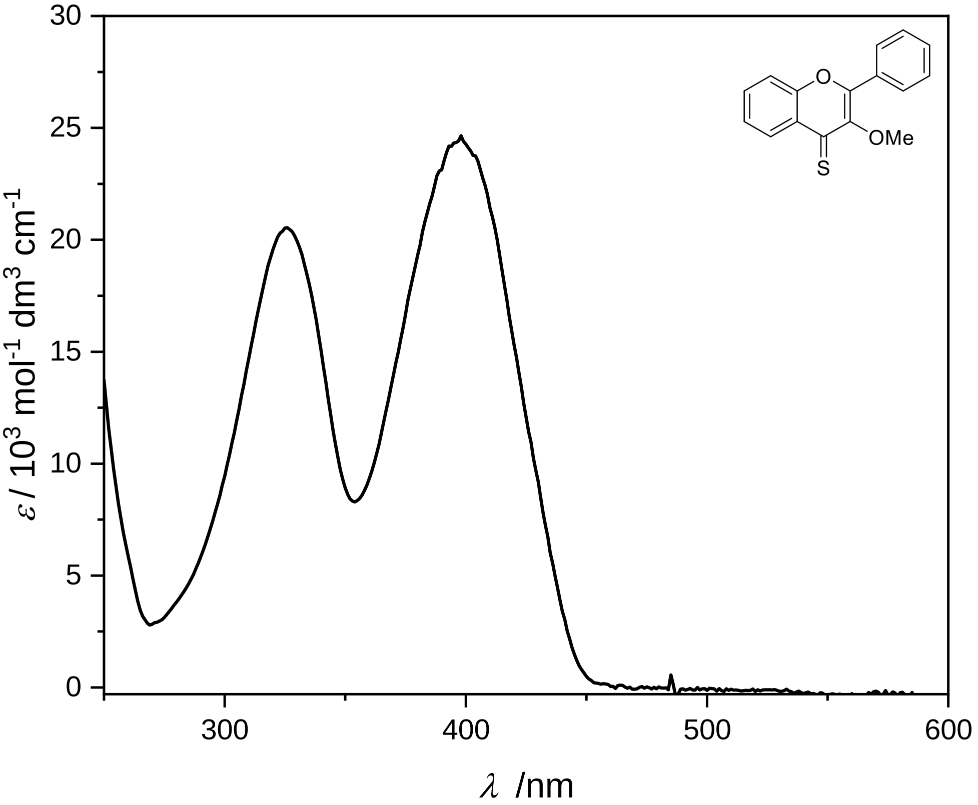

Figure S130. UV-VIS absorption of **20** (c ~4 × 10^{-5} M, methanol).

Experimental Procedures

Material and Methods

Reagents and solvents of the highest purity available were used as purchased, or they were purified/dried when necessary by standard methods. Syntheses were performed under ambient air unless stated otherwise. UV–VIS absorption spectra were measured in a 1.0 cm quartz cuvette, and molar absorption coefficients determined from the absorption spectra were averaged over three independent measurements. HPLC analyses were carried out using a reverse-phase column with a mixture of methanol and Mili-Q® water as an eluent. The exact masses of the synthesized compounds were obtained using a TOF triple quadrupole ESI and APCI mass spectrometer working in a positive or negative mode coupled with direct inlet or liquid chromatography. NMR spectra were recorded on 300 and 500 MHz spectrometers. All NMR measurements were performed in $CDCl_3$ (7.26 ppm for ^{1}H NMR and 77.16 ppm for ^{13}C NMR), DMSO-d_6 (2.50 ppm for ^{1}H NMR and 39.52 ppm for ^{13}C NMR) or CD_2Cl_2 (5.32 ppm for ^{1}H NMR and 53.84 ppm for ^{13}C NMR). Chemical shifts are reported in ppm relative to tetramethylsilane using residual signals of solvents as internal standards. CO yield determinations were performed using headspace injection in GC with an MS detector in a SIM mode.

Hazards

Caution! Zinc cyanide is a hazardous substance and must be handled with special care. It is toxic by inhalation, ingestion or contact with skin or eyes. Contact with acid produces toxic hydrogen cyanide.

Determination of p*K*as

A freshly prepared solution of a 3-hydroxyflavothione (**13**, c ~3.0 × 10^{-4} M) in water/methanol (1:1, *v/v*) was transferred into a matched 1.0 cm quartz cuvette, and the UV–VIS absorption spectrum was recorded. The solution was basified by the addition of small aliquots of aq. NaOH (0.1 eq; 0.025 M) and the pH and UV–VIS absorption spectra were recorded upon each addition. The pK_a values were determined from 3 experiments, from which the standard deviation was calculated.

Irradiation in UV Cuvettes

A methanol or ethanol solution (3.0 mL) of the given compounds in a 1.0 cm quartz cell with a PTFE cap equipped with a stirring bar was irradiated with an array of 28 LEDs (λ_{irr} from 355 to 545 nm). The course of the photoreaction was monitored by UV–VIS spectroscopy using a diode-array spectrophotometer at the given time intervals. Three freeze-pump-thaw cycles were performed to prepare degassed samples. Kinetic traces were fitted with single or multiple exponential decay functions.

Stability of the Studied Compounds in the Dark

Acid forms of all 3-hydroxyflavone and 3-hydroxyflavothione derivatives were found to be stable in aerated solutions in the dark, kept for several days, and monitored by UV-vis spectroscopy. Base forms of the 3-hydroxyflavone derivatives were found to be stable in aerated solutions in the dark except for **12B**, which decomposed within several hours. However, all tested flavothiones degraded within several hours in basic solutions in the dark.

Fluorescence Measurements

Emission and excitation spectra were measured in the corresponding solvent at concentrations adjusted to keep the absorbance value around 0.1 at the excitation wavelength using a fluorescence spectrometer in 1.0 cm quartz cuvettes. Each sample was measured five times, and the spectra were averaged. Quantum yields of fluorescence were determined as absolute values using an integration sphere as an average of three measurements. Emission and excitation spectra were normalized and corrected by the photomultiplier sensitivity function using correction files supplied by the manufacturer.

Quantum Yield of Decomposition

Absorbance changes at the absorption maxima (ΔA_{max}) and the molar absorption coefficients (ε) at the solution volume (V) were obtained using the Beer-Lambert law. n_p^{Abs} is the total amount of photons absorbed by the sample over time calculated from the incident photon flux q_0 (mol s^{-1}) to establish the following equations

$$n_p^{Abs}(t) = q_0 \int_0^t \int_0^\infty \left(1 - 10^{-A(\lambda,t')}\right) I_{norm}^{em}(\lambda) d\lambda dt' \quad (1)$$

$$q_0 = \frac{I}{N_a hc}\int_0^{\infty}\frac{I_{norm}^{em}(\lambda)}{R(\lambda)}d\lambda \tag{2}$$

where I stands for the current of the photodiode in A, λ is the wavelength in m, N_a is Avogadro constant in mol^{-1}, h the Planck constant in J s, c is the velocity of light, I_{norm}^{em} is the normalized emission spectrum of the irradiation source ($\int_0^{\infty} I_{norm}^{em}\, d\lambda = 1$), and $R(\lambda)$ is the sensitivity of the photodiode dependent on the wavelength (the data provided by the manufacturer).

Φ_{dec} is then given as the slope of the linear fit of the plot of Δn_{dec} (where Δn_{dec} is the number of moles decomposed upon irradiation of the corresponding compound, calculated from the absorbance values at λ_{max}) on the y-axis and n_p^{Abs} on the x-axis.

CO Yields

A methanol (**1**–**4** , **6**–**16**, and **18–20**) or ethanol (**5** and **17**) solution of a flavonol or flavothione derivative (0.2–1.0 mL, c ~5×10^{-5}–10^{-4} mol dm^{-3}) or a solution containing a flavonol derivative (c ~5×10^{-5}) and furfuryl alcohol ($c = 1 \times 10^{-2}$ mol dm^{-3}, ~200 equiv) or NaN_3 ($c = 1 \times 10^{-2}$ mol dm^{-3}, ~200 equiv) as singlet oxygen traps in a sealed GC transparent vial were irradiated by an LED array at wavelengths ranging from 355 to 545 nm (close to the absorption maximum of corresponding derivative) to complete conversion. The amount of released CO was determined with a GC-headspace instrument equipped with a MXT®-Msieve 5A PLOT column (30 m, 0.53 mm ID). The instrument was calibrated with the CO release from a cyclopropenone derivative (6,7-dihydro-4,9-dimethoxy-1*H*-dibenzo[*a*,*e*]cyclopropa[*c*]cycloocten-1-one)[1] solution (25-800 mL, c ~1.0 10^{-4} mol dm^{-3}, irradiated by a xenon lamp). The reported values are an average of 3–10 measurements.

Quantum Yields of CO Release

A solution of the studied flavonol in methanol/DMSO (98:2 or 90:10) at the given volume (400 µL or 1000 µL) in a sealed vial fitted with a PTFE septum was irradiated by a xenon short-arc lamp through a monochromator set at the corresponding excitation wavelength. Samples were irradiated through the bottom of the vial for the given time so that the conversion did not exceed 10%. The photon flux was measured as an absolute value by a calibrated Si-photodiode. The molar amount of CO released during the irradiation was determined by GC-headspace, and the quantum yield was determined as the absolute value.

Determination of Rate Constants for the Reaction with $^1O_2$

A methanol solution (3.0 mL) of 1,3-diphenylisobenzofuran (DPBF, $c = 5.0 \times 10^{-5}$ M) or a flavonol derivative ($c \sim 1 \times 10^{-5}$ M) with rose bengal (RB; $c = 1.0 \times 10^{-5}$ M) or methylene blue (c = 1.5×10^{-5} M) as singlet oxygen sensitizers was irradiated in a quartz cell (1.0 cm) with LEDs at 572 (RB) or 650 nm (MB). UV–VIS absorption spectra were recorded at the given time intervals. The DPBF or flavonol derivatives decomposition rate was determined from the decay monitored at the corresponding absorption maxima. The bimolecular reaction rates of the reaction between flavonol derivatives and $^1O_2$ were determined using the rate constant of singlet oxygen quenching in methanol ($k_d = 9 \times 10^4$ s^{-1}) and the rate constant of the reaction between DPBF reaction and $^1O_2$ ($k_r = 1.2 \times 10^9$ M^{-1} s^{-1}).[2]

Reaction with Singlet Oxygen

The reaction with singlet oxygen was studied using naphthalene 1,4-endoperoxide, which can produce singlet oxygen upon thermal decomposition.[3] A solution of the corresponding flavonol or flavothione derivative (**1**–**5**, **13**; c ~1×10^{-4} mol dm^{-3}) and naphthalene 1,4-endoperoxide (~100 equiv) in methanol or ethanol in a UV quartz cuvette, placed in a thermostat at 25 °C and kept in the dark, equipped with a stirring bar was monitored by UV–VIS spectroscopy at given time intervals.

Quantum Yields of Singlet Oxygen Production

A methanol/DMSO (98:2) solution of DPBF ($c = 5.0 \times 10^{-5}$ M) with a flavonol derivative (**7B**, **12A** or **12B**, $c = 3.0 \times 10^{-5}$ M) or rose bengal ($c = 1.0 \times 10^{-5}$ M) as a singlet oxygen sensitizer were irradiated in a quartz cuvette (1 cm) equipped with a stirring bar was irradiated by LEDs at 528 nm up to 10% conversion of DPBF. The UV–VIS spectra were recorded at the given time intervals, and the decomposition of DPBF monitored at the absorption maximum was fitted with a pseudo-first-order rate law. The quantum yield of singlet oxygen production was calculated from that of rose bengal as a reference ($\Phi_\Delta = 0.75$).[4]

A methanol solution of a flavonol derivative (**1A** or **7A**, $c = 5.0$–7.0×10^{-5} M) and furfuryl alcohol (FFA, $c = 5.0 \times 10^{-4}$ M) was irradiated with LEDs at 365 or 440 nm. The amount of consumed FFA was determined using HPLC (a reverse-phase column, a mixture of acetonitrile and miliQ water from 10:90 to 95:5, 1 mL min^{-1} as eluent), and the decomposition of flavonol derivative was followed by UV–VIS spectroscopy. The quantum yields of singlet oxygen were calculated either using Φ_r of **1A** (Table 1) or as an absolute number using a calibrated

Si-photodiode (**7A**) and a bimolecular rate constant of the reaction between FFA and singlet oxygen (k_r = 1.03×10^8 M^{-1} s^{-1}).[5]

Nanosecond Transient Spectroscopy

Laser pulses of a ≤170–700 ps duration at 355 nm (~180 mJ) or 532 nm (~ 200 mJ) were obtained from an Nd:YAG laser in a right-angle arrangement with an overpulsed Xe arc lamp as a source of the probe light. The laser beam was dispersed onto a 40×10×10 mm modified quartz cuvette in a laying arrangement. Transient absorption spectra were recorded with an ICCD camera equipped with a spectrograph. Kinetic traces were monitored with a photomultiplier equipped with a monochromator. The signal was fitted with a single exponential decay function. Three freeze-pump-thaw cycles under reduced pressure (~0.05 mbar) were performed for degassed samples. Absorption spectra of samples were regularly recorded during the measurement to monitor the decomposition of the sample.

Emission Spectra in Solvents of Different Polarity

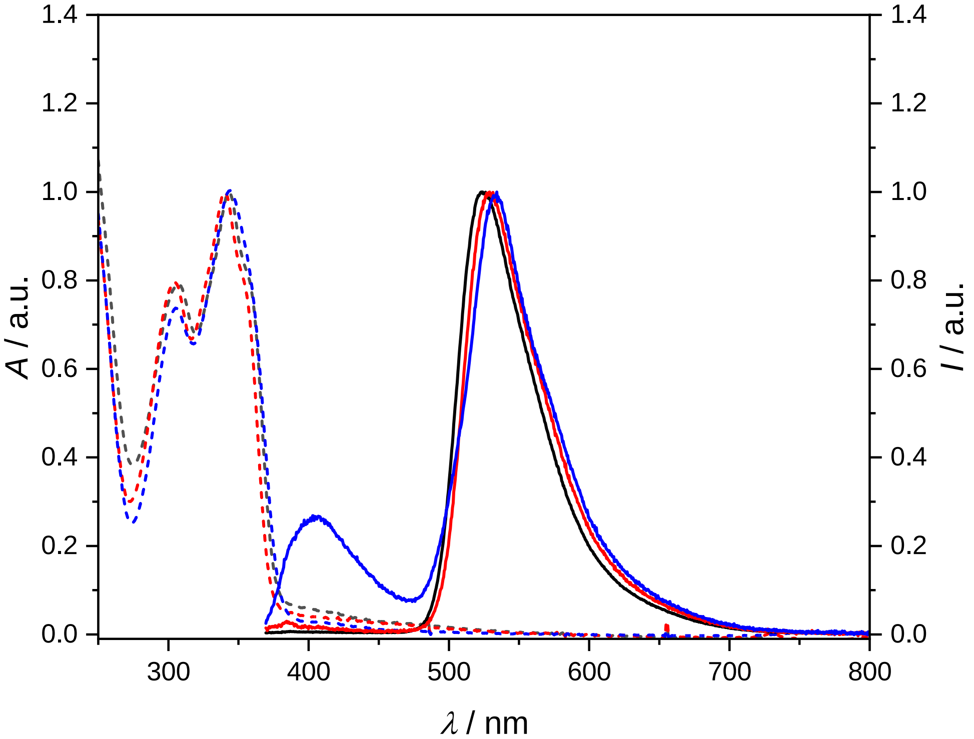

Figure S131. Normalized absorption (dashed line) and normalized emission (solid line) spectra of **1A** acquired in dichloromethane (black), acetonitrile (red), and methanol (blue), c ~10^{-5} M.

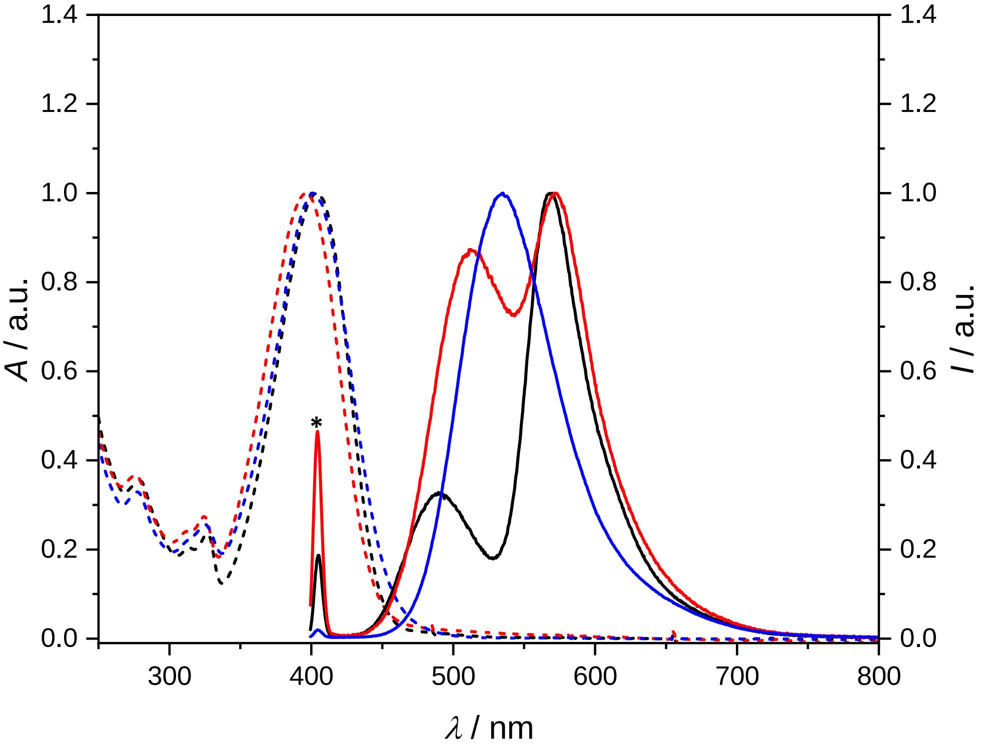

Figure S132. Normalized absorption (dashed line) and normalized emission (solid line) spectra of **3A** acquired in dichloromethane (black), acetonitrile (red), and methanol/DMSO, 98:2 (blue), c ~10^{-5} M. The asterisk denotes emission from the light source.

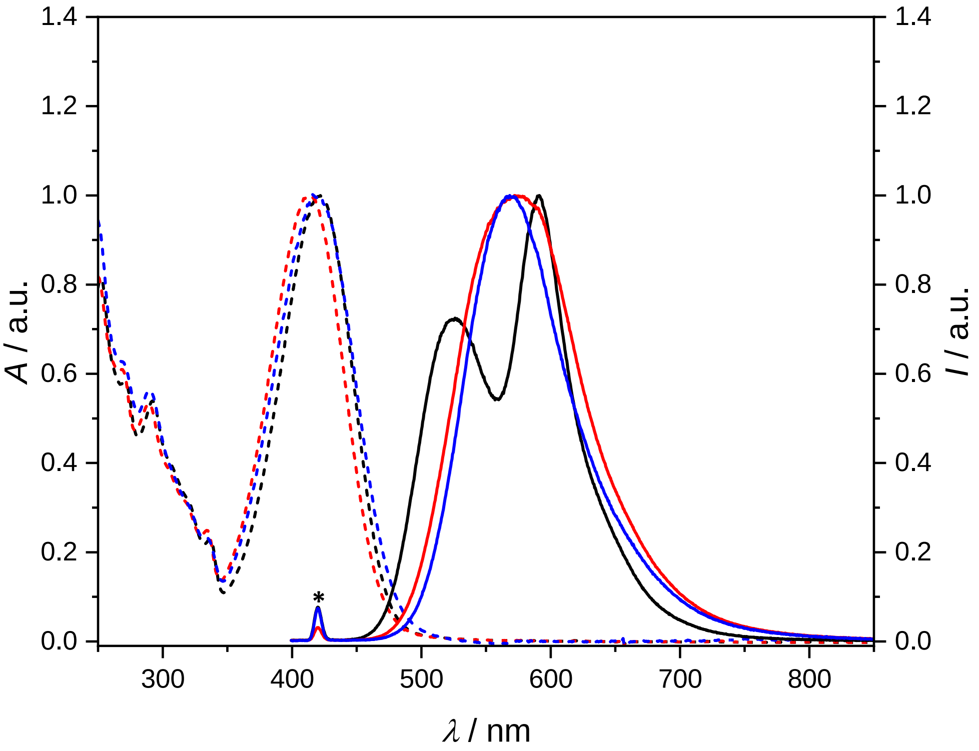

Figure S133. Normalized absorption (dashed line) and normalized emission (solid line) spectra of **7A** acquired in dichloromethane (black), acetonitrile (red), and methanol/DMSO, 98:2 (blue), c ~10^{-5} M. The asterisk denotes emission from the light source.

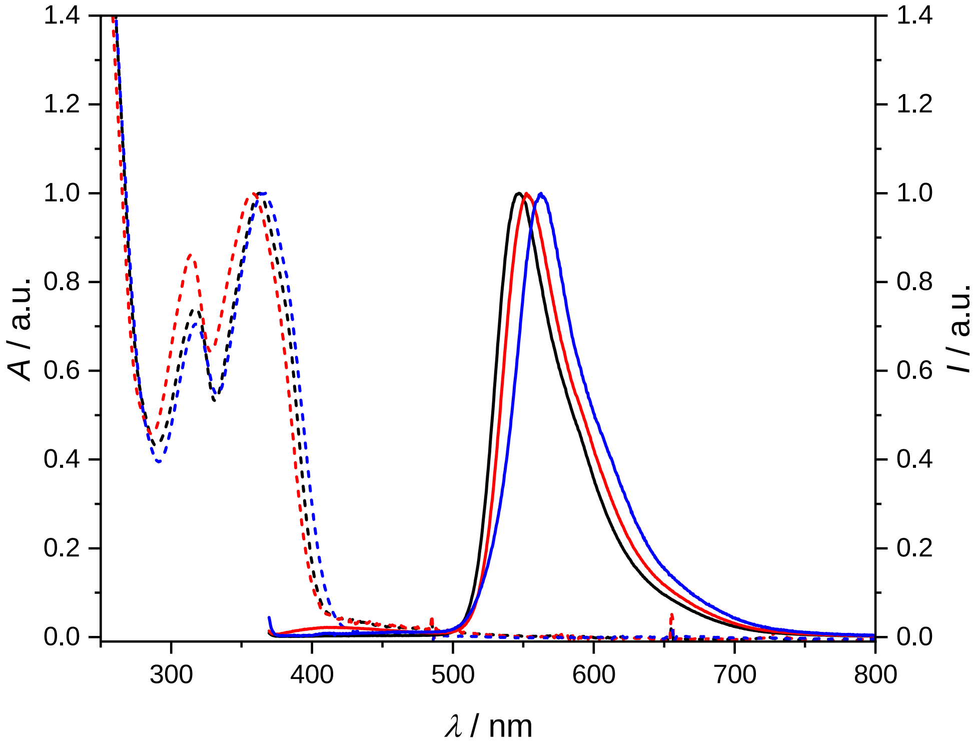

Figure S134. Normalized absorption (dashed line) and normalized emission (solid line) spectra of **9A** acquired in dichloromethane (black), acetonitrile (red), and methanol (blue), c ~10^{-5} M (~50 equiv. of HCl).

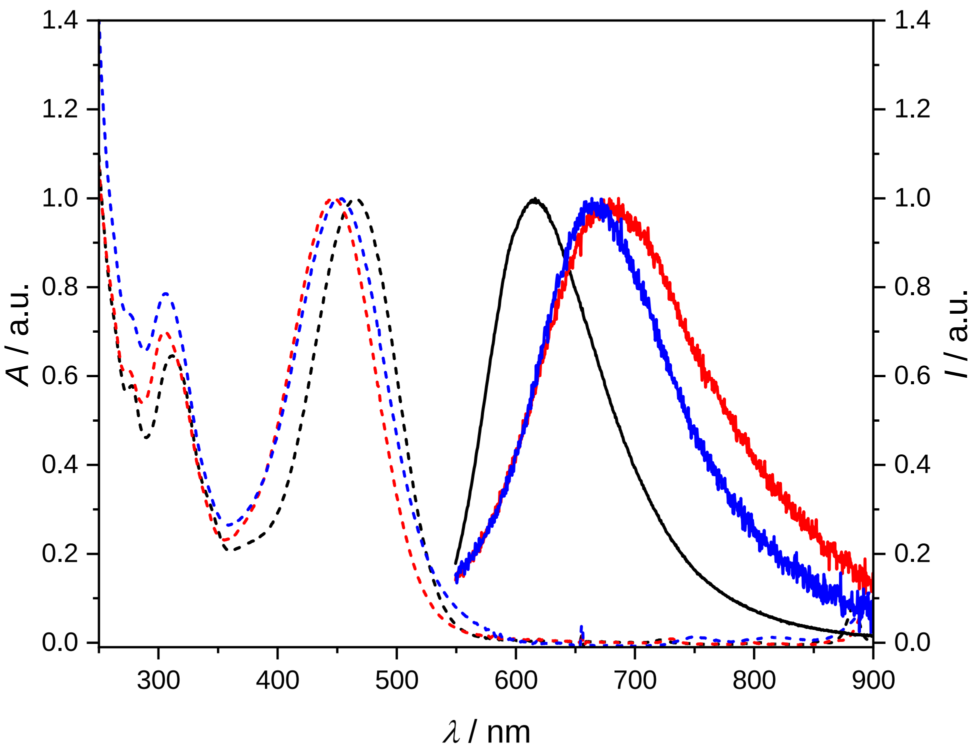

Figure S135. Normalized absorption (dashed line) and normalized emission (solid line) spectra of **12A** acquired in dichloromethane (black), acetonitrile (red), and methanol/DMSO, 98:2 (blue), c ~10^{-5} M. The signals have a low signal-to-noise ratio due to a low intensity of emission in acetonitrile and methanol.

Kinetic of Degradation in Degassed and Aerated Solutions

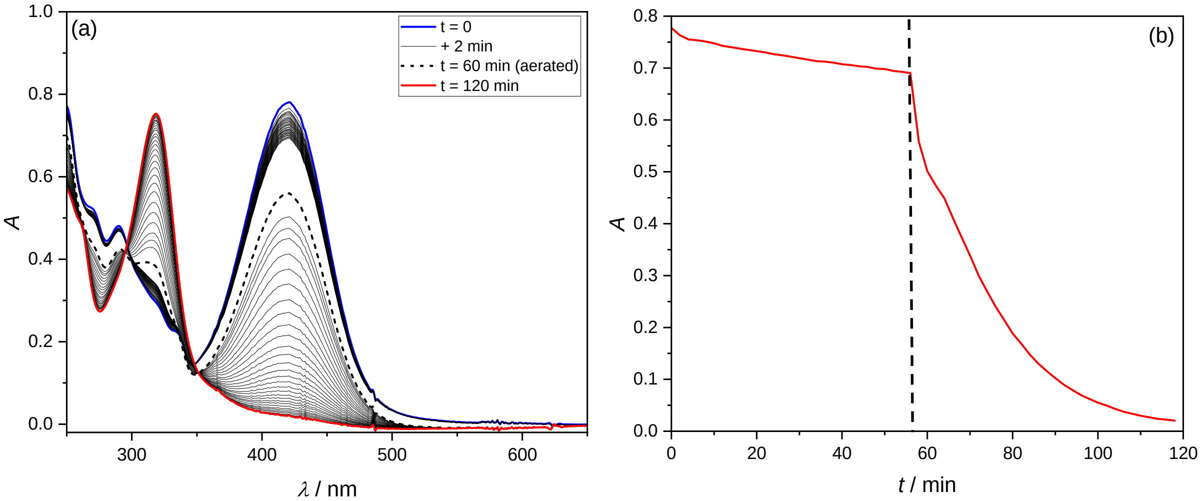

Figure S136. (a) UV-VIS spectra of **7A** (c ~2.5 × 10^{-5} M, methanol/DMSO, 98:2, degassed) irradiated at 440 nm. (b) Traces of the absorbance at 418 nm plotted over time, a dashed line indicates aeration of the measuring cell.

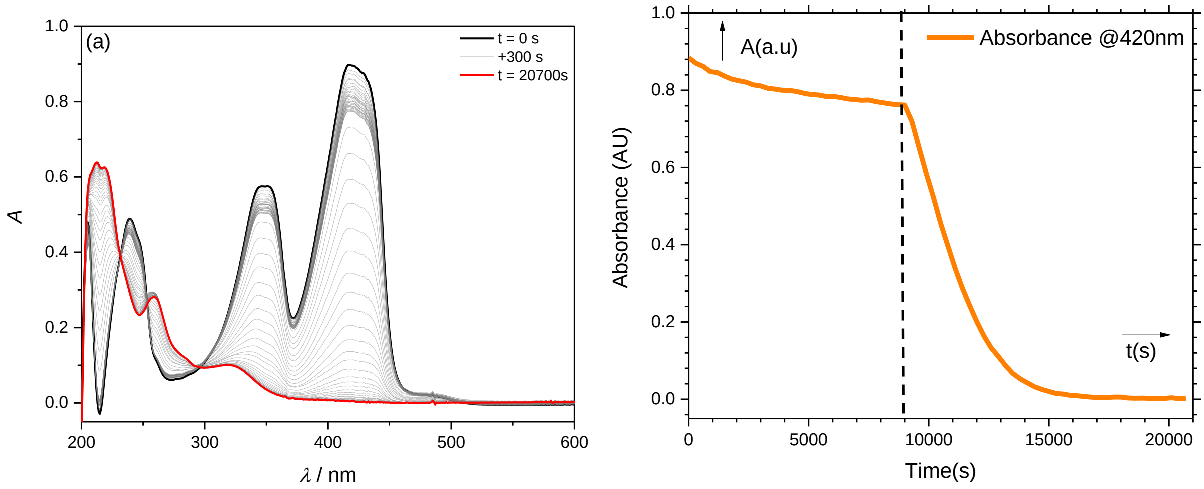

Figure S137. (a) UV-VIS spectra of a methanol solution of **13A** (c ~4.0 × 10^{-5} mol L^{-1}) irradiated by LEDs at λ = 420 nm. Traces of the absorbance at 420 nm are plotted over time (right). The UV cell is aerated at t = 9000 s (dashed lines).

Analysis of Photoproducts

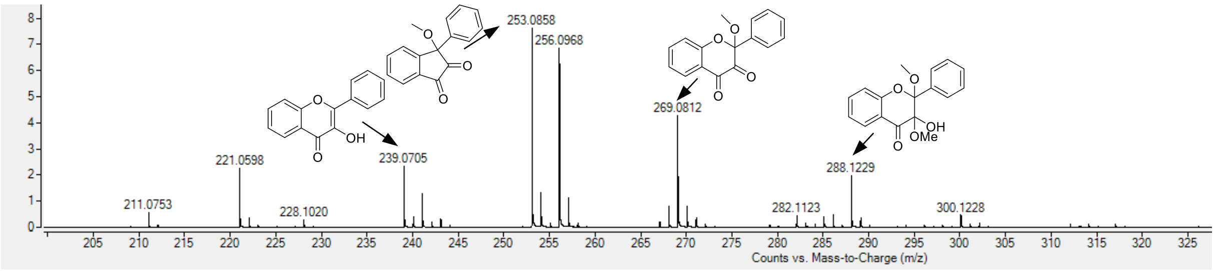

Figure S138. HRMS analysis of photoproducts for **1A** (c ~1 × 10^{-4} M, methanol, aerated) irradiated with 365 nm LEDs. APCI+ (MMI): nitrogen flow 5 L min^{-1}, gas temperature 325 °C, nebulizer 45 psig, skimmer 65 V, vaporizer 200 °C, fragmentor 15 V.

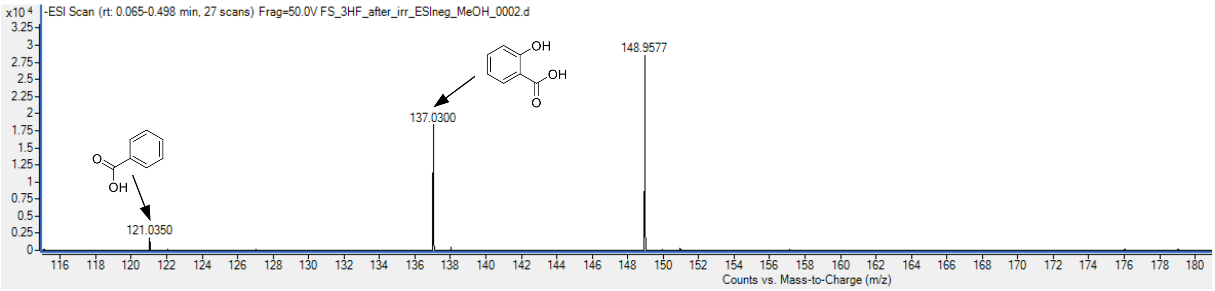

Figure S139. HRMS analysis of photoproducts for **1A** (c ~1 × 10^{-4} M, methanol, aerated) irradiated with 365 nm LEDs. ESI– (MMI): nitrogen flow 5 L min^{-1}, gas temperature 325 °C, nebulizer 45 psig, skimmer –65 V, V_{cap} 2000 V, fragmentor –50 V.

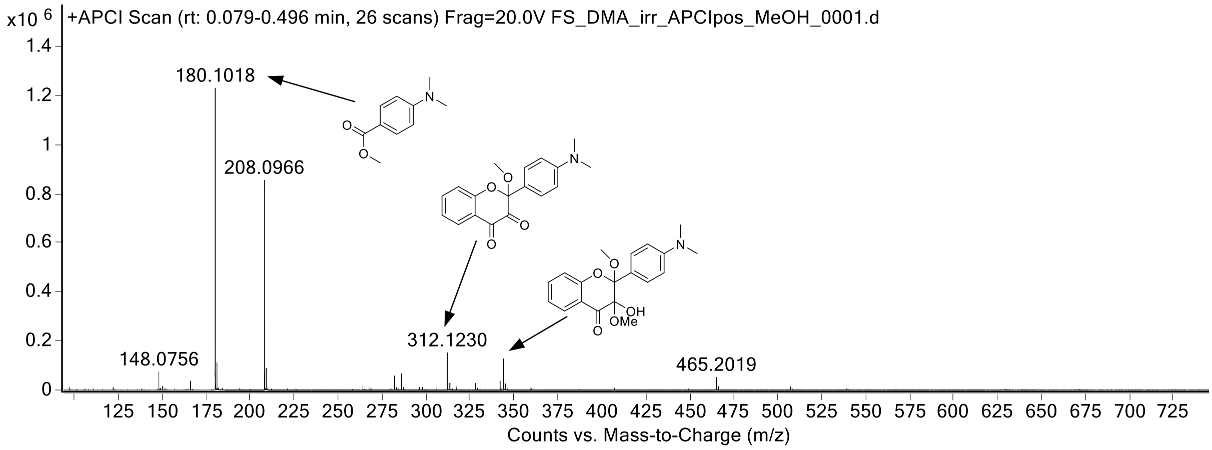

Figure S140. HRMS analysis of photoproducts for **3A** (c ~2 × 10^{-4} M, methanol, aerated) irradiated with 405 nm LEDs. APCI+ (MMI): nitrogen flow 5 L min^{-1}, gas temperature 325 °C, nebulizer 45 psig, skimmer 65 V, vaporizer 200 °C, fragmentor 20 V.

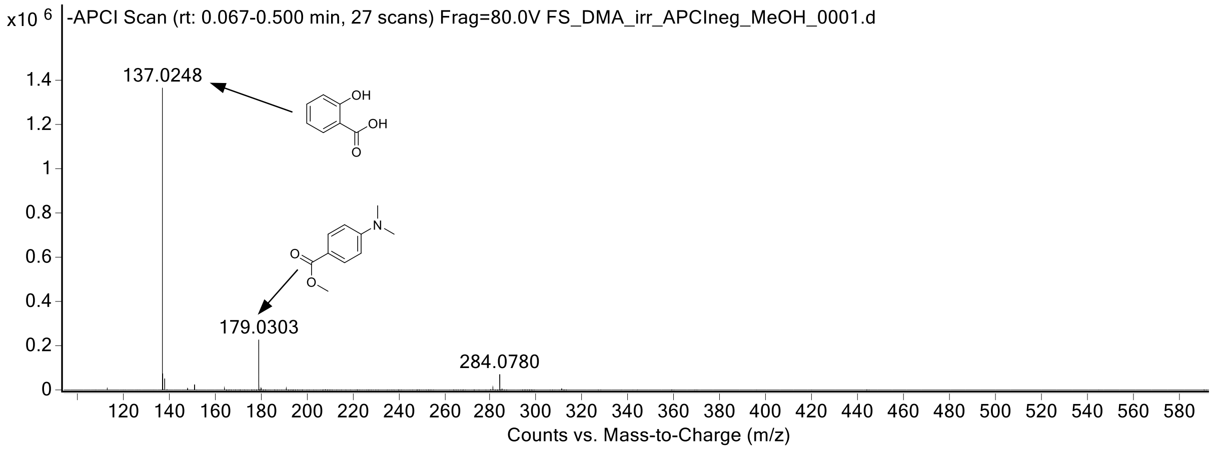

Figure S141. HRMS analysis of photoproducts for **3A** (c ~2 × 10^{-4} M, methanol, aerated) irradiated with 405 nm LEDs. APCI– (MMI): nitrogen flow 5 L min^{-1}, gas temperature 325 °C, nebulizer 45 psig, skimmer –65 V, vaporizer 200 °C, fragmentor –80 V.

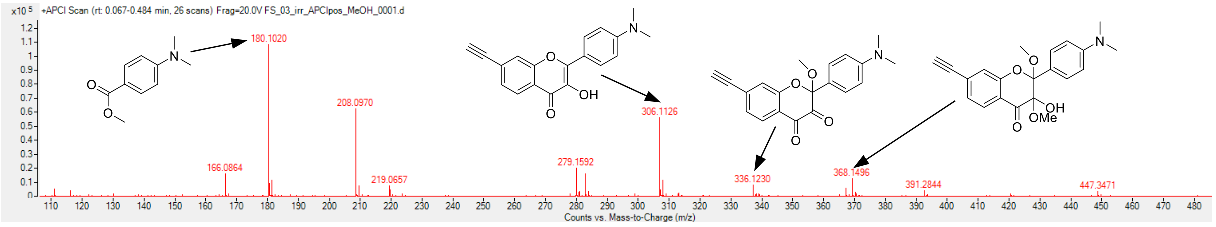

Figure S142. HRMS analysis of photoproducts for **7A** (c ~5 × 10^{-5} M, methanol, aerated) irradiated with 440 nm LEDs. APCI+ (MMI): nitrogen flow 5 L min^{-1}, gas temperature 325 °C, nebulizer 45 psig, skimmer 65 V, vaporizer 200 °C, fragmentor 20 V.

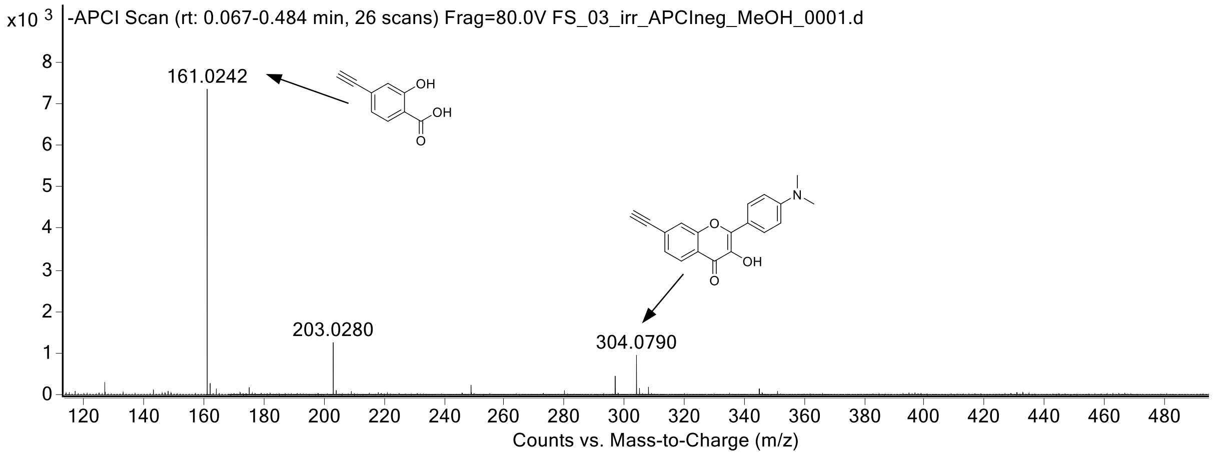

Figure S143. HRMS analysis of photoproducts for 7**A** (c ~5 × 10^{-5} M, methanol, aerated) irradiated with 440 nm LEDs. APCI+ (MMI): nitrogen flow 5 L min^{-1}, gas temperature 325 °C, nebulizer 45 psig, skimmer –65 V, vaporizer 200 °C, fragmentor –80 V.

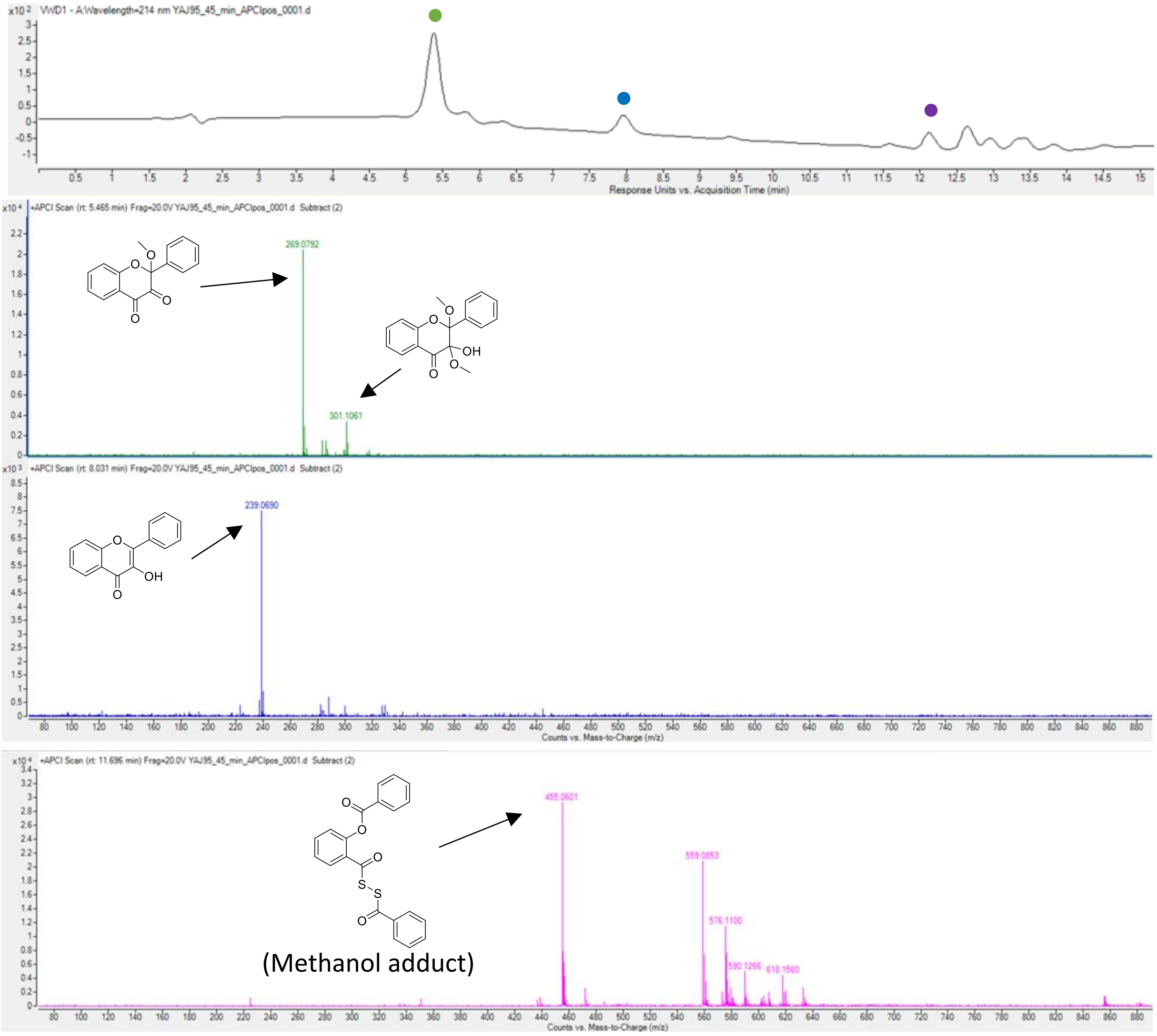

Figure S144. LC/HRMS analysis of the photoproducts for **13A** (c ~1 × 10^{-4} M, methanol, aerated) irradiated with 425 nm LEDs. LC: 20 min gradient water/MeOH (50:50). APCI+ (MMI): nitrogen flow 3 L min^{-1}, gas temperature 325 °C, nebulizer 45 psig, skimmer 65 V, vaporizer 200 °C, fragmentor 20 V.

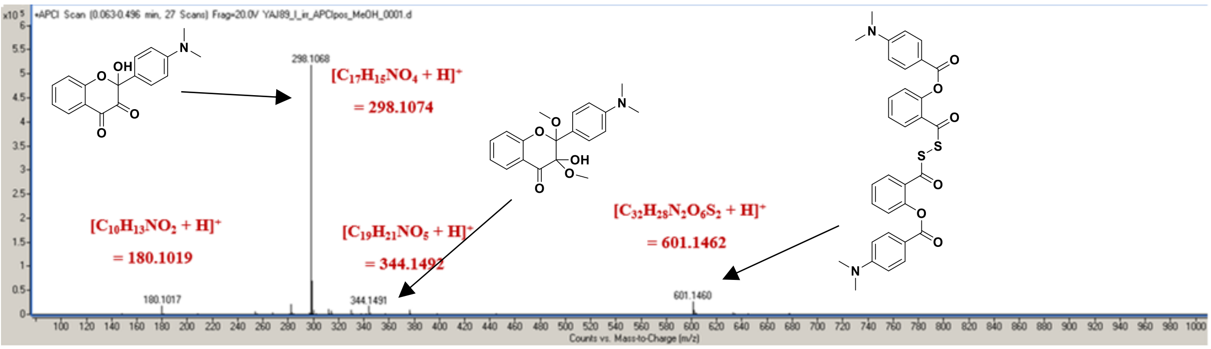

Figure S145. HRMS analysis of photoproducts for **15A** (c ~1 × 10^{-4} M, methanol, aerated) irradiated with 505 nm LEDs. APCI+ (MMI): nitrogen flow 3 L min^{-1}, gas temperature 325 °C, nebulizer 45 psig, skimmer 65 V, vaporizer 200 °C, fragmentor 20 V.

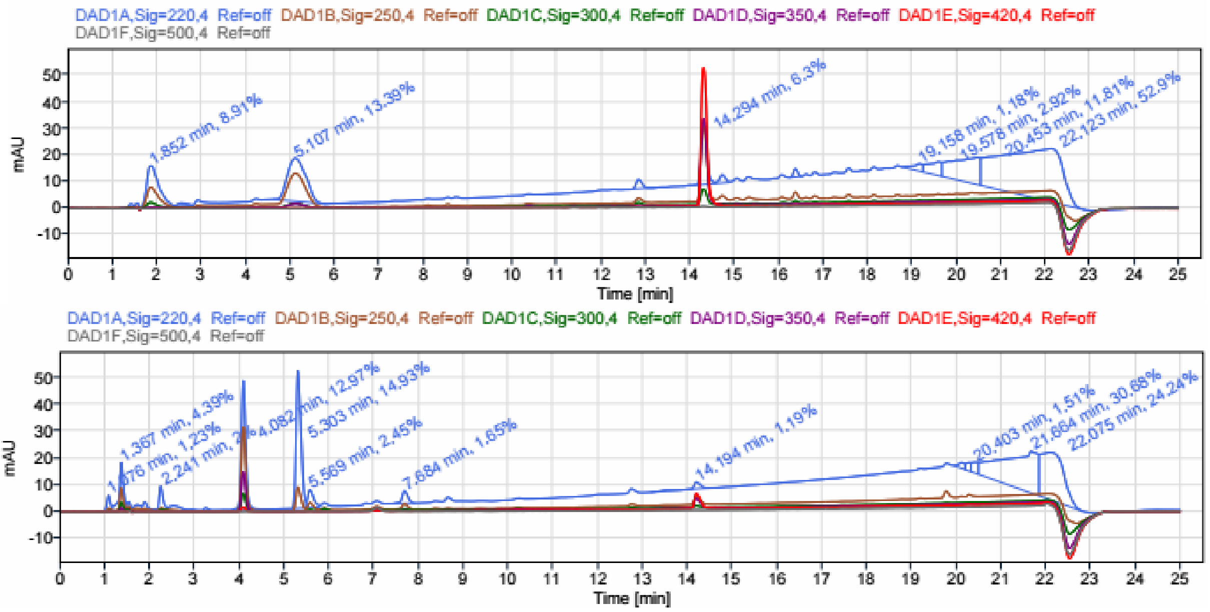

Figure S146. LC chromatograms of the photoproducts of **13A** (up) and **13B** (down), c ~5 × 10^{-5} M. All samples are irradiated by LEDs until reaching ~50% of conversion, at λ = 437 nm (**13A**) and at λ = 490nm (**13B**).

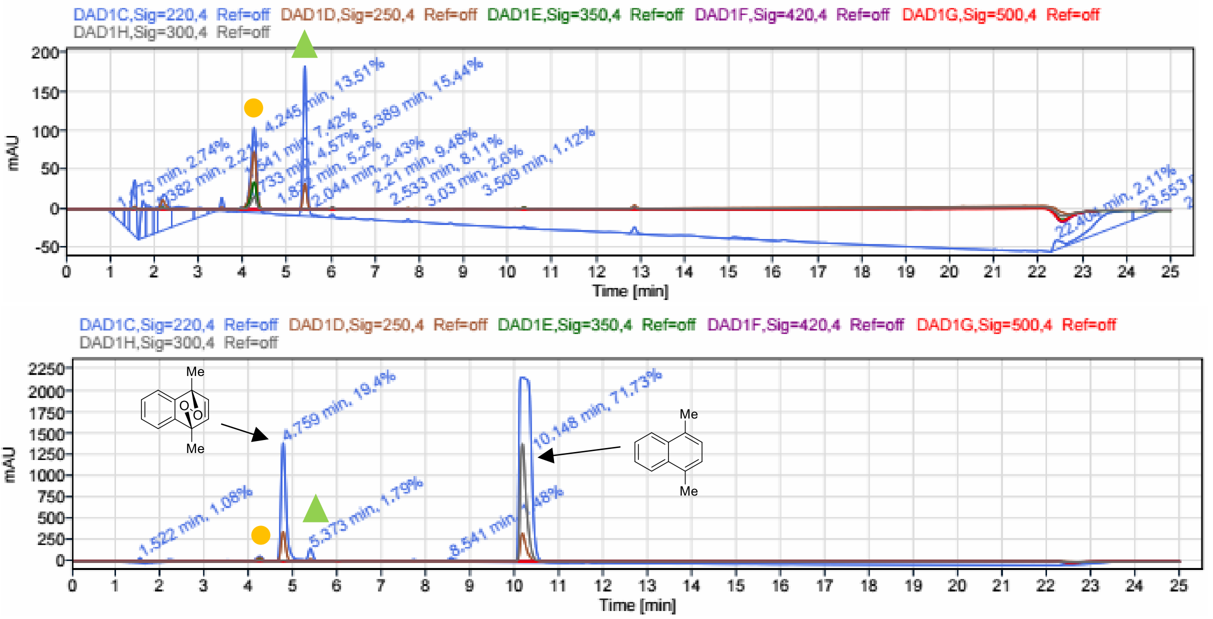

Figure S147. LC chromatograms of **13B** (c ~4 × 10^{-4} M, aerated methanol) from two different experiments: irradiation with LEDs at λ = 490 nm (up) and the reaction in the dark with thermally generated singlet oxygen (T = 25 °C) from naphthalene-1,4-endoperoxide (70 equiv.).

Transient Spectroscopy

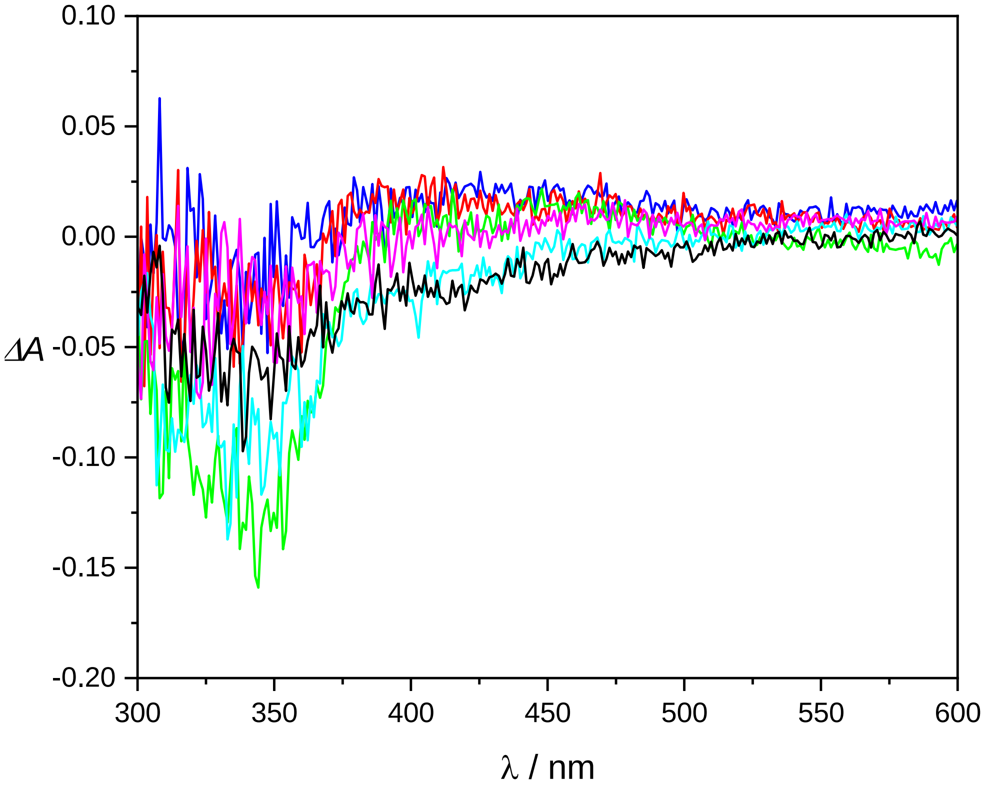

Figure S148. Transient absorption spectra for **1A** (c ~1 × 10^{-5} M, methanol, aerated) taken after a 355 nm flash at various time delays (blue: 10 ns, red: 100 ns, green: 1 µs, cyan: 10 µs, magenta: 100 µs, black: 1 ms).

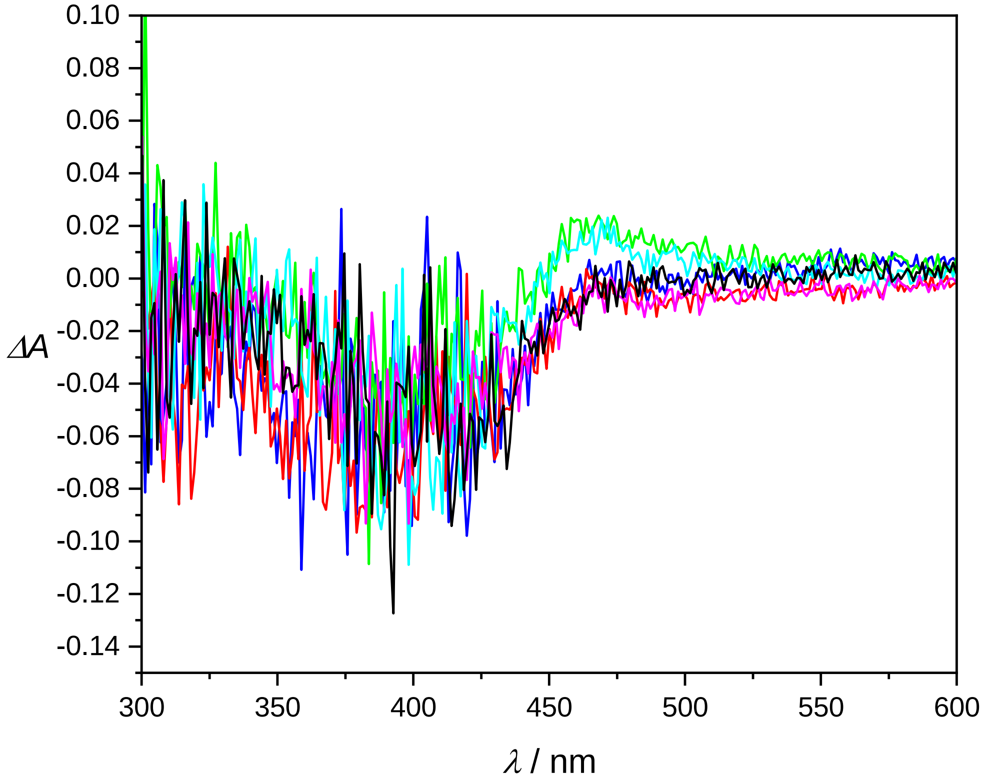

Figure S149. Transient absorption spectra for **1B** (c ~1 × 10^{-5} M, methanol, aerated, ~10 equiv. NaOH) taken after a 355 nm flash at various time delays (blue: 10 ns, red: 100 ns, green: 1 µs, cyan: 10 µs, magenta: 100 µs, black: 1 ms).

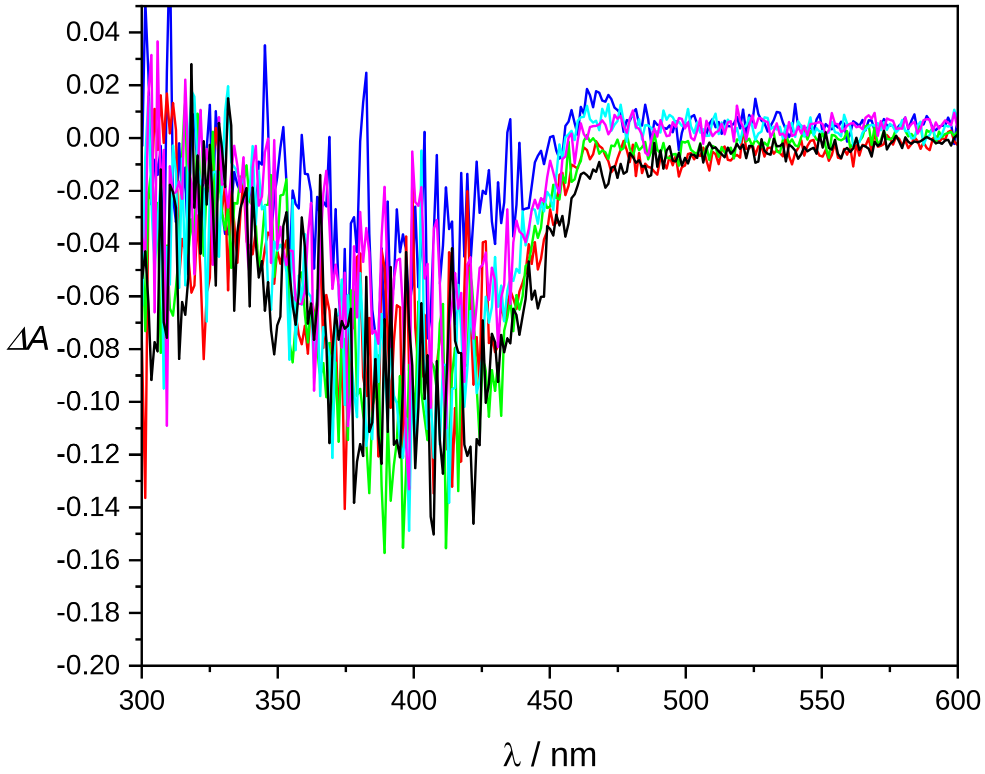

Figure S150. Transient absorption spectra for **1B** (*c* ~1 × 10^{-5} M, methanol, degassed, ~10 equiv. of NaOH) taken after a 355 nm flash at various time delays (blue: 10 ns, red: 100 ns, green: 1 µs, cyan: 10 µs, magenta: 100 µs, black: 1 ms).

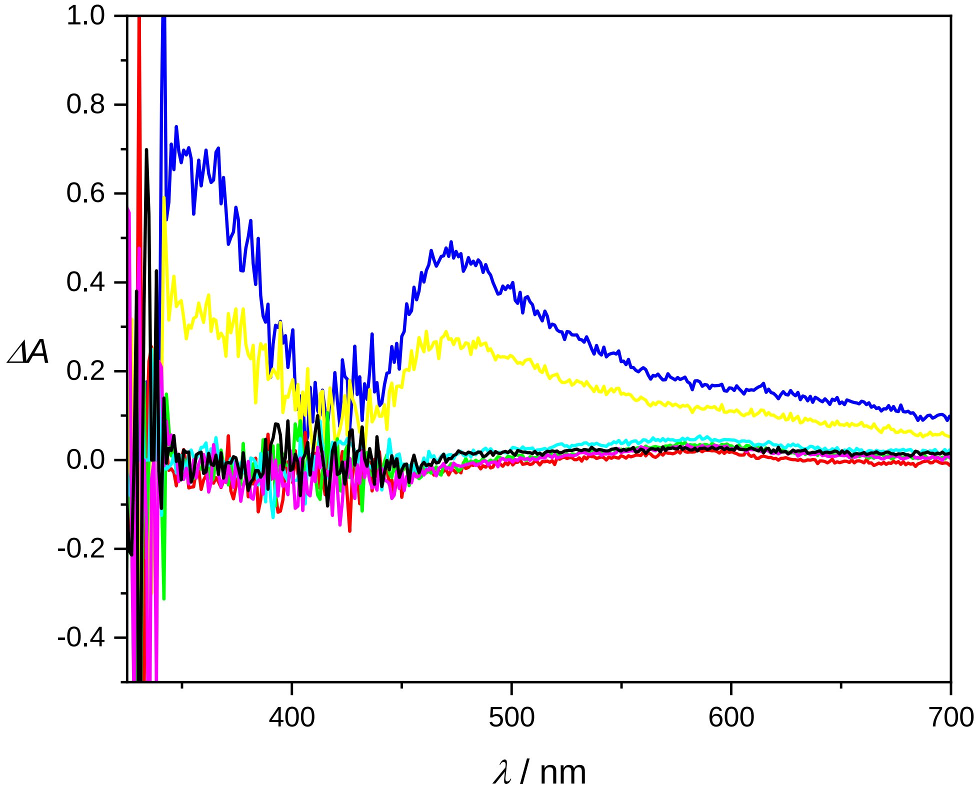

Figure S151. Transient absorption spectra for **7A** (*c* ~2.5 × 10^{-5} M, methanol/DMSO, 98:2, aerated) taken after a 355 nm flash at various time delays (blue: 10 ns, yellow: 50 ns, red: 100 ns, green: 1 µs, cyan: 10 µs, magenta: 100 µs, black: 1 ms).

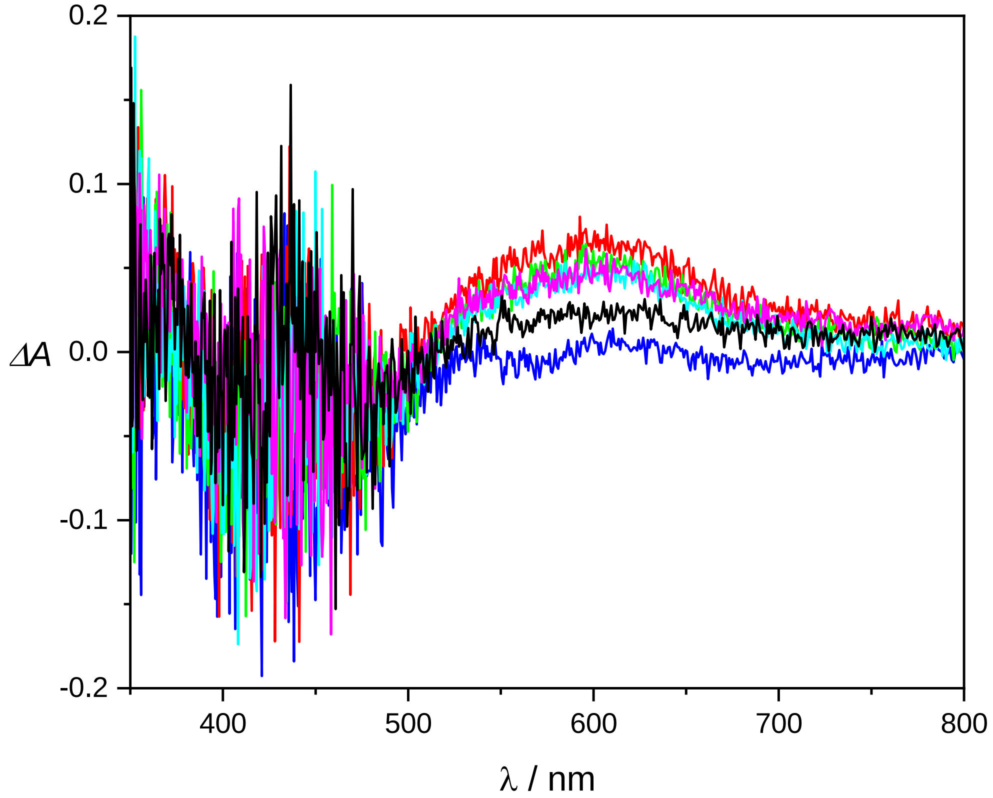

Figure S152. Transient absorption spectra for **7B** (c ~4 × 10^{-5} M, methanol/DMSO, 98:2, aerated, ~1000 equiv. of NaOH) taken after a 355 nm flash at various time delays (blue: 10 ns, red: 100 ns, green: 1 µs, cyan: 10 µs, magenta: 100 µs, black: 1 ms).

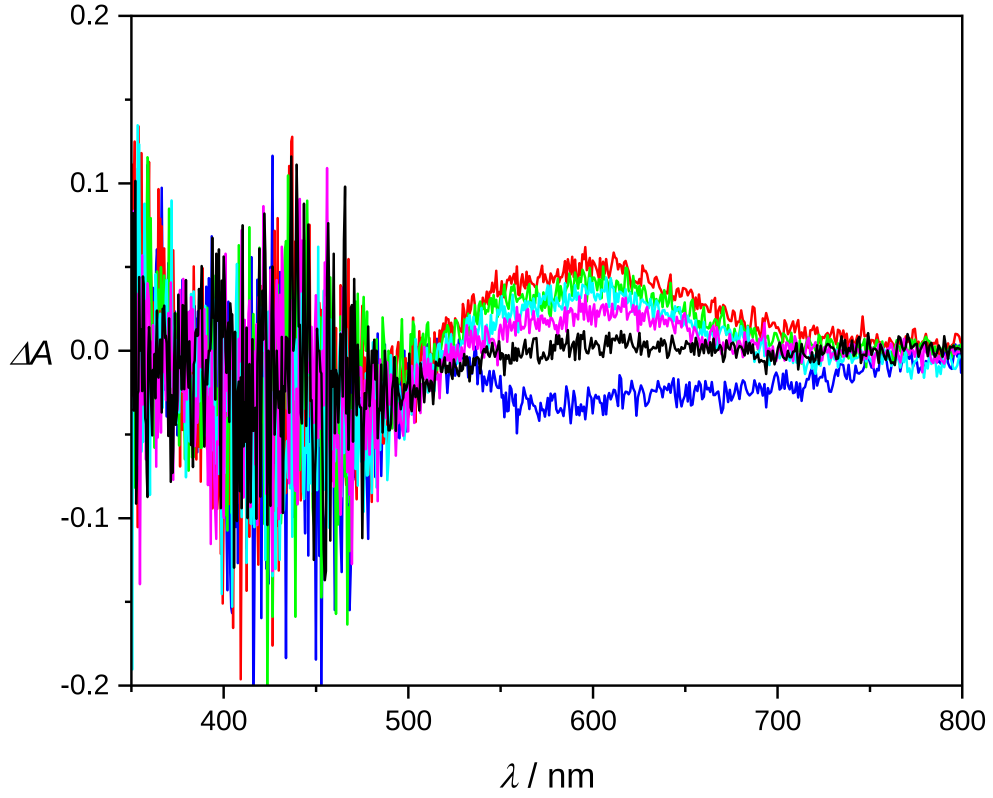

Figure S153. Transient absorption spectra for **7B** (c ~4 × 10^{-5} M, methanol/DMSO, 98:2, degassed, ~1000 equiv. of NaOH) taken after a 355 nm flash at various time delays (blue: 10 ns, red: 100 ns, green: 1 µs, cyan: 10 µs, magenta: 100 µs, black: 1 ms).

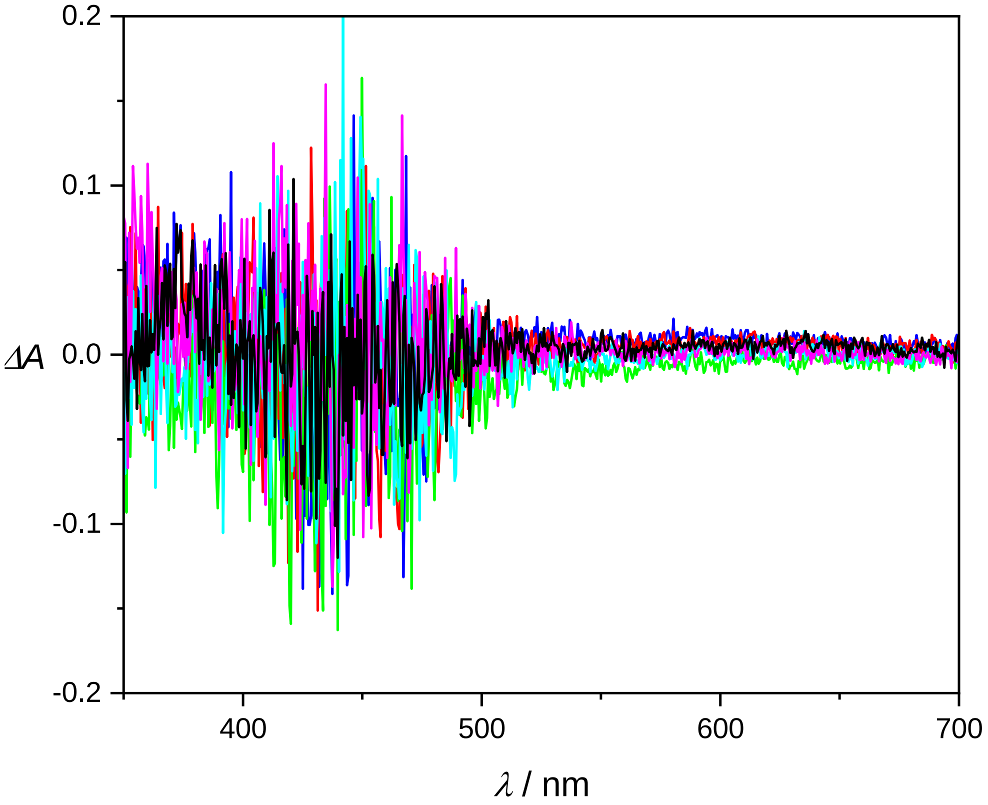

Figure S154. Transient absorption spectra for **12A** (c ~3 × 10^{-5} M, methanol/DMSO, 98:2, aerated) taken after a 355 nm flash at various time delays (blue: 10 ns, red: 100 ns, green: 1 µs, cyan: 10 µs, magenta: 100 µs, black: 1 ms).

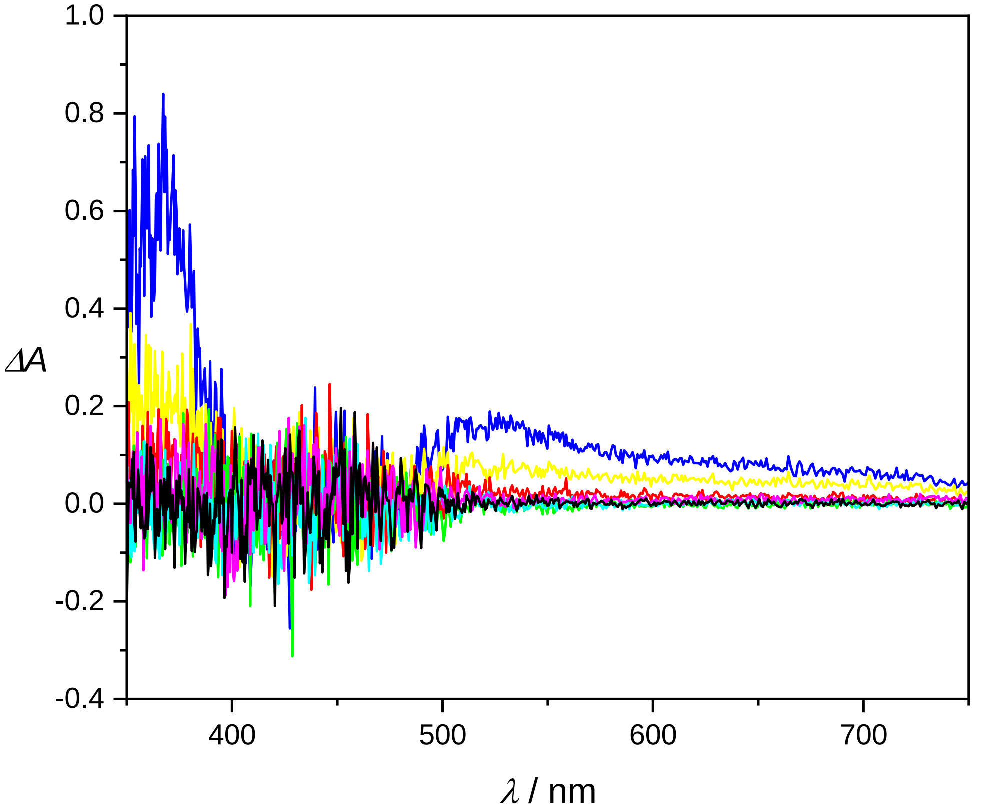

Figure S155. Transient absorption spectra for **12A** (c ~3 × 10^{-5} M, methanol/DMSO, 98:2, degassed) taken after a 355 nm flash at various time delays (blue: 10 ns, yellow: 50 ns, red: 100 ns, green: 1 µs, cyan: 10 µs, magenta: 100 µs, black: 1 ms).

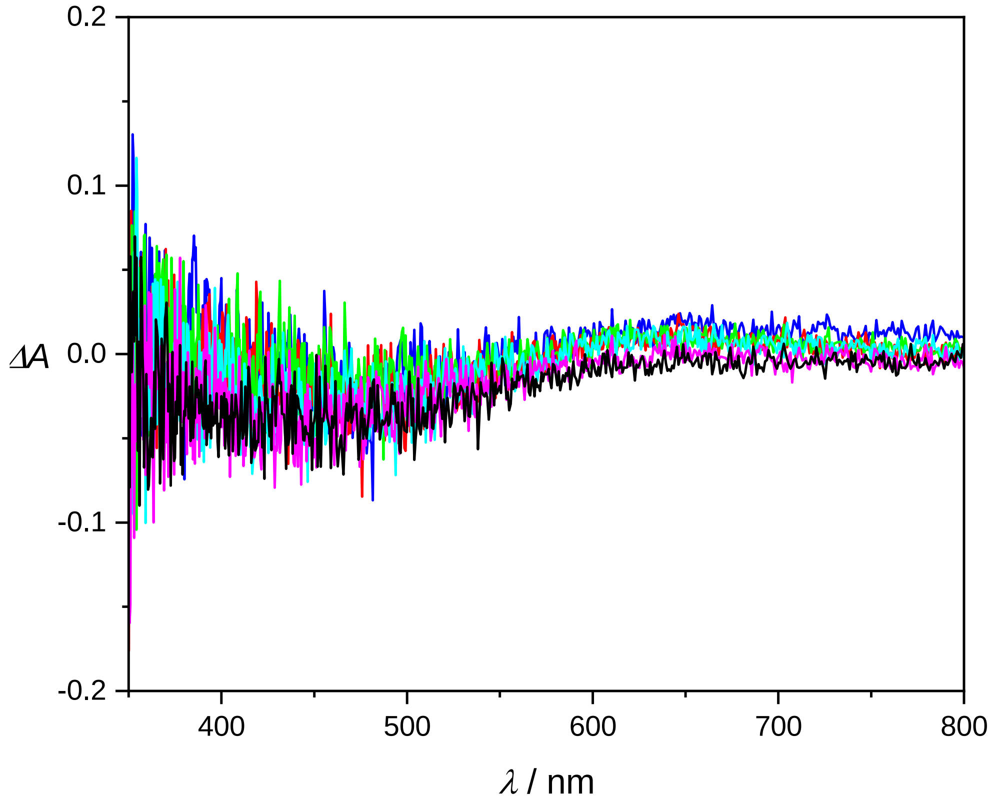

Figure S156. Transient absorption spectra for **12B** (*c* ~3 × 10^{-5} M, methanol/DMSO 98:2, aerated, ~200 equiv. of NaOH) taken after a 355 nm flash at various time delays (blue: 10 ns, red: 100 ns, green: 1 µs, cyan: 10 µs, magenta: 100 µs, black: 1 ms).

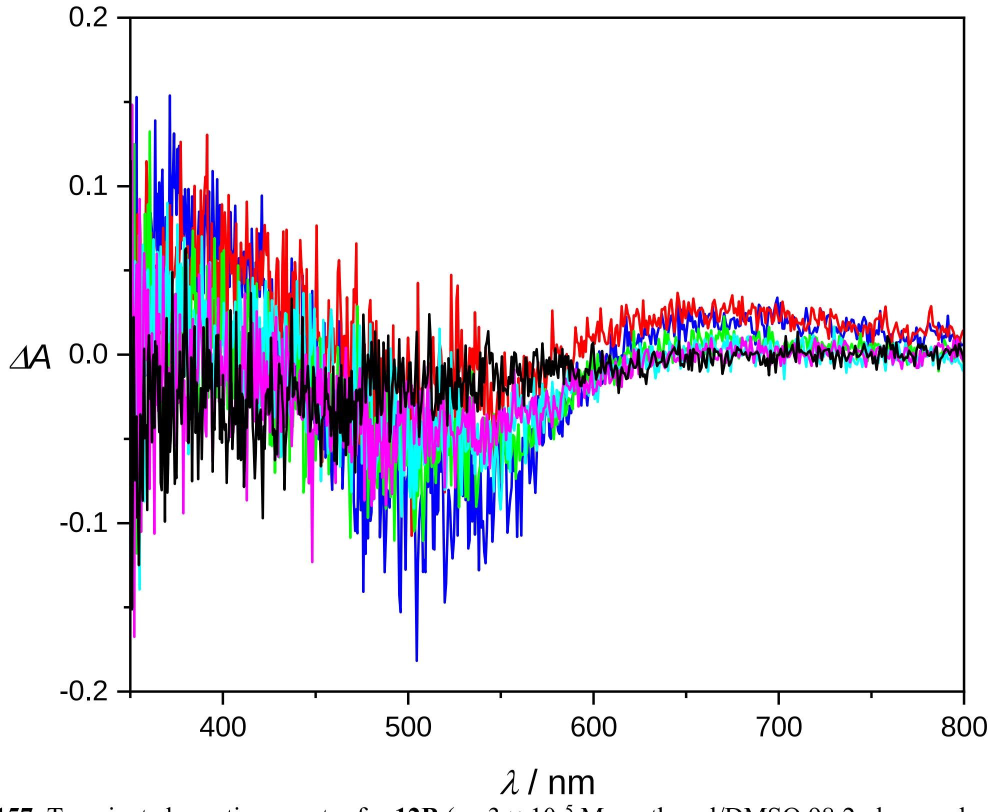

Figure S157. Transient absorption spectra for **12B** (*c* ~3 × 10^{-5} M, methanol/DMSO 98:2, degassed, ~200 equiv. of NaOH) taken after a 355 nm flash at various time delays (blue: 10 ns, red: 100 ns, green: 1 µs, cyan: 10 µs, magenta: 100 µs, black: 1 ms).

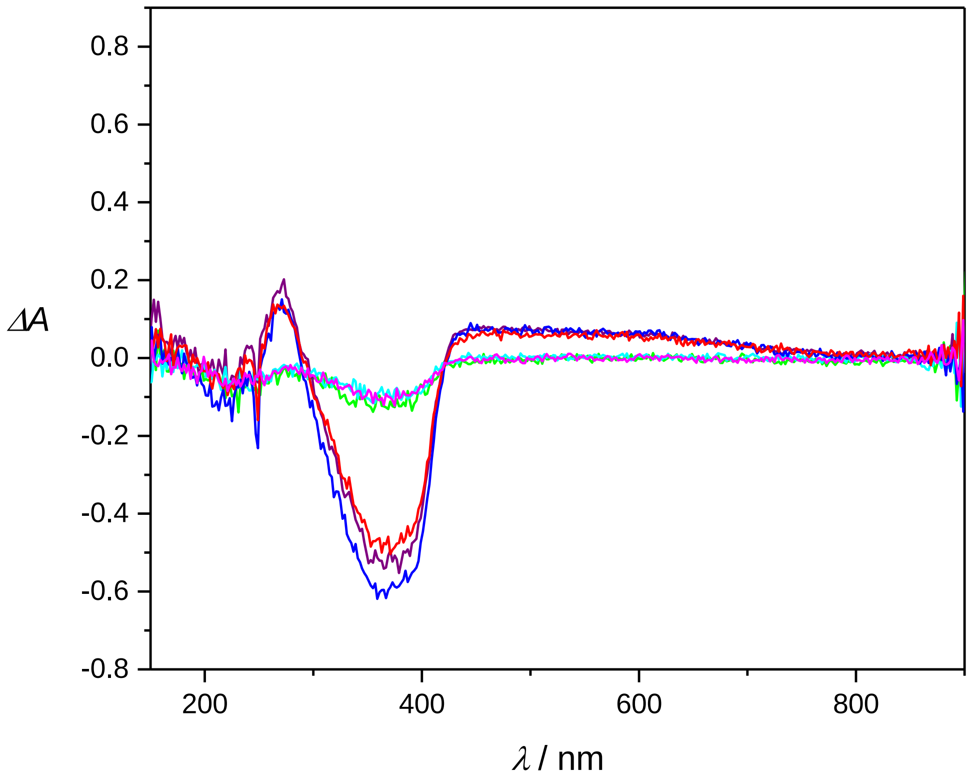

Figure S158. Transient absorption for **13A** (c ~2 × 10^{-5} M, methanol, aerated) taken after a 532 nm flash at various time delay (violet: 5 ns, blue: 10 ns, red: 100 ns, green: 1 μs, cyan: 10 μs, magenta: 100 μs).

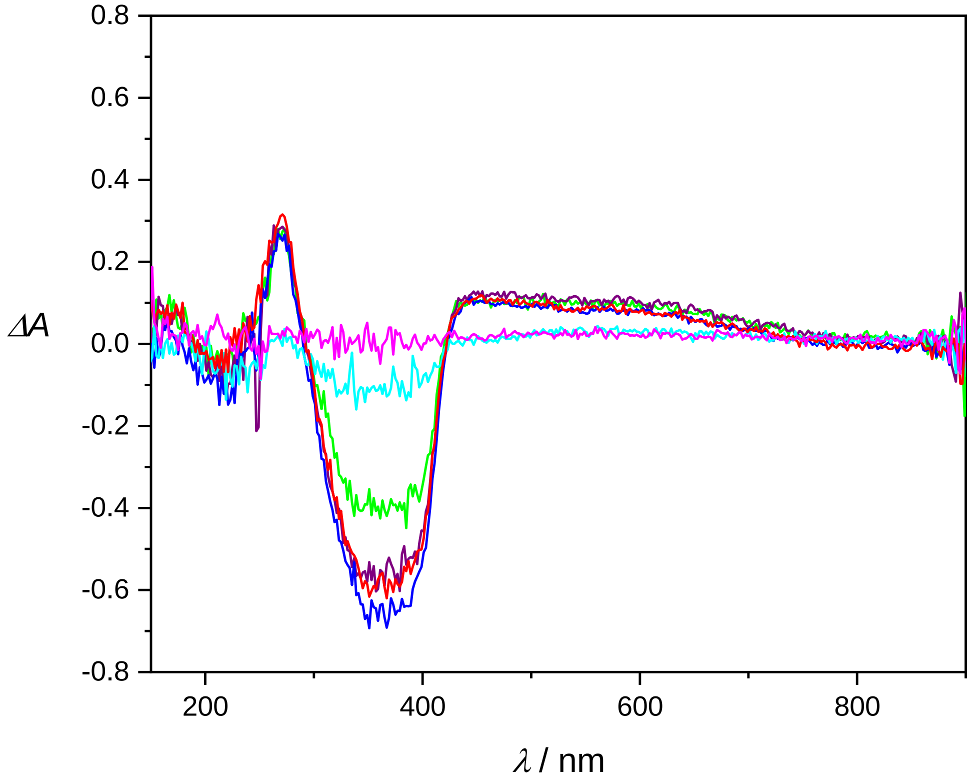

Figure S159. Transient absorption for **13A** (c ~2 × 10^{-5} M, methanol, degassed) taken after a 532 nm flash at various time delay (violet: 5 ns, blue: 10 ns, red: 100 ns, green: 1 μs, cyan: 10 μs, magenta: 100 μs).

Spectroscopic Determination of the pK_a

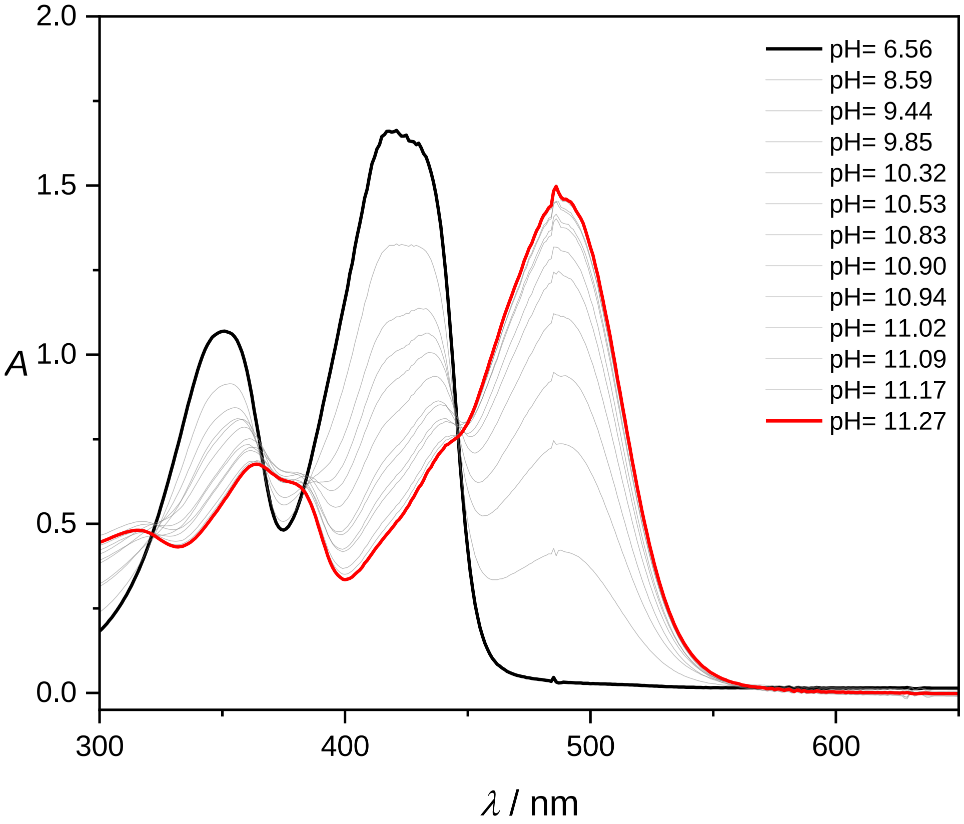

Figure S160. Spectroscopic determination of the pK_a of **13** in methanol/water (50:50). Two different bands can be observed: **13A** (λ_{max} = 420 nm, black), and **13B** (λ_{max} = 490 nm, red). One isosbestic point is visible at λ_{max} = 446 nm.

Sensitivity of Flavothione 13 Toward Singlet Oxygen

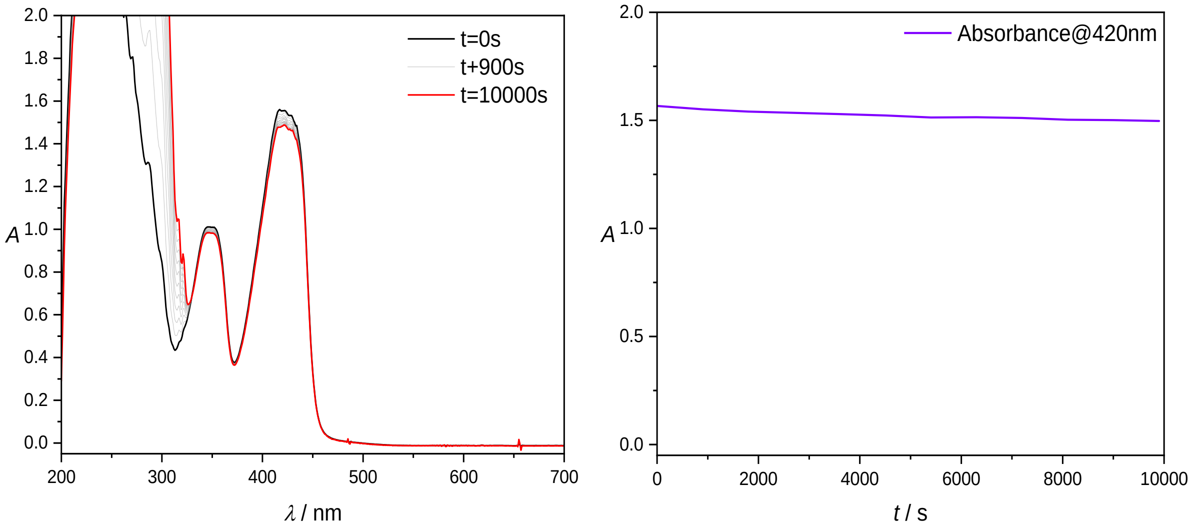

Figure S161. (left) UV-VIS spectra of a methanol solution of **13A** (c ~7.0 × 10^{-5} mol L^{-1}) with 80 equiv. of 1,4-dimethyl-1,4-dihydro-1,4-epidioxynaphthalene kept in the dark for the given time period. (right) The changes of the absorbance at 420 nm during the monitored time.

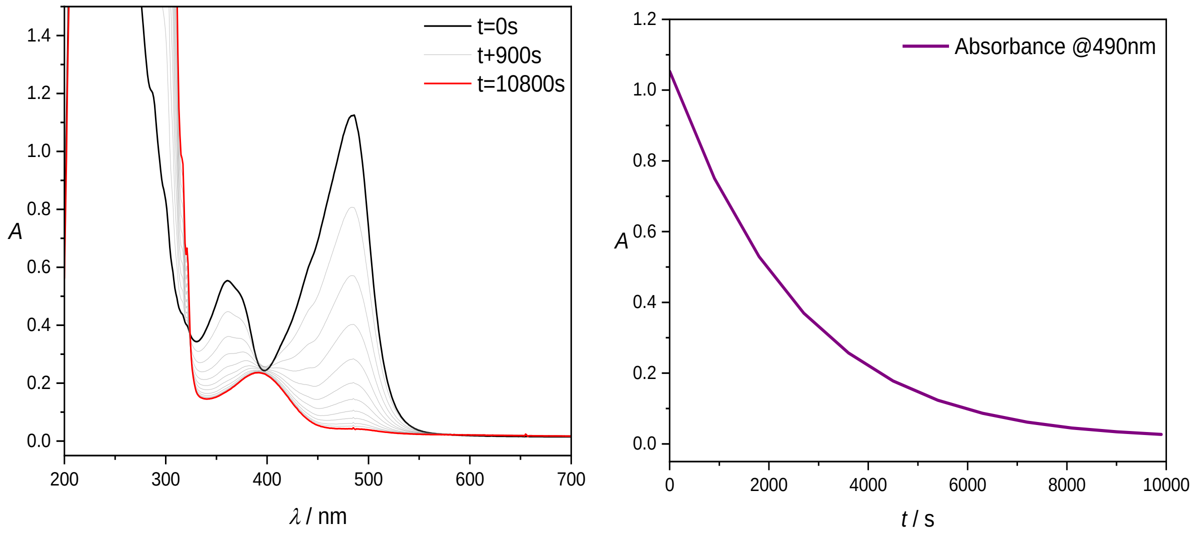

Figure S162. (left) UV-VIS spectra of a methanol solution of **13B** (c ~5.0 × 10^{-5} mol L^{-1}) with 70 equiv. of 1,4-dimethyl-1,4-dihydro-1,4-epidioxynaphthalene kept in the dark for the given time period. (right) The changes of the absorbance at 490 nm during the monitored time.

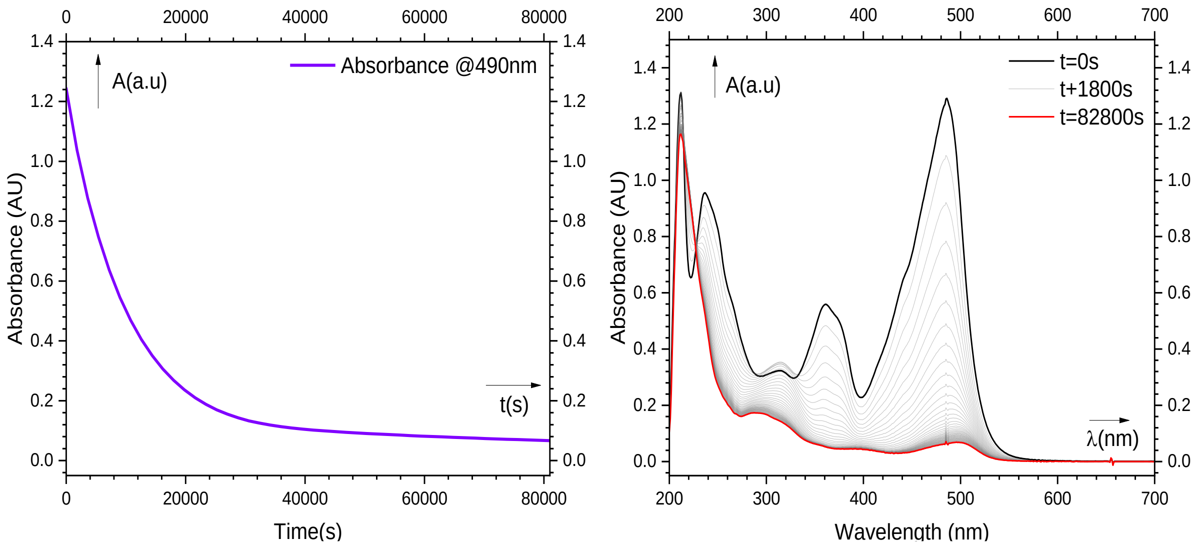

Figure S163. Compound **13B** (c ~5.0 × 10^{-5} mol L^{-1}) in methanol kept in the dark for the given time period: (left) UV-VIS spectra; (right) the changes of the absorbance at 490 nm during the monitored time.

References

(1) Mishiro, K.; Kimura, T.; Furuyama, T.; Kunishima, M. Phototriggered active alkyne generation from cyclopropenones with visible light-responsive photocatalysts. *Org Lett* **2019**, *21*, 4101-4105.

(2) Young, R. H.; Brewer, D.; Keller, R. A. Determination of rate constants of reaction and lifetimes of singlet oxygen in solution by a flash photolysis technique. *J. Am. Chem. Soc.* **1973**, *95*, 375-379.

(3) Saito, I.; Matsuura, T.; Inoue, K. Formation of superoxide ion from singlet oxygen. Use of a water-soluble singlet oxygen source. *J. Am. Chem. Soc.* **1981**, *103*, 188-190.

(4) Gandin, E.; Lion, Y.; Van de Vorst, A. Quantum yield of singlet oxygen production by xanthene derivativesquantum yield of singlet oxygen production by xanthene derivatives. *Photochem. Photobiol.* **1983**, *37*, 271-278.

(5) Haag, W. R.; Gassman, E. Singlet oxygen in surface waters—Part I: Furfuryl alcohol as a trapping agent. *Chemosphere* **1984**, *13*, 631-640.
